# One-pot multi-substrate screening of ligation reactions using PNA tags

**DOI:** 10.1039/d5sc08732e

**Published:** 2026-02-20

**Authors:** Aki Kohyama, Sofia Barluenga, Nicolas Winssinger

**Affiliations:** a Department of Organic Chemistry, Faculty of Sciences, University of Geneva Geneva 12004 Switzerland koyama.aki.6a@kyoto-u.ac.jp Nicolas.Winssinger@unige.ch

## Abstract

Chemical ligation is an essential tool for constructing complex biomolecular architectures. To accelerate reaction discovery, a one-pot multi-substrate screening (OPMSS) platform was developed, combining peptide nucleic acid (PNA) tagging with direct MALDI analysis. This approach enables the simultaneous evaluation of multiple substrate pairs in a single pot without the need for chromatographic separation. Short PNA tags promote a uniform combinatorial pairing of substrates while the neutral polyamide backbone facilitates MALDI analysis to allow direct readout of ligated products as predominantly singly charged ions. Using this system, we readily detected established ligations, including Huisgen cycloaddition and amide bond formation, validating the platform in pilot screens pairing 8 × 8 substrates (64 possible combinations). Applying the method to discovery-mode screening of 13 × 11 substrates under visible-light photocatalytic conditions identified a previously unexplored ligation between alkyl azides and alkenes, consistent with pathways involving aminyl radicals or aminium radical cations. This work demonstrates the potential of OPMSS with PNA tagging as a practical and discovery-oriented approach for identifying new ligation reactions directly from complex mixtures.

## Abstract 2

Chemical ligation is an essential tool for constructing complex biomolecular architectures.

## Graphical abstract

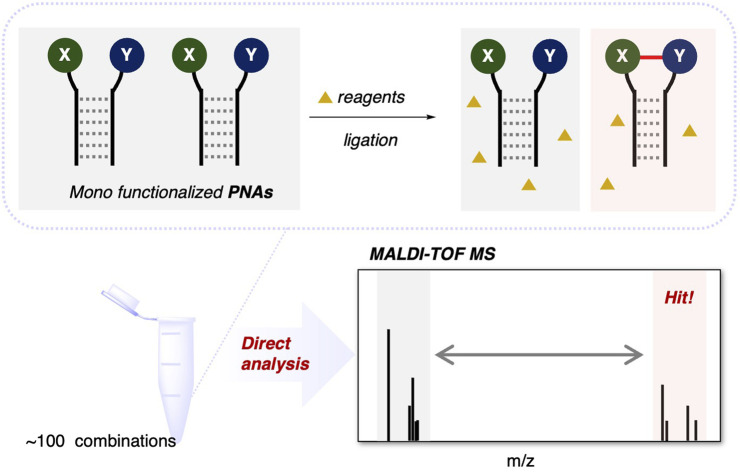

## 1 Introduction

Chemical ligation has proven to be a powerful tool in medicinal chemistry, chemical biology, and the synthesis of macromolecules.^1–3^ The discovery of new ligation chemistries, however, often relies on serendipity or the execution of a large number of experimental trials. To accelerate reaction discovery, two modern approaches—high-throughput experimentation (HTE) and one-pot multi-substrate screening (OPMSS)—have been developed, supported by advanced analytical technologies. These platforms allow a large number of reactions to be performed and analysed within a short time compared with traditional one-to-one methods.

HTE, which typically employs multi-well plates, significantly increases experimental throughput, although it requires the individual preparation and analysis of each reaction mixture.^4–10^ This approach often requires automated equipment to handle the repetitive manipulations involved. In contrast, OPMSS allows many combinations of substrates to be examined within a single solution, thereby greatly accelerating reaction discovery by generating a diverse dataset.^11–13^ A major advantage of OPMSS is its minimal number of manipulations compared with HTE. However, this approach suffers from a bottleneck—a simultaneous analysis of hundreds to thousands of reactions.

**Fig. 1:**
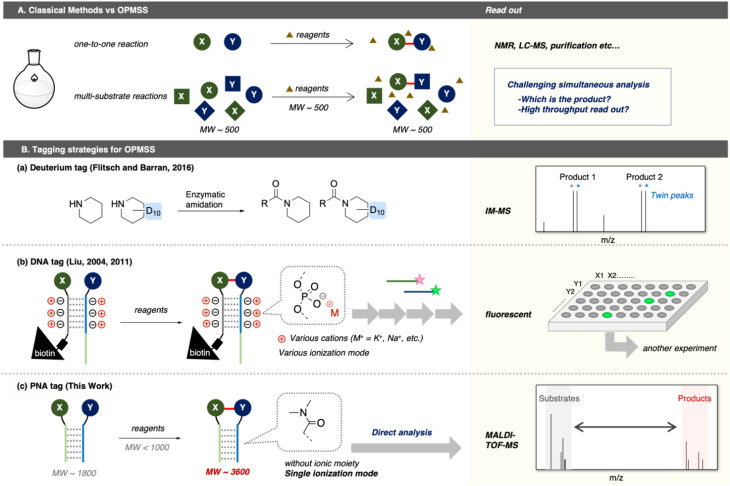
Methods for reaction discovery. (A) Classical methods *versus* OPMSS, (B) Tagging strategies for OPMSS: (a and b) previous reports, (c) this work.

In small-molecule reactions, most reaction components (substrates, reagents, products, and side products) possess similar molecular weights (MWs) around 500 (Fig. 1A). Consequently, unexpected products generated in OPMSS cannot be directly identified by conventional mass spectrometry by falling within a narrow *m*/*z* window (∼500 Da). This analytical challenge can be overcome through the introduction of suitable tagging strategies. For instance, a deuterium tag provides twin peaks in ion mobility mass-spectrometry (IM-MS) analysis, serving as clear landmarks to assist product identification (Fig. 1B_a).^14^ Such tagging approaches have also been applied to evaluate deuteriation reactions in other OPMSS systems.^15^ A fluoride tag combined with ^19^F-NMR spectroscopy and a chiral NMR shift reagent enables simultaneous determination of reaction yields and enantiomeric excesses of 21 asymmetric aminations.^16^ Historically, OPMSS has been widely used to optimise asymmetric catalytic reactions across a broad substrate scope. In contrast, OPMSS for discovering novel ligation reactions of two different substrates remains limited due to the three inherent challenges to reaction discovery:

(1) Substrates must selectively react with their pairs without undesired cross reactivity.

(2) Competitive reactions must be minimized to avoid false positives; and

(3) Ligated products must be efficiently identified even when their structure is unanticipated.

To meet these requirements, several tag-free OPMSS systems have been reported. One example employed two substrate pools, each containing four or five substrates, followed by GC-MS or ESI-MS readout to identify new reactions.^17^ The workflow includes second and third round screening steps. Although successful in discovering three novel reactions, this approach relies on careful substrate and reagent design, accurate product prediction, and a limited substrate number per pool to avoid analytical complexity.

DNA tagging offered a more powerful OPMSS platform, combining DNA-templated reactions with fluorescence-based microarray readouts^18,19^ or next-generation DNA sequencing^20^ (Fig. 1B_b). DNA-templated systems inherently minimize unproductive interactions while maximizing productive encounters between complementary substrates,^21,22^ thereby satisfying the three aforementioned criteria. While DNA tagging is powerful, it often necessitates a costly, multistep workflow involving linker cleavage and amplification prior to readout. Furthermore, long DNA strands augment the risk of interference in the reaction and hybridization buffers for substrates restrict the reaction conditions that can be practically screened.

Given these limitations, we envisioned that a simpler and more accessible approach would greatly enhance the general applicability of OPMSS. Fundamentally, reaction mixtures containing highly functionalized DNA tags are difficult to analyse directly by MALDI due to multiple ionization modes arising from the negatively charged phosphates. In contrast, uncharged peptide nucleic acid (PNA) tags, which exhibit a uniform ionization behaviour, should facilitate direct MALDI analysis (Fig. 1B_c). We therefore hypothesised that PNA tagging could enable the direct and simultaneous analysis of multi-substrate ligations in a single pot. In principle, MALDI–TOF MS provides a straightforward spectral separation between reagents (MW < 1000), substrates (MW ≈ 1800) and products (MW ≈ 3600), as the mass gap between substrate and product regions (Δ*m*/*z* > 1000) prevents overlap. This allows reaction monitoring without chromatographic separation, greatly shortening analysis time.^23^ Moreover, PNA tags retain the templating advantage of DNA while offering reliable pseudo-intramolecular reaction environments between substrates X and Y.^24^ Such a templated system allows proximity-enabled reactions, which has been widely utilized in chemical biology and covalent drug development^25^ in addition to normal chemical reactions. MALDI analysis, compared with GC-MS or LC-MS, further offers time efficiency and scalability benefit.^8^ Herein, we report a novel OPMSS based on PNA tagging combined with direct MALDI analysis of multi-substrate reactions. Here, MALDI–TOF MS was used for rapid molecular weight – based identification of ligated products, rather than for definitive structural elucidation.

**Fig. 2:**
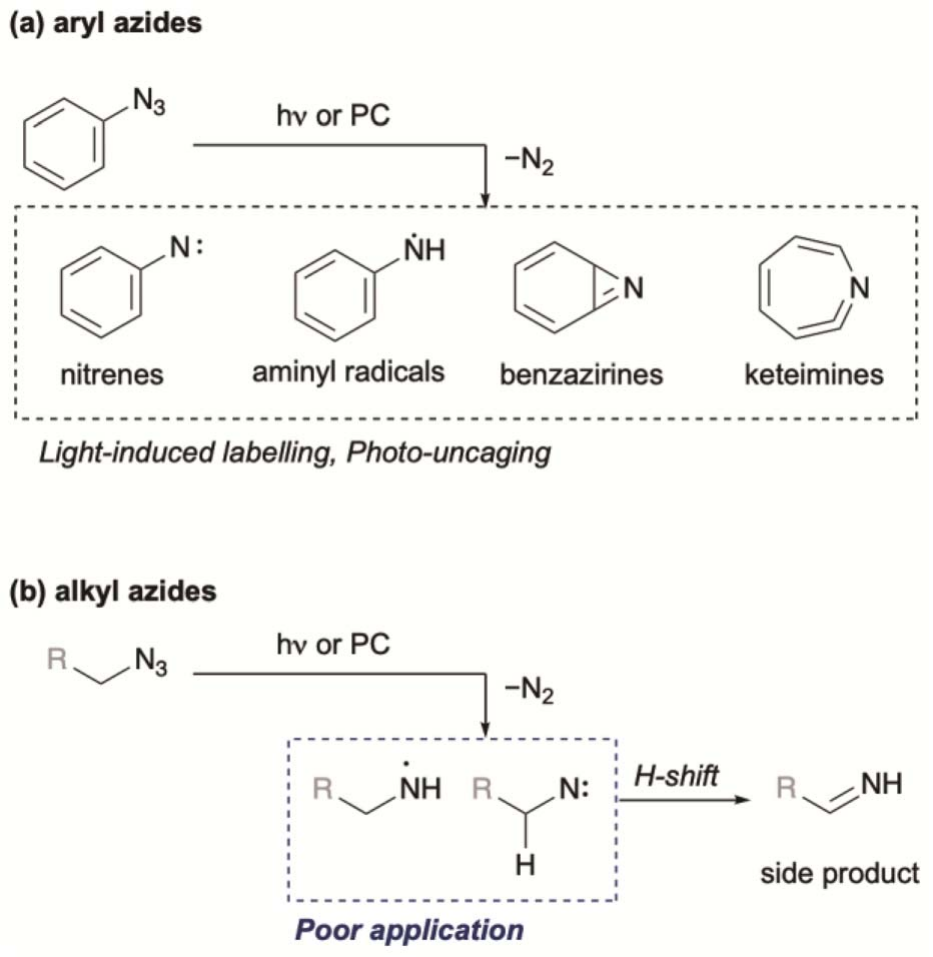
Photo-triggered reactions from (a) aryl azides or (b) alkyl azides.

Organic azides are ubiquitous and biocompatible functional groups widely employed in Huisgen cycloadditions, Staudinger–Bertozzi ligations, and as amine precursors.^26^ Aryl azides, in particular, have been extensively studied as photo-triggered tools in biological experiments.^27^ Upon ultraviolet (UV) irradiation, aryl azides form highly reactive singlet nitrenes that undergo X–H insertion (X = C, N, O) or rearrange into other reactive intermediates such as benzazirines and ketenimines (Fig. 2a).^28^ Visible-light photocatalysis can also transform aryl azides into aminyl radicals and nitrenes.^19,27,29–31^ These reactive intermediates give a scaffold for photolytic or photocatalytic proximity protein labelling. In contrast, alkyl azides have been less explored for photo-triggered applications owing to their lower extinction coefficients,^32^ lower reactivity,^33^ and tendency to decompose to imines (Fig. 2b).^34–37^

**Scheme 1:**
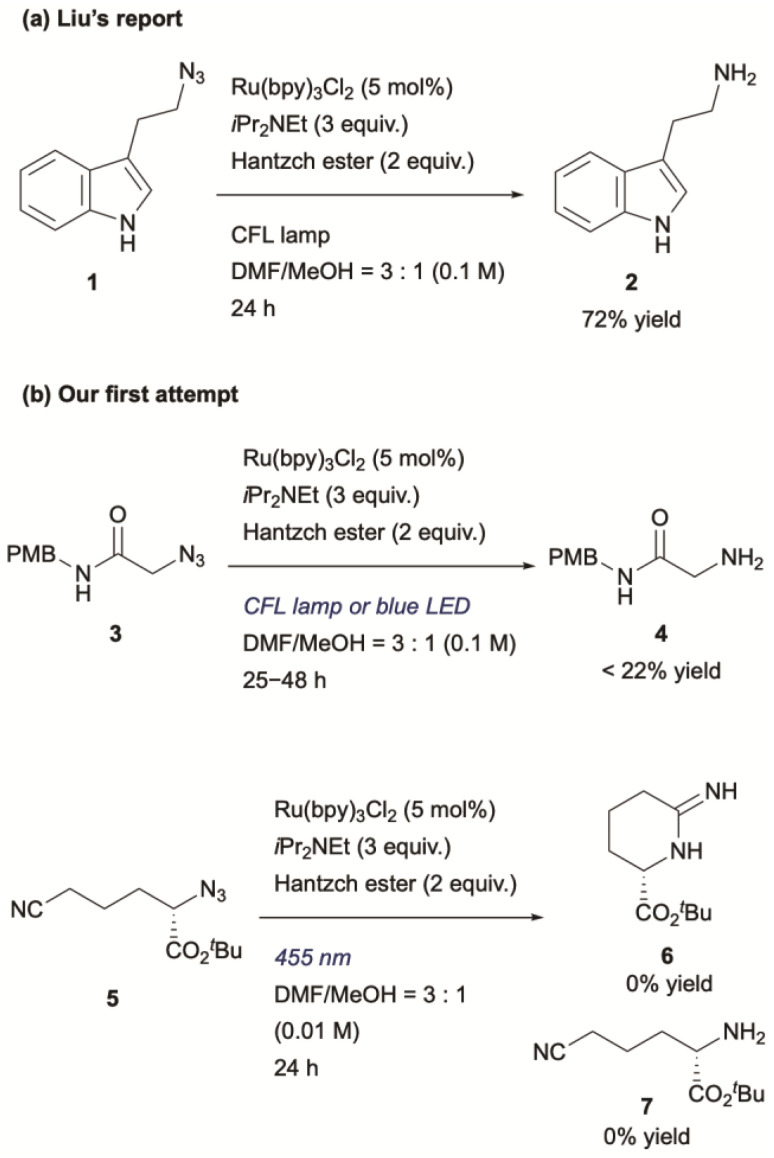
(a) Liu's report and (b) our initial attempt.

To utilize the highly reactive nitrogen-centred radicals (NCRs), intramolecular trapping^38^ or metal trapping^39^ are required. In other words, biocompatible photocatalytic intermolecular bond formation involving alkyl azides remains particularly challenging. To date, one example of biocompatible photocatalytic intermolecular bond formation of alkyl azides has been reported.^19^ In that DNA-templated system, Liu and co-workers demonstrated that aminyl radicals generated from alkyl azides could react with norbornene. However, their final experiment using small molecules demonstrated only the photocatalytic reduction of alkyl azides, suggesting that NCRs might not participate in bond formations with alkenes under those conditions (Scheme 1a). To evaluate this chemistry, we initially applied similar reaction conditions to azide 3 (Scheme 1b) using catalytic Ru(bpy)_3_Cl_2_, *i*Pr_2_NEt, and Hantzsch ester under compact fluorescence lamp (CFL) irradiation. The reaction afforded amine 4 in less than 22% yield, and an intramolecular trapping attempt with azide 5 failed to produce the expected cyclized product 6.^40^ These unsatisfactory results motivated us to explore photoredox-catalyzed bond formation of alkyl azides as a potential new ligation reaction.

Our objectives in this study were therefore: (i) to establish a new OPMSS platform capable of identifying novel ligation reactions, and (ii) to discover a previously unknown visible-light-triggered chemical ligation of alkyl azides. We focused particularly on photocatalytic conditions employing visible light, given their compatibility with biomolecules and their ability to selectively activate alkyl azides. Alkyl azides are chemically more stable than aryl azides, while NCRs from alkyl azides are expected to display higher reactivity than their aryl counterparts—making them interesting, even if challenging candidates for photo-triggered ligations.

## 2 Results and discussion

The rate of templated reactions can depend on the length of the PNA sequences. For example, a 4-mer may not provide sufficient pairing lifetime for the reactants, resulting in an overall reaction rate far below the maximum (*v*_max_). In contrast, increasing the PNA length from 5-mer to 7-mer led to an increase in the apparent reaction rate.^41,42^ Based on these findings, we designed 6-mer PNA sequences (PNA-X: GCGGCG, PNA-Y: CGCCGC) as substrates for OPMSS. While these sequences are palindromic sequences, the preferred anti-parallel alignment should predominate in the hybridization equilibria since hybridization of these short sequences remains dynamic.^43^ These palindromic sequences benefit from requiring only two PNA monomers. During synthesis, linkers composed of fewer than five Gly were used for PNA-Y, because longer linkers were found to reduce PNA solubility. DAP and Lys linker were selected for PNA-X as representing different conformation and reactivity. We prepared 17 PNA-X and 19 PNA-Y bearing different linkers and 25 distinct functional groups (*e.g.* alkyl azides, carboxylic acids, and amino acids) (SI, Tables S2_1, and S2_2). Each functional group and linker were attached to the C-terminal end of PNA-X or N-terminal end of PNA-Y. Using these substrates, PNA pools were constructed for subsequent OPMSS.

### 2.1. Control experiments

**Fig. 3:**
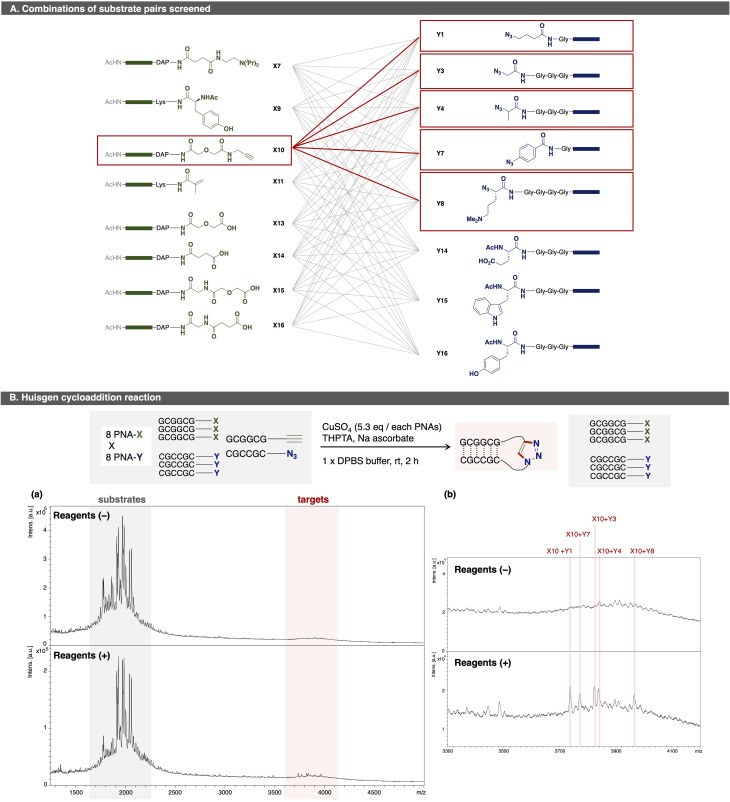
(A) Combinations of substrate pairs screened. Gray lines indicates examined pairs; red lines, hit pairs. Green boxes indicate PNA sequence “GCGGCG”; Blue boxes, “CGCCGC”. (B) Huisgen cycloaddition reaction among 64 substrate pairs catalysed by Cu(ii). (a) MALDI spectra of reaction mixtures. (b) Expanded spectra. Upper: without reagents; lower: with reagents. Pink lines indicate the ligation products. Spectra were scaled relative to the base peak. Reaction conditions: eight PNA-Xs (5.0 µM x 8), eight PNA-Ys (5.0 µM x 8), CuSO_4_ (0.43 mM, 85 equiv. per PNA alkyne), THPTA (4.3 mM), Na ascorbate (2.1 mM), 1× DPBS buffer.; reaction scale: 100 pmol per each PNA; 0.10 nmol of PNA was used for one analysis.

As a proof of concept for OPMSS using PNA tags, Huisgen cycloaddition and condensation reaction were examined as reliable model reactions. First, Huisgen cycloaddition was evaluated using 64 combinations of PNA pairs containing one alkyne-PNA-X and five azide-PNA-Ys (Fig. 3).^44^ As expected, five distinct ligated products were detected after 2 h of incubation. Although background peaks corresponding to [2M + H]^+^ species of the substrates were observed due to strong laser intensity used for detection, these signals do not affect the detection of a ligation product. This result showed that more than 50 PNA pairs can be screened in a single microtube, and that MALDI-based readout is suitable for small-scale (pmol-level) OPMSS screening.

**Fig. 4:**
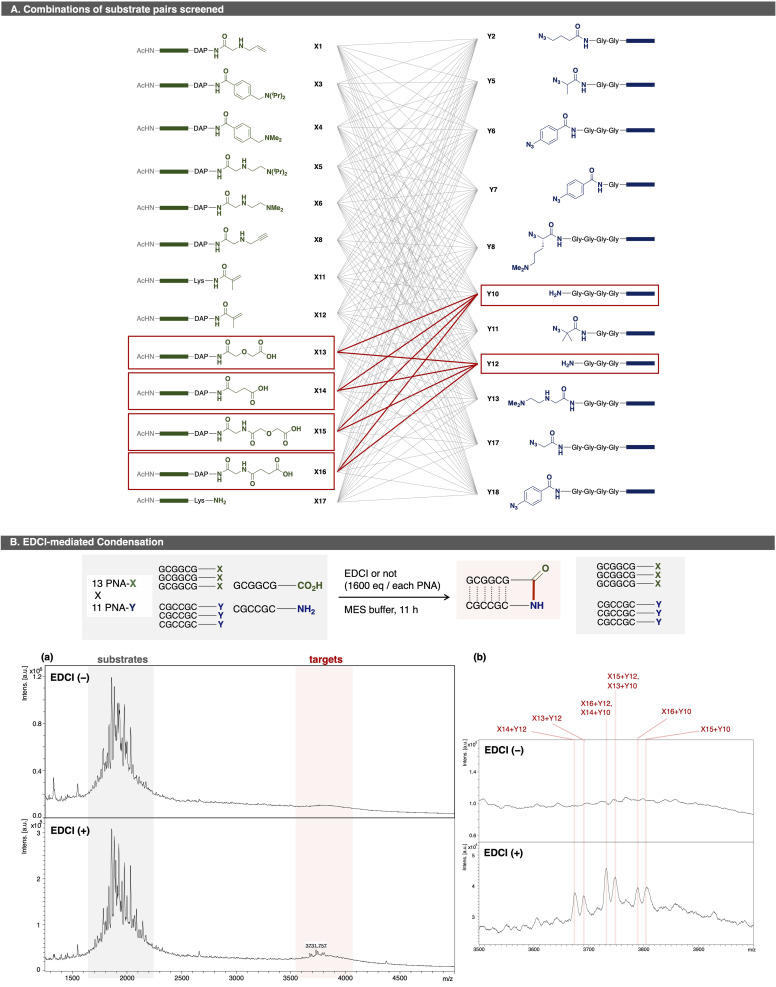
(A) Combinations of substrate pairs screened. Gray lines indicates examined pairs; red lines, hit pairs. Green boxes indicate PNA sequence “GCGGCG”; Blue boxes, “CGCCGC”. (B) EDCI-mediated condensation among 143 substrate pairs. (a) MALDI spectra of reaction mixtures. (b) Expanded spectra. Upper: without EDCI. Lower: with EDCI. Pink lines indicate the ligation products. Spectra were scaled relative to the base peak. Reaction conditions: thirteen PNA-Xs (5.0 µM × 13), eleven PNA-Ys (5.0 µM × 11), EDCI (0.20 M, 1.3 × 10^4^ equiv. per PNA-CO_2_H), 1xMES buffer : DMF = (2 : 1). Reaction scale: 100 pmol per each PNA; scale for analysis: 0.10 nmol of PNA was used for one analysis.

Next, a condensation reaction was evaluated using 143 PNA pairs comprising 13 PNA-X and 11 PNA-Y species (Fig. 4). Among them, four PNA-X (X13–X16) carried a carboxylic acid at C-terminus, while two PNA-Y (Y10, Y12) carried an amine at N-terminus. As expected, eight ligated products were detected with signal intensities higher than background peaks. These results confirmed that more than 64 PNA pairs can be screened in a single microtube by OPMSS. An inherent challenge of multi-pair screening is that the yield of any individual ligation is low relative to a given substrate since only a subset of the pairings undergo a product reaction. Despite this limitation, the products are clearly identifiable in linear and reflector mode (Fig. S3-2-2).

### 2.2. Reaction discovery using OPMSS

**Fig. 5:**
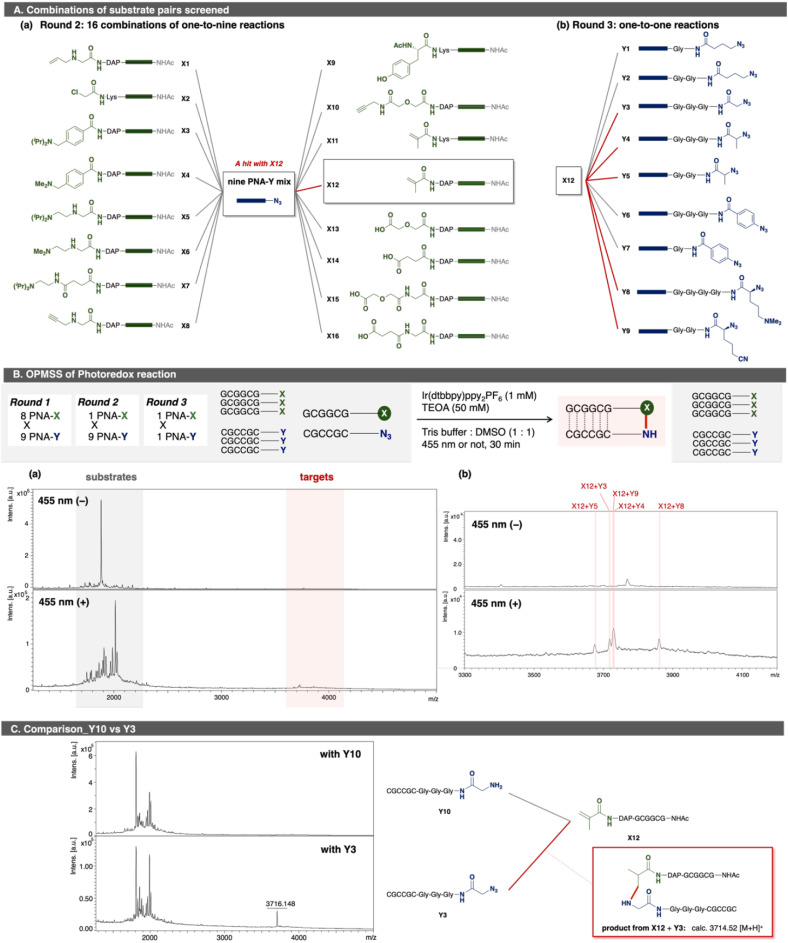
(A) Combinations of substrate pairs screened in (a) round 2, and (b) round 3. Green boxes indicate PNA sequence “GCGGCG”; blue boxes, “CGCCGC”. Gray lines indicates examined pairs; red lines, hit pairs. (B) OPMSS of Photoredox reaction. (a) MALDI spectra of the reaction mixture of X12 and nine PNA-Y (Y1–Y9): round 2. (b) Expanded spectra. Upper: 455 nm irradiation (−). Lower: 455 nm irradiation (+). Pink lines indicate the ligation products. (C) Comparison of photoredox reaction of X12 and Y10 or Y3. Spectra were scaled relative to the base peak. Reaction conditions in round 2: one PNA-X (75 µM), nine PNA-Ys (7.5 µM × 9), [Ir(dtbbpy)(ppy)_2_]PF_6_ (1.0 mM, 15 equiv. per PNA-N_3_), 1 M triethanol amine aq. (50 mM, 7.4 × 10^2^ equiv. per PNA-N_3_), 1.0 M Tris buffer : DMSO = 1 : 1, 455 nm irradiation; reaction scale: 150 pmol per each PNA; scale for analysis: 1.5 nmol of each PNA was used for one analysis.

Encouraged by these positive results, we sought to discover new reactions involving active species derived from azides. The workflow was as follows: (1) screening of reaction conditions to form active species, NCRs, from azides by using several PNA-Ys; (2) applying the optimized conditions to 72 PNA pairs, comprising PNA-Xs functionalized with complementary reactive groups and PNA-Ys bearing various azide-containing functional groups; (3) testing multi-to-multi PNA reactions to validate possible hits; and (4) performing multi-to-one reactions and the following one-to-one reactions to confirm findings outside of OPMSS format. In the initial screening, two photoredox conditions were found to reduce azides (Y1, Y3, and Y4) to amines, presumably *via* NCRs (SI 3.3.3.1). Both conditions used [Ir(dtbbpy)(ppy)_2_]PF_6_ as a photoredox catalyst and triethanol amine (TEOA) or tetramethylethylenediamine (TMEDA) as reducing agents under 455 nm irradiation. Using these two conditions, two sets of photoredox reactions comprising 72 PNA pairs (8 PNA-X and 9 PNA-Y) were examined in one pot (SI 3.3.3.2). Although MALDI–TOF MS enables rapid molecular weight readout, reliable mass identification becomes challenging under highly pooled conditions when ligation efficiency is low and background signals dominate. In the initial round 1 screening, this limitation prevented unambiguous mass assignment of ligated products (Fig. S3-5-2). Given the vast array of chemistries possible with different NCRs, results were validated with smaller pools (Fig. 5A and B). PNA X12 uniquely afforded some ligated products and recapitulated the results obtained from the larger screen (round 2). Notably, PNA X11, which shares an acrylamide moiety with X12, produced no ligated products, suggesting that linker length affects ligation efficiency, as exemplified in reductive amination.^45^ In the reaction of PNA X12 with nine PNA-Ys, four clearly discernible ligated products were observed, with intensities stronger than background peaks (Fig. 5B and S3-7-12). These peaks corresponded to five ligated products between X12 and Y3, Y4, Y5, Y8, or Y9. Round 2 enabled confident mass identification of these targets, whereas round 1 did not. This outcome is likely attributable to the intrinsically low yield of the newly identified ligation under OPMSS conditions, in contrast to the higher efficiencies observed for Huisgen cycloaddition and condensation. Subsequent one-to-one reaction confirmed that each observed product from OPMSS was indeed the product of unique substrate pairs (Fig. S3-8). Given the reaction conditions, we hypothesized that NCRs generated from azides reacted with acrylamide X12. To test this, we replaced Y3 with its reduced analog Y10 and examined the same reaction (Fig. 5C). No ligated product was observed, ruling out an aza-Michael addition mechanism, suggesting a radical-mediated process.

### 2.3. Photo-triggered reaction of azides with 1,1-diphenylethylene

Finally, the newly discovered reaction was validated using small-molecule analogues. Similar to the PNA-templated reaction between Y3 and X12, a photo-triggered reaction between azides and acrylamides was examined. Initially, an excess amount of photoredox catalyst (3.3 or 15 equiv.) and high dilution (75–300 µM), as used during the screen, was required to establish suitable conditions for small-molecule reactions. However, under these conditions, only trace amounts of the ligated product were obtained, with amines from azides as the main products (Scheme S2). This outcome indicated that changing from PNA-templated reactions to non-templated reaction caused the loss of proximity effect and the optimized microenvironment. To identify better partners for the activated azides, we rescreened several alkenes and found that 1,1-diphenylethylene reacted effectively with azide 3 under optimized photoredox conditions (Scheme S3, and Table 1). We also examined various reducing agents, as water solubility (important for PNA reactions) was unnecessary in this context. Among those tested, *N*-methyl morpholine (NMM) gave the highest yield (61%) of ligated product 8 in MeCN (entry 8). In contrast, *i*-PrNEt_2_, Et_3_N, and TMEDA primarily produced reduced product 4 (entries 2–4), while quinuclidine led to incomplete conversion (entry 1). TEOA and BnNMe_2_ afforded moderate yields (36% and 41%; entries 6, 7). Adding water slightly decreased the yield with NMM (48%; entry 8), but TEOA gave the best yield (80%; entry 9). The optimized conditions exhibit several advantages: applicability to alkyl azides, short reaction time, and tolerance to dilution. The main limitation is the need for excess 1,1-diphenylethylene. Considering the effectiveness of TEOA, we compared the pK_BH_ values of the amine reductants. TEOA and NMM, both having relatively low pK_BH_ (<8), correlated with higher yields of ligated product.

**Table 1:** Screening of reducing agents

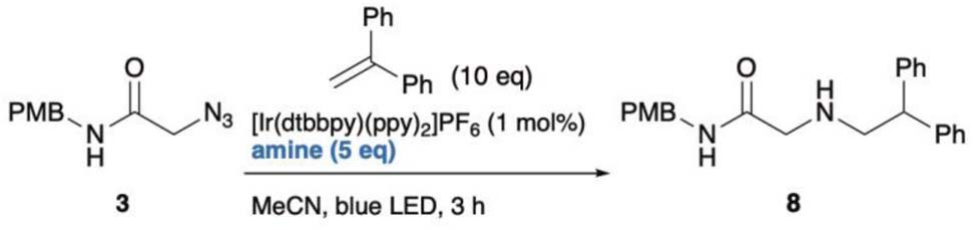			
Entry	Reducing agents	pK_BH_	Yield^a^ of 8 (%)
1	Quinuclidine	11.3	6
2	*i*-Pr_2_NEt	10.9	31
3	TMEDA	9.2	16
4	Et_3_N	9.0	22
5	DABCO	8.7	15
6	TEOA	7.7	44
7	BnNMe_2_	7.6	36
8	NMO	7.4	61
9	1 M TEOA aq.	7.7	80
10	1 M NMO aq.	7.4	48

a HPLC yields.

**Fig. 6:**
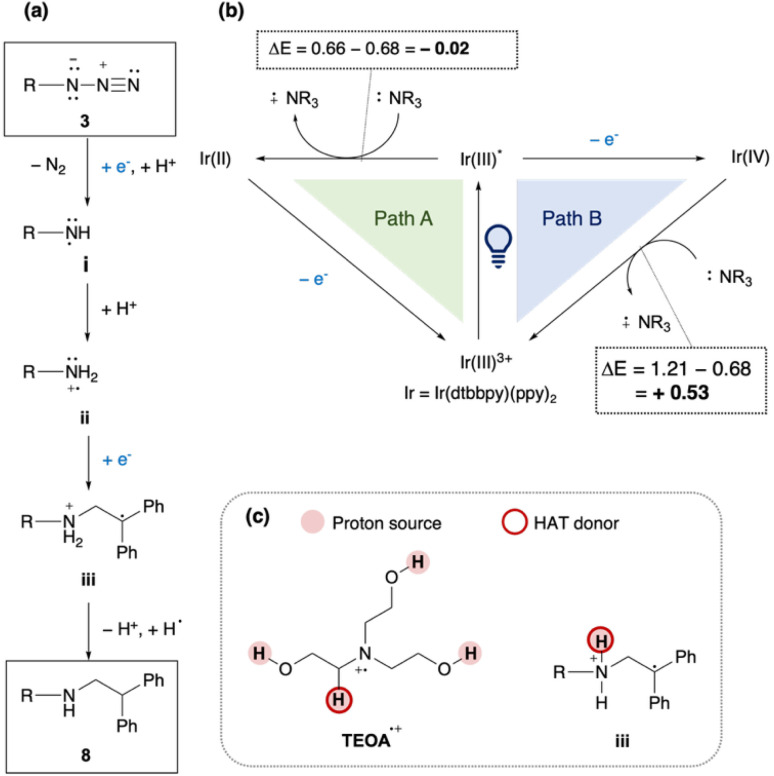
Proposed reaction mechanism. (a) Formation of product 8 from 3. (b) Redox cycle by the Ir catalyst and reducing agents. (c) Possible proton sources and HAT donors; Redox potentials of Ir(dtbbpy)(ppy)_2_PF_6_ and *i*-PrNEt are shown as follows;^46,47^*E*_ox_ (Ir^III^*/Ir^II^) = + 0.66 V *vs.* SCE, *E*_ox_ (Ir^IV^/Ir^III^) = + 1.21 V *vs.* SCE, *E*_ox_ (*i*-PrNEt^*Σ*+^/*i*-PrNEt) = + 0.68 V *vs.* SCE.

Based on these findings, a plausible mechanism is proposed (Fig. 6). (i) Azide 3 accepts one electron and one proton, releasing N_2_ to form the aminyl radical i. (ii) Protonation of i affords an aminium radical cation (ARC) ii, which reacts with 1,1-diphenylethylene to generate radical intermediate iii. (iii) Hydrogen atom transfer (HAT) and protonation of iii then yield product 8. In this mechanism, Ir(ii) or Ir(iii)* can serve as the electron donor (Fig. 6b). In Path A, photo-excited Ir(iii)* is reduced by TEOA to Ir(ii), which then transfers an electron to the substrate or intermediates. In Path B, Ir(iii)* directly reduces the substrate, and the resulting Ir(iv) is subsequently reduced by TEOA. Redox potential analysis^48,49^ indicated that Path A is an endergonic process (Δ*E* < 0), whereas Path B is exergonic process (Δ*E* > 0), making Path B more plausible, although it is unclear whether alkyl azides can be reduced by Ir(iii)*. Since TEOA proved to be the most effective reductant, we propose that both of its radical cation (TEOA^*Σ*+^) and intermediate iii serve as the proton source and H-atom donor. The α-hydrogen of tertiary amine radical cations (*e.g.*, Et_3_N^*Σ*+^) is relatively acidic (estimated p*K*_a_ = 14.7).^50,51^ This mechanism also rationalizes why TEOA was crucial for obtaining 8 (Table 1): abundant proton sources from TEOA^*Σ*+^ promote the formation of highly reactive ARC species toward alkenes, rather than less reactive aminyl radicals.^52^ It is noted that the reaction of PNAs can proceed *via* aminyl radicals, because the reaction partner was acrylamide X12 as an electrophilic radical acceptor and PNA hybridization forms a pseudo intramolecular systems.

## 3 Conclusion

This study demonstrates that PNA tagging combined with direct MALDI readout offers a simple yet powerful platform for the discovery of new chemical ligations through OPMSS. The rapid data acquisition using MALDI (>10 reactions per h) offers a rapid design-test cycle time. By systematically exploring 64 combinations of PNA pairs in Huisgen cycloaddition and 143 combinations in EDCI-mediated condensation, we identified five and eight ligated products, respectively, highlighting the capability of this approach to discover ligation reactions. Moreover, screening of 72 PNA pairs led to the discovery of a novel ligation initiated by NCR formation from alkyl azides. Although the reactions were divided into 16 subsets to facilitate analysis, this modular screening strategy effectively revealed a visible-light-induced ligation between small-molecule alkyl azides and alkenes. The optimized reaction conditions employ catalytic amounts of [Ir(dtbbpy)ppy_2_]PF_6_ and TEOA. These findings not only expand the utility of OPMSS for reaction discovery but also provide a framework for investigating light-driven radical processes in complex molecular systems.

## Author contributions

Aki Kohyama: conceptualization, data curation, formal analysis, investigation, methodology, writing - original draft. Sofia Barluenga: data curation, investigation, writing - editing. Nicolas Winssinger: conceprualization, formal analysis, writing - review & editing.

## Conflicts of interest

There are no conflicts to declare.

## Supplementary Material

SC-017-D5SC08732E-s001

SC-017-D5SC08732E-s002

SC-017-D5SC08732E-s003

## Data Availability

The experimental procedures and additional data including spectra can be found in the supplementary information (SI). Supplementary information is available. See DOI: https://doi.org/10.1039/d5sc08732e.
