# Supplementary material for: One-pot multi-substrate screening of ligation reactions using PNA tags
Source: Chem Sci. 2026 Feb 20;17(15):7756–65. doi: 10.1039/d5sc08732e (PMC12937129; doi:10.1039/d5sc08732e)

Supplementary Information (SI) for Chemical Science.
This journal is © The Royal Society of Chemistry 2026

Supporting Information

One-pot multi-substrate screening of ligation reactions using PNA tags

Aki Kohyama,* Sofia Barluenga, Nicolas Winssinger *

Department of Organic Chemistry, NCCR Chemical Biology, Faculty of Science, University of Geneva, 30 quai Ernest Ansermet, 1211 Geneva, Switzerland

koyama.aki.6a@kyoto-u.ac.jp; Nicolas.Winssinger@unige.ch

Table of Contents

1. **General Considerations** **S2**
2. **Preparation of PNA libraries** **S3**
3. **Procedure for OPMSS and Data of OPMSS** **S7**
4. **Synthesis of small molecules** **S45**
5. **Experimental data using small molecules** **S49**
6. **References** **S51**
7. **NMR spectra** **S52**

1. General Considerations

LED irradiation at 455 nm were conducted with three types of light as shown below.
Format: Type of lamp (wavelength) : wattage: manufacturer and product number, distance to reaction vessel.

- LED lamp (455 nm): 12W: Thorlabs, M455L2-C1, M00259452, COP-1A), *11 cm*.
- Blue LED (wavelength of peak intensity: 440 nm): 32 W, Kessil, A 160 WE Tuna Blue, *0–2 cm*.
- CFL (compact fluorescent lamp): 20 W, Panasonic EFD25EL20EF2, *2 cm*.

1.1. Experimental procedures

All reagents and solvents for the organic synthesis were purchased from commercial sources and were used without further purification. NovaPEG Rink amide resin for PNA synthesis was obtained from EMD Millipore.
Thin layer chromatography (TLC) was performed on plates of silica precoated with 0.25 mm Kieselgel 60 F254 from Merck. Flash chromatography was performed using silica gel SiliaFlash® P60 (230-400 mesh) from Silicycle or Wakogel® 60N (particle size 63-212 µm) from Wako. Automated solid-phase synthesis was carried out on an Intavis AG Multipep RS instrument. Concentration of the PNA stocks was measured by NanoDrop™ 2000c at 260 nm wavelength.

1.2. Analytical instrumentation

^{1}H-NMR and ^{13}C-NMR spectra were recorded on Bruker Avance AMX-400 or AMX-500 Bruker Avance spectrometers. Chemical shifts are reported in parts per million (ppm) with TMS as an external reference (δ 0.00 ppm) for ^{1}H NMR and referenced to residual solvent signal for ^{13}C NMR ($CDCl_3$ at 77.16 ppm). NMR data are reported as follows: chemical shift, multiplicity (s = singlet, d = doublet, t = triplet, q = quartet, quint = quintet, m = multiplet), coupling constants (Hz), and integration.
High-resolution mass spectra (HRMS) were obtained on a Xevo G2 Tof spectrometer.
LC-MS spectra were recorded on a DIONEX Ultimate 3000 UHPLC (condition for elution gradient: 0 min, A:B =100:0; 4 min, A:B = 10:90; solution A: 0.01% aqueous TFA solution; solution B, 0.01% TFA in HPLC grade acetonitrile; flow rate: 0.750 mL/min) with a Thermo LCQ Fleet Mass Spectrometer System using PINNACLE DB C18 column (1.9 µm, 50 x 2.1 mm) operated in positive mode. All the LC-MS spectra were measured by electrospray ionization (ESI), linear gradient 0 to 100%. MALDI-TOF MS spectra were measured using a Bruker Daltonics Autoflex spectrometer* operated in positive mode. The samples were analyzed using 2,5-dihydroxybenzoic acid (DHB) as matrix. Retention times (RT) are given in minutes.

* MALDI-TOF MS spectra for Table S3-5-2 and Figure 3-2-2 were measured using a Bruker neofleX spectrometer operated in positive mode.

2. Preparation of PNA libraries

2.2.1 Synthesis of PNA-peptide conjugates.

All reagents and solvents for the organic synthesis are purchased from commercial sources and are used without further purification. HPLC purification is performed with an Agilent Technologies 1260 Infinity HPLC using a ZORBAX 300SB-C18 column (5 µm, 9.4 x 250 mm). LC-MS spectra are recorded on a DIONEX Ultimate 3000 UHPLC with a Thermo LCQ Fleet Mass Spectrometer System using a PINNACLE DB C18 column (1.9 µm, 50 x 2.1 mm) operated in positive mode. All LC-MS spectra were acquired by electrospray ionization (ESI) using a linear gradient from 0 to 100%. MALDI-TOF MS spectra are measured using a Bruker Daltonics Autoflex spectrometer operated in positive mode. Automated solid-phase synthesis is carried out on an Intavis AG Multipep RS instrument.

2.2.1.1 General Procedure for synthesis of PNA 6-mers.

5.0 mg of Nova PEG® Rink amide resin (0.44 mmol/g, NovaBiochem, 2.2 µmol) is swollen in CH_2Cl_2 for 10 minutes and washed two times with DMF. Iterative cycles of amide coupling (**Procedure 2**), capping of the resin (**Procedure 5**) and deprotection of the main chain protecting group (**Procedure 3** or **4**) are done to synthesize the PNA-peptide sequences. The compounds are deprotected and cleaved from the resin using **Procedure 6** and finally purified using HPLC when needed.

2.2.1.3 Procedure 2: Amide coupling.

The corresponding Fmoc protected PNA monomer or amino acid (4.0 equiv, 0.2 M in NMP) is mixed with a pipette for 30 seconds with HATU (3.5 equiv, 0.25 M in NMP) and base solution [DIPEA, 1.2 M (4.0 equiv) and 2,6-lutidine 1.8 M (6.0 equiv) in NMP]. The mixture is then added to the corresponding resin. After 30 minutes, the mixture is filtered, the resin is washed with DMF, and a new premixed reaction solution is added to the resin and allowed to react for another 30 minutes. Finally, the resin is washed with 2x DMF, 2x CH_2Cl_2, and 2x DMF.

2.2.1.4 Procedure 3: Fmoc deprotection.

A solution of 20% piperidine in DMF is added to the resin and allowed to react for 5 minutes. The mixture is then filtered, the resin is washed with DMF, and the sequence repeated a second time for another 5 minutes. Finally, the resin is washed with 2x DMF, 2x CH_2Cl_2, and 2x DMF.

2.2.1.5 Procedure 4: Mtt deprotection.

A solution (made from 244 mg of HOBt in 10 mL of HFIP and 10 mL of DCE) is added to the prewashed resin to reach a volume of 10 mL/g of resin and allowed to react for 5 minutes. The solution is flushed, the resin is washed with CH_2Cl_2, and the sequence is repeated a second time for another 5 minutes. Finally, the resin is washed with 2x CH_2Cl_2 and 2x DMF.

2.2.1.6 Procedure 5: Capping.

The resin is treated with a capping mixture (0.92 mL of acetic anhydride and 1.3 mL of 2,6 lutidine in 18 mL of DMF: 10 mL of solution/g of resin) for 5 minutes. After flushing the solution, the resin is washed with 2x DMF, 2x CH_2Cl_2 and 2x DMF.

2.2.1.7 Procedure 6: Cleavage from the resin and final deprotection.

The resin (2.2 µmol) is treated with 125 µL of a mixture of TFA and scavengers (440 µL of TFA + 25 mg phenol + 25 µL water + 10 µL triisopropylsilane) for 2 hours. The resin is filtered, washed with TFA (80 µL) and the collected fractions of cleavage product are precipitated in cold ether (1.5 mL). After centrifugation (13 K rpm, 5 minutes), the supernatant is removed and the pellet is vortexed again with cold Et_2O (1.5 mL) and centrifuged (13 K rpm, 5 minutes). The resulting pellet is dissolved in H_2O/CH_3CN (1/1, 1.5 mL) and lyophilized or evaporated with nitrogen to obtain a white powder. The white powder is dissolved in H_2O and the sample concentration is measured using NanoDrop.

2.2.1.8 Procedure 7: Microcleavage for quality control.

The minimum number of beads is picked up with a pipette plastic tip and transferred to 50 µL of TFA. After the solution has been left for 30 minutes, TFA is removed with a centrifugal evaporator. The resulting pellet is dissolved in 20 µL of 1:1 acetonitrile/water, which is then analyzed by MALDI-TOF MS and/or LC-MS.

2.2.1.9 Procedure 8: Amidation with cyclic anhydrides. (for X13—X16)

After Mtt deprotection of Dap-NHMtt at the C-terminal, the resin (2.2 μmol) is treated with succinic anhydride or diglycolic anhydride (15 equiv, 0.27 M in NMP) and DIPEA (11 μL, 65 μmol, 30 equiv). After 30 minutes, the mixture is filtered, the resin is washed with DMF, and a new reaction solution is added to the resin and allowed to react for another 30 minutes. Finally, the resin is washed with 2x DMF, 2x CH_2Cl_2, and 2x DMF.

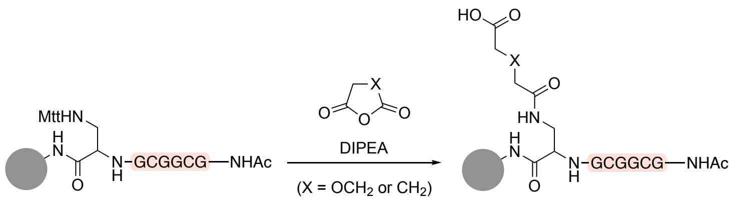

2.2.1.10 Procedure 9: SN2 reaction of primary amines with α-chloro amide in PNA. (for X1, X5, X6, X8, Y13)

The resin (1.1 or 2.2 μmol) is treated with a mixture of amines [primary amine, 0.25 M (25 equiv or 50 equiv) and DIPEA 0.10 M (10 or 20 equiv) in 1,4-dioxane]. After 5 hours at 50 °C (for **Y13**, 6.5 hours at 80 °C), the mixture is filtered, and the resin is washed with 2x DMF, 2x CH_2Cl_2, and 2x DMF. **X5**, **X6**, and **Y13** are kept at this stage.

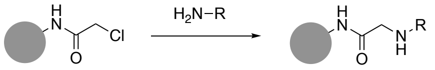

For **X1**, a new reaction solution is added to the resin and allowed to react for another 11 hours at 50 °C. Finally, the mixture is filtered and the resin is washed with 2x DMF, 2x CH_2Cl_2, and 2x DMF.

For **X8**, a new reaction solution is added to the resin and allowed to react for another 11 hours at 50 °C. After the mixture is filtered and the resin is washed with 2x DMF, 2x CH_2Cl_2, and 2x DMF, a new reaction solution is added to the resin and allowed to react for another 7 hours at 70 °C. Finally, the mixture is filtered and the resin is washed with 2x DMF, 2x CH_2Cl_2, and 2x DMF.

2.2.1.11 Procedure 10: Condensation of primary amines with carboxylic acid in PNA. (for X7, X10)

The resin (1.7 μmol) is treated with primary amines (10 equiv, 0.25 M in NMP) and HATU (8.9 equiv, 0.25 M in NMP) and base solution [DIPEA, 1.2 M (10 equiv) and 2,6-lutidine 1.8 M (15 equiv) in NMP]. After 2 hours, the mixture is filtered, the resin is washed with DMF, and a new reaction solution is added to the resin and allowed to react for another 15 hours. Finally, the resin is washed with 2x DMF and 2x CH_2Cl_2.

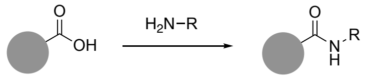

2.3. Characterization of PNA.

Characterization of the PNAs was done by MALDI-TOF MS (Bruker Daltonics Autoflex spectrometer with FlexControl 3.4 software and analysis with FlexAnalysis 3.4) and/or LC-MS (DIONEX Ultimate 3000 UHPLC with a Thermo LCQ Fleet Mass Spectrometer System using PINNACLE DB C18 column (1.9 μm, 50 x 2.1 mm) with Thermo Xcalibur 2.2.SP1.48 software and analysis with Thermo Xcalibur Qual Browser 2.2.SP1.48). For MALDI analysis, 1.0 μL of the sample (in either water or water/acetonitrile 1:1) was mixed with 1.0 μL of DHB matrix solution (30 mg of DHB in 1.0 mL of 70:30:0.01 water/acetonitrile/TFA), and the mixture was spotted on a MALDI plate. The measurements were done in a positive linear mode*. Calibration Standard Peptide II (Bruker LabScape – Daltonics 8222570) was used for instrument calibration prior to measurement. For LC-MS analysis, 20 μL of sample in water or water/acetonitrile 1:1 were injected into the LC-MS system.

*

Table S2-1. List of PNA X (12 functional groups)

No.	Structure	Huisgen	Amidation	PC
**X1**	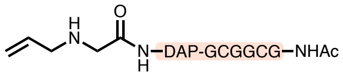	–	✓	✓
**X2**	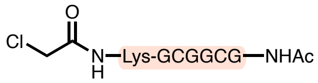	–	–	✓
**X3**	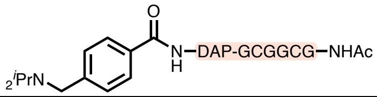	–	✓	✓
**X4**	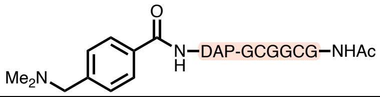	–	✓	✓
**X5**	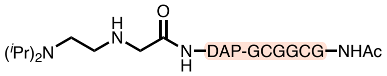	–	✓	✓
**X6**	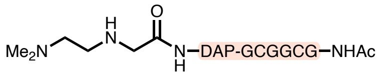	–	✓	✓
**X7**	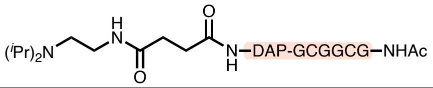	✓	–	✓
**X8**	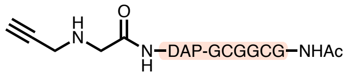	–	✓	✓
**X9**	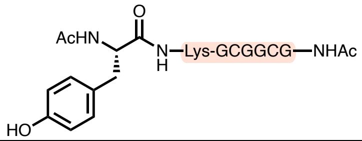	✓	–	✓
**X10**	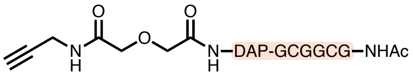	✓<br>positive	–	✓
**X11**	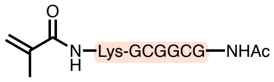	✓	✓	✓
**X12**	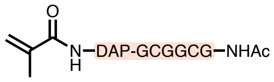	–	✓	✓
**X13**	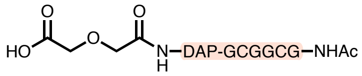	✓	✓<br>positive	✓
**X14**	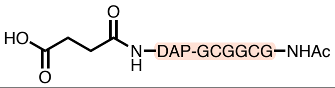	✓	✓<br>positive	✓
**X15**	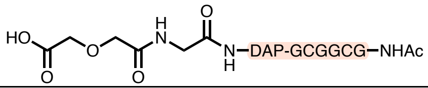	✓	✓<br>positive	✓
**X16**	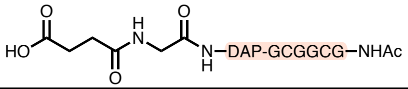	✓	✓<br>positive	✓
**X17**	H_2N–Lys-GCGGCG–NHAc	–	✓	–

Table S2-2. List of PNA Y (13 functional groups)

No.	Structure	Huisgen	Amidation	photo
**Y1**	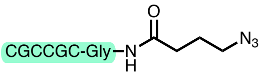	✓ positive	–	✓
**Y2**	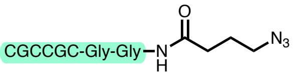	–	✓	✓
**Y3**	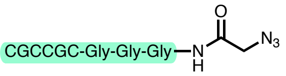	✓ positive	–	✓
**Y4**	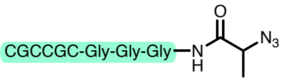	✓ positive	–	✓
**Y5**	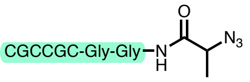	–	✓	✓
**Y6**	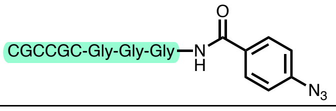	–	✓	✓
**Y7**	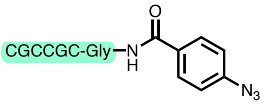	✓ positive	✓	✓
**Y8**	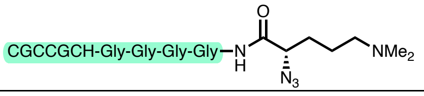	✓ positive	✓	✓
**Y9**	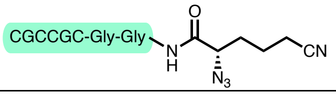	–	–	✓
**Y10**	CGCCGC-Gly-Gly-Gly-Gly—NH_2	–	✓	–
**Y11**	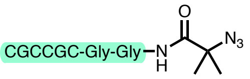	–	✓	✓
**Y12**	CGCCGC-Gly-Gly-Gly-NH_2	–	✓ positive	–
**Y13**	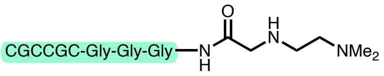	–	✓	–
**Y14**	CGCCGC-Gly-Gly-Gly-Glu—NHAc	✓	–	–
**Y15**	CGCCGC-Gly-Gly-Gly-Trp—NHAc	✓	–	–
**Y16**	CGCCGC-Gly-Gly-Gly-Tyr—NHAc	✓	–	–
**Y17**	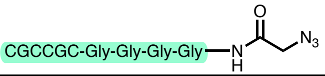	–	✓	–
**Y18**	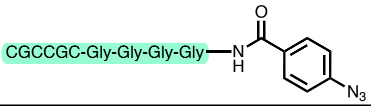	–	✓	–
**Y19**	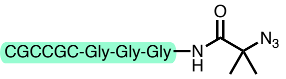	–	–	✓

3. Procedure for OPMSS and Data of OPMSS

3.1. General information

A 1.5 mL Eppendorf tube or 0.2 mL PCR tube (corning, PCR-02-C) was used for each reaction. Each reaction mixture was monitored and analyzed by following **GT1**.

3.2. General treatment before MALDI-TOF MS (GT1)

The reaction mixture (total 10-100 mM, PNA) was diluted tenfold with MeCN-H_2O (1 : 1). After DHB matrix solution was spotted on a MALDI plate, the resulting PNA solution (0.5 mL) was spotted on the plate and mixed with DHB matrix solution by pipetting. After drying*, the resulting solid was analyzed by using linear positive mode or reflector positive mode of MALDI-TOF MS.

*After drying, DHB matrix solution was spotted and dried it again, if the reaction mixture contained DMF or DMSO.

3.3.1. Click reaction (Fig.3)

The click reaction cocktail was prepared as shown in Table S3-2. PNA pool Xclick and PNA pool Yclick were dissolved in the cocktail as shown in Table S3-1. After being incubated at room temperature for 2 h, the reaction mixture was analyzed by following **GT1**.

Table S3-1. Reaction conditions for click reaction: 8 x 8

Name	Volume / μL	Final concentration	note
100 μM PNA pool 635_X aq. *1	1.0	5.0 μM x 8 (total 40 μM)	—
50 μM PNA pool 635_Y aq. *2	2.0	5.0 μM x 8 (total 40 μM)	—
click reaction cocktail*3	17	$CuSO_4$; 0.43 mM	$CuSO_4$; 86 equiv/PNA-alkyne
Total	20	—	—

*1PNA pool Xclick (eight PNA X): **X7**, **X9**, **X10**, **X11**, **X13**, **X14**, **X15**, and **X16**
*2PNA pool Yclick (eight PNA Y): **Y1**, **Y3**, **Y4**, **Y7**, **Y8**, **Y16**, **Y17**, and **Y18**
*3For a negative control experiment, 1 x DPBS buffer (17 μL) was used instead of the click reaction cocktail.

Table S3-2. Click reaction cocktail[1]

Reagents	Final concentration in 1 x DPBSbuffer
$CuSO_4$	0.5 mM
THPTA	5 mM
Na ascorbate	2.5 mM
1 x DPBS buffer	—

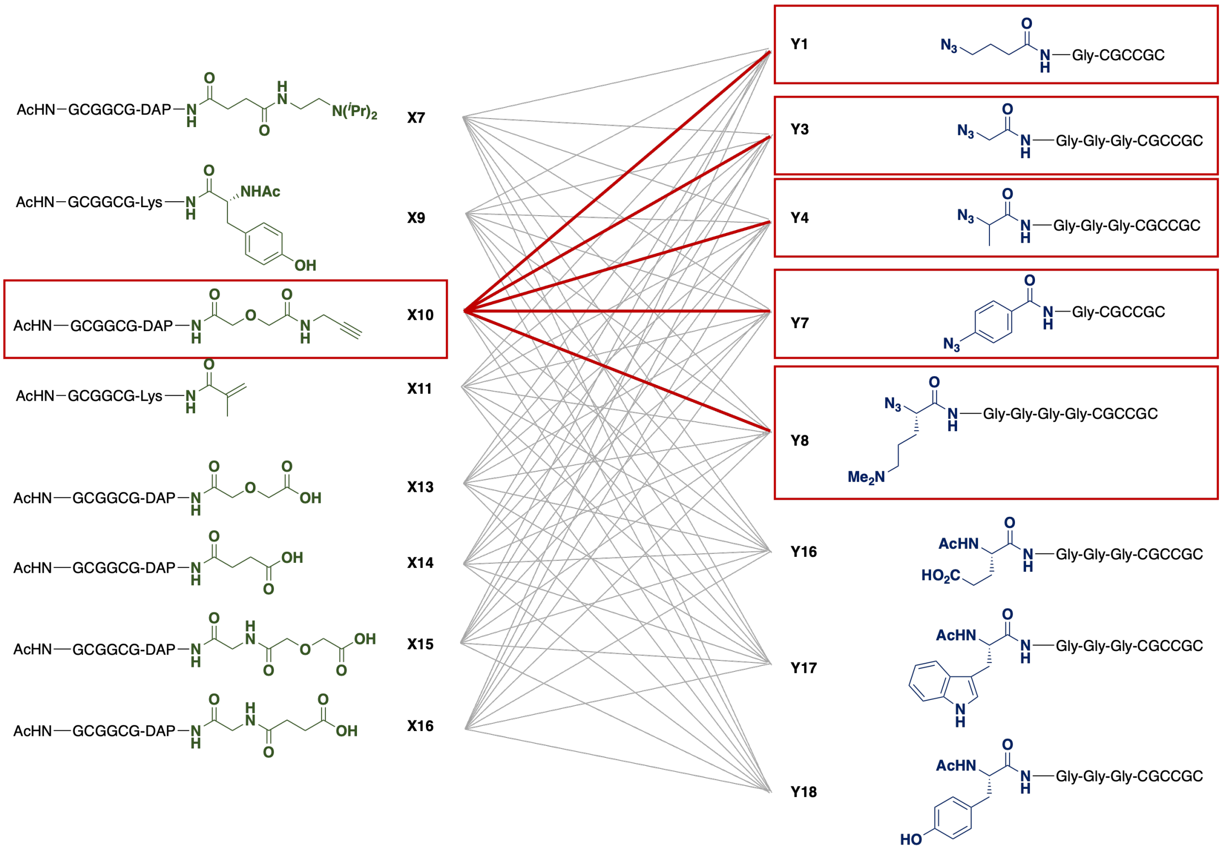

Figure S3-1. Substrate hybridization combinations and ligation outcomes.
Gray lines represent possible hybridization combinations, and red lines represent ligated products detected after the reaction.

Table S3-3. List of observed peaks and assigned PNA sequences_ Huisgen cycloaddition_2 h

found. m/z	assigned PNA	assigned PNA sequence (N to C)	Molecular formula	calc. m/z average mass [M+H]$^{+}$	error
3736.840	X10 + Y1	GCGGCG-DAP-**linker2-T1**-Gly-CGCCGC	C144H185N81O43	3739.535	-2.70
3771.292	X10 + Y7	GCGGCG-DAP-**linker2-T4**-Gly-CGCCGC	C147H183N81O43	3773.551	-2.26
3822.327	X10 + Y3	GCGGCG-DAP-**linker2-T2**-Gly-Gly-Gly-CGCCGC	C146H187N83O45	3825.583	-3.26
3837.561	X10 + Y4	GCGGCG-DAP-**linker2-T3**-Gly-Gly-Gly-CGCCGC	C147H189N83O45	3839.610	-2.05
3965.061	X10 + Y8	GCGGCG-DAP-**linker2-T5**-Gly-Gly-Gly-Gly-CGCCGC	C153H201N85O46	3967.783	-2.72

Linear positive ion mode was utilized for Table S3-3.

*Average mass was calculated with enviPat Web(https://www.envipat.eawag.ch/index.php).

Observed mass deviations (±0–4.3 Da) may be attributed to instrument calibration drift.

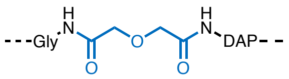

linker 1

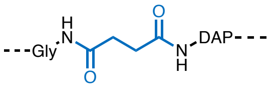

linker 2

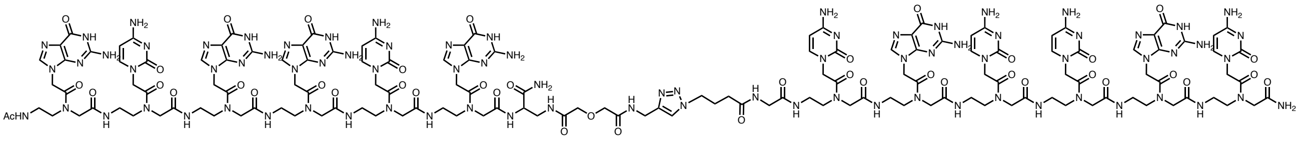

X10 + Y1: GCGGCG-DAP-**linker2-T1**-Gly-CGCCGC
Chemical Formula: $C_{144}H_{185}N_{81}O_{43}$

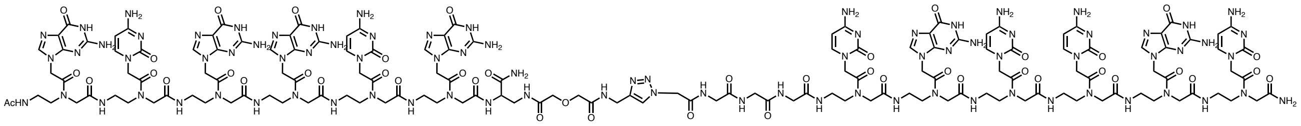

X10 + Y3: GCGGCG-DAP-**linker2-T2**-Gly-Gly-Gly-CGCCGC
Chemical Formula: $C_{146}H_{187}N_{83}O_{45}$

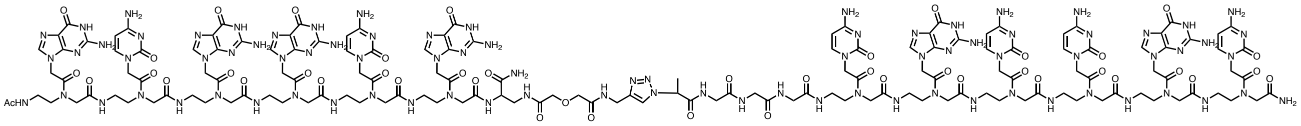

X10 + Y4: GCGGCG-DAP-**linker2-T3**-Gly-Gly-Gly-CGCCGC
Chemical Formula: $C_{147}H_{189}N_{83}O_{45}$

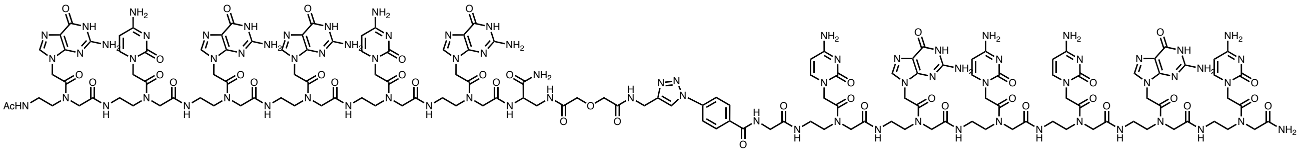

X10 + Y7: GCGGCG-DAP-**linker2-T4**-Gly-CGCCGC
Chemical Formula: $C_{147}H_{183}N_{81}O_{43}$

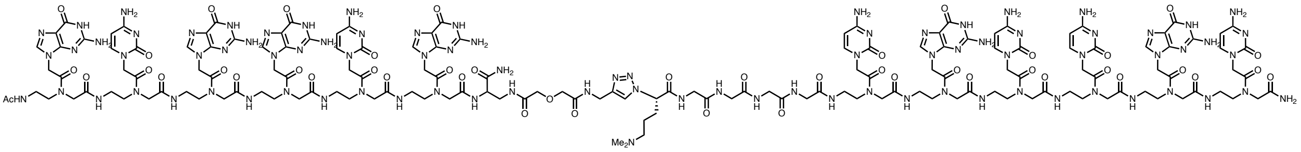

X10 + Y8: GCGGCG-DAP-**linker2-T5**-Gly-Gly-Gly-Gly-CGCCGC
Chemical Formula: $C_{153}H_{201}N_{85}O_{46}$

3.3.2. Condensations (Fig. 4)

PNA pool Xamide and PNA pool Yamide were dissolved in a reaction mixture containing EDCI in DMF : MES buffer as shown in Table S3-4. After being incubated at room temperature for 11 h, the reaction mixture was analyzed.

Table S3-4. Reaction conditions for condensation

Name	Volume / μL	Final concentration	Note
33 μM PNA pool 447_X aq.*1	3.0	5.0 μM x 13	—
33 μM PNA pool 447_Y aq.*2	3.0	5.0 μM x 11	—
0.50 M EDCI in DMF-H_2O (1 : 1)*3	5.0	0.20 M	1.3 x 10^4 equiv / PNA-CO_2H
H_2O	1.0	—	—
DMF	4.0	—	—
5 x MES buffer	4.0	—	—
Total	20	—	—

*1PNA pool Xamide (13 PNA X): **X1**, **X3**, **X4**, **X5**, **X6**, **X8**, **X11**, **X12**, **X13**, **X14**, **X15**, **X16**, and **X17**
*2PNA pool Yamide (11 PNA Y): **Y2**, **Y5**, **Y6**, **Y7**, **Y8**, **Y10**, **Y11**, **Y12**, **Y13**, **Y17**, and **Y18**
*3For a negative control experiment, DMF-H_2O (1 : 1, 5 mL) was used instead of 0.5 M EDCI.

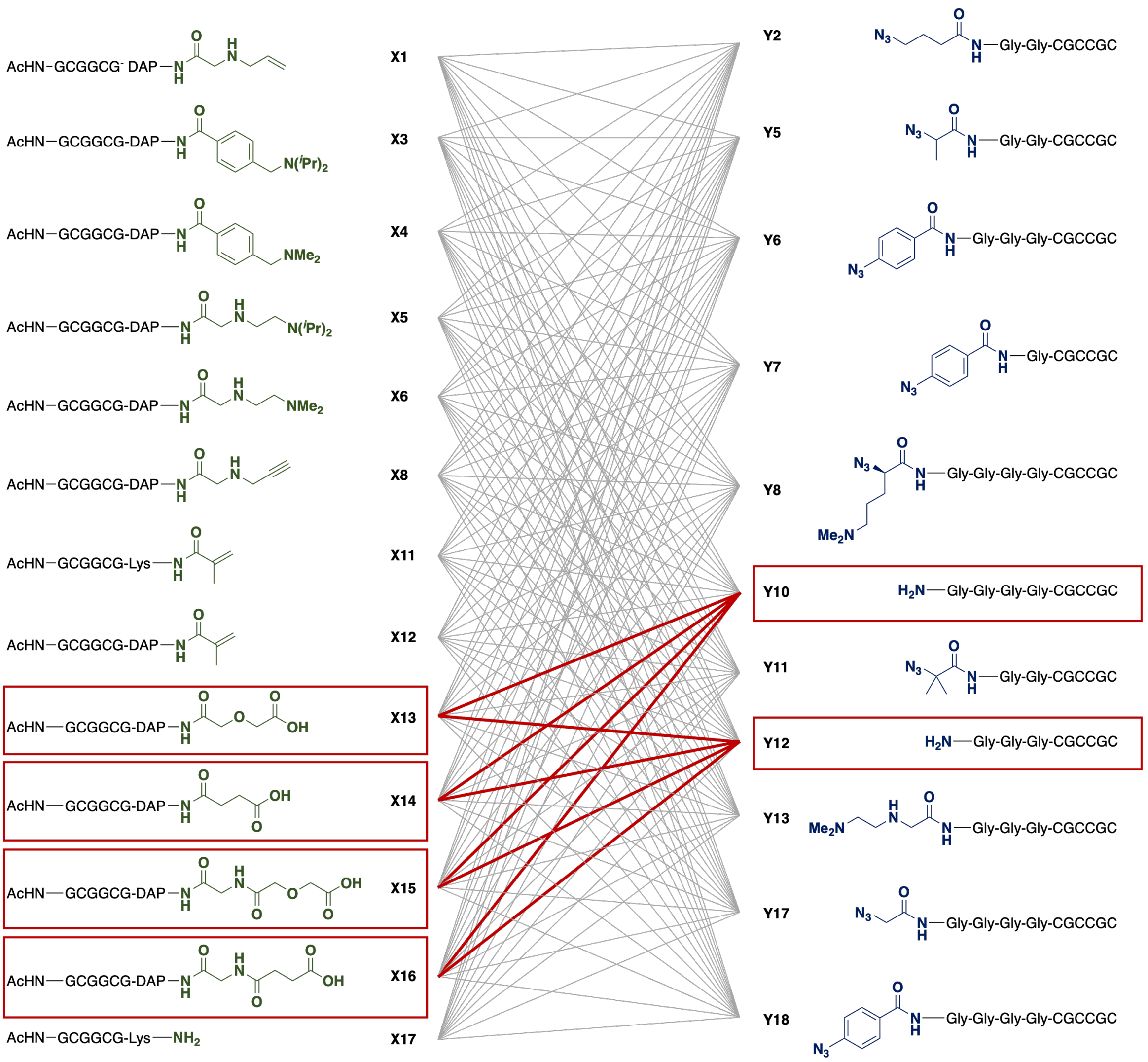

Figure S3-2-1. Substrate combinations and condensation outcomes.
Gray lines represent possible hybridization combinations, and red lines represent condensed products detected after the reaction.

Table S3-5-1. List of observed peaks and assigned PNA sequences after EDCI coupling, 11 h.

found. m/z	assigned PNA	assigned PNA sequence (N to C)	Molecular formula	calc. m/z average mass [M+H]$^+$	error
3674.884	X14 + Y12	GCGGCG-DAP-**linker2**-Gly-Gly-Gly-CGCCGC	C141H181N79O43	3671.457	3.43
3690.895	X13 + Y12	GCGGCG-DAP-**linker1**-Gly-Gly-Gly-CGCCGC	C141H181N79O44	3687.457	3.44
3731.869	X16 + Y12	GCGGCG-DAP-Gly-**linker2**-Gly-Gly-Gly-CGCCGC	C143H184N80O44	3728.508	3.36
	X14 + Y10	GCGGCG-DAP-**linker2**-Gly-Gly-Gly-Gly-CGCCGC			
3747.939	X15 + Y12	GCGGCG-DAP-Gly-**linker1**-Gly-Gly-Gly-CGCCGC	C143H184N80O45	3744.508	3.43
	X13 + Y10	GCGGCG-DAP-**linker1**-Gly-Gly-Gly-Gly-CGCCGC			
3789.504	X16 + Y10	GCGGCG-DAP-Gly-**linker2**-Gly-Gly-Gly-Gly-CGCCGC	C145H187N81O45	3785.560	3.94
3805.784	X15 + Y10	GCGGCG-DAP-Gly-linker1-Gly-Gly-Gly-Gly-CGCCGC	C145H187N81O46	3801.559	4.22

Linear positive ion mode was utilized for Table S3-5-1.

Observed mass deviations (±0–4.3 Da) may be attributed to instrument calibration drift.

Table S3-5-2. List of observed peaks and assigned PNA sequences after EDCI coupling, 11 h.

found. m/z	assigned PNA	assigned PNA sequence (N to C)	Molecular formula	calc. m/z peak in isotopic distribution [M+H]$^+$	error	colour
3671.034	X14 + Y12	GCGGCG-DAP-**linker2**-Gly-Gly-Gly-CGCCGC	C141H181N79O43	3670.450	0.58	
3687.121	X13 + Y12	GCGGCG-DAP-**linker1**-Gly-Gly-Gly-CGCCGC	C141H181N79O44	3686.447	0.67	
3728.057	X16 + Y12	GCGGCG-DAP-Gly-**linker2**-Gly-Gly-Gly-CGCCGC	C143H184N80O44	3727.473	0.58	
	X14 + Y10	GCGGCG-DAP-**linker2**-Gly-Gly-Gly-Gly-CGCCGC				
3744.132	X15 + Y12	GCGGCG-DAP-Gly-**linker1**-Gly-Gly-Gly-CGCCGC	C143H184N80O45	3743.465	0.67	
	X13 + Y10	GCGGCG-DAP-**linker1**-Gly-Gly-Gly-Gly-CGCCGC				
3785.132	X16 + Y10	GCGGCG-DAP-Gly-**linker2**-Gly-Gly-Gly-Gly-CGCCGC	C145H187N81O45	3784.495	0.64	
3801.193	X15 + Y10	GCGGCG-DAP-Gly-linker1-Gly-Gly-Gly-Gly-CGCCGC	C145H187N81O46	3800.487	0.71	

Reflector positive ion mode was utilized for Table S3-5-2.

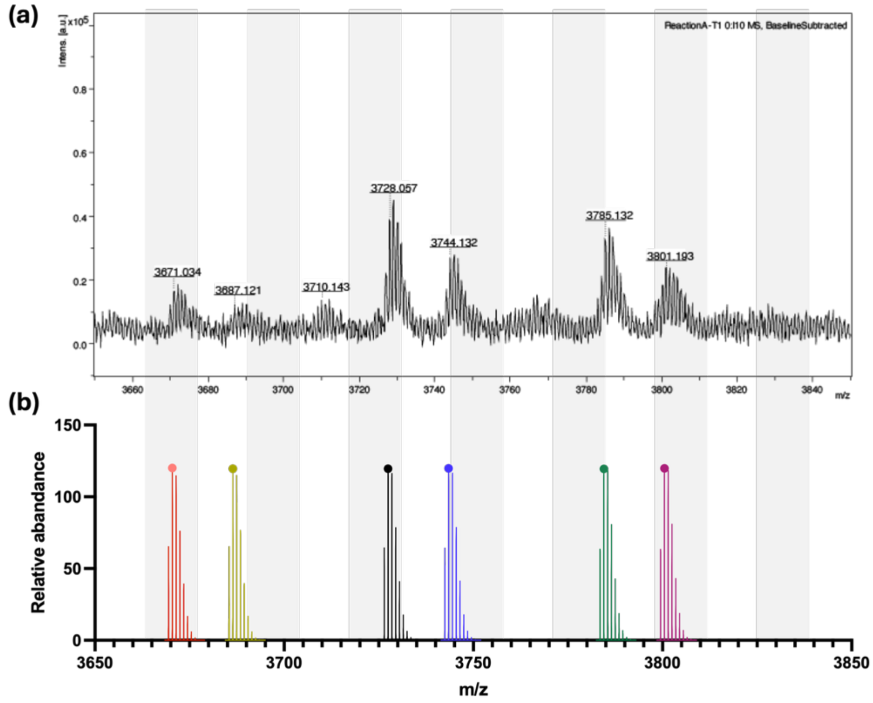

Figure S3-2-2. MALDI-TOF MS spectra of the reaction mixture (Reflector positive mode). (a) Observed spectra, (b) Calculated spectra.

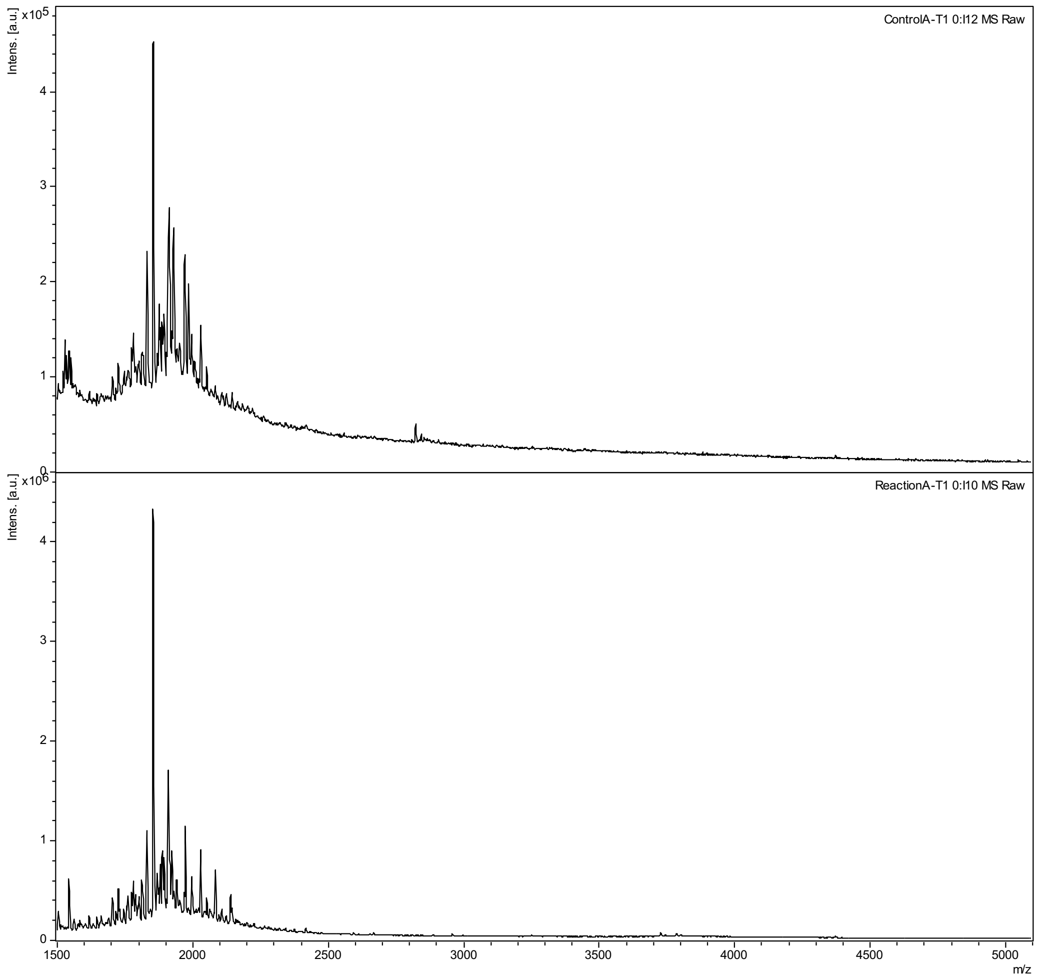

Figure S3-2-3. Observed spectra of reaction mixtures (Reflector positive mode). Upper: without EDCI, Lower: with EDCI.

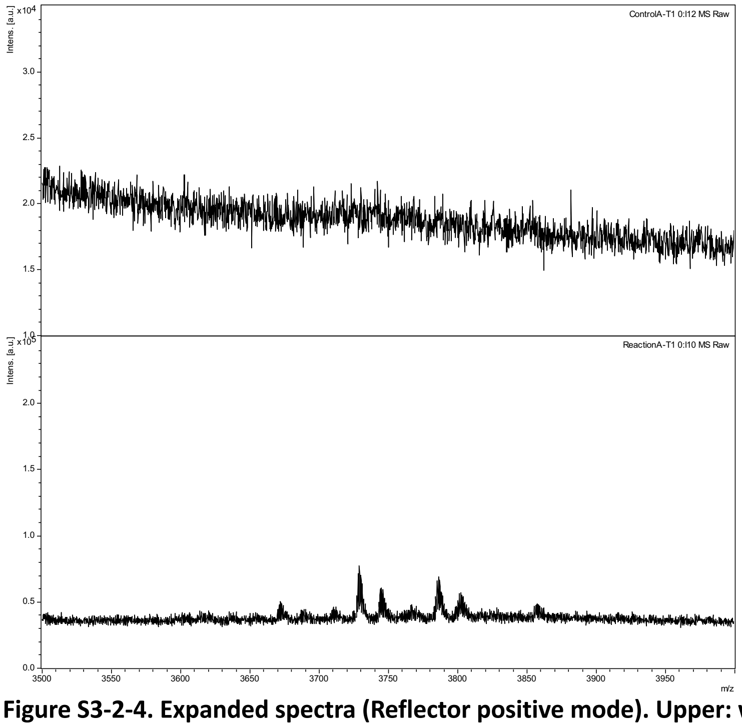

Figure S3-2-4. Expanded spectra (Reflector positive mode). Upper: without EDCI, Lower: with EDCI

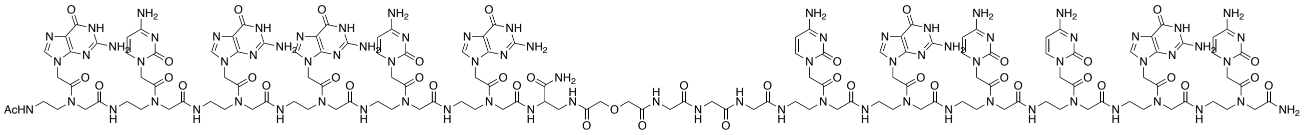

X13 + Y12: GCGGCG-DAP-**linker1**-Gly-Gly-Gly-CGCCGC
Chemical Formula: $C_{141}H_{181}N_{79}O_{44}$

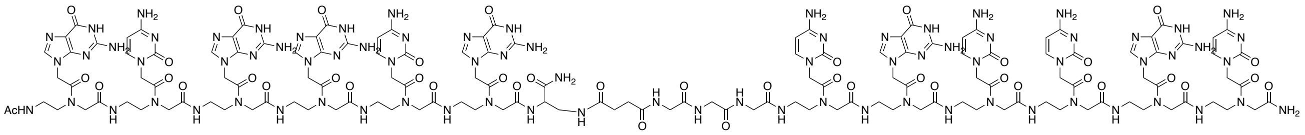

X14 + Y12: GCGGCG-DAP-**linker2**-Gly-Gly-Gly-CGCCGC
Chemical Formula: $C_{141}H_{181}N_{79}O_{43}$

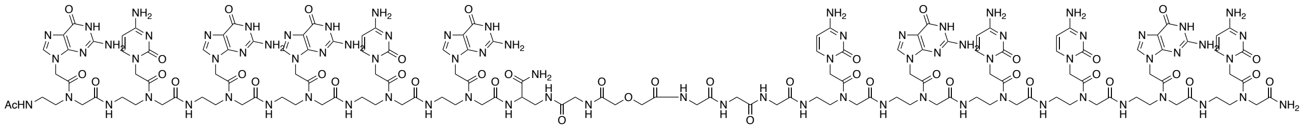

X15 + Y12: GCGGCG-DAP-Gly-**linker1**-Gly-Gly-Gly-CGCCGC
Chemical Formula: $C_{143}H_{184}N_{80}O_{45}$

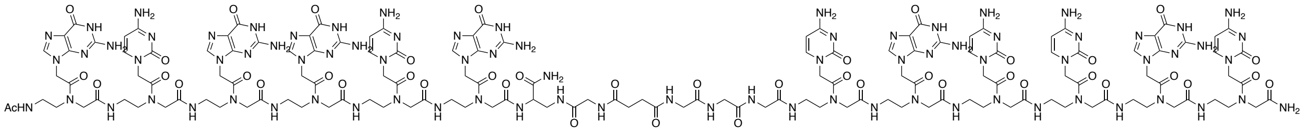

X16 + Y12: GCGGCG-DAP-Gly-**linker2**-Gly-Gly-Gly-CGCCGC
Chemical Formula: $C_{143}H_{184}N_{80}O_{44}$

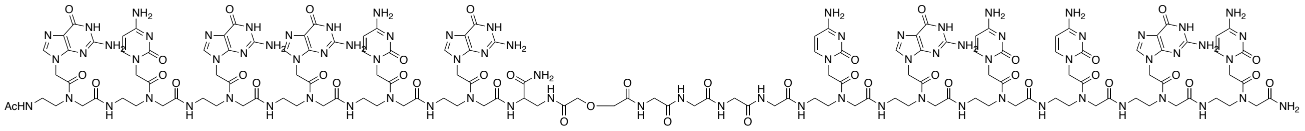

X13 + Y10: GCGGCG-DAP-**linker1**-Gly-Gly-Gly-Gly-CGCCGC
Chemical Formula: $C_{143}H_{184}N_{80}O_{45}$

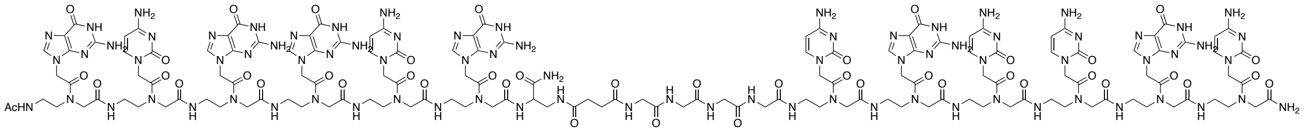

X14 + Y10: GCGGCG-DAP-**linker2**-Gly-Gly-Gly-Gly-CGCCGC
Chemical Formula: $C_{143}H_{184}N_{80}O_{44}$

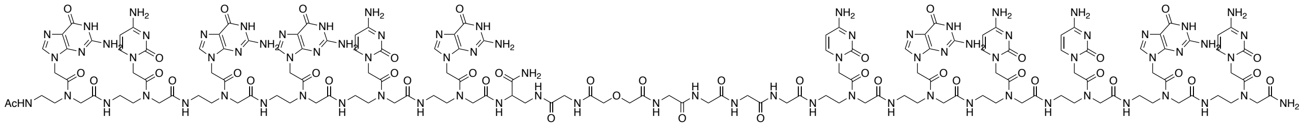

X15 + Y10: GCGGCG-DAP-Gly-**linker1**-Gly-Gly-Gly-Gly-CGCCGC
Chemical Formula: $C_{145}H_{187}N_{81}O_{46}$

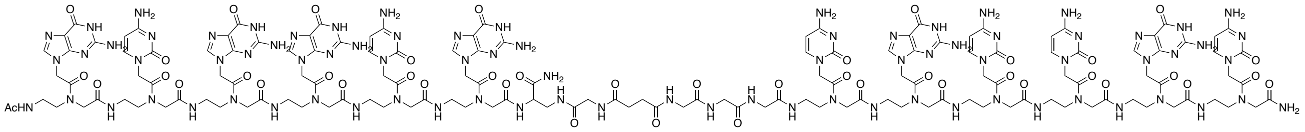

X16 + Y10: GCGGCG-DAP-Gly-**linker2**-Gly-Gly-Gly-CGCCGC
Chemical Formula: $C_{143}H_{184}N_{80}O_{44}$

3.3.3. Photoredox reactions

Before bond forming by photoredox reaction, we investigated the reaction conditions to activate PNAs containing azides via denitrogenation (see 3.3.3.1.)

3.3.3.1. Preliminary experiments

Table S3-6_Preliminary experiment for photoredox reactions

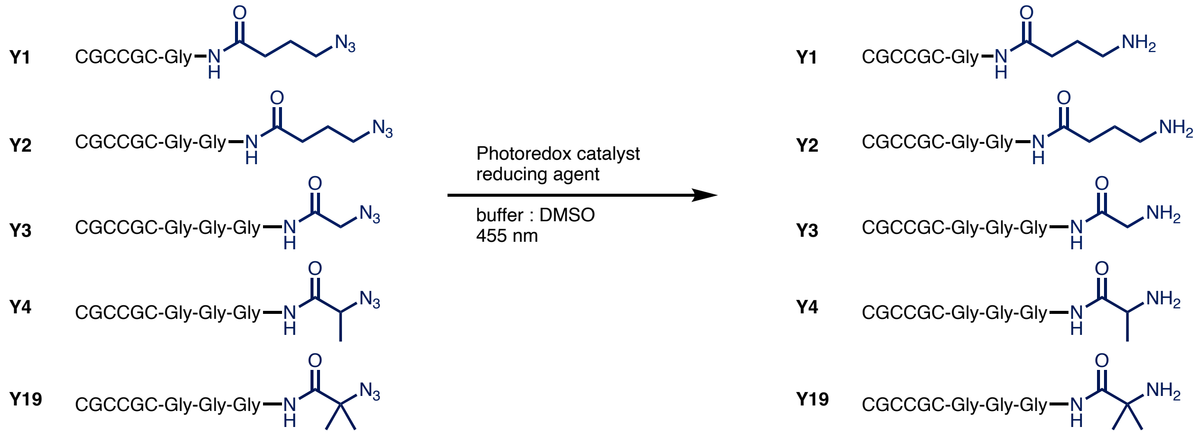

(a)

Name	Volume / μL	Final concentration	Note
100 μM*1 PNA pool aq.(three PNA)	1.5	7.5 μM x 8	—
20 mM Ru(bpy)$_3$Cl$_2$ aq.	1.0	1 mM	17 equiv. / PNA-N_3
reducing agent	< 1.0	50 mM	20 equiv. / PNA-N_3
1.0 M Tris buffer	4.0	200 mM	—
H_2O	12.5	—	—
Total	20	—	—

Name: photoredox catalyst, reducing agent
Condition 1: Ru(bpy)$_3$Cl$_2$, Na ascorbate
Condition 2: Ru(bpy)$_3$Cl$_2$, DIPEA
Condition 3: Ru(bpy)$_3$Cl$_2$, TMEDA

(b)

Name	Volume / μL	Final concentration	Note
100 μM*1 PNA pool aq.(three PNA)	1.5	7.5 μM x 8	—
20 mM Ru(bpy)$_3$Cl$_2$ catalyst in H_2O	1.0	1 mM	17 equiv. / PNA-N_3
60 mM Na_2CO_3 aq.	1.0	3 mM	50 equiv. / PNA-N_3
H_2O	16.5	—	—
Total	20	—	—

Condition 4: Ru(bpy)$_3$Cl$_2$, none, (Na_2CO_3 was used instead of Tris buffer)

Table S3-7_Preliminary experiment for photoredox reaction

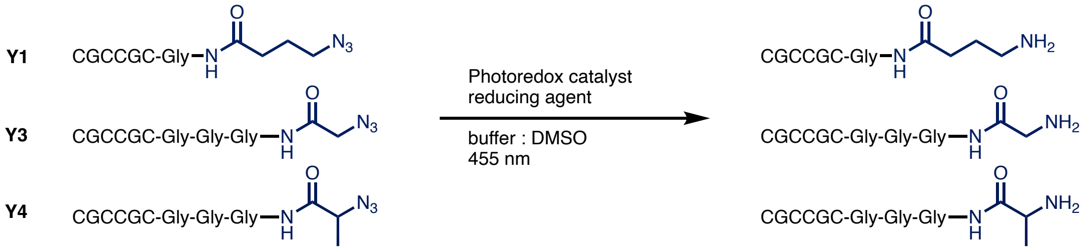

(a)

Name	Volume / μL	Final concentration	Note
100 μM*1 PNA pool aq.(three PNA)	1.5	7.5 μM x 3	—
20 mM photoredox catalyst	1.0	1 mM	44 equiv. / PNA-N_3
reducing agent	< 1.0	50 mM	2.2 x 10^3 equiv. / PNA-N_3
1.0 M Tris buffer	4.0	200 mM	—
H_2O	8.5	—	—
DMSO	4.0	—	—
Total	20	—	—

Used photoredox catalysts: [Ir(dtbbpy)(ppy)$_2$]PF$_6$ or Ir[dF(CF$_3$)ppy]$_2$(dtbpy)PF$_6$ in DMSO, Ru(bpy)$_3$Cl$_2$ in H_2O

Used reducing agents (volume): 1.0 M Na ascorbate aq. (1.0 μL), 1.0 M TEOA aq. (1.0 μL), TMEDA (0.2 μL), non

*[1]a concentration of each PNA, PNA pool (**Y1**, **Y3**, and **Y4**)

Condition 5: [Ir(dtbbpy)(ppy)$_2$]PF$_6$, Na ascorbate
Condition 6: [Ir(dtbbpy)(ppy)$_2$]PF$_6$, TEOA
Condition 7: [Ir(dtbbpy)(ppy)$_2$]PF$_6$, non
Condition 8: [Ir(dtbbpy)(ppy)$_2$]PF$_6$, TMEDA
Condition 9: Ir[dF(CF$_3$)ppy]$_2$(dtbpy)PF$_6$, Na ascorbate
Condition 10: Ir[dF(CF$_3$)ppy]$_2$(dtbpy)PF$_6$, TEOA
Condition 11: Ir[dF(CF$_3$)ppy]$_2$(dtbpy)PF$_6$, non
Condition 12: Ir[dF(CF$_3$)ppy]$_2$(dtbpy)PF$_6$, TMEDA
Condition 13: Ru(bpy)$_3$Cl$_2$, TEOA
Condition 14: Ru(bpy)$_3$Cl$_2$, none

(b)

Name	Volume / μL	Final concentration	Note
100 μM*1 PNA pool aq.(three PNA)	1.5	7.5 μM x 3	—
20 mM photoredox catalyst	1.0	1 mM	44 equiv. / PNA-N_3
reducing agent	< 1.0	50 mM	2.2 x 10^3 equiv. / PNA-N_3
1.0 M Tris buffer	4.0	200 mM	—
H_2O	3.5	—	—
DMSO	9.0	—	—
Total	20	—	—

Condition 15: [Ir(dtbbpy)(ppy)$_2$]PF$_6$, Na ascorbate
Condition 16: [Ir(dtbbpy)(ppy)$_2$]PF$_6$, TEOA
Condition 17: [Ir(dtbbpy)(ppy)$_2$]PF$_6$, non
Condition 18: [Ir(dtbbpy)(ppy)$_2$]PF$_6$, TMEDA
Condition 19: Ir[dF(CF$_3$)ppy]$_2$(dtbpy)PF$_6$, Na ascorbate
Condition 20: Ir[dF(CF$_3$)ppy]$_2$(dtbpy)PF$_6$, TEOA
Condition 21: Ir[dF(CF$_3$)ppy]$_2$(dtbpy)PF$_6$, non
Condition 22: Ir[dF(CF$_3$)ppy]$_2$(dtbpy)PF$_6$, TMEDA
Condition 23: Ru(bpy)$_3$Cl$_2$, TEOA
Condition 24: Ru(bpy)$_3$Cl$_2$, none

Procedure for Table S3-6, S3-7, and S3-10

All reagents were mixed. Then, 18 μL of the reaction mixture was irradiated at 455 nm with a collimated LED light 6 cm above the plate. On the other hand, 2 μL of the reaction mixture was incubated in the dark, which was used as a negative control. After being irradiated for 10 min, 30 min, and 3 h, the reaction mixture was analyzed at each time point.

Note: DIPEA was not suitable due to low water solubility.

Results

Among 18 conditions, two reaction conditions giving better result are shown below. Although condition 20 also resulted in high conversion of azides, the solubility of *Ir[dF(CF_3)ppy]$_2$(dtbpy)PF_6 was lower than that of [Ir(dtbbpy)(ppy)$_2$]PF_6. Therefore, condition 16 and 18 were selected as optimized conditions.*

-Condition 16: [Ir(dtbbpy)(ppy)$_2$]PF_6, TEOA

-Condition 18: [Ir(dtbbpy)(ppy)$_2$]PF_6, TMEDA

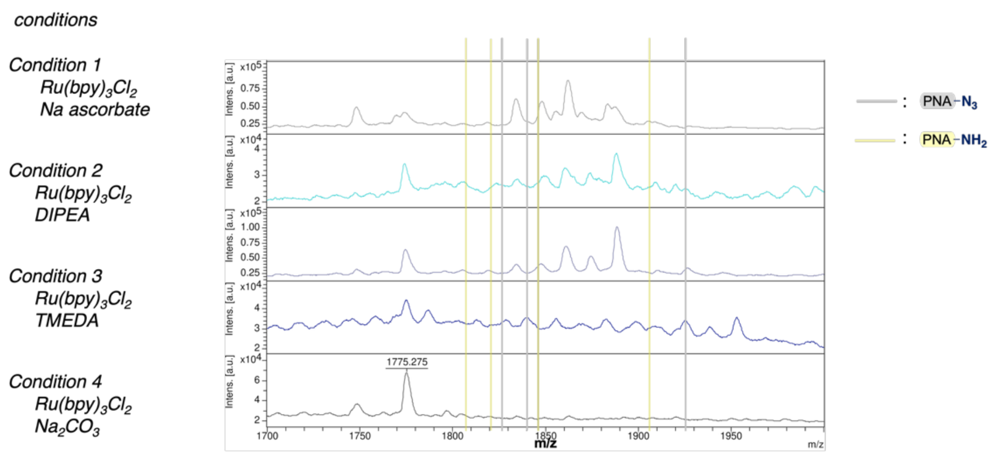

Figure S3-3-1. MALDI spectra of photoredox reduction of PNA Y1, Y2, Y3, Y4, and Y19 under light irradiation for 30 min.

Note: In condition 2 and 4, these MALDI spectra indicated an aggregation of PNA.

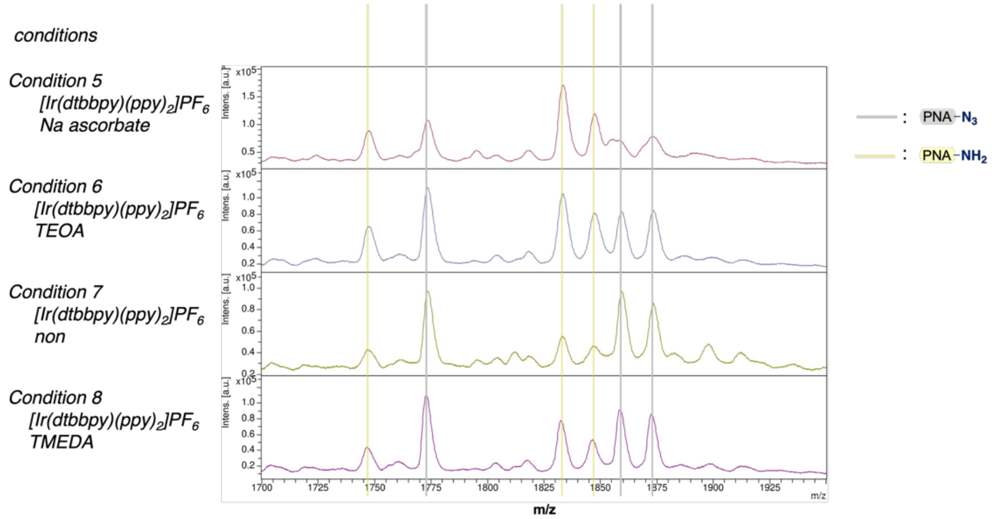

Figure S3-3-2. MALDI spectra of photoredox reduction of PNA Y1, Y3, and Y4 under light irradiation for 30 min.

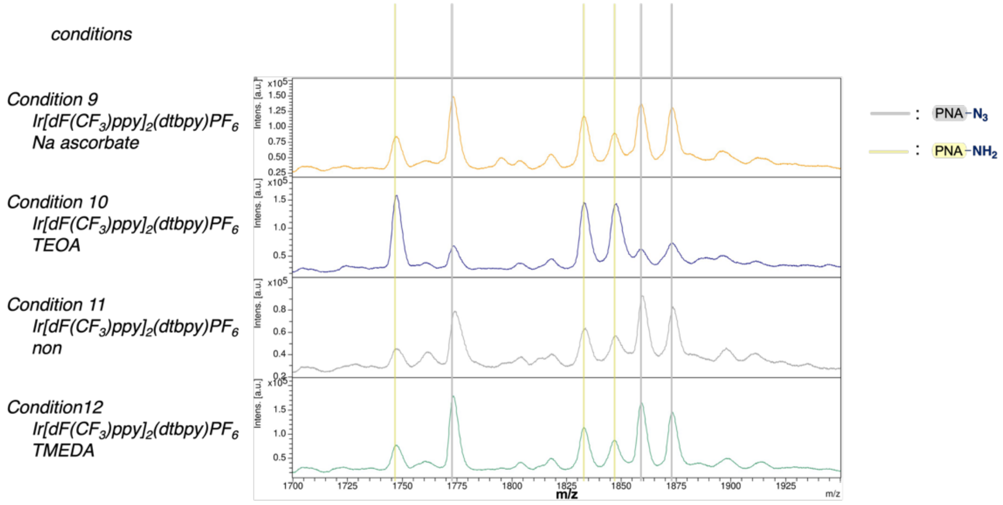

Figure S3-3-3. MALDI spectra of photoredox reduction of PNA Y1, Y3, and Y4 under light irradiation for 30 min.

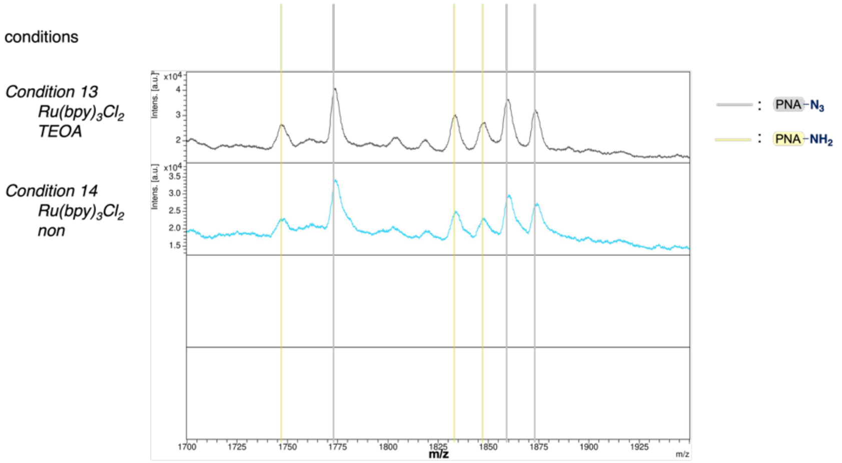

Figure S3-3-4. MALDI spectra of photoredox reduction of PNA Y1, Y3, and Y4 under light irradiation for 30 min.

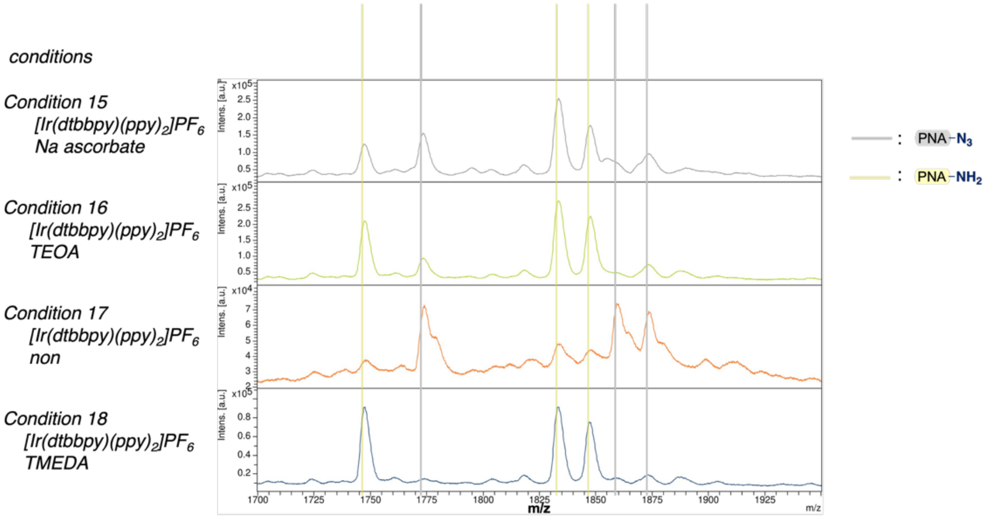

Figure S3-3-5. MALDI spectra of photoredox reduction of PNA Y1, Y3, and Y4 under light irradiation for 30 min.

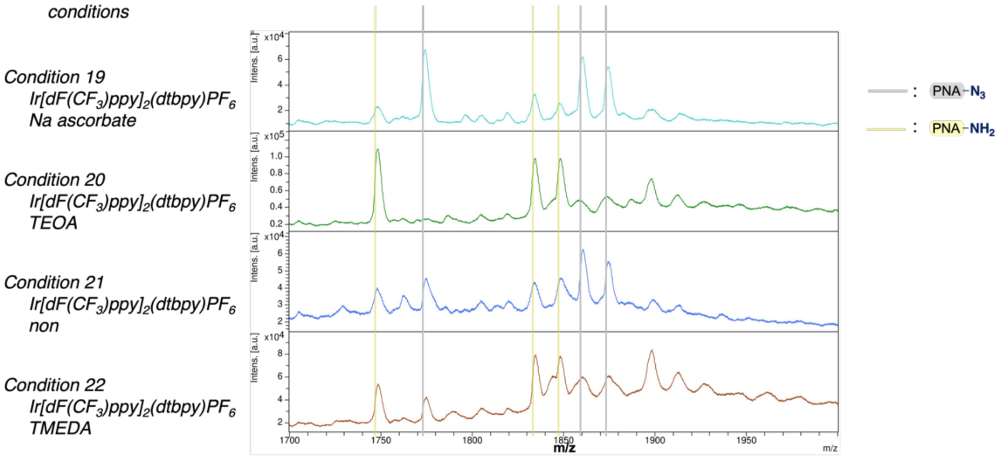

Figure S3-3-6. MALDI spectra of photoredox reduction of PNA Y1, Y3, and Y4 under light irradiation for 30 min.

3.3.3.2. Round 1 of OPMSS under photocatalytic conditions

Table S3-8. Reaction condition for Photoredox reaction: 8 PNAs to 9 PNAs

Name		Volume / μL	Final concentration	Note
100 μM PNA pool 634_**Xa**[*1] or **Xb**[*2] aq. (8 PNAs)		1.5	7.5 μM x 8	
100 μM PNA pool 625_**Y**[*3] aq. (9 PNAs)		1.5	7.5 μM x 9	
Photoredox reaction cocktail A 17 μL	20 mM [Ir(dtbbpy)(ppy)$_2$]PF$_6$ in DMSO	1.0	1.0 mM	15 equiv. / PNA Y
	1 M TEOA aq.	1.0	50 mM	7.4 x 10^2 equiv. / PNA Y
	1.0 M Tris buffer	4.0	200 mM	—
	H_2O	2.0	—	—
	DMSO	9.0	—	—
	Total	20	—	—

[*1]PNA pool 634_**Xa**: **X1**, **X2**, **X3**, **X4**, **X6**, **X7**, **X8**, and **X12**,
[*2]PNA pool 634_**Xb**: **X5**, **X9**, **X10**, **X11**, **X13**, **X14**, **X15**, and **X16**
[*3]PNA pool 625_Y: **Y1**, **Y2**, **Y3**, **Y4**, **Y5**, **Y6**, **Y7**, **Y8**, and **Y9**

Table S3-9. Photoredox reaction cocktail_A

Reagents	Volume / μL	Final concentration
20 mM [Ir(dtbbpy)(ppy)$_2$]PF$_6$ in DMSO	2.5	1.2 mM
1.0 M TEOA	2.5	59 mM
1.0 M Tris buffer	10	0.24 M
H_2O	5.0	—
DMSO	22.5	—
Total	42.5	—

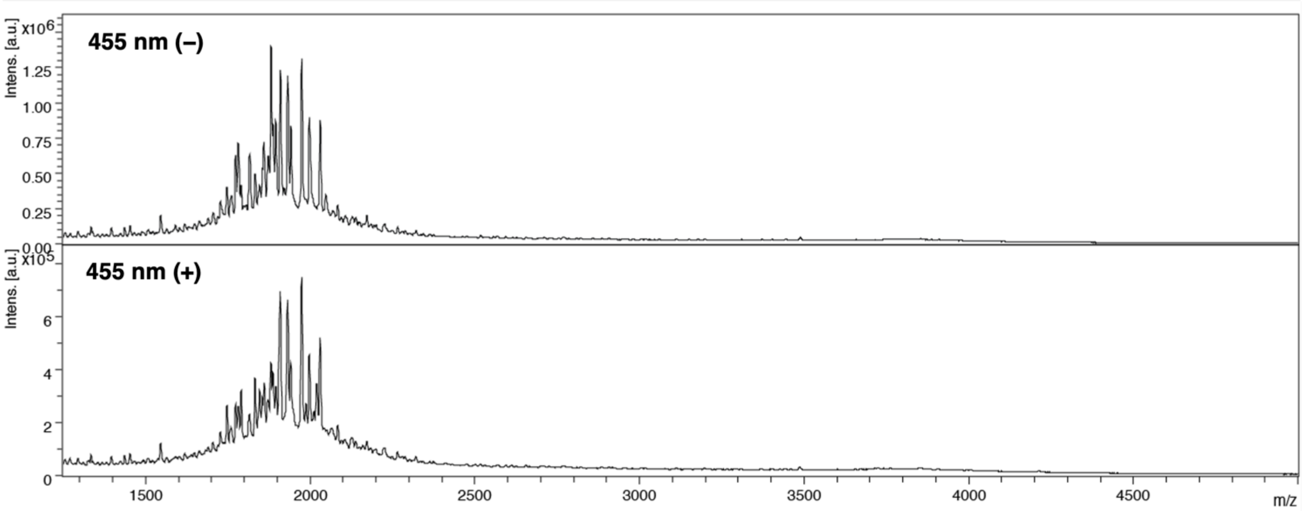

Figure S3-5-1. MALDI spectra of reaction mixtures from eight PNA-Xs (X1–X4, X6–X8, and X12) and nine PNA-Y (Y1–Y9) (a) 455 nm irradiation (−). (b) 455 nm irradiation (+) for 10 min.

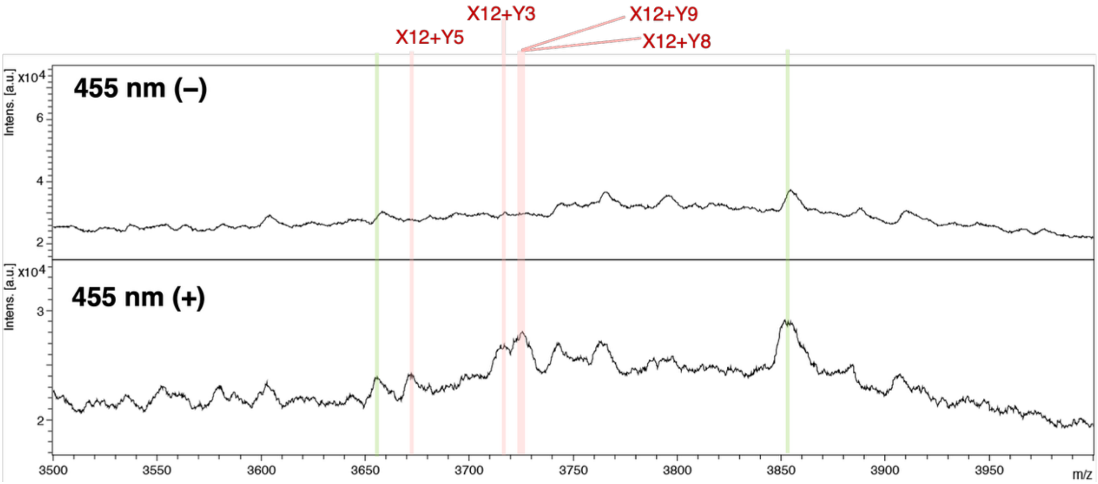

Figure S3-5-2. Expanded MALDI spectra. Upper: 455 nm irradiation (−). Lower spectra: 455 nm irradiation (+) for 10 min. Pink lines: observed ligated products. green lines: below the confidence threshold. Spectra were scaled relative to the base peak.

2.3.3.3. Round 2 of OPMSS under photocatalytic conditions (Fig. 5)

Table S3-10. Reaction condition for Photoredox reaction: one PNA to nine PNAs

Name		Volume / μL	Final concentration	Note
1.0 mM PNA **X**[*1] aq. (one PNA)		1.5	75 μM	1.1 equiv. / PNA Y
100 μM PNA pool 625_**Y**[*2] aq. (nine PNAs)		1.5	7.5 μM x 9	1.0 equiv.
20 mM [Ir(dtbbpy)(ppy)$_2$]PF$_6$ in DMSO[*3]		1.0	1.0 mM	15 equiv. / PNA Y
Photoredox reaction cocktail A or B[*4] 16 μL	TEOA or TMEDA	—	50 mM	7.4 x 10^2 equiv. / PNA Y
	1.0 M Tris buffer	4.0	200 mM	—
	H_2O	3.0	—	—
	DMSO	9.0	—	—
	Total	20	—	—

[*1]PNA X: each solution of **X1**, **X2**, **X3**, **X4**, **X5**, **X6**, **X7**, **X8**, **X9**, **X10**, **X11**, **X12**, **X13**, **X14**, **X15**, or **X16**
[*2]PNA pool 625_Y: **Y1**, **Y2**, **Y3**, **Y4**, **Y5**, **Y6**, **Y7**, **Y8**, and **Y9**
[*3]For a negative control experiment without catalyst, DMSO (1.0 μL) was used instead of 20 mM [Ir(dtbbpy)(ppy)$_2$]PF$_6$ in DMSO.
[*4]These four components were added as 16 μL of photoredox reaction cocktail A or B (see Table S3-11,12) which were prepared prior to use.

Table S3-11. Photoredox reaction cocktail A

Reagents	Volume / μL	Final concentration
1.0 M TEOA	10	63 mM
1.0 M Tris buffer	40	0.25 M
H_2O	20	—
DMSO	90	—
Total	160	—

Table S3-12. Photoredox reaction cocktail B

Reagents	Volume / μL	Final concentration
TMEDA	1.5	63 mM
1.0 M Tris buffer	40	0.25 M
H_2O	30	—
DMSO	88.5	—
Total	160	—

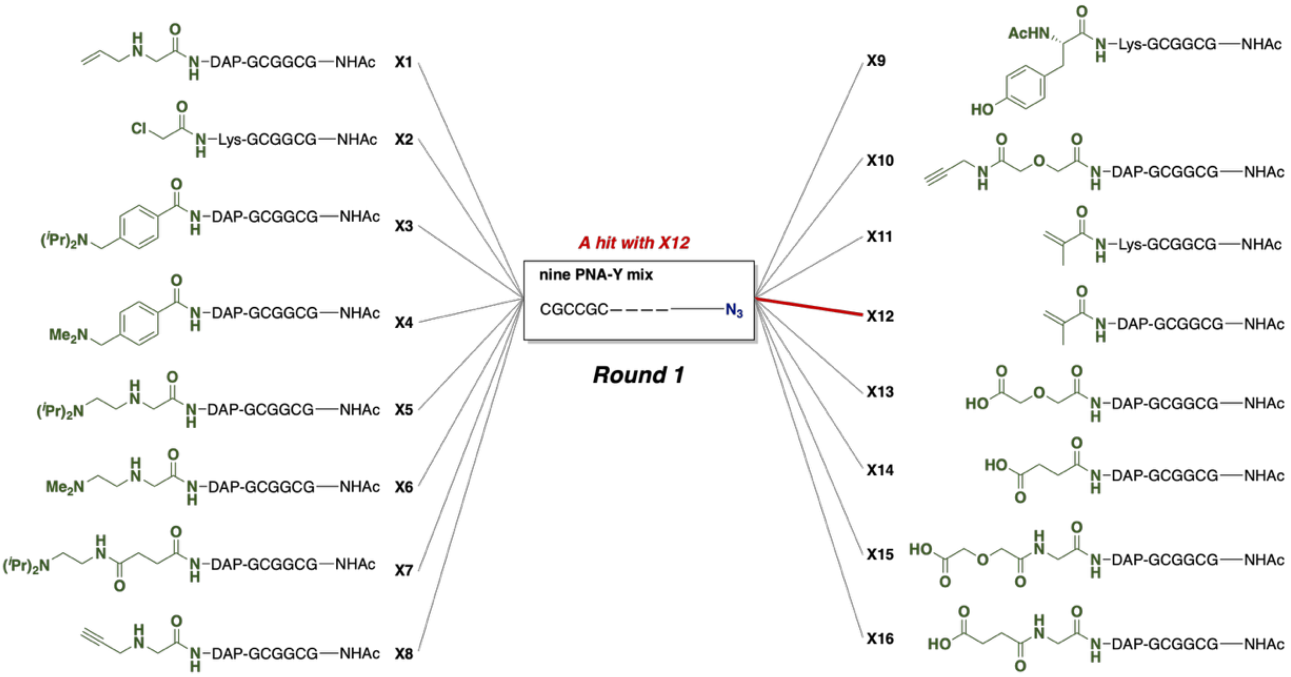

Figure S3-6. Substrate hybridization combinations and ligation outcomes. Gray lines represent possible hybridization combinations, and red lines represent ligated products detected after the reaction

Procedure for Table S3-10

All reagents were mixed. Then, 18 μL of the reaction mixture was irradiated at 455 nm with a collimated LED light 6 cm above the plate. On the other hand, 2 μL of the reaction mixture was incubated in the dark, which was used as a negative control. After being irradiated for 10 min, 30 min, and 3 h, the reaction mixture was analyzed respectively. Results from reaction mixtures using cocktail A are shown as below.

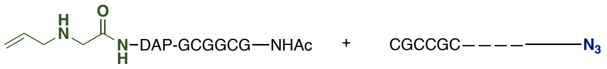

X1 **Y1–9**

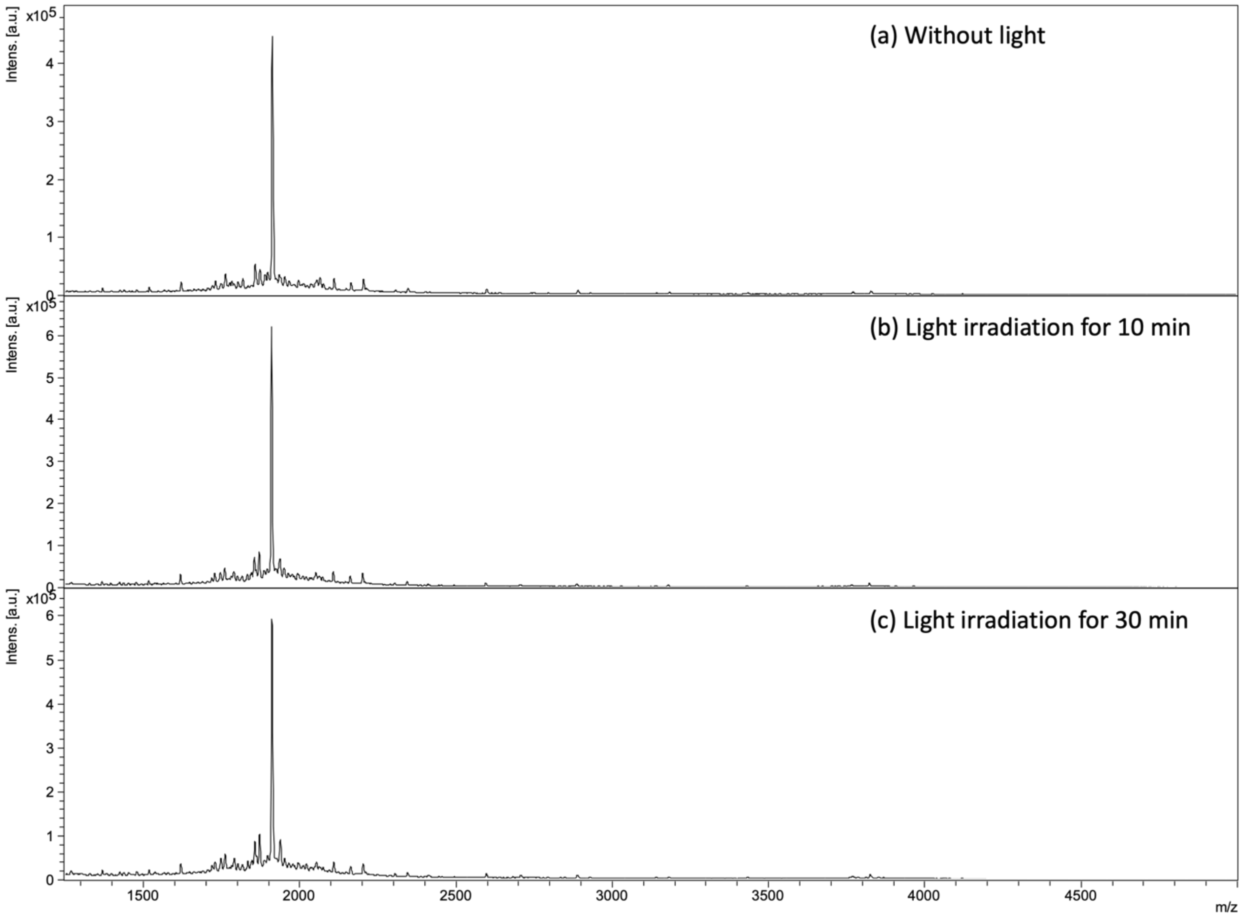

Figure S3-7-1a. MALDI spectra of reaction mixture from PNA X1 and 9 PNA-Ys (a) without light (b) under light irradiation for 10 min or (c) 30 min.

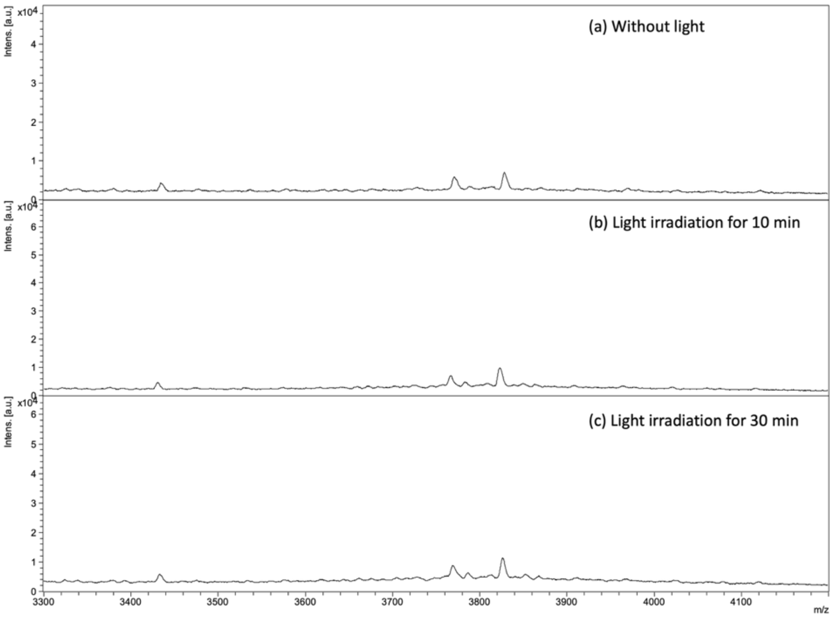

Figure S3-7-1b. Zoomed spectra of Figure S3-7-1a.

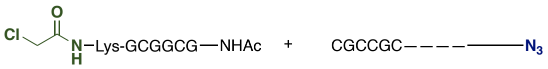

X2 **Y1–9**

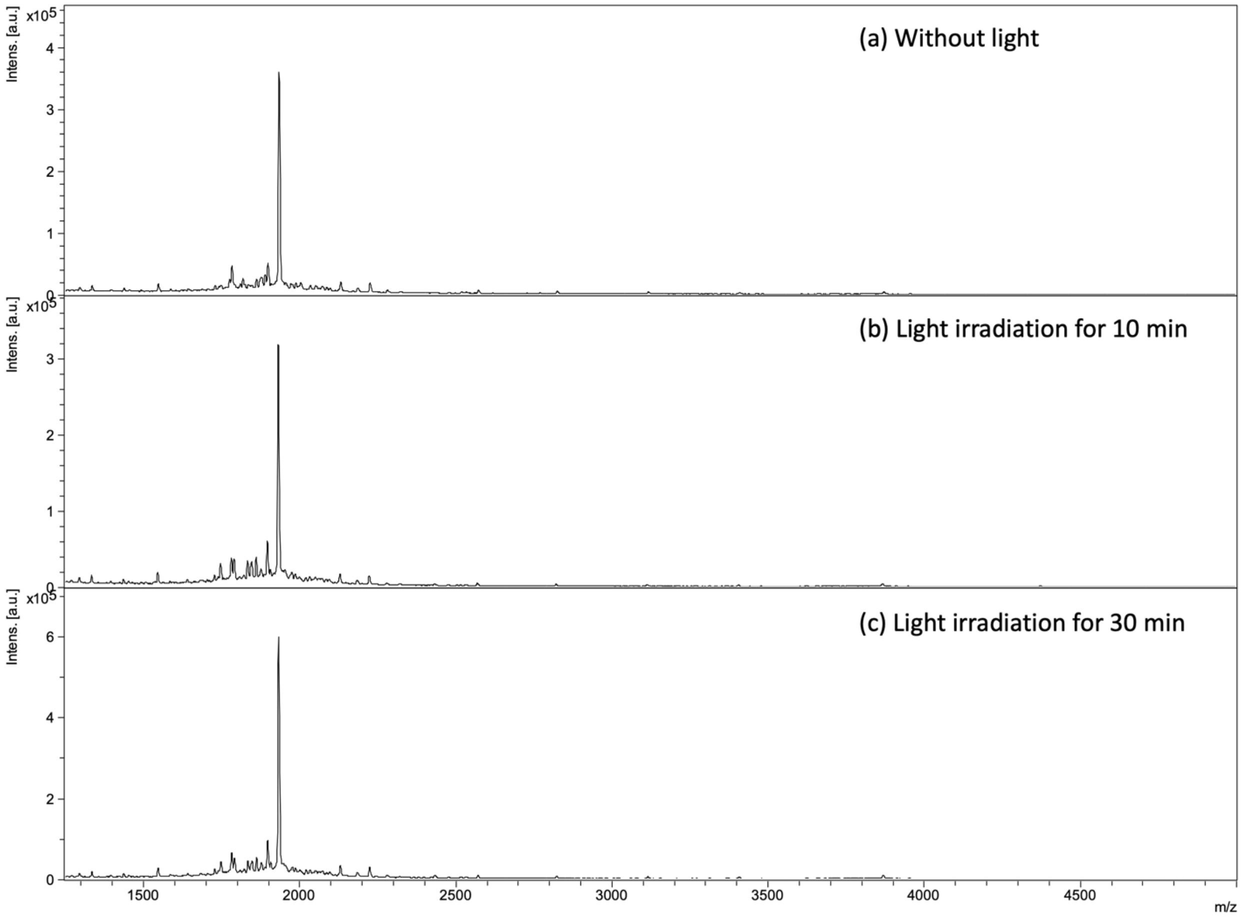

Figure S3-7-2a. MALDI spectra of reaction mixture from PNA X2 and 9 PNA-Ys (a) without light (b) under light irradiation for 10 min or (c) 30 min.

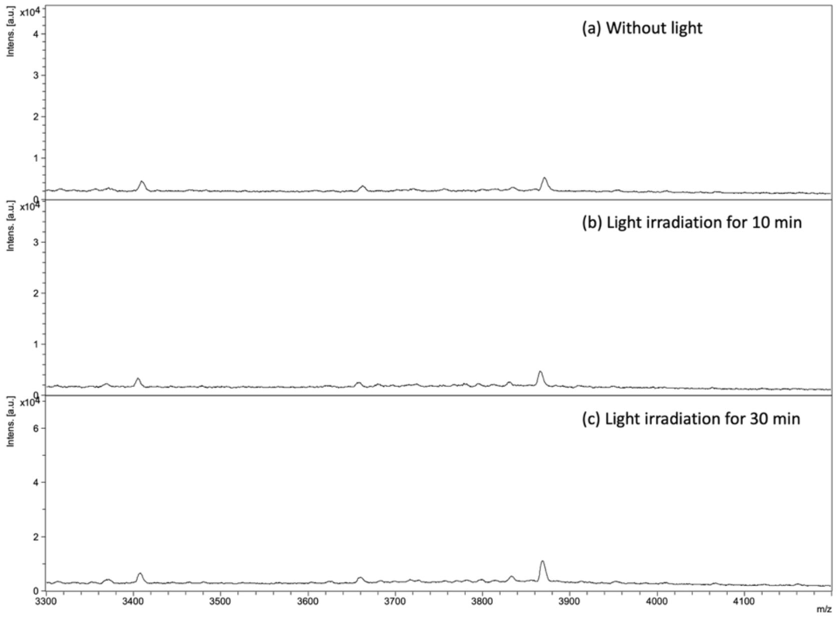

Figure S3-7-2b. Zoomed spectra of Figure S3-7-2a.

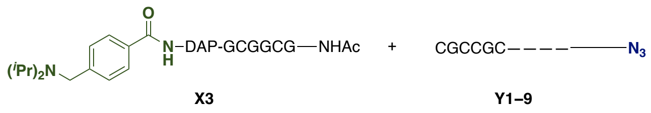

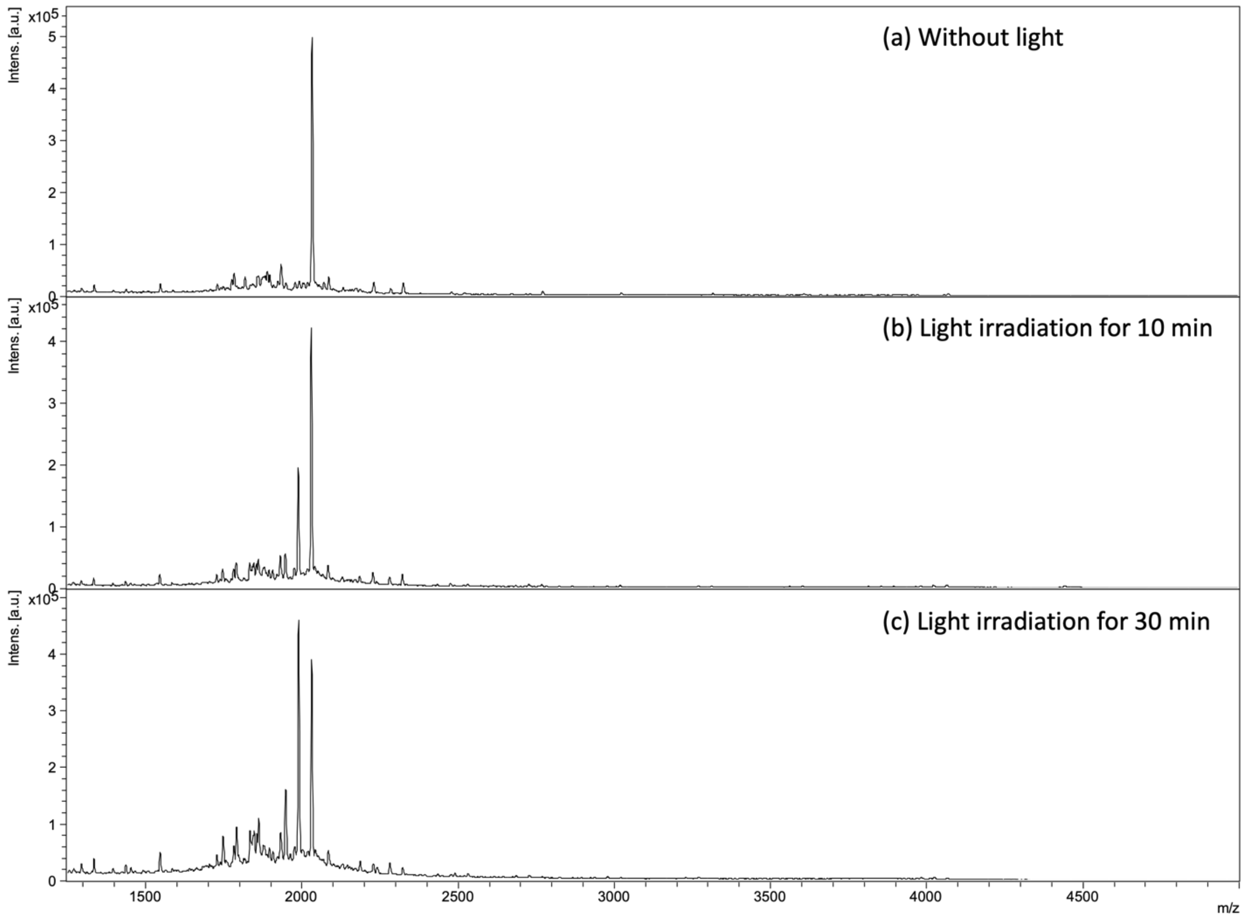

Figure S3-7-3a. MALDI spectra of reaction mixture from PNA X3 and 9 PNA-Ys (a) without light (b) under light irradiation for 10 min or (c) 30 min.

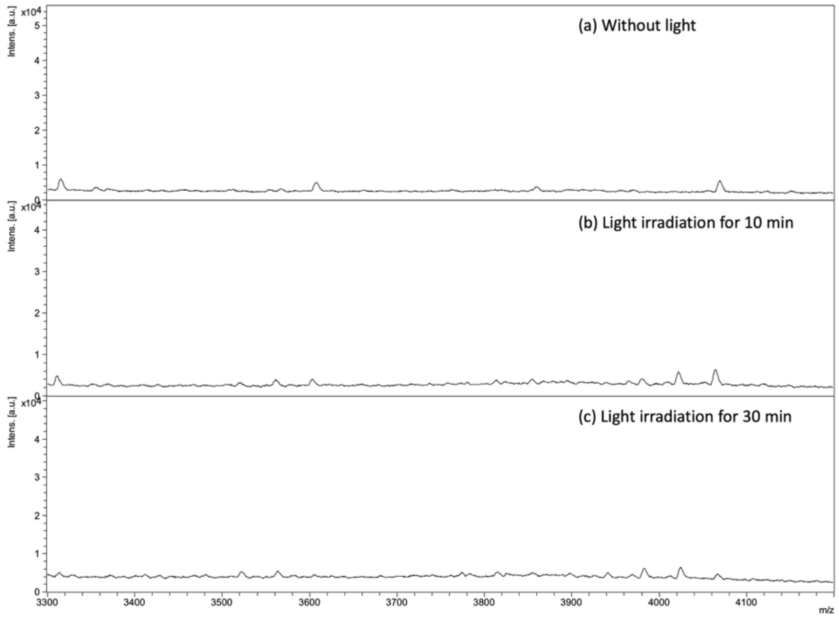

Figure S3-7-3b. Zoomed spectra of Figure S3-7-3a.

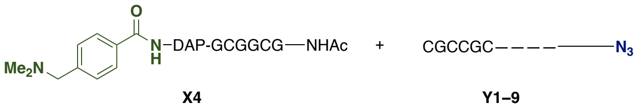

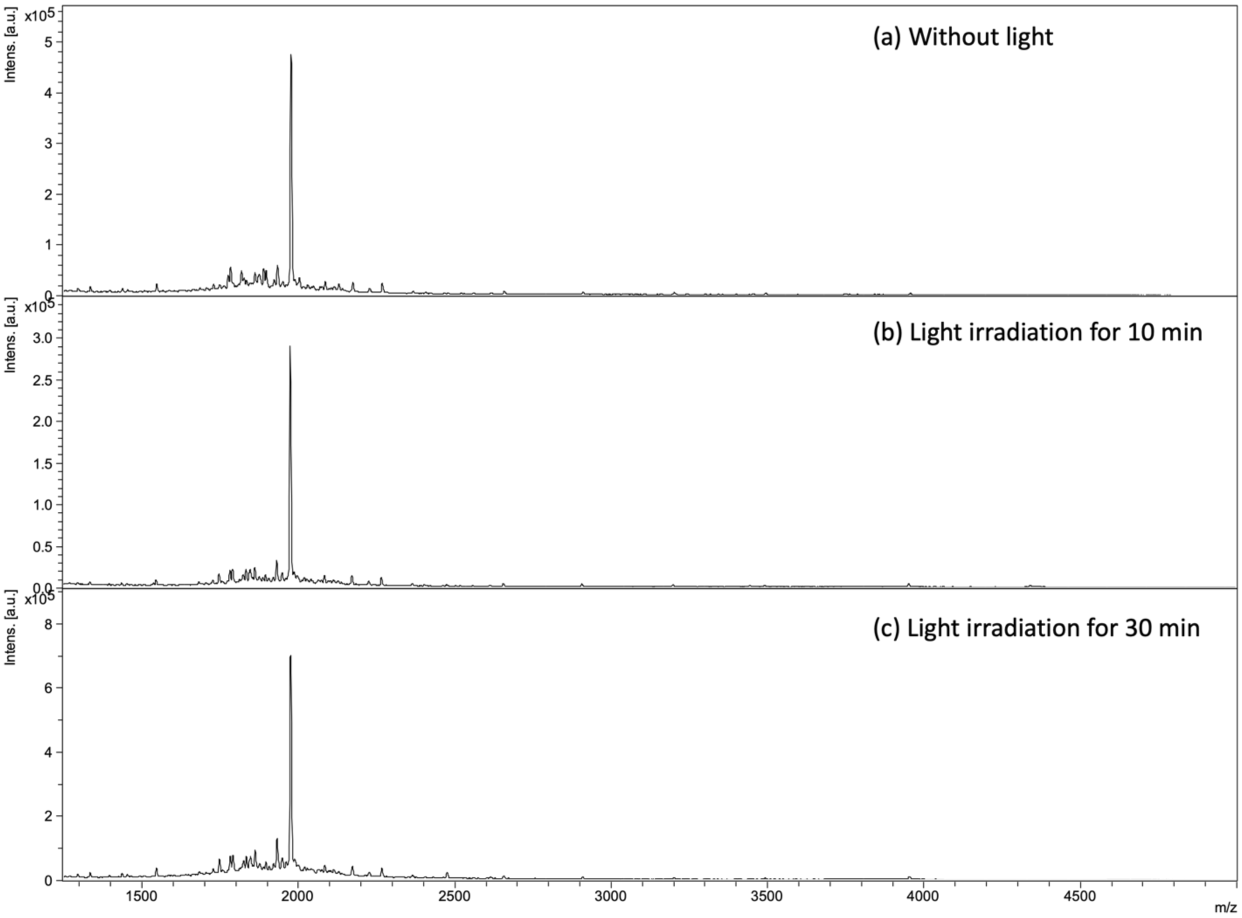

Figure S3-7-4a. MALDI spectra of reaction mixture from PNA X4 and 9 PNA-Ys (a) without light (b) under light irradiation for 10 min or (c) 30 min.

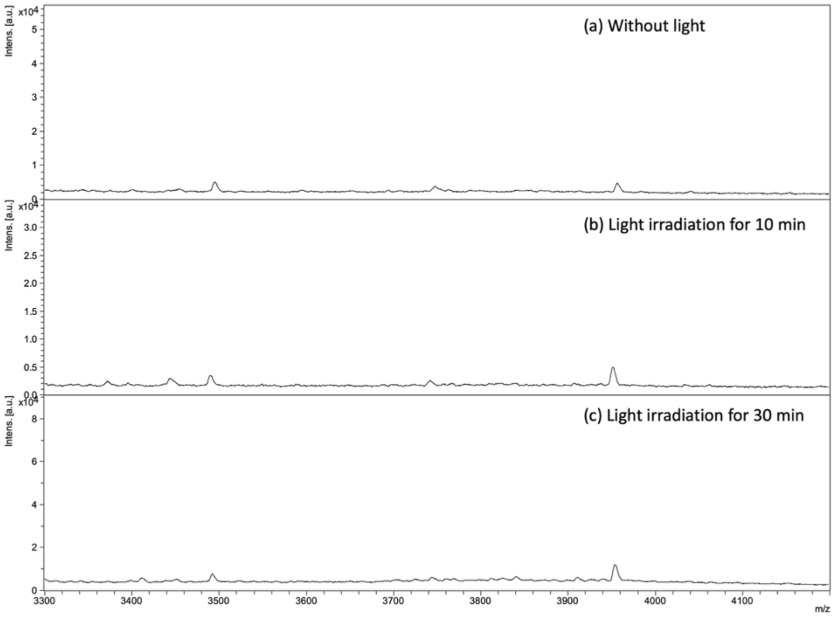

Figure S3-7-4b. Zoomed spectra of Figure S3-7-4a.

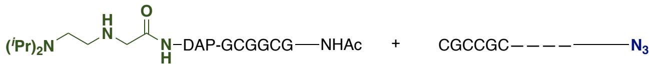

X5 **Y1–9**

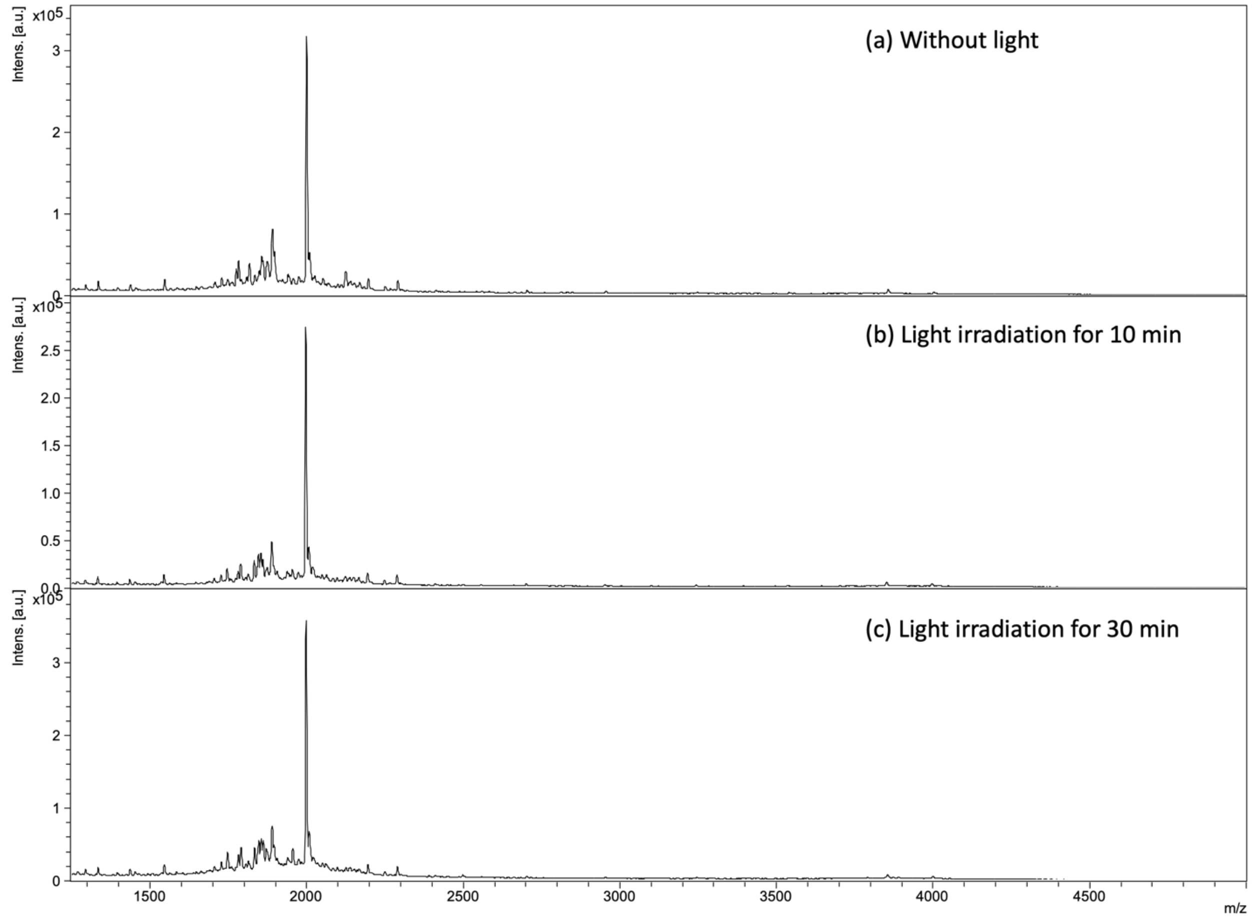

Figure S3-7-5a. MALDI spectra of reaction mixture from PNA X5 and 9 PNA-Ys (a) without light (b) under light irradiation for 10 min or (c) 30 min.

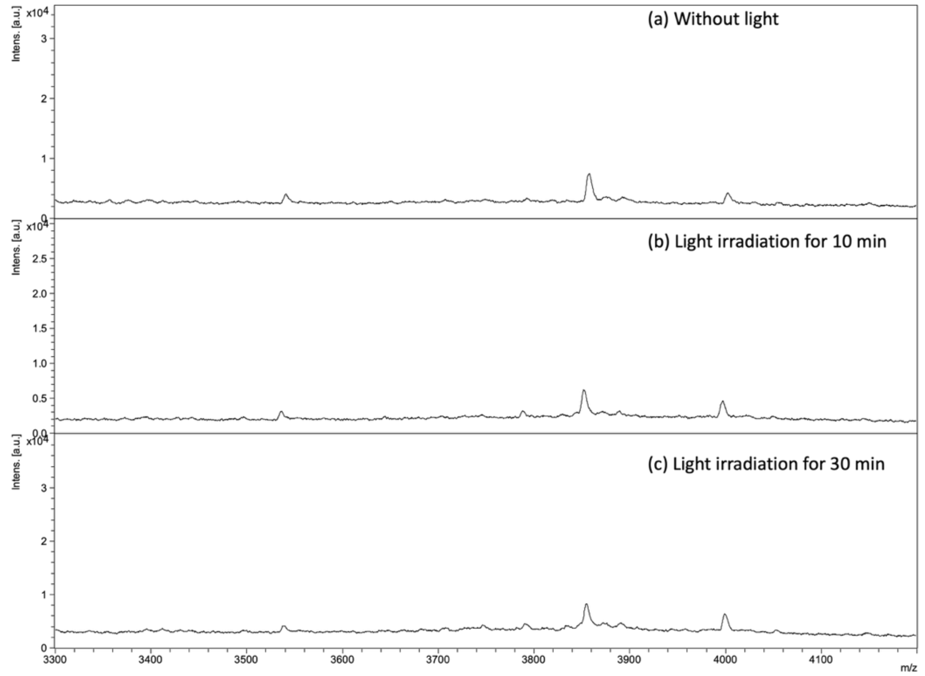

Figure S3-7-5b. Zoomed spectra of Figure S3-7-5a.

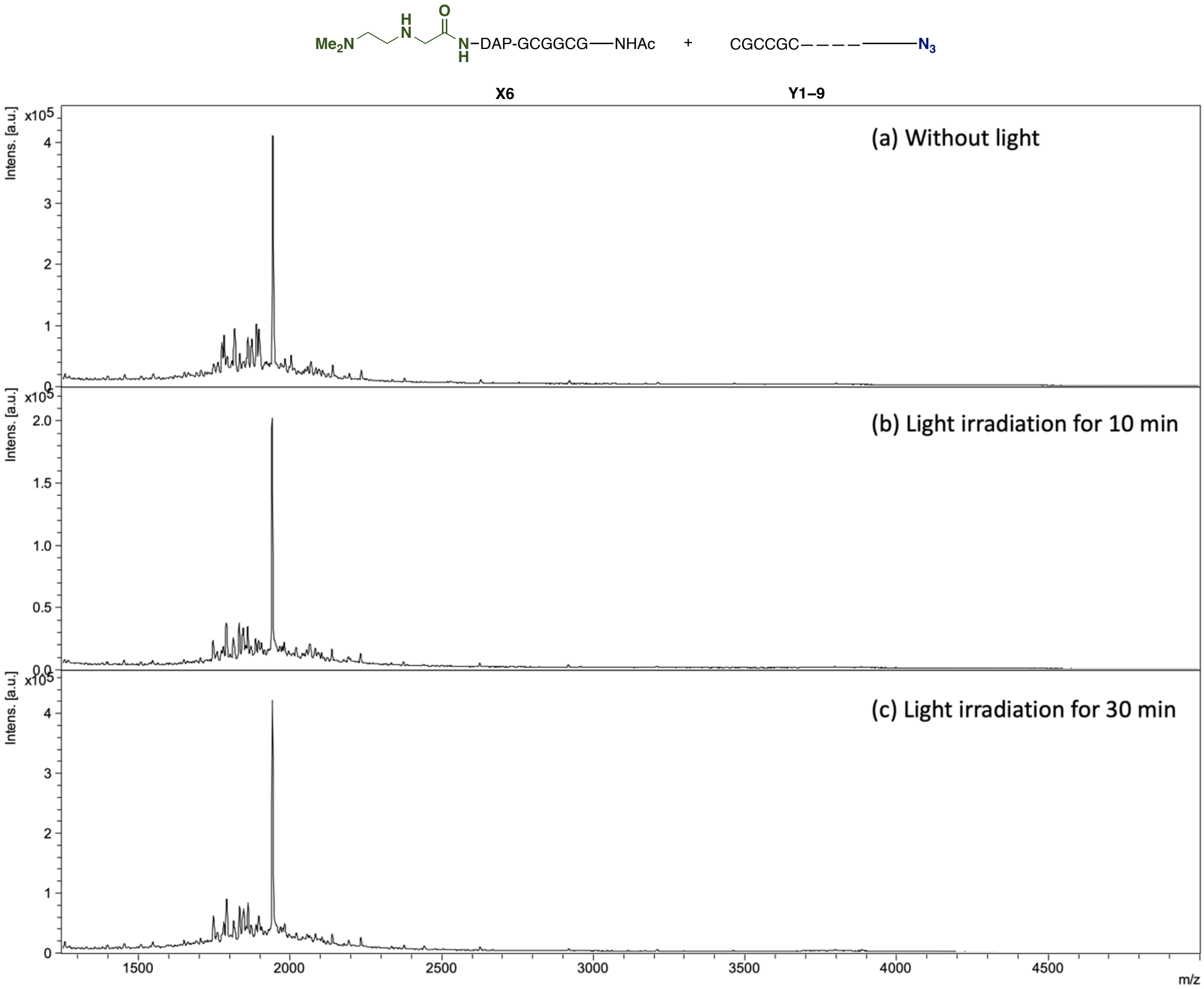

Figure S3-7-6a. MALDI spectra of reaction mixture from PNA X6 and 9 PNA-Ys (a) without light (b) under light irradiation for 10 min or (c) 30 min.

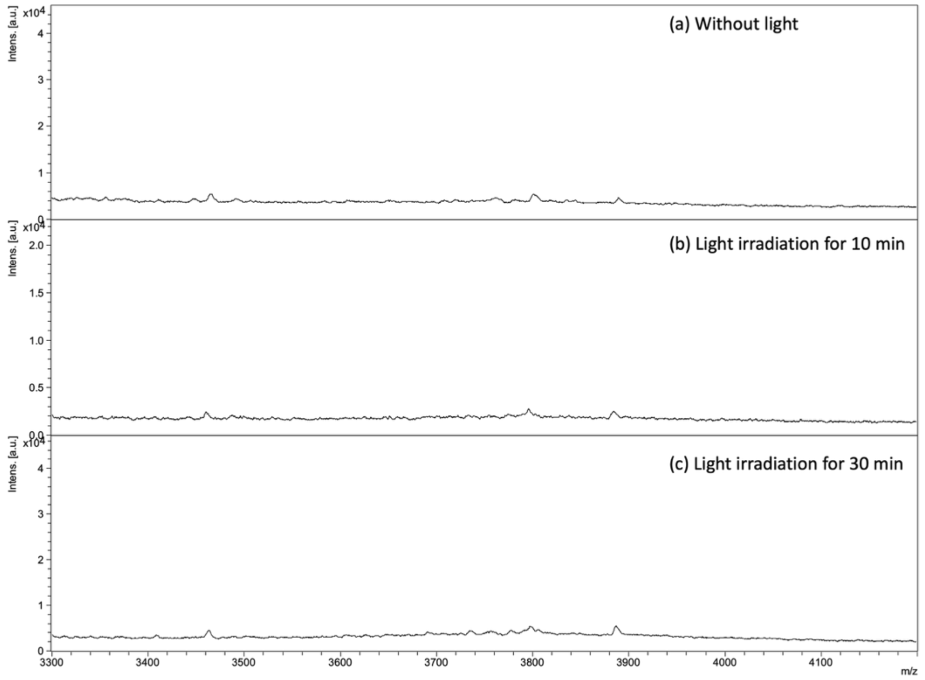

Figure S3-7-6b. Zoomed spectra of Figure S3-7-6a.

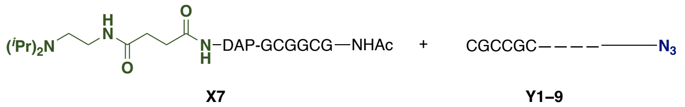

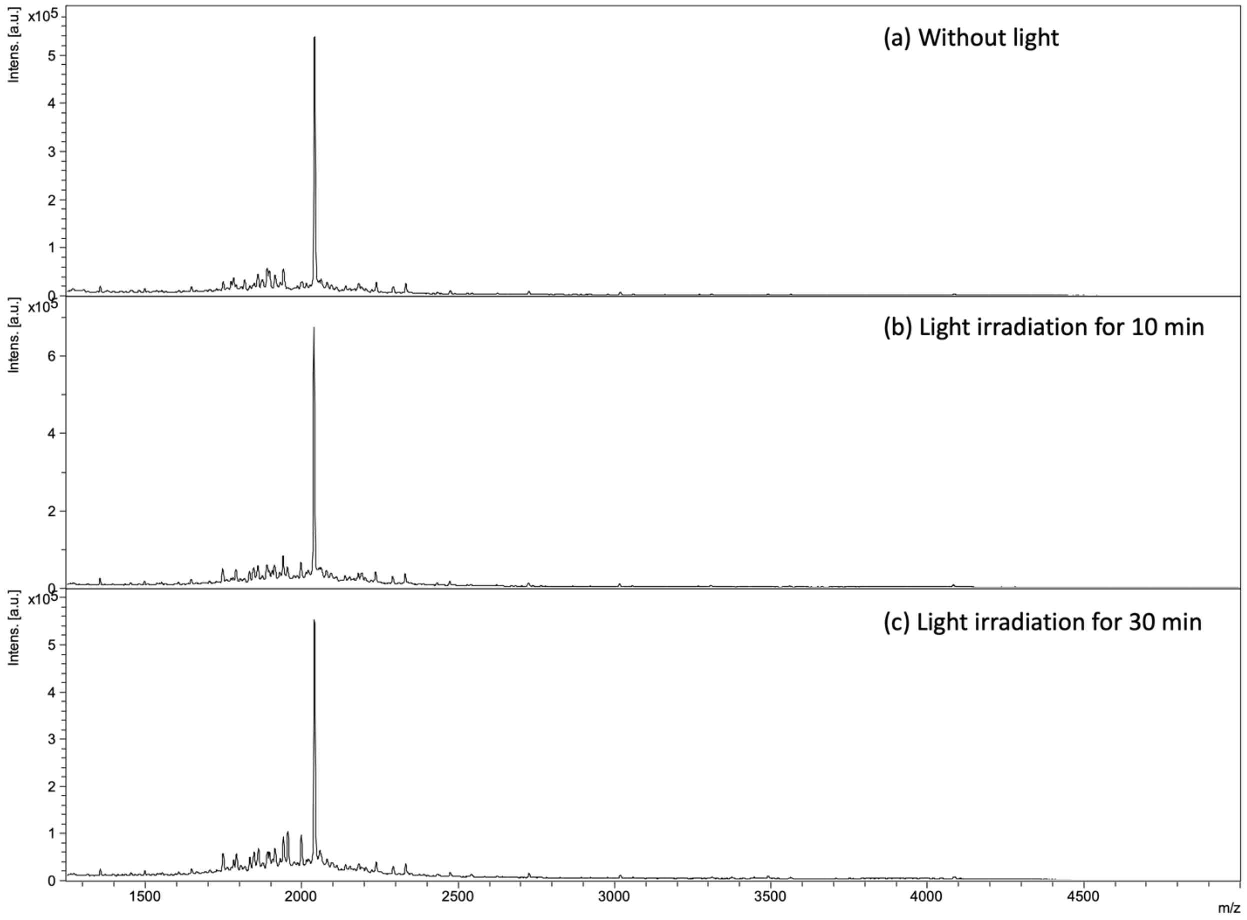

Figure S3-7-7a. MALDI spectra of reaction mixture from PNA X7 and 9 PNA-Ys (a) without light (b) under light irradiation for 10 min or (c) 30 min.

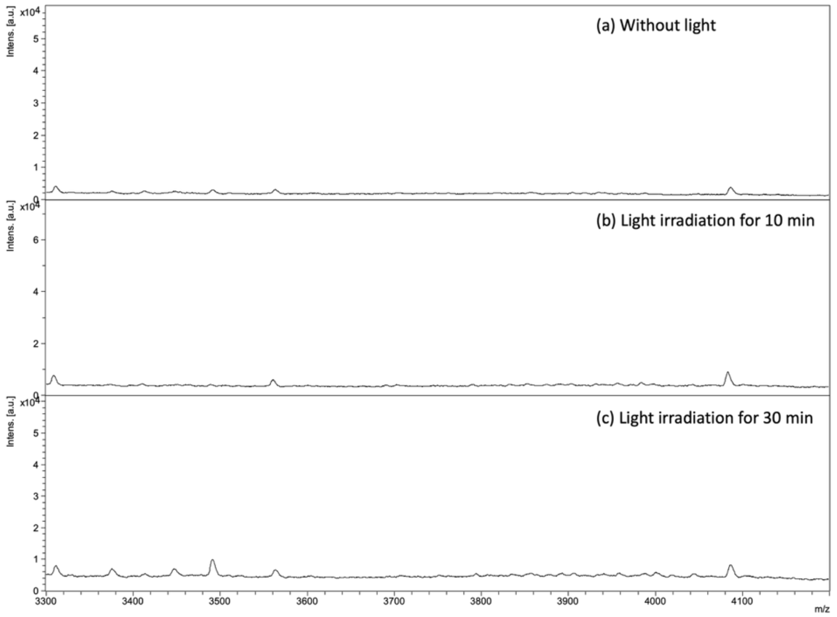

Figure S3-7-7b. Zoomed spectra of Figure S3-7-7a.

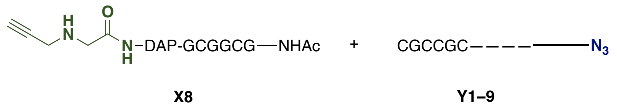

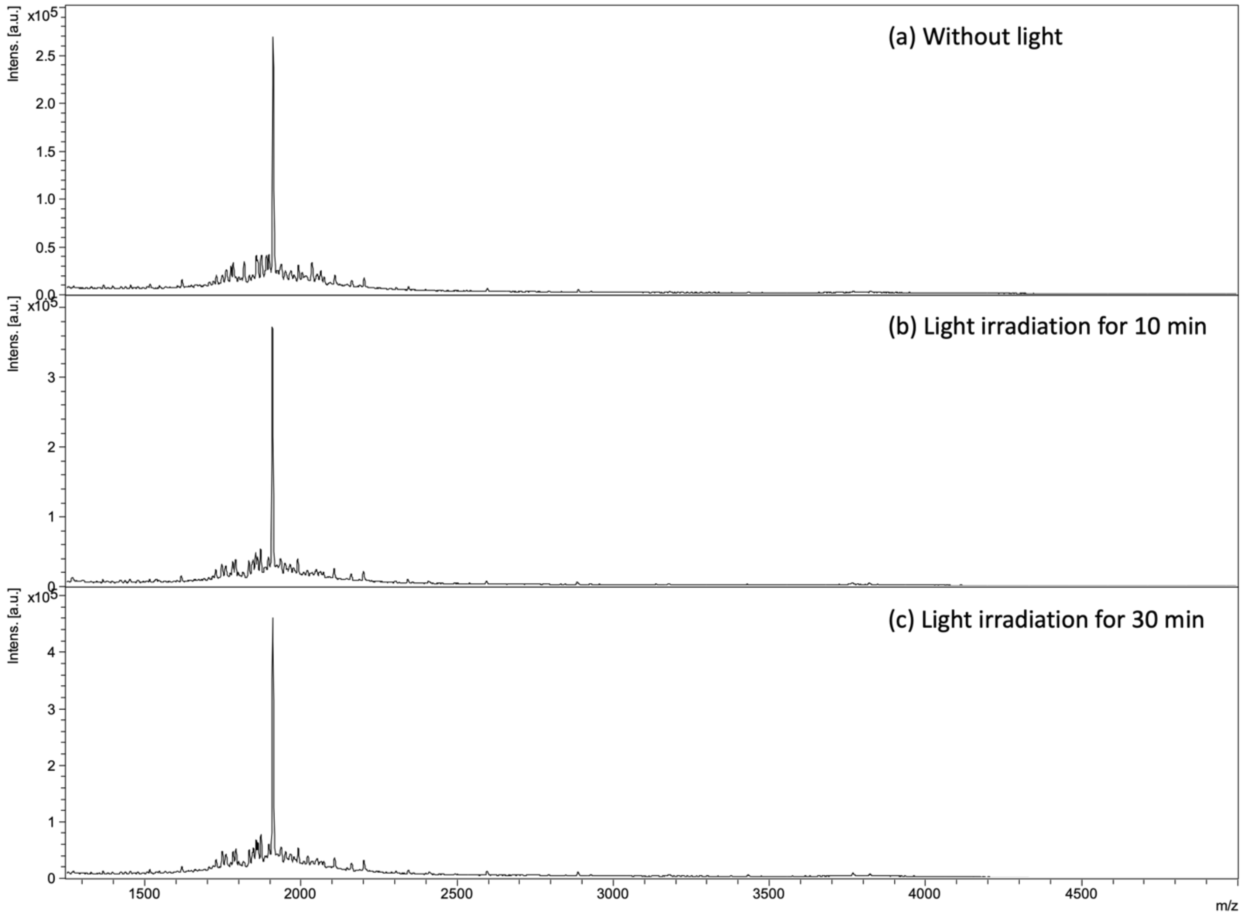

Figure S3-7-8a. MALDI spectra of reaction mixture from PNA X8 and 9 PNA-Ys (a) without light (b) under light irradiation for 10 min or (c) 30 min.

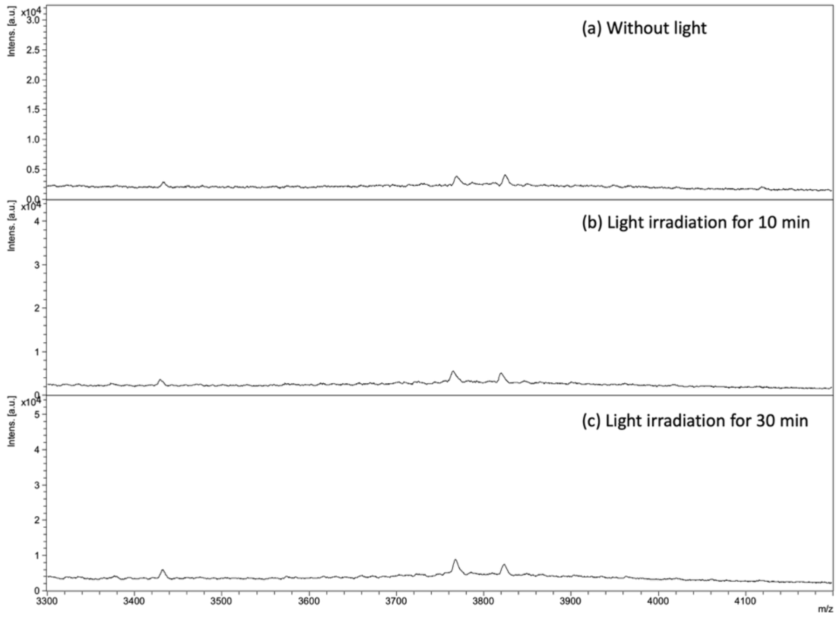

Figure S3-7-8b. Zoomed spectra of Figure S3-7-8a.

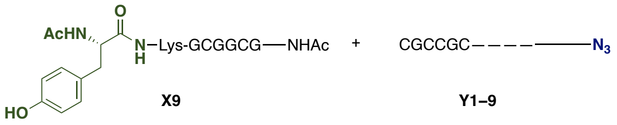

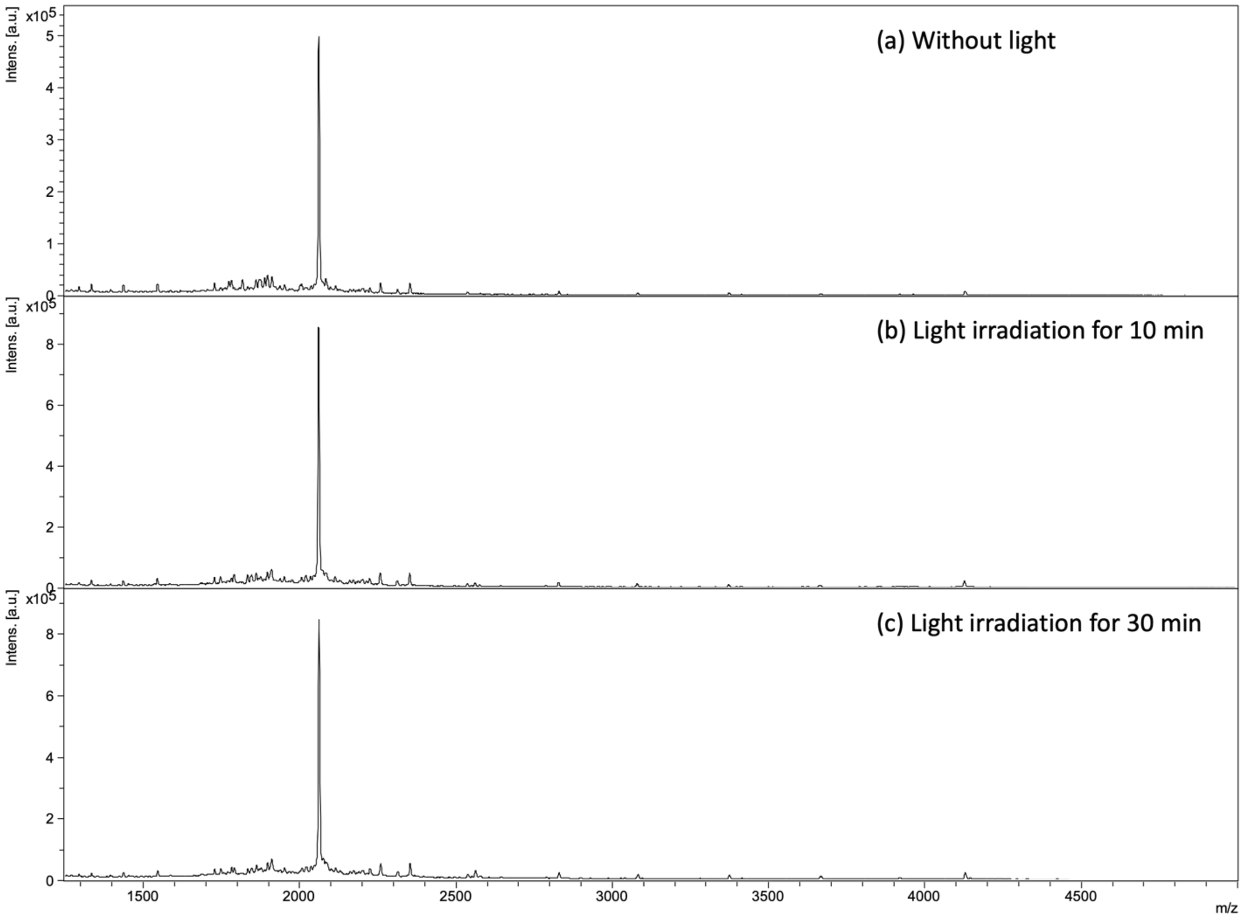

Figure S3-7-9a. MALDI spectra of reaction mixture from PNA X9 and 9 PNA-Ys (a) without light (b) under light irradiation for 10 min or (c) 30 min.

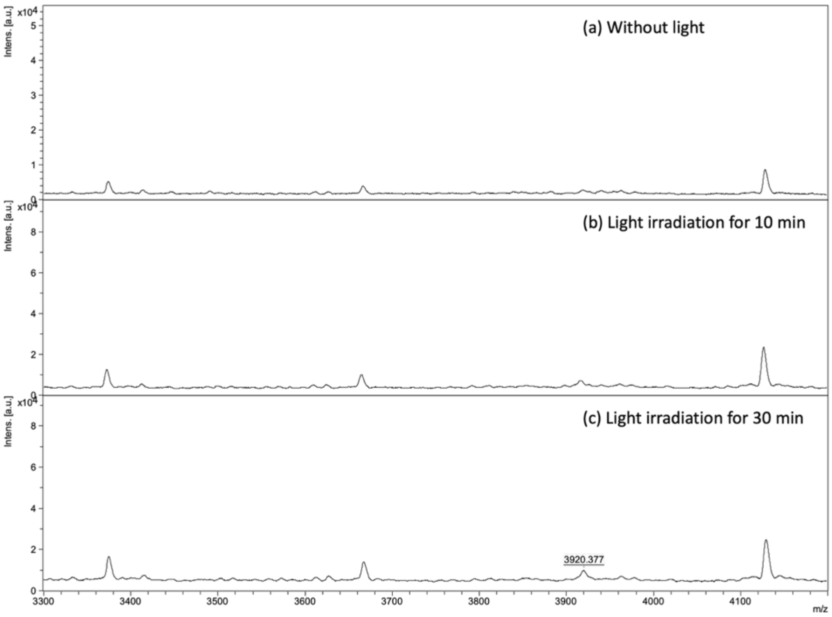

Figure S3-7-9b. Zoomed spectra of Figure S3-7-9a.

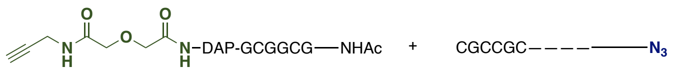

X10 **Y1–9**

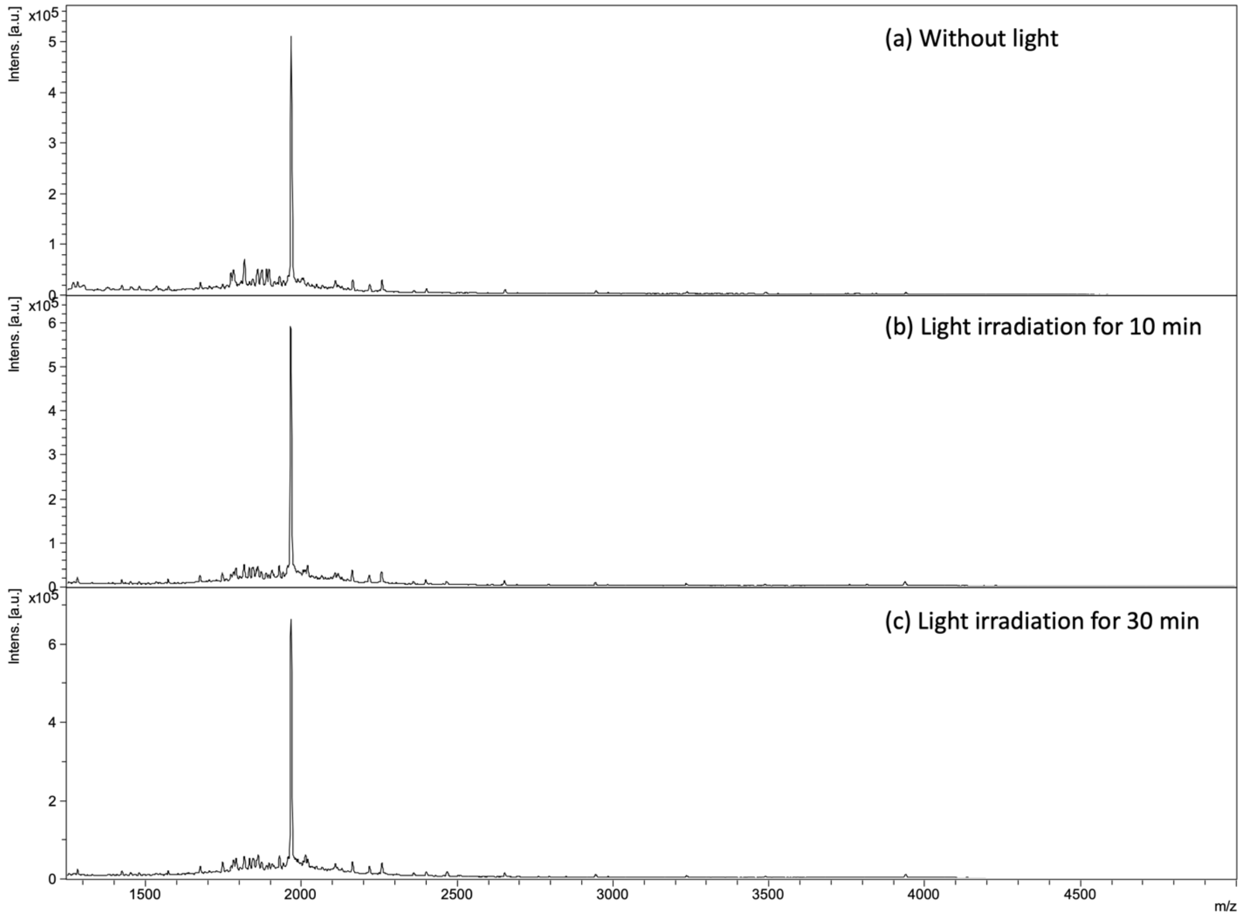

Figure S3-7-10a. MALDI spectra of reaction mixture from PNA X10 and 9 PNA-Ys (a) without light (b) under light irradiation for 10 min or (c) 30 min.

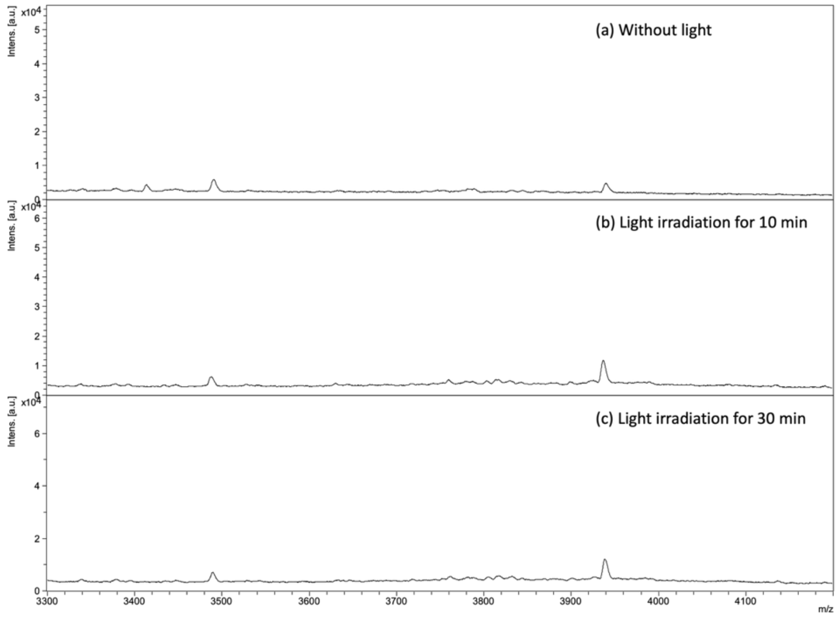

Figure S3-7-10b. Zoomed spectra of Figure S3-7-10a.

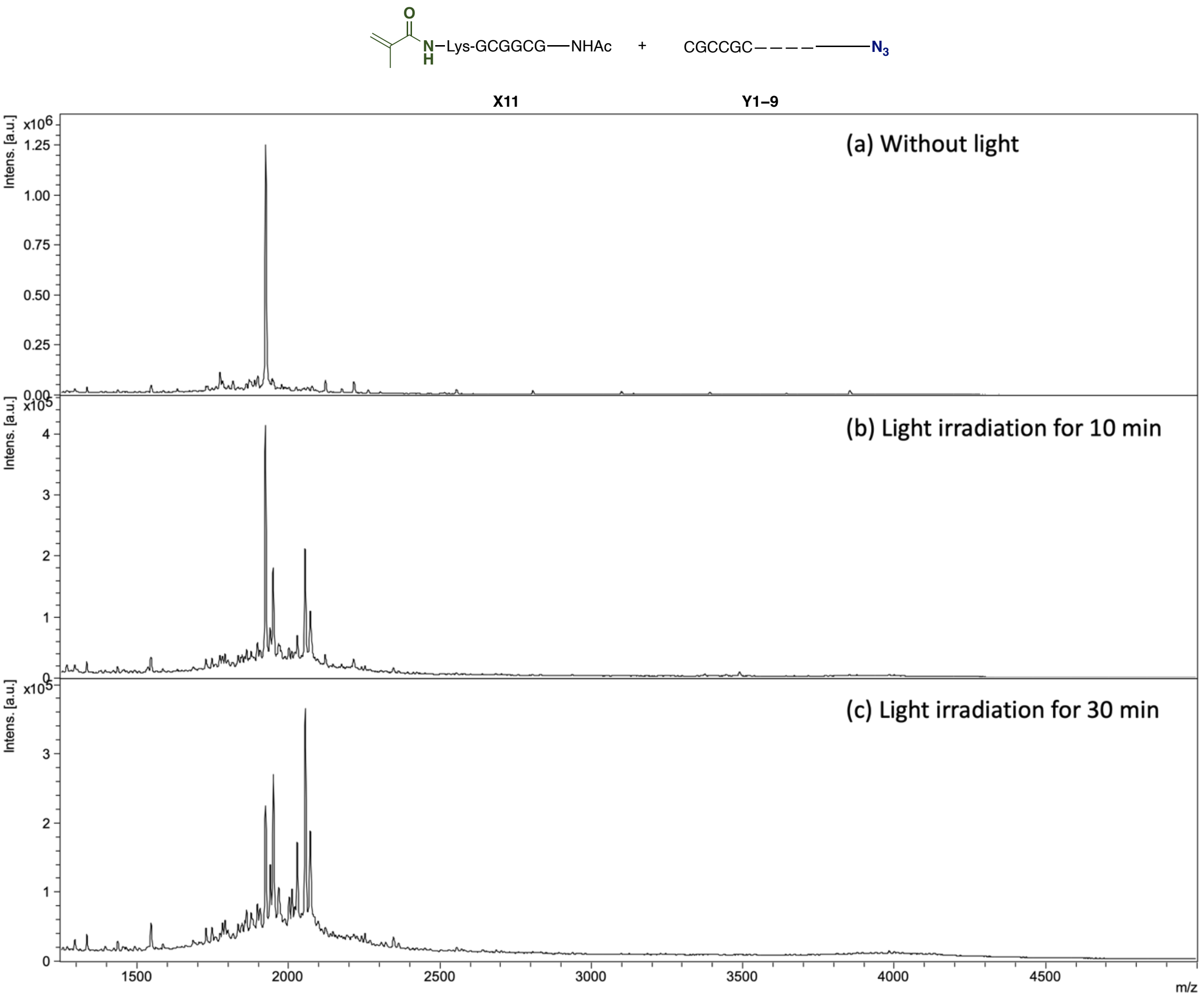

Figure S3-7-11a. MALDI spectra of reaction mixture from PNA X11 and 9 PNA-Ys (a) without light (b) under light irradiation for 10 min or (c) 30 min.

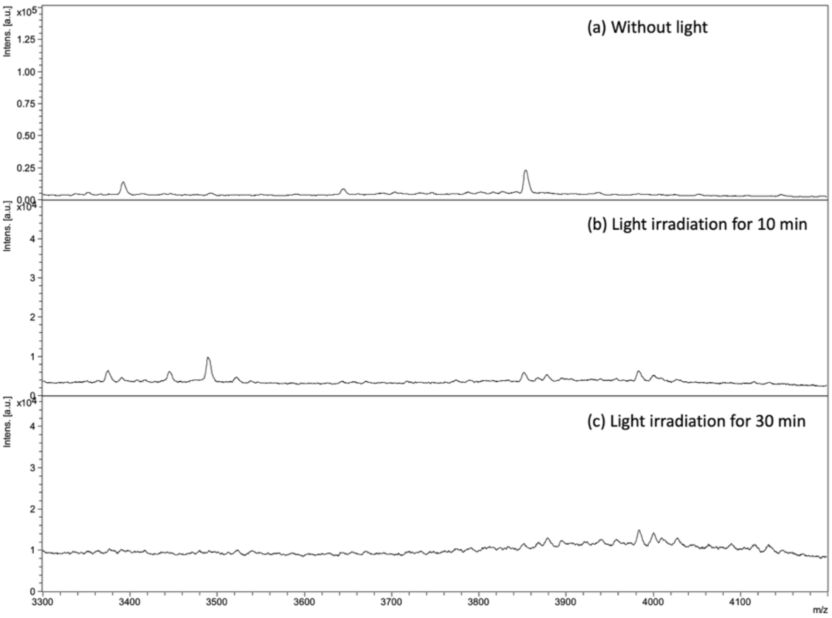

Figure S3-7-11b. Zoomed spectra of Figure S3-7-11a.

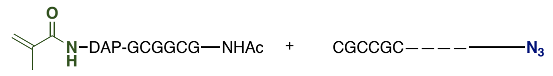

X12 **Y1–9**

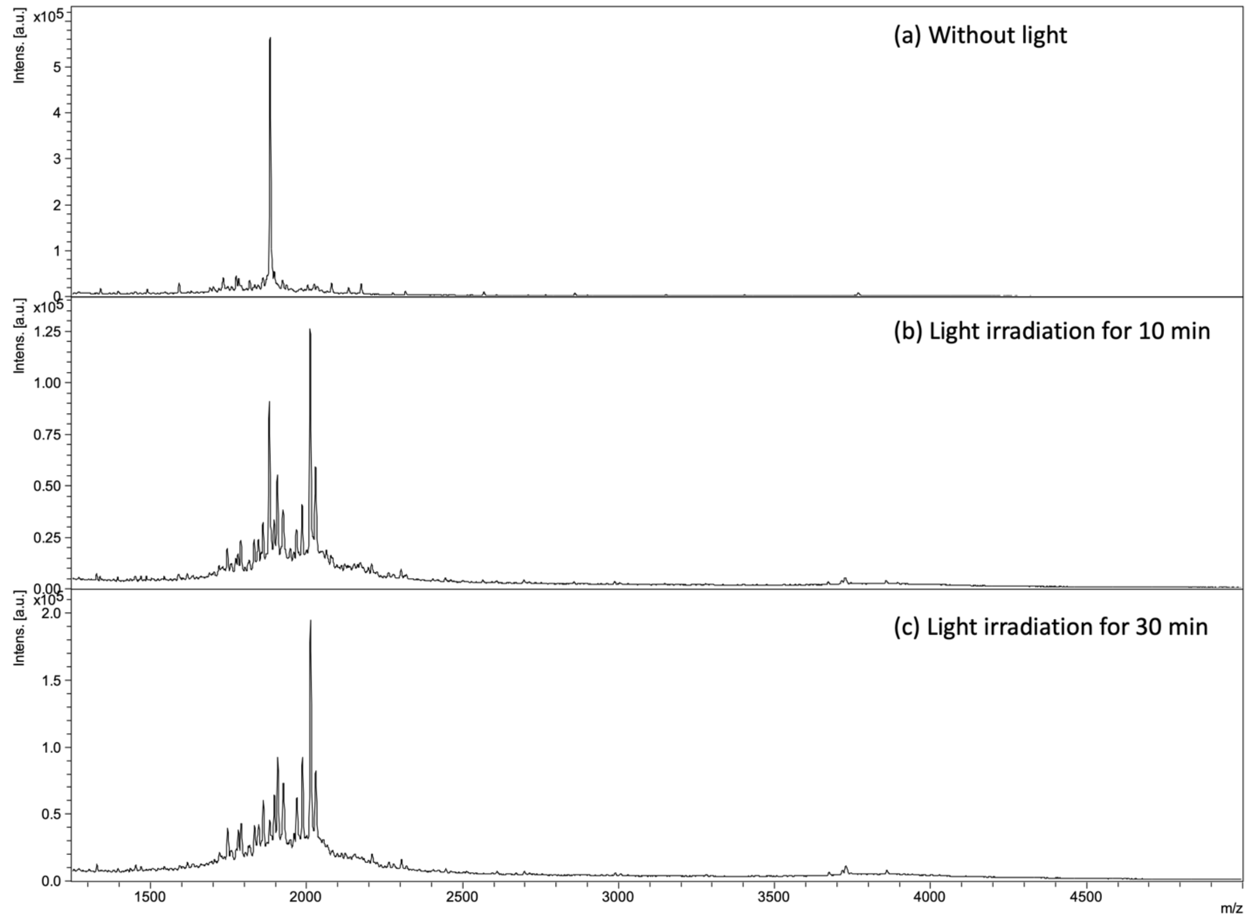

Figure S3-7-12a. MALDI spectra of reaction from PNA X12 and 9 PNA-Ys (a) without light (b) under light irradiation for 10 min or (c) 30 min.

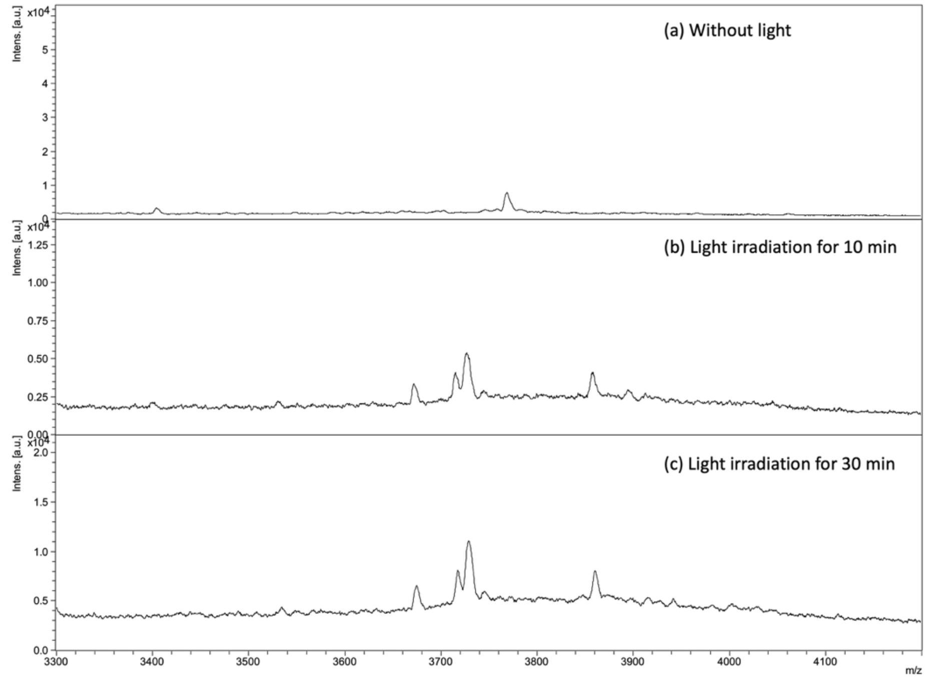

Figure S3-7-12b. Zoomed spectra of Figure S3-7-12a.

Table S3-13. List of observed peaks and assigned PNA sequences: Photoredox reaction (+ light), 10 minutes

found. m/z Reflection mode*	assigned PNA	assigned PNA sequence (N to C)	Molecular formula	calc. m/z monoisotopic mass $[M+H]^+$	error	colour
3713.32	X12 + Y3	GCGGCG-DAP-N-**C1**-Gly-Gly-Gly-CGCCGC	C143H186N80O43	3712.49	0.83	
peak overlap	X12 + Y4	GCGGCG-DAP-N-**C1(Me)**-Gly-Gly-Gly-CGCCGC	C144H188N80O43	3726.51	#######	
3670.30	X12 + Y5	GCGGCG-DAP-N-**C1(Me)**-Gly-Gly-CGCCGC	C142H185N79O42	3669.48	0.82	
3855.47	X12 + Y8	GCGGCG-DAP-N-**C1(NMe2)**-Gly-Gly-Gly-Gly-CGCCGC	C150H200N82O44	3854.60	0.87	
3723.35	X12 + Y9	GCGGCG-DAP-N-**C1(CN)**-Gly-Gly-CGCCGC	C145H188N80O42	3722.52	0.83	

Reflector positive-ion mode was utilized for Table S3-13.

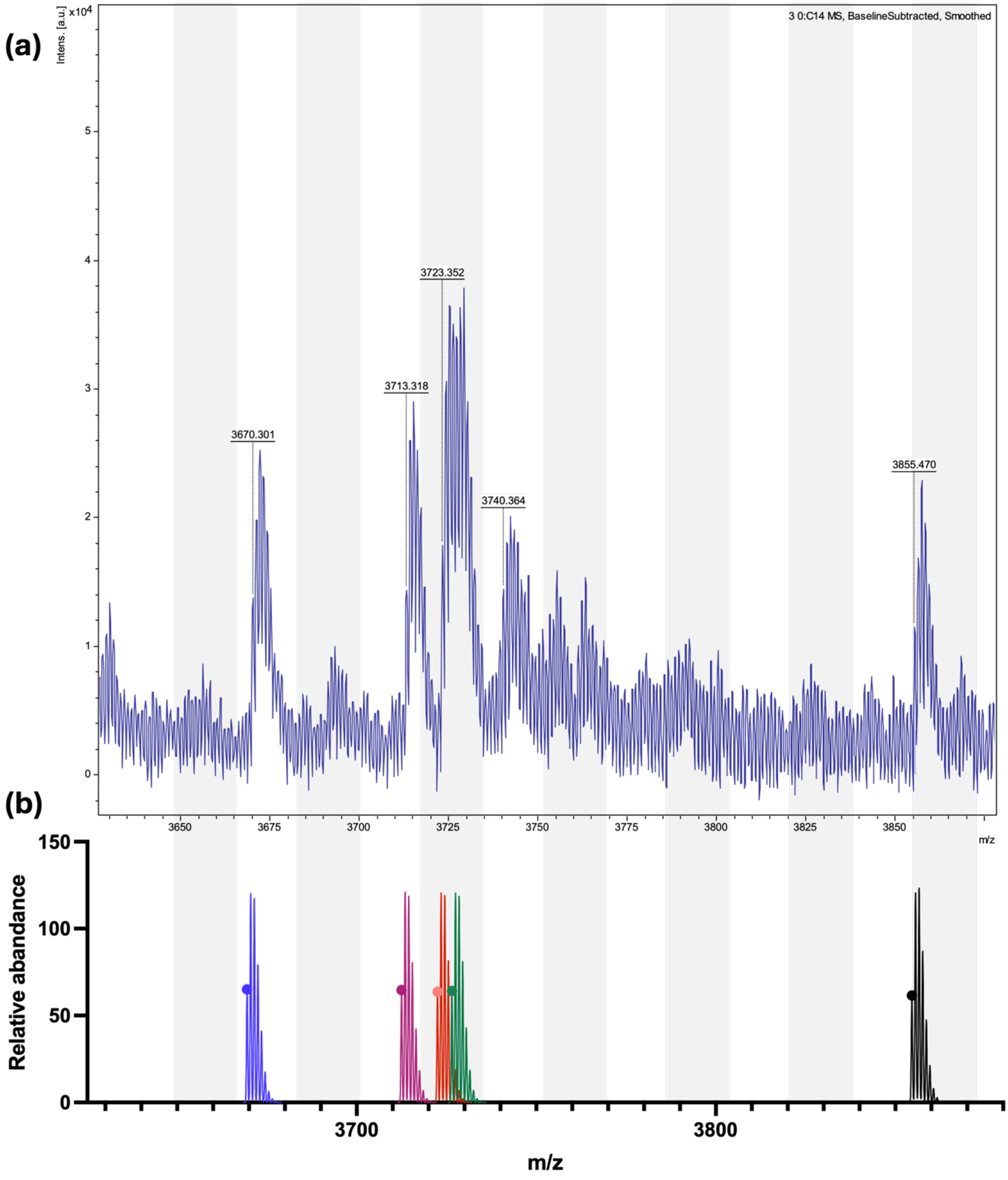

Figure S3-7-12c. MALDI spectra of the reaction mixture from PNA X12 and 9 PNA-Ys (Reflector positive-ion mode). (a) Observed spectra, (b) Calculated spectra.

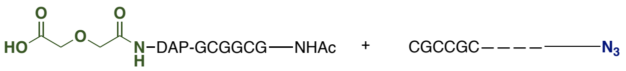

X13 **Y1–9**

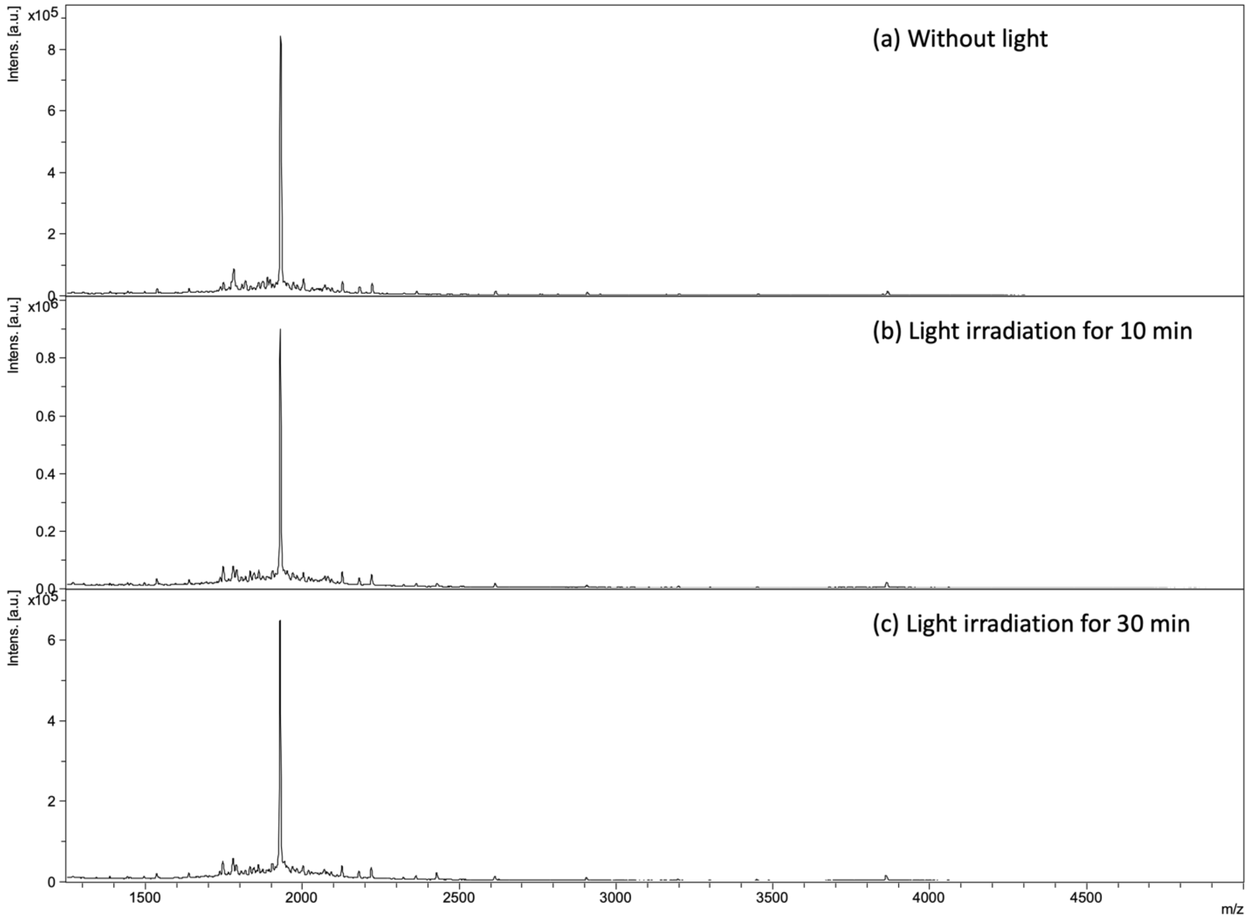

Figure S3-7-13a. MALDI spectra of reaction mixture from PNA X13 and 9 PNA-Ys (a) without light (b) under light irradiation for 10 min or (c) 30 min.

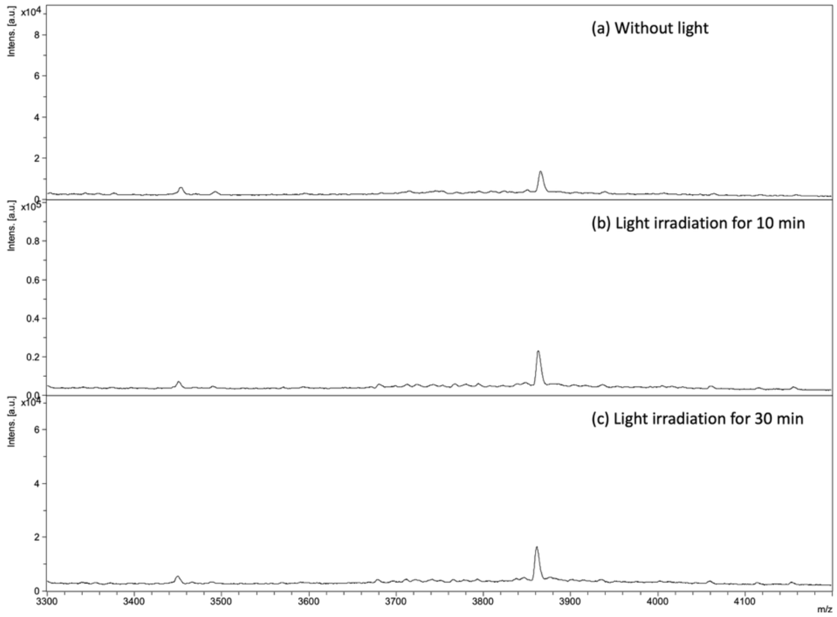

Figure S3-7-13b. Zoomed spectra of Figure S3-7-13a.

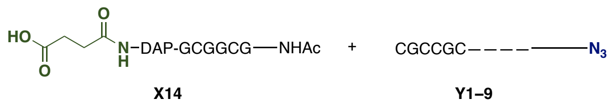

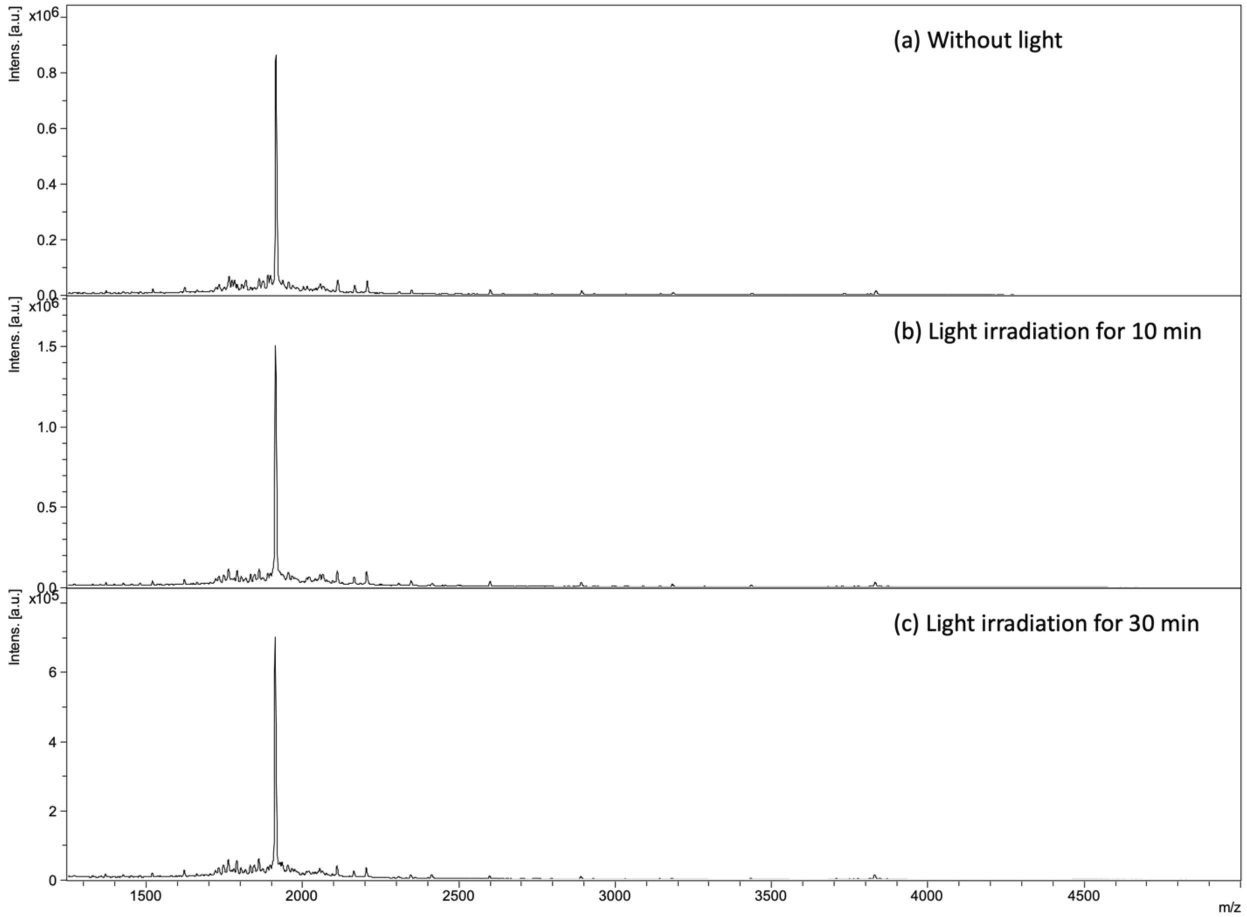

Figure S3-7-14a. MALDI spectra of reaction mixture from PNA X14 and 9 PNA-Ys (a) without light (b) under light irradiation for 10 min or (c) 30 min.

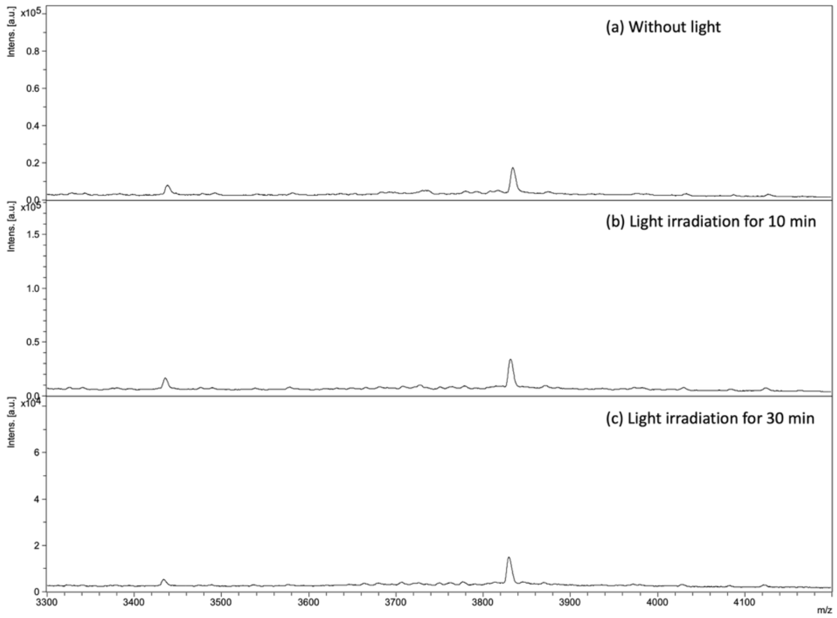

Figure S3-7-14b. Zoomed spectra of Figure S3-7-14a.

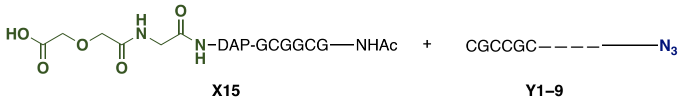

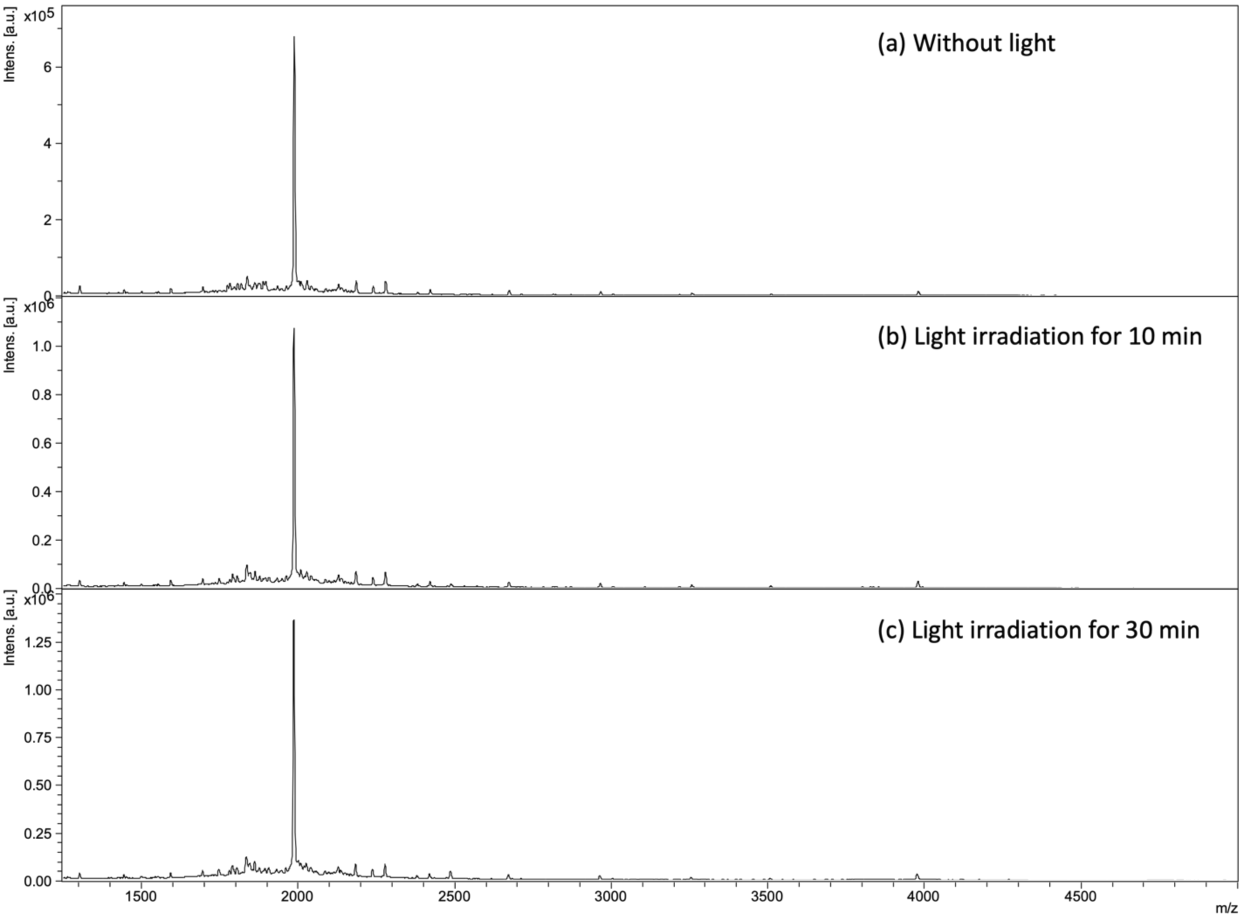

Figure S3-7-15a. MALDI spectra of reaction mixture from PNA X15 and 9 PNA-Ys (a) without light (b) under light irradiation for 10 min or (c) 30 min.

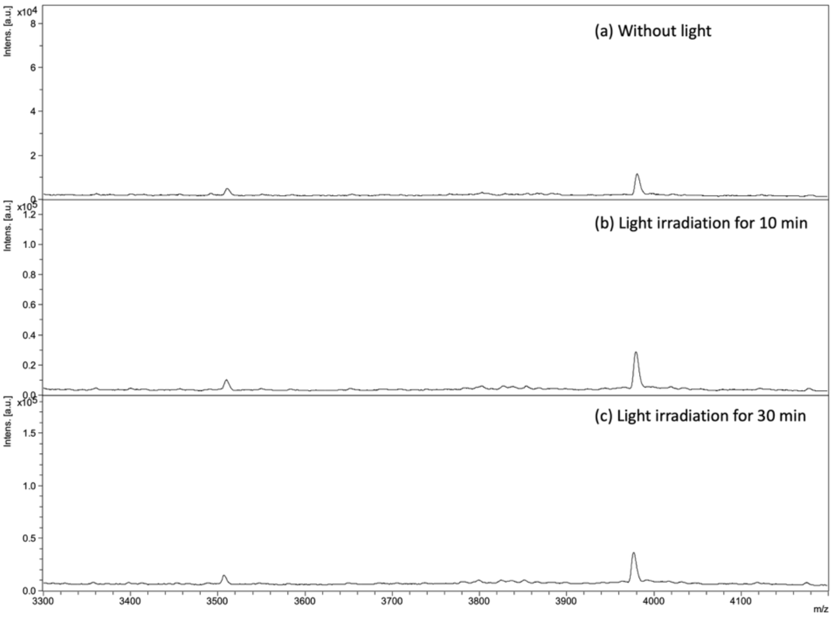

Figure S3-7-15b. Zoomed spectra of Figure S3-7-15a.

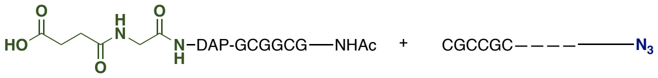

X16

Y1–9

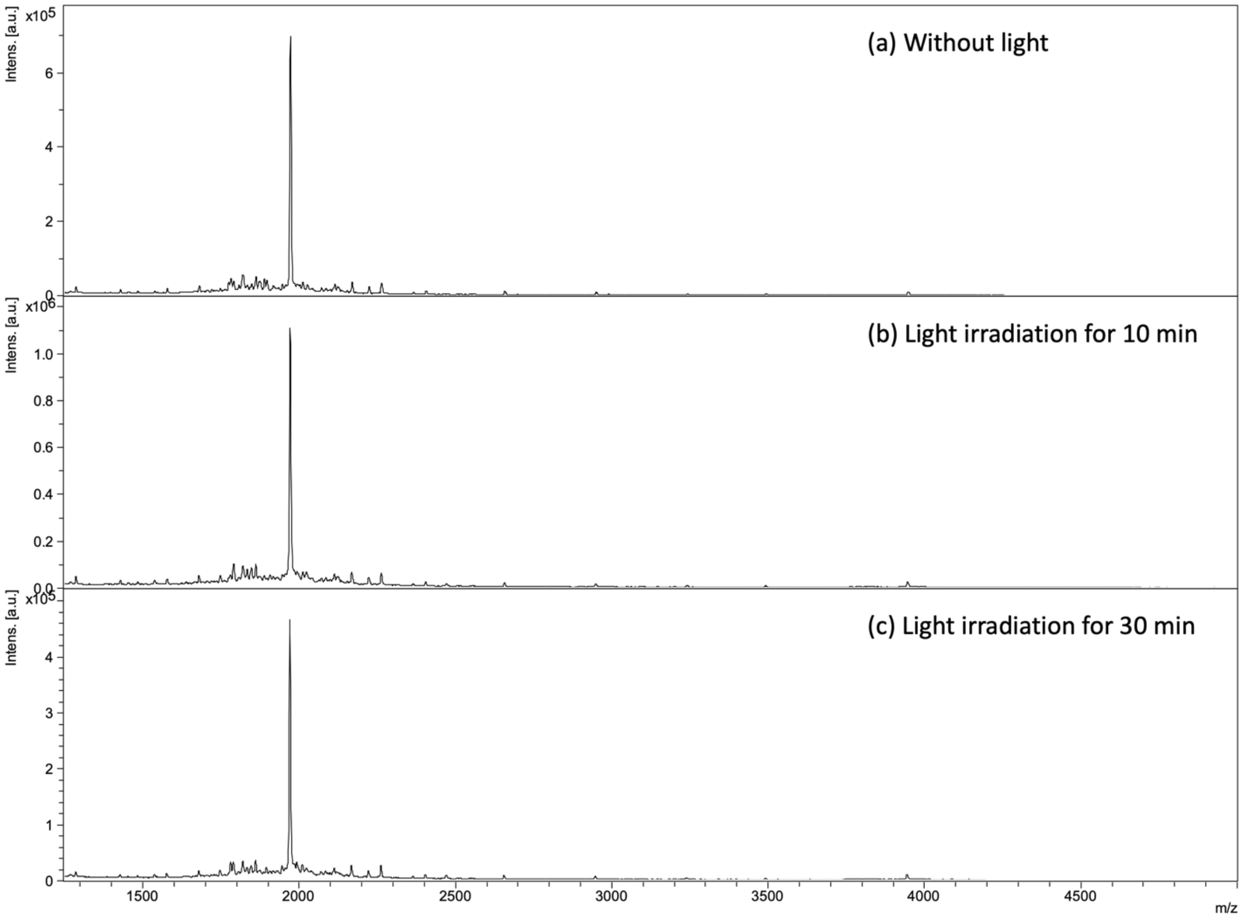

Figure S3-7-16a. MALDI spectra of reaction mixture from PNA X16 and 9 PNA-Ys (a) without light (b) under light irradiation for 10 min or (c) 30 min.

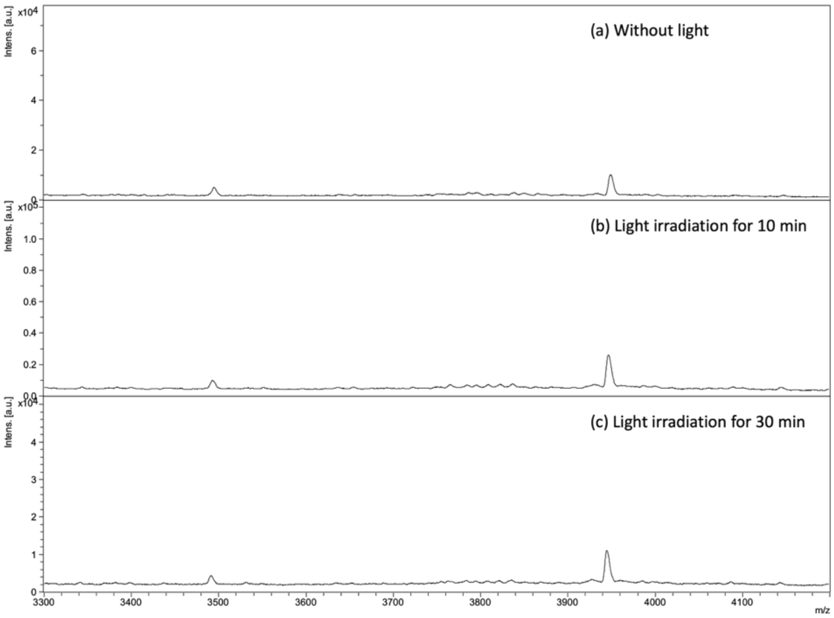

Figure S3-7-16b. Zoomed spectra of Figure S3-7-16a.

2.3.3.4. Round 3 of OPMSS under photocatalytic conditions

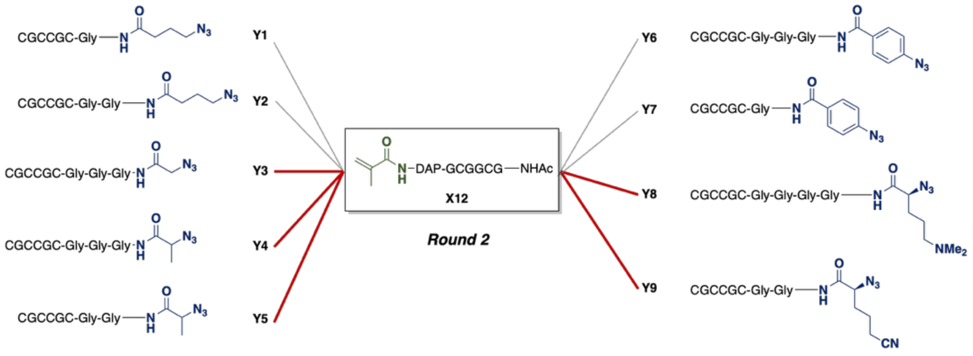

Table S3-14. Reaction condition for one to one Photoredox reaction

Name		Volume / μL	Final concentration	Note
400 μM PNA **X12** aq.		1.5	0.30 mM	1.0 equiv.
400 μM PNA **Y***1 aq. (one PNA)		1.5	0.30 mM	1.0 equiv.
20 mM $[Ir(dtbbpy)(ppy)_2]PF_6$ in DMSO*3		1.0	1.0 mM	3.3 equiv. / PNA Y
Photoredox reaction cocktail A or B*2 16 μL	TEOA or TMEDA	—	50 mM	1.7 x 10^2 equiv. / PNA Y
	1.0 M Tris buffer	4.0	200 mM	—
	H_2O	3.0	—	—
	DMSO	9.0	—	—
	Total	20	—	—

*1PNA Y: **Y1**, **Y2**, **Y3**, **Y4**, **Y5**, **Y6**, **Y7**, **Y8**, **Y9** or **Y10**

*2These four components were added as 16 μL of Photoredox reaction cocktail A or B (see Table S3-15 and S3-16) which were prepared prior to use.

*3 For a negative control experiment without catalyst, DMSO (1.0 μL) was used instead of 20 mM $[Ir(dtbbpy)(ppy)_2]PF_6$ in DMSO.

Procedure for Table S3-13

All reagents were mixed. Then, 18 μL of the reaction mixture was irradiated at 455 nm with a collimated LED light 6 cm above the plate. On the other hand, 2 μL of the reaction mixture was incubated in the dark, which was used as a negative control. After being irradiated for 10 min and 30 min, the reaction mixture was analyzed respectively.

Note: DIPEA was not suitable due to low water solubility.

Table S3-15. Photoredox reaction cocktail_A

Reagents	Volume / μL	Final concentration
1.0 M TEOA	10	63 mM
1.0 M Tris buffer	40	0.25 M
H_2O	20	—
DMSO	90	—
Total	160	—

Table S3-16. Photoredox reaction cocktail_B

Reagents	Volume / μL	Final concentration
TMEDA	1.5	63 mM
1.0 M Tris buffer	40	0.25 M
H_2O	30	—
DMSO	88.5	—
Total	160	—

Table S3-17. list of observed peaks and assigned PNA sequences: Photoredox reaction (+ light), 30 minutes

found. m/z Reflection mode*	assigned PNA	assigned PNA sequence (N to C)	Molecular formula	calc. m/z top peak in isotopic distribution $[M+H]^+$	error
3714.81	X12 + Y3	GCGGCG-DAP-N-**C1**-Gly-Gly-Gly-CGCCGC	C143H186N80O43	3713.45	1.36
3729.07	X12 + Y4	GCGGCG-DAP-N-**C1(Me)**-Gly-Gly-Gly-CGCCGC	C144H188N80O43	3728.53	0.54
3671.50	X12 + Y5	GCGGCG-DAP-N-**C1(Me)**-Gly-Gly-CGCCGC	C142H185N79O42	3670.49	1.01
3857.45	X12 + Y8	GCGGCG-DAP-N-**C1(NMe2)**-Gly-Gly-Gly-Gly-CGCCGC	C150H200N82O44	3856.61	0.84
3725.25	X12 + Y9	GCGGCG-DAP-N-**C1(CN)**-Gly-Gly-CGCCGC	C145H188N80O42	3724.53	0.72

Reflector positive ion mode was utilized for Table S3-17.

*See Figure S3-8.

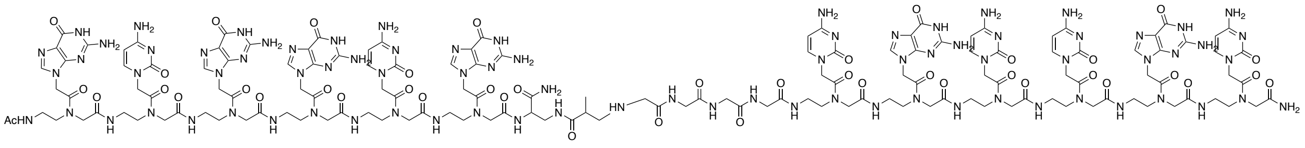

X12 + Y3: GCGGCG-DAP-**N**-Gly-Gly-Gly-Gly-CGCCGC

Chemical Formula: $C_{143}H_{186}N_{80}O_{43}$

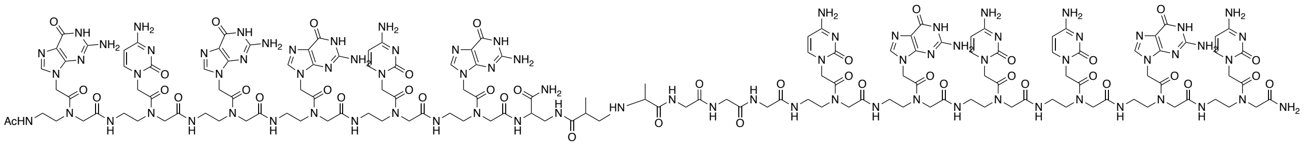

X12 + Y4: GCGGCG-DAP-**N**-C1(Me)-Gly-Gly-Gly-CGCCGC

Chemical Formula: $C_{144}H_{188}N_{80}O_{43}$

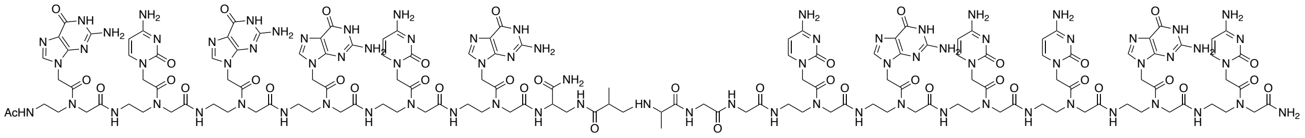

X12 + Y5: GCGGCG-DAP-**N**-C1(Me)-Gly-Gly-CGCCGC

Chemical Formula: $C_{142}H_{185}N_{79}O_{42}$

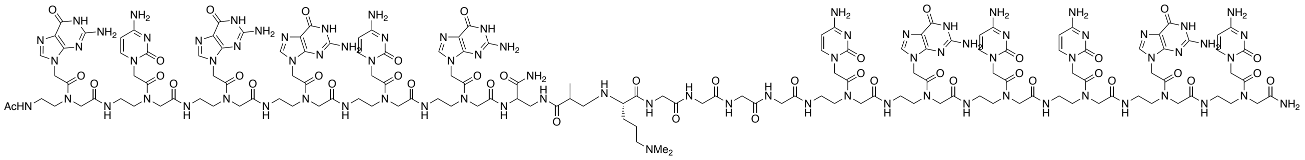

X12 + Y8: GCGGCG-DAP-**N**-C1(NMe_2)-Gly-Gly-Gly-Gly-CGCCGC

Chemical Formula: $C_{150}H_{200}N_{82}O_{44}$

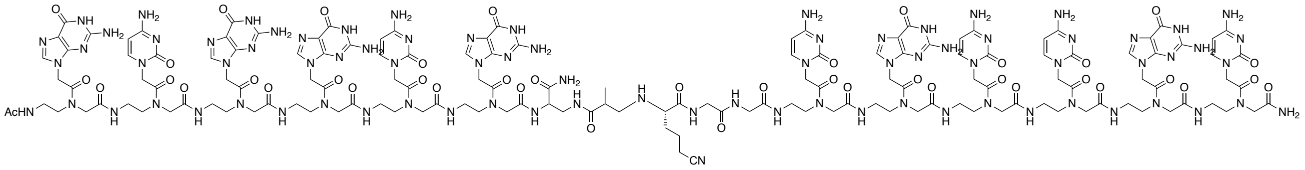

X12 + Y9: GCGGCG-DAP-**N**-C1(CN)-Gly-Gly-CGCCGC

Chemical Formula: $C_{145}H_{188}N_{80}O_{42}$

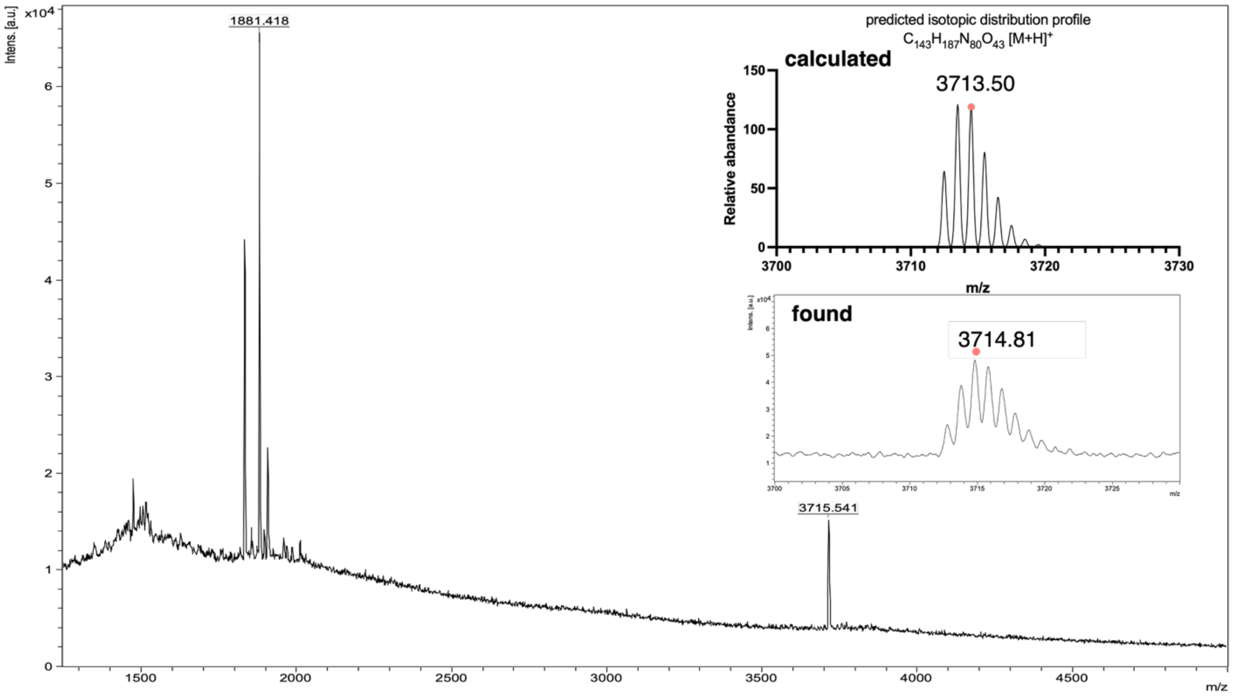

Figure S3-8-1. MALDI spectra of photoredox reaction of X12 and Y3 under light irradiation for 30 minutes.

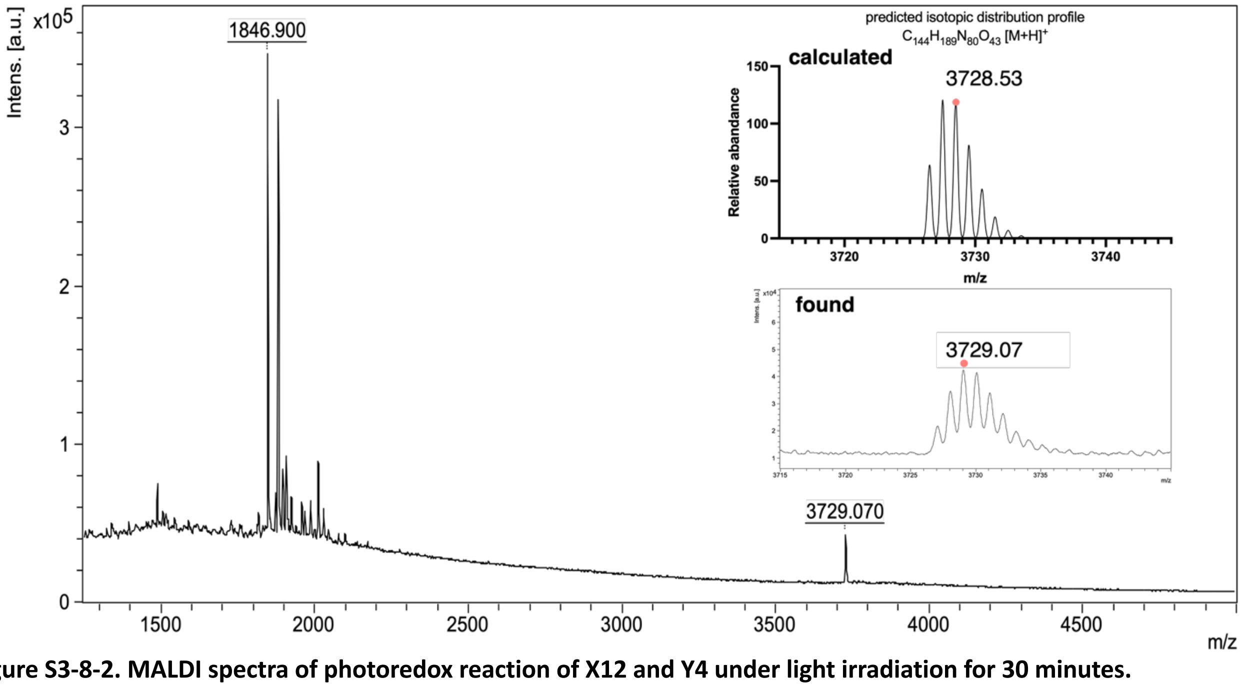

Figure S3-8-2. MALDI spectra of photoredox reaction of X12 and Y4 under light irradiation for 30 minutes.

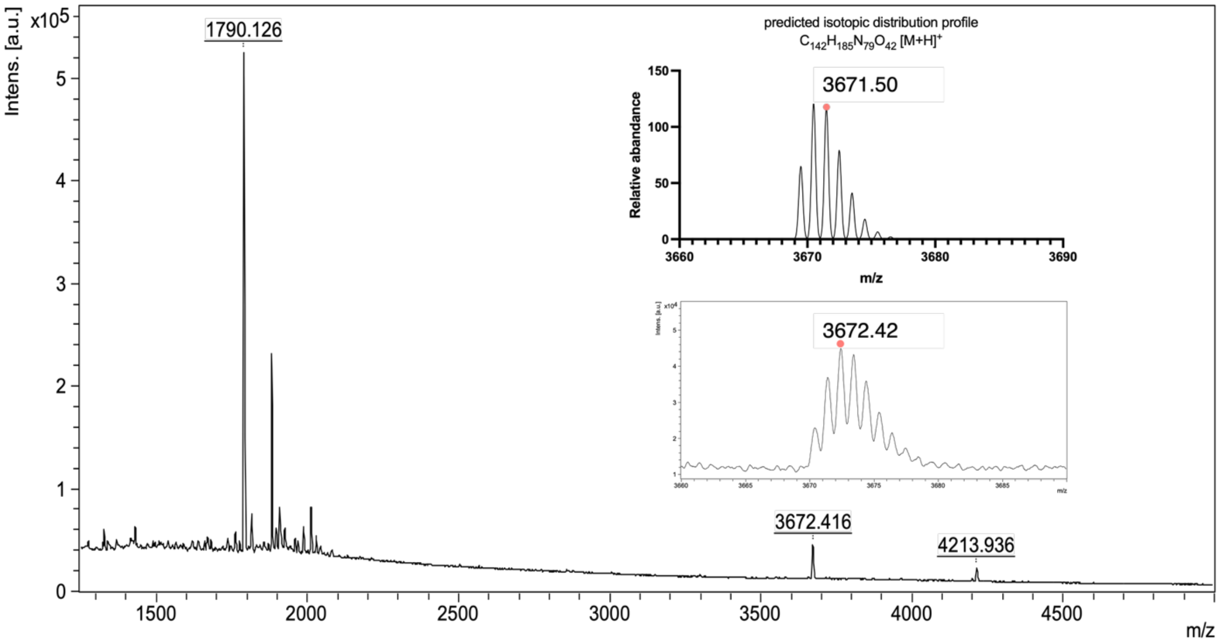

Figure S3-8-3. MALDI spectra of photoredox reaction of X12 and Y5 under light irradiation for 30 minutes.

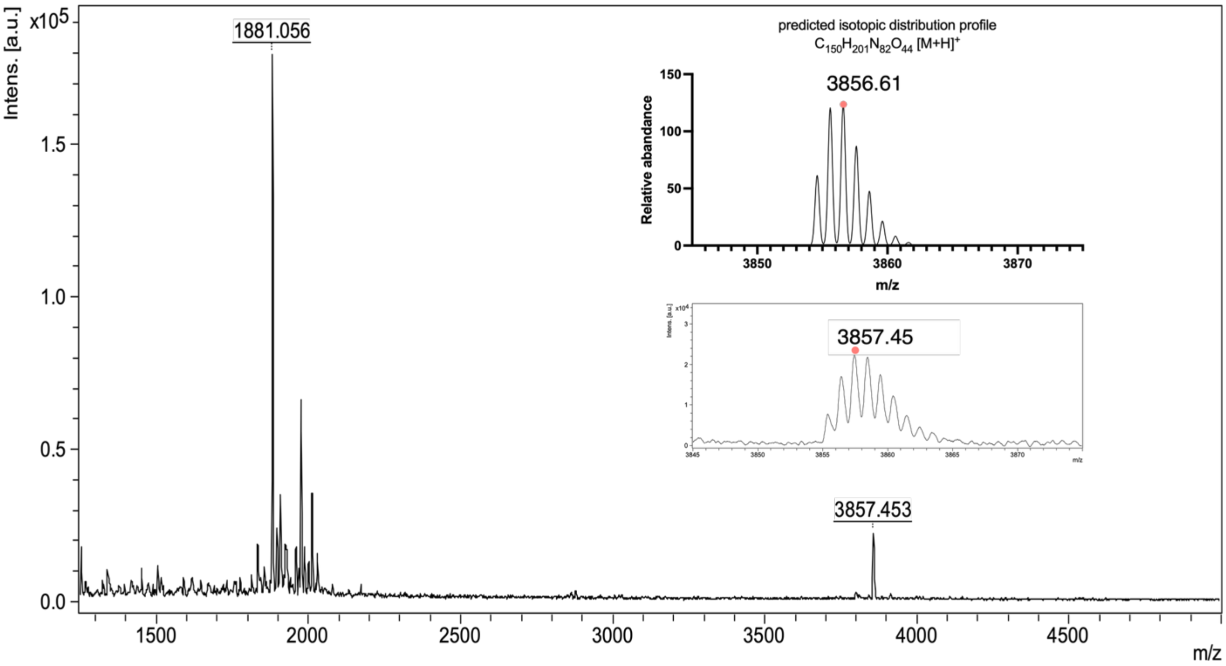

Figure S3-8-4. MALDI spectra of photoredox reaction of X12 and Y8 under light irradiation for 30 minutes.

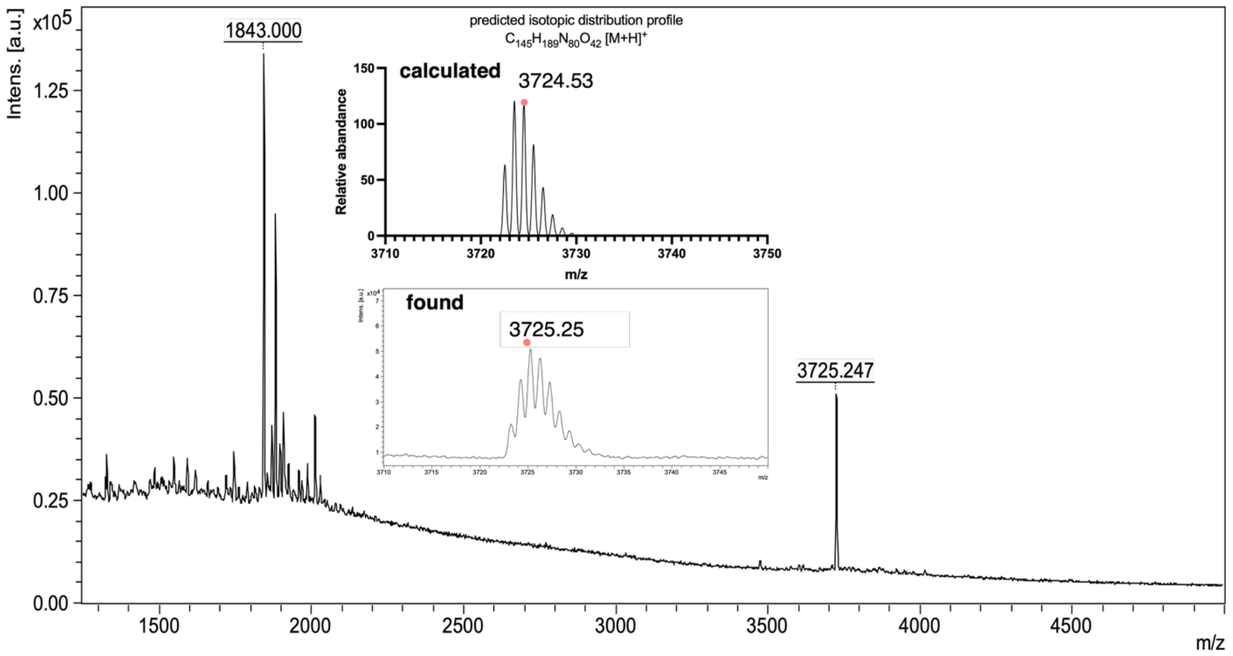

Figure S3-8-5. MALDI spectra of photoredox reaction of X12 and Y9 under light irradiation for 30 minutes.

4. Synthesis of small molecules

4.1. Preparation of fragments for PNA syntheses

To synthesize PNAs, eight carboxylic acids **S1**–**S8** were prepared from commercially available reagents. **S1**–**S6** are known compounds, therefore, they were prepared by following previous report as shown below.
Note: All reactions in batch were carried out under argon atmosphere with dehydrated solvents under anhydrous conditions.

S3 was prepared from ethyl-4-bromobutyrate and NaN_3 in two steps. [2]
S4 (CAS: 18523-48-3) is a commercially available reagent.
S5 was prepared from 2-bromo-2-methylpropanoic acid and NaN_3 in one step.[3]

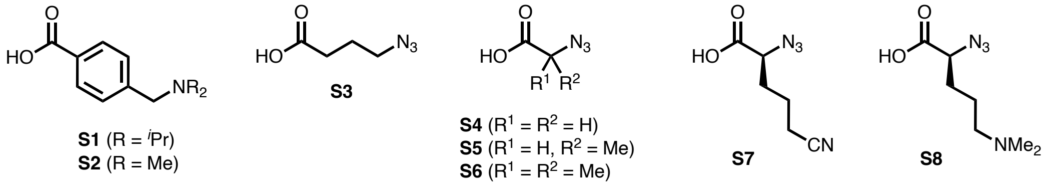

Figure S4_1. List of carboxylic acids prepared for PNA synthesis

Amine S1[4]

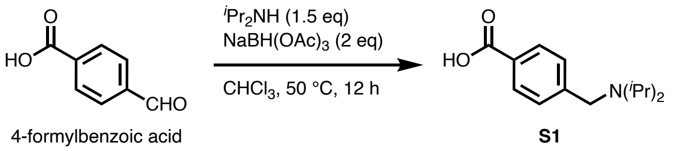

AcOH (3.87 mL, 67.7 mmol) was added dropwise to a solution of $NaBH_4$ (570 mg, 15.0 mmol) in $CHCl_3$ (10 mL) at 0 °C. After the reaction mixture was stirred for 50 min at 0–10 °C, a solution of 4-formylbezoic acid (1.13 g, 7.52 mmol) and ^{i}Pr$_2$NH (1.58 mL, 11.3 mmol) in $CHCl_3$ (10 mL) was added to the reaction mixture. After being stirred for 24 h at 50 °C, the reaction mixture was cooled to room temperature and treated with H_2O (100 mL) and Na_2CO_3 until pH 8. The resulting solution was extracted with $CHCl_3$/MeOH (9:1, 100 mL x 5) and the combined organic extracts were dried over Na_2SO_4, filtered, and concentrated under reduced pressure. The resulting crude was washed with pentane/AcOEt (2/1) to afford **S1** (196 mg, 0.834 mmol, 11%yield) as a colorless solid.

S1: ^{1}H NMR (400 MHz, D_2O) δ 8.09 (d, *J* = 7.7 Hz, 2H), 7.64 (d, *J* = 7.8 Hz, 2H), 4.46 (s, 2H), 3.84 (sep, *J* = 6.7 Hz, 2H), 1.46 (d, *J* = 6.6 Hz, 1H), 1.39 (d, *J* = 6.6 Hz, 13H); ^{13}C{^{1}H} NMR (101 MHz, D_2O) δ 170.2 (C=O), 136.2 (aromatic), 131.2 (aromatic), 130.8 (aromatic), 130.4 (aromatic), 55.0 (CH_2), 49.9 (CH), 18.0 (CH_3), 17.2 (CH_3). LCMS (ESI) calcd for $C_{14}H_{22}NO_2$ $[M+H]^+$: 236.16

Amine S2

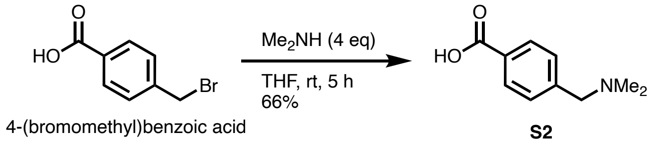

To a solution of 4-(bromomethyl)benzoic acid (263 mg, 1.22 mmol) in THF (4 mL), Me_2NH (33% EtOH solution, 0.87 mL, 4.89 mmol) was added. After being stirred for 5 h, the resulting suspension was filtered. The resulting mother liquid was concentrated under reduced pressure to afford HBr salt of **S2** (231 mg containing 0.27 mmol of $MeNH_3Br$, 0.800 mmol, 66%) as a colorless solid. The solid was washed with MeOH to enhance the purity (HBr salt of **S2** : $MeNH_3Br$ = 100 : 6.5).

S2: ^{1}H NMR (400 MHz, D_2O) δ 7.92 (d, *J* = 8.3 Hz, 2H), 7.53 (d, *J* = 8.2 Hz, 2H), 4.35 (s, 2H), 2.87 (s, 6H); ^{13}C{^{1}H} NMR (101 MHz, D_2O) δ 175.0 (C=O), 138.1 (aromatic), 132.1 (aromatic), 130.8 (aromatic), 129.6 (aromatic), 60.8 (CH_2), 42.3 (CH_3); LCMS (ESI) calcd for $C_{10}H_{14}NO_2$ $[M+H]^+$: 180.10

Scheme S1. Synthesis of S7 and S8.

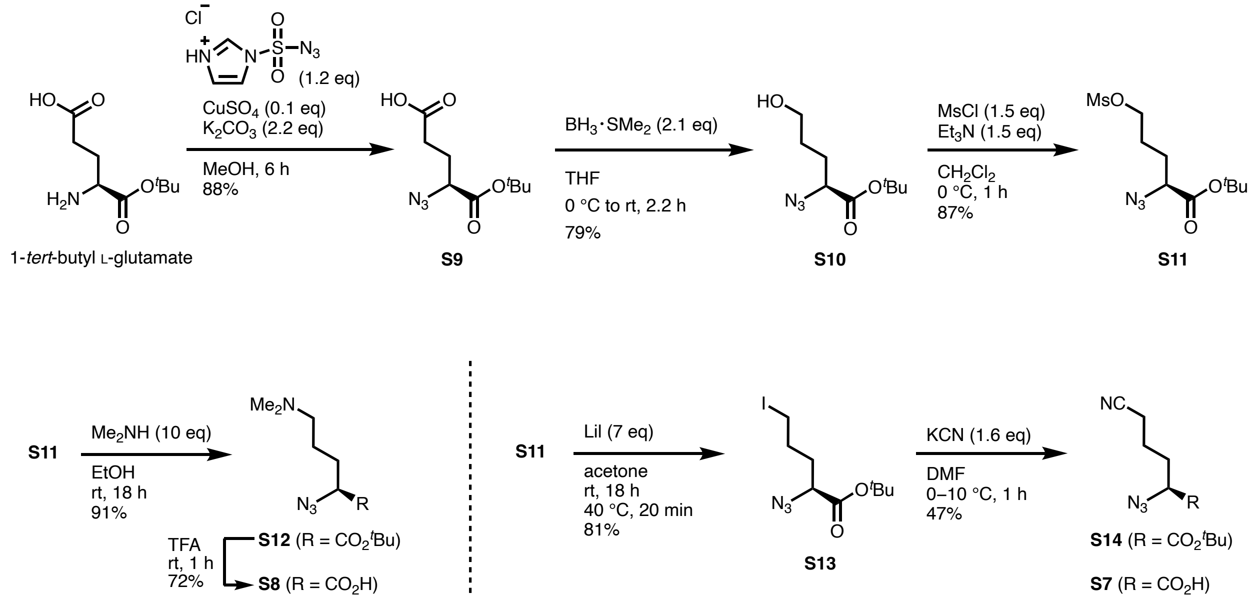

Azide S9

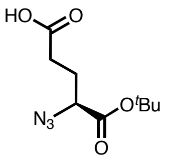

S9

To a solution of L-glutamic acid *t*-butyl ester (1.24 g, 6.10 mmol), $CuSO_4$-H_2O (152 mmol, 0.610 mmol), and K_2CO_3 (1.85 g, 13.4 mmol) in MeOH (30 mL) was added imidazole-1-sulfonyl azide hydrochloride (1.26 g, 7.32 mmol) [5]. After being stirred for 6 h at room temperature, the mixture was quenched with H_2O (50 mL) and 2 M aqueous HCl (17 mL). The mixture was extracted with AcOEt and the combined organic extracts were washed with brine, dried over Na_2SO_4, filtered, and concentrated under reduced pressure. The residue was purified by silica gel column chromatography (pentane/AcOEt = 4/1 to 2/1) to give azide **S9** (1.23 g, 5.35 mmol, 88%yield) as a colorless oil.

S9: ^{1}H NMR (400 MHz, $CDCl_3$) δ 3.87 (1H, dd, *J* = 8.7, 5.1 Hz, CH), 2.54-2.49 (2H, m, -CH_2-CO_2H), 2.19-2.09 (1H, m, -CH_2-), 2.05-1.93 (1H, m, -CH_2-), 1.50 (9H, s, *t*Bu); ^{13}C{^{1}H} NMR (101 MHz, $CDCl_3$) δ 178.1 (CO_2H), 169.0 (CO_2tBu), 83.5 (C-O), 61.6 (CH), 30.0 (CH_2), 28.2 (CH_3), 26.3 (CH_2); HRMS (EI) calcd for $C_9H_{15}N_3NaO_4$ ([M+Na]$^+$): 252.0960, found 252.0971

Alcohol S10

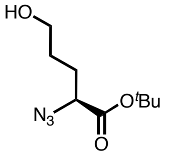

S10

To a solution of carboxylic acid **S9** (1.23 g, 5.35 mmol) in THF (18 mL) was added BH_3-SMe_2 (2.0 M THF solution, 5.5 mL, 11 mmol) at 0 °C. After being stirred for 5 min at 0 °C, the mixture was warmed up to room temperature and stirred for 2.1 h. The reaction mixture was quenched with H_2O (22 mL) and K_2CO_3 (2.2 g). The resulting mixture was extracted with AcOEt and the combined organic extracts were washed with brine, dried over Na_2SO_4, filtered, and concentrated under reduced pressure. The residue was purified by silica gel column chromatography (pentane/AcOEt = 4/1 to 3/1) to give alcohol **S10** (911 mg, 4.24 mmol, 79%yield) as a colorless oil.

S10: ^{1}H NMR (400 MHz, $CDCl_3$) δ 3.74 (1H, dd, *J* = 8.2, 5.3 Hz, *CH*), 3.68 (2H, t, *J* = 6.2 Hz, -CH_2OH), 1.98-1.75 (2H, m, -CH_2-), 1.74-1.60 (2H, m, -CH_2-), 2.05-1.93 (1H, m, -CH_2-), 1.50 (9H, s, *t*Bu); ^{13}C{^{1}H} NMR (101 MHz, $CDCl_3$) δ 169.7 (C=O), 83.1 (C-O), 62.5 (O-CH_2 or CH), 62.2 (O-CH_2 or CH), 28.9 (CH_2), 28.1 (CH_2), 28.0 (CH_3); HRMS (EI) calcd for $C_9H_{17}N_3NaO_3$ [M+Na]$^+$ 238.1168, Found 238.1149

mesylate S11

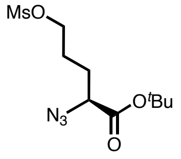

S11

To a solution of alcohol **S10** (621 g, 2.88 mmol) in CH_2Cl_2 (14 mL) were added Et_3N (0.61 mL, 4.4 mmol) and MsCl (0.33 mL, 4.3 mmol) at 0 °C. After being stirred for 1 h at 0 °C, the mixture was quenched with H_2O. The resulting mixture was extracted with AcOEt and the combined organic extracts were washed with brine, dried over Na_2SO_4, filtered, and concentrated under reduced pressure. The residue was purified by silica gel column chromatography (pentane/AcOEt = 9/1 to 4/1) to give mesylate **S11** (738 mg, 2.51 mmol, 87%yield) as a colorless oil.

S11: ^{1}H NMR (400 MHz, $CDCl_3$) δ 4.26 (1H, t, *J* = 5.78 Hz, CH_2-OMs), 3.79 (1H, dd, *J* = 7.5, 4.9 Hz, *CH*), 3.02 (3H, s, CH_3-OSO_2-), 1.97-1.76 (4H, m, -CH_2-x2), 1.51 (9H, s, *t*Bu); ^{13}C{^{1}H} NMR (101 MHz, $CDCl_3$) δ 169.1 (C=O), 83.5 (C-O), 69.0 (O-CH_2), 62.0 (CH), 37.7 (CH_3-OSO_2), 28.2 (CH_3 of t-Bu), 27.6 (CH_2), 25.7 (CH_2); HRMS (EI) calcd for $C_{10}H_{19}N_3NaO_5S$ [M+Na]$^+$ 316.0943, Found 316.0937

amine S12

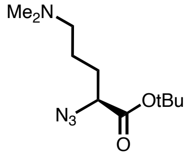

S12

Mesylate **S11** (45.4 mg, 0.154 mmol) were mixed with Me_2NH (EtOH solution, 33%, 0.275 mL, 1.54 mmol). After being stirred for 18 h at room temperature, the mixture was concentrated under reduced pressure. The residue was purified by silica gel column chromatography (pentane/AcOEt = 2/1 to $CHCl_3$/MeOH/Et_3N 95/5/0.1) to give amine **S12** (34.0 mg, 0.140 mmol, 91%yield) as a colorless oil.

S12: ^{1}H NMR (400 MHz, $CDCl_3$) δ 3.71 (1H, dd, J = 8.2, 5.5 Hz, *CH*), 2.31 (2H, t, J = 7.2 Hz, -CH_2-NMe$_2$), 2.23 (6H, s, CH_3-N), 1.90-1.70 (2H, m, -CH_2-), 1.63-1.53 (2H, m, -CH_2-), 1.50 (9H, s, *t*Bu); ^{13}C{^{1}H} NMR (101 MHz, $CDCl_3$) δ 169.8 (C=O), 82.9 (C-O), 62.6 (CH or *N*-CH_2), 59.1 (CH or *N*-CH_2), 45.5 (N(CH_3)$_2$), 29.4 (CH_2), 28.2 (CH_3), 23.9 (CH_2); HRMS (EI) calcd for $C_{11}H_{23}N_4O_2$ [M+H]$^+$ 243.1821, Found 243.1862

Carboxylic acid S8

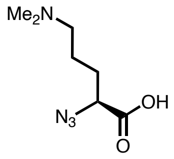

S8

t-butyl ester **S12** (15.2 mg, 62.7 μmol) and TFA (129 μL, 1.68 mmol) were mixed and the resulting solution was incubated on a shaker for 1 h at room temperature. The reaction mixture was concentrated under reduced pressure. An azeotrope of the resulting residue with toluene gave carboxylic acid **S8** (8.4 mg, 45 mmol, 72%yield) as a colorless oil, which was used in the next PNA synthesis without further purification.

S8: ^{1}H NMR (400 MHz, CD_3OD) δ 4.15 (1H, dd, J = 4.3, 8.0 Hz, *CH*), 3.23-3.08 (2H, m, -CH_2-NMe$_2$), 2.88 (6H, s, CH_3-N), 1.99-1.70 (4H, m, -CH_2-) LCMS (ESI) calcd for $C_7H_{15}N_4O_2$ [M+H]$^+$:187.12

iodide S13

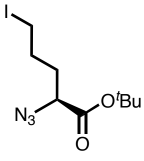

S13

To a solution of mesylate **S11** (228 mg, 0.778 mmol) in acetone (3.9 mL) was added lithium iodide (312 mg, 2.33 mmol). After being stirred for 12 h at room temperature, the reaction mixture was heated at 40 °C and stirred for 20 min, then LiI (624 mg, 4.66 mmol). After being stirred for 6 h at room temperature, the mixture was quenched with H_2O. The resulting mixture was extracted with AcOEt/pentane (1/2) and the combined organic extracts were washed with brine, dried over Na_2SO_4, filtered, and concentrated under reduced pressure. The residue was purified by silica gel column chromatography (pentane/AcOEt = 10/0 to 10/1) to give iodide **S13** (204 mg, 0.628 mmol, 81%yield) as a colorless oil.

S13: ^{1}H NMR (400 MHz, $CDCl_3$) δ 3.80-3.68 (1H, m *CH*), 3.26-3.12 (2H, m, CH_2-I), 2.01-1.71 (4H, m, CH_2), 1.51 (9H, s, ^{t}Bu); ^{13}C{^{1}H} NMR (101 MHz, $CDCl_3$) δ 169.3 (C=O), 83.3 (C-O), 61.7 (CH), 32.3 (CH_2-I), 29.6 (CH_2), 28.2 (CH_3 of *t*Bu), 5.25 (CH_2)

cyanide S14

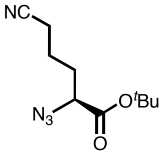

S14

To a solution of iodide **S13** (85.0 mg, 0.261 mmol) in DMF (1.3 mL) was added KCN (27.1 mg, 0.416 mmol) at 0 °C. After being stirred for 20 min at 0-10 °C and for 1 h at room temperature, the reaction mixture was quenched with H_2O at 0 °C. The resulting mixture was extracted with Et_2O and the combined organic extracts were washed with sat. $NaHCO_3$ and brine, dried over Na_2SO_4, filtered, and concentrated under reduced pressure. The residue was purified by silica gel column chromatography (pentane/Et_2O = 100/0 to 30/1 to 10/1 to 4/1) to give cyanide **S14** (40.3 mg, 0.124 mmol, 48%yield) and cyanide X (27.6 mg, 0.123 mmol, 47%yield) as a colorless oil.

S14: ^{1}H NMR (400 MHz, $CDCl_3$) δ 3.80 (1H, dd, J = 7.8, 5.0 Hz, *CH*), 2.40 (2H, t, J = 6.9 Hz, CH_2-CN), 2.00-1.68 (4H, m, CH_2), 1.50 (9H, s, *t*Bu); ^{13}C{^{1}H} NMR (101 MHz, $CDCl_3$) δ 168.9 (C=O), 119.1 (CN), 83.5 (C-O), 61.8 (CH), 30.3 (CH_2-*CN*), 28.1 (CH_3 of *t*Bu), 21.9 (CH_2), 17.0 (CH_2); HRMS (TOF MS ES+) calcd for $C_{10}H_{17}N_4O_2$ [M+H]$^+$ 225.1352, Found 225.1336

Carboxylic acid S7

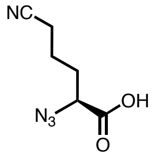

S7

To a solution of *t*-butyl ester **S14** (29.8 mg, 0.132 mmol) in CH_2Cl_2 (1 mL) TFA (0.3 mL, 4 mmol) was added at 0 °C. After being stirred for 50 min at room temperature, the reaction mixture was concentrated under reduced pressure. The residue was purified by silica gel column chromatography (pentane/AcOEt = 2/1 to 1/1 to 0/1) to give carboxylic acid **S7** (12.1 mg, 71.9 μmol, 54%yield) as a colorless oil, which was used in the next PNA synthesis without further purification.

S7: ^{1}H NMR (400 MHz, CD_3OD) δ 4.13-4.07 (1H, m, *CH*), 2.51 (2H, t, J = 6.9 Hz, CH_2-CN), 2.00-1.70 (4H, m, CH_2).

4.2. Chemistry parts

azide S15

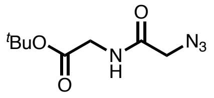

To a solution of glycine *t*-butyl ester hydrochloride (247 mg, 1.47 mmol) in CH_2Cl_2 (15 mL) were added chloroacetic acid (209 mg, 2.21 mmol), DMAP (8.9 mg, 0.0735 mmol), Et_3N (0.611 mL, 4.41 mmol), and EDCI (422.6 mg, 2.21 mmol) at 0 °C. After being stirred for 13 h at room temperature, the reaction mixture was quenched with H2O at 0 °C. The resulting mixture was extracted with CH_2Cl_2 and the combined organic extracts were washed with 1 M HCl aq., sat. $NaHCO_3$, and brine. The resulting solution was dried over Na_2SO_4, filtered, and concentrated under reduced pressure. The resulting crude amide (196 mg, 0.948 mmol, colorless solid) was used for the next reaction without further purification. To a solution of α-chloro amide (196 mg, 0.948 mmol) in DMF (9.5 mL) was added NaN_3 (123 mg, 1.89 mmol). After being stirred for 2 h, the reaction mixture was cooled to room temperature and Et_2O (20 mL) and H_2O (20 mL) were added. The resulting solutions were separated, and the aqueous layer was extracted with Et_2O (20 mLx2). The combined organic extracts were washed with brine, dried over Na_2SO_4, filtered, and concentrated under reduced pressure. The residue was recrystallized with pentane/AcOEt (10/1) to afford azide **S15** (133 mg, 0.621 mmol, 66% yield) as a colorless plate.
^{1}H NMR spectrum of azide **S15** was identical with reported data[6].

azide S16

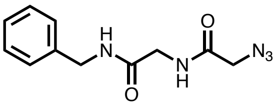

To a solution of *t*-butyl ester **S15** (239 mg, 1.11 mmol) in CH_2Cl_2 (7 mL) was added TFA (2 mL). After being stirred for 2 h at room temperature, the reaction mixture was concentrated under reduced pressure. The residue was used for the next reaction without purification. To a solution of the crude carboxylic acid (1.11 mmol) in CH_2Cl_2 (7 mL) were added $BnNH_2$ (0.36 mL, 3.33 mmol), Et_3N (0.46 mL, 3.3 mmol), DMAP (6.8 mg, 0.055 mmol), and EDCI-HCl (319 mg, 1.66 mmol) at 0 °C. After the reaction mixture was stirred for 2 h at room temperature, $BnNH_2$ (0.36 mL, 3.33 mmol), Et_3N (0.46 mL, 3.3 mmol), and EDCI-HCl (319 mg, 1.66 mmol) were added at 0 °C. After being stirred for 15 h at room temperature, the reaction mixture was quenched with sat. NH_4Cl aq at 0 °C. The resulting mixture was extracted with AcOEt (30 mLx3) and the combined organic extracts were washed with brine, dried over Na_2SO_4, filtered, and concentrated under reduced pressure. The residue was purified by silica gel column chromatography (pentane/AcOEt = 3/1 to 1/1 to 0/1) to give benzylamide **S16** (114 mg, 0.459 mmol, 41%yield) as a colorless solid.

S16: ^{1}H NMR (400 MHz, $CDCl_3$) δ 7.41 – 7.25 (m, 5H, aromatic), 7.03 (brs, 1H, *NH*), 6.19 (brs, 1H, *NH*), 4.47 (d, *J* = 5.8 Hz, 2H, *CH_2*-Ph), 4.01 (s, 2H, *CH_2*-N_3), 3.98 (d, *J* = 5.3 Hz, 2H, *CH_2*-); $^{13}C\{^1H\}$ NMR (101 MHz, $CDCl_3$) δ 167.9 (C=O), 167.4 (C=O), 137.6 (aromatic), 129.0 (aromatic), 128.0 (aromatic), 127.9 (aromatic), 52.6 (CH_2), 43.9 (CH_2), 43.1 (CH_2); HRMS (ESI) calcd for $C_{11}H_{13}N_5O_2Na$ $[M]^+$ 270.0961, Found 270.0979

5. Experimental data using small molecules

amide S17

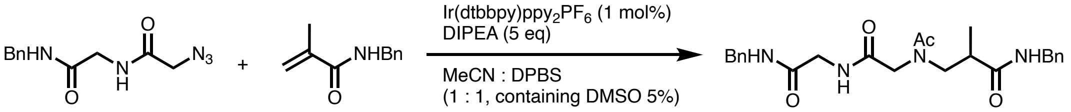

S16 S17

Scheme S2

Azide **S16** (0.50 M DMSO solution, 50 μL, 25 μmol), *N*-benzylmethacrylamide* (0.50 M DMSO solution, 500 μL, 250 μmol), Ir(dtbbpy)ppy$_2$PF$_6$ (20 mM DMSO solution, 12.5 μL, 0.25 μmol), MeCN (5.41 mL), and DPBS (-) (6 mL) were mixed. The resulting solution (1.5 mL) was moved to 8 Eppendorf tubes (2 mL) and DIPEA (2.75 μL x 8, 12.8 μmol x 8) was added to each tube. The reaction mixtures were irradiated at 455 nm for 2 h. This experiment was done three times. The resulting reaction mixture was combined and concentrated under reduced pressure. The resulting crude was purified by silica gel column chromatography (pentane/AcOEt = 1/1 to CHCl$_3$/MeOH = 9/1), PTLC (CHCl$_3$/MeOH = 9/1, ten times), and HPLC (MeCN/0.01% aqueous TFA solution = 5/96 to 90/10) to afford **S17** (1 mg, 2 μmol, < 3% yield).

* *N*-benzylmethacrylamide was prepared by following a reported procedure.[7]

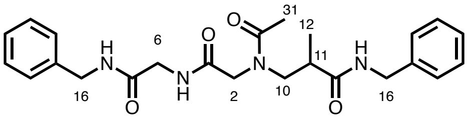

S17

S17: ^{1}H NMR (400 MHz, CDCl$_3$): δ 8.02 (1H, brs, *NH*), 7.38 – 7.17 (10H, m, *aromatic*), 6.71 (2H, brs, *NH*), 4.42 (2H, d, *J* = 5.8 Hz, *H16*), 4.40–4.22 (3H, m, *H16* and *H2*), 4.06 – 3.86 (2H, m, *H6* and *H10*), 3.40 – 3.28 (2H, m, *H2* and *H6*), 2.99 (1H, dd, *J* = 3.1, 14.8 Hz, *H10*), 2.78 – 2.65 (1H, m, *H11*), 2.04 – 1.93 (3H, s, *H31*), 1.19 – 1.09 (3H, d, *J* = 6.9 Hz, *H12*). ^{13}C{^{1}H} NMR (126 MHz, CDCl$_3$) δ 174.0 (C=O), 172.9 (C=O), 170.5 (C=O), 168.7 (C=O), 138.6 (aromatic, C), 138.0 (aromatic, C), 128.8 (aromatic, C-H), 128.8 (aromatic, C-H), 128.2 (aromatic, C-H), 128.1 (aromatic, C-H), 127.7 (aromatic C-H), 127.5 (aromatic, C-H), 56.1 (-NAc-*CH$_2$*-CH), 53.3 (CO-*CH$_2$*-NAc), 43.8 (Ph-*CH$_2$*, two carbon are overlapping), 43.1 (CO-*CH$_2$*-NHCO-), 40.4 (CH), 21.0 (CO-*CH$_3$*), 15.5 (CH-*CH$_3$*).; HRMS (ESI) calcd for $C_{24}H_{31}N_4O_4$ $[M+H]^+$ 439.2345, Found 439.2267.

Azide 3

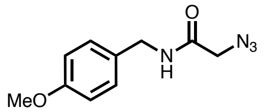

3

To a solution of benzylamine (2.00 g, 14.6 mmol) in CH_2Cl_2 (32 mL) was added triethylamine (2.40 mL, 17.8 mmol) at 0°C. After the reaction mixture was stirred at 0°C for 5 minutes, chloroacetyl chloride (1.65 g, 14.6 mmol) was added to the reaction mixture. After stirring at room temperature for 2 hours, the reaction mixture was added 10% HCl aq. (30 mL). Extract with AcOEt (30 mL×2). After separate, organic layer was dried (Na_2SO_4) and concentrated under reduced pressure. The residue was dissolved in acetone/water = 2/1 (51 mL). This solution was added NaN_3 (2.61 g, 40.2 mmol, 2.75 equiv.) and warmed at 60 °C. After being stirred overnight, the reaction mixture was added Brine (30 mL) and AcOEt (50 mL). After separate, organic layer was dried (Na_2SO_4) and concentrated under reduced pressure. The residue purified by flash column chromatography (SiO_2, 1/1 hexanes/EtOAc) to afford **3** (2.79 g, 87%) as brown solids.

^{1}H NMR (600 MHz, CDCl$_3$): δ 7.21 (2H, d, *J* = 8.7 Hz), 6.88 (2H, d, *J* = 8.7 Hz), 6.52 (1H, brs), 4.40 (2H, d, *J* = 5.8 Hz), 4.03 (2H, s), 3.80 (3H, s);^{13}C{^{1}H} NMR (151 MHz, CDCl$_3$): δ 166.4, 159.4, 129.6, 129.5, 114.4, 55.4, 52.9, 43.1; HRMS (ESI): calcd for $C_{10}H_{12}N_4NaO_2$ $[M+Na]^+$ 243.0852, found 243.0854.

Scheme S3. Unsuccessful results.

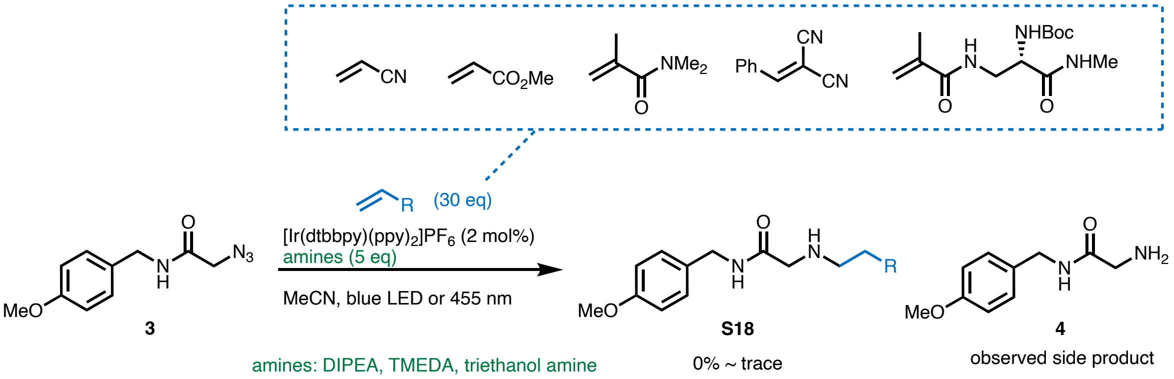

Procedure for Table 1

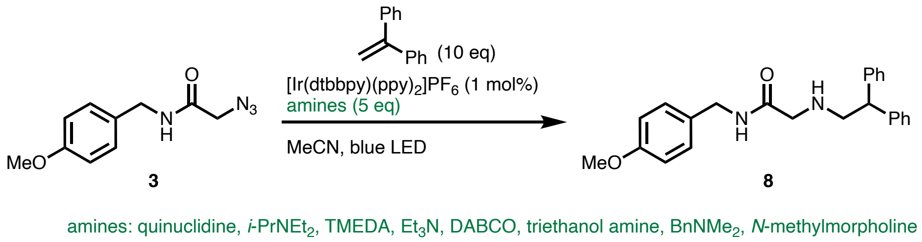

After Azide **3** (0.20 M MeCN solution, 250 µL, 50 µmol), diphenylethene (90.1 mg, 500 µmol), MeCN (1 mL), amines (neat or 1.0 N H_2O solution, 250 µmol) were mixed, $Ir(dtbbpy)ppy_2PF_6$ (20 mM DMSO solution, 25 µL, 0.50 µmol) was added to the reaction mixture. The reaction mixtures were irradiated with blue LED for 3 h at room temperature.
After acetoanilide (50 mM MeCN solution, 1.0 mL, 50 µmol) was added to the reaction mixture, the resulting solution was analyzed by LCMS.

Analytical condition

LC-MS spectra were recorded on LCMS8030 and Labsolutions (condition for elution gradient: 0 min, 0–2 min, A:B = 90:10; 30.1–35 min, A:B = 10:90; solution A: 0.1% aqueous TFA solution; solution B, HPLC grade acetonitrile; flow rate: 0.50 mL/min) with a SIMADZU LCMS8030 and Labsolutions using GL Sciences Inertsustain C18 column (3 µm, 4.6 × 150 mm) operated in positive mode. Detection wavelength: 275 nm

Amine 8

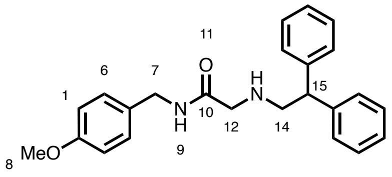

8: ^1H NMR (600 MHz, $CDCl_3$) δ 7.35 – 7.23 (m, 6H, *H9*, *Ph*), 7.23 – 7.16 (m, 5H, *Ph*), 7.14 (d, *J* = 8.7 Hz, 2H, *H6*), 6.85 (d, *J* = 8.7 Hz, 2H, *H1*), 4.29 (d, *J* = 6.0 Hz, 2H, *H7*), 4.05 (t, *J* = 7.7 Hz, 1H, *H15*), 3.79 (s, 3H, -*OMe*), 3.31 (s, 2H, *H12*), 3.21 (d, *J* = 7.7 Hz, 2H, *H14*). ^{13}C NMR (151 MHz, $CDCl_3$) δ 171.4 (C=O), 159.0 (aromatic, C), 142.5 (aromatic, C), 130.7 (aromatic, C), 129.2 (aromatic, CH), 128.8 (aromatic, CH), 128.0 (aromatic, CH), 126.8 (aromatic, CH), 114.1 (aromatic, CH), 55.4 (OMe), 54.8 (*C7*), 52.4 (*C12*), 51.5 (*C15*), 42.4 (*C14*). HRMS (ESI) calcd for $C_{24}H_{27}N_2O_2$ $[M+H]^+$ 375.2067, Found 375.2076.

6. References

[1] X. Peng, H. Li, M. Seidman, *European J Org Chem* **2010**, *2010*, 4194-4197.

[2] C. A. DeForest, K. S. Anseth, *Nat Chem* **2011**, *3*, 925-931.

[3] H. Y. Jo, J. M. Lee, E. Pietrasiak, E. Lee, Y. H. Rhee, J. Park, *J Org Chem* **2021**, *86*, 17409-17417.

[4] E. V. Koroleva, A. P. Kadutskii, A. V. Farina, J. V. Ignatovich, A. L. Ermolinskaya, K. N. Gusak, E. N. Kalinichenko, *Tetrahedron Letters* **2012**, *53*, 5056-5058.

[5] E. D. Goddard-Borger, R. V. Stick, *Org Lett* **2007**, *9*, 3797-3800.

[6] S. Fatemi, N. Gernigon, D. G. Hall, *Green Chemistry* **2015**, *17*, 4016-4028.

[7] S. Sarkar, A. Banerjee, J. A. Shah, U. Mukherjee, N. C. Frederiks, C. J. Johnson, M.-Y. Ngai, *Journal of the American Chemical Society* **2022**, *144*, 20884-20894.

7. NMR spectra

^{1}H-NMR of **S1**

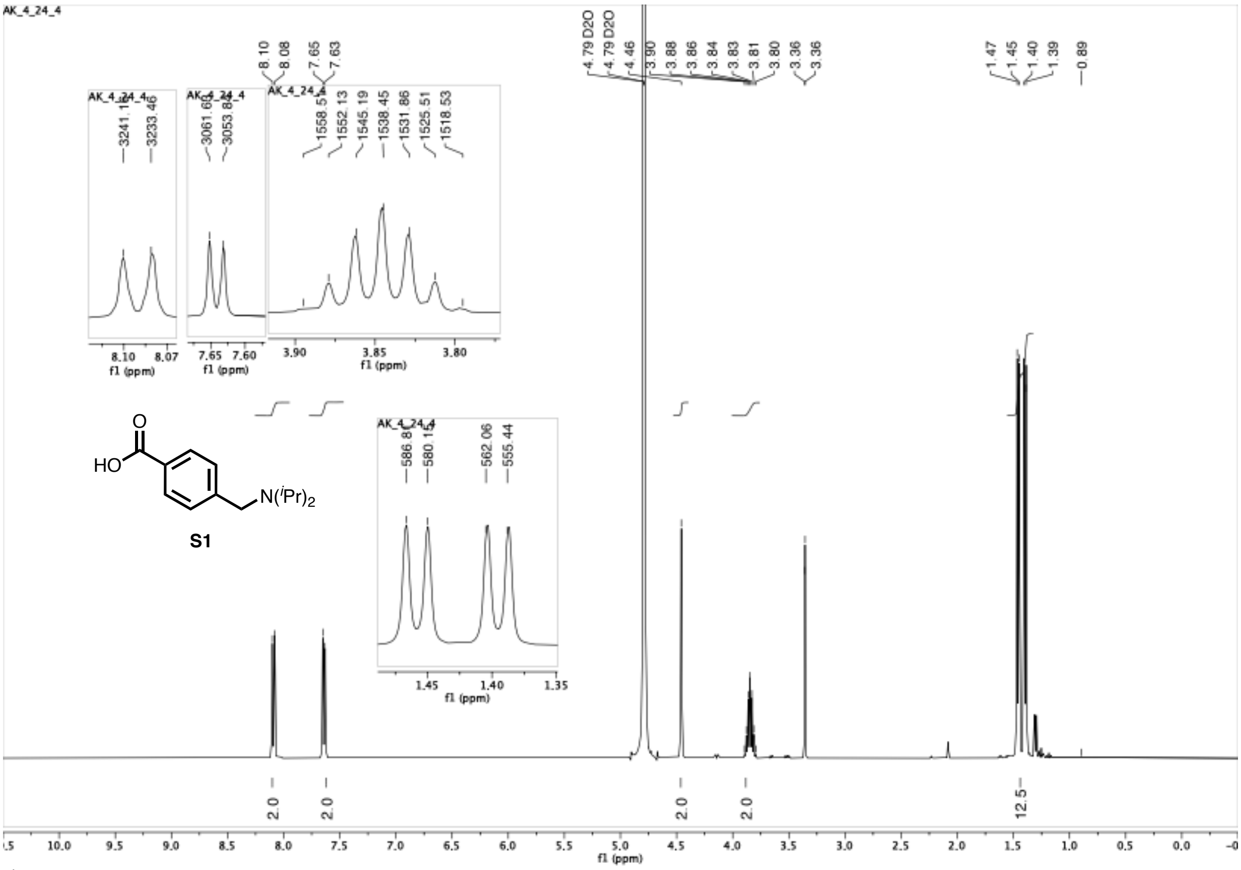

^{13}C{^{1}H}-NMR of **S1**

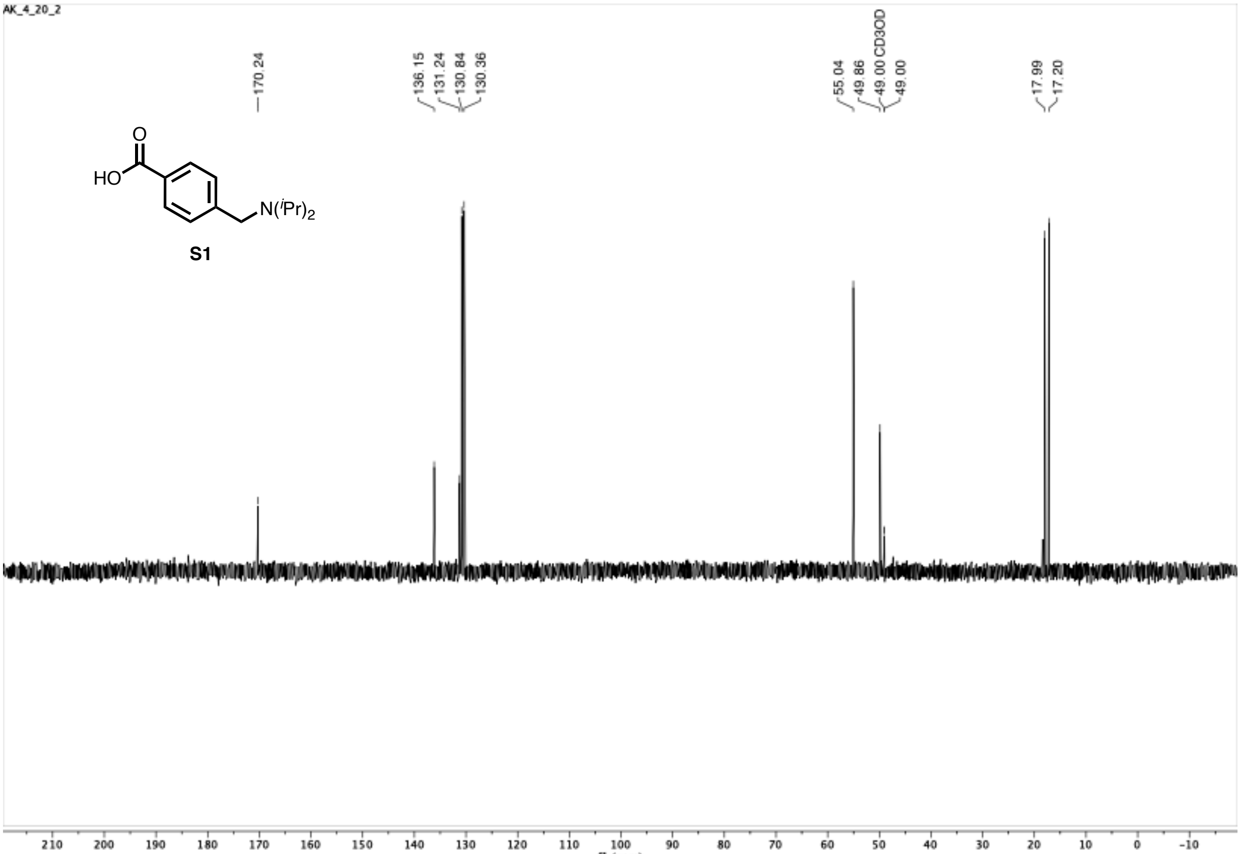

^{1}H-NMR of **S2**

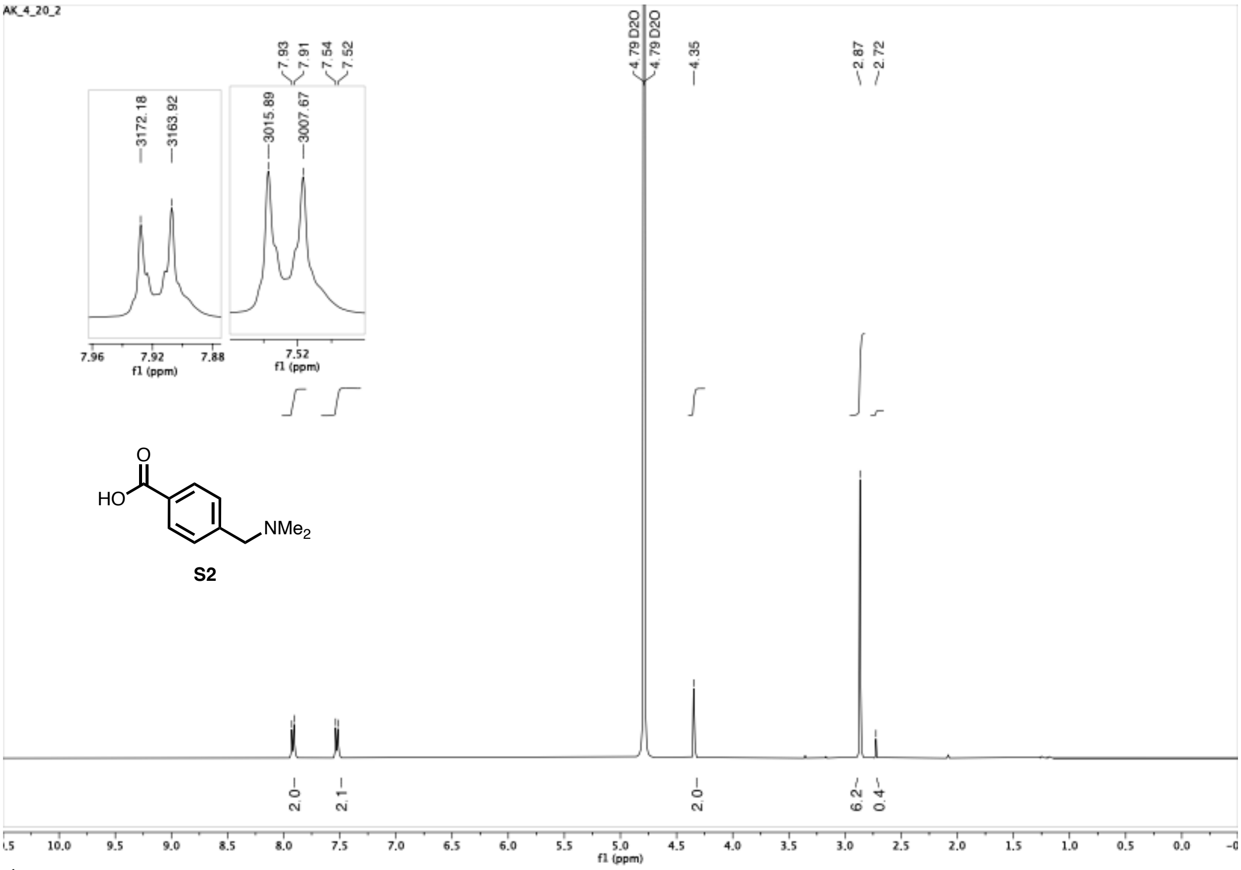

^{13}C{^{1}H}-NMR of **S2**

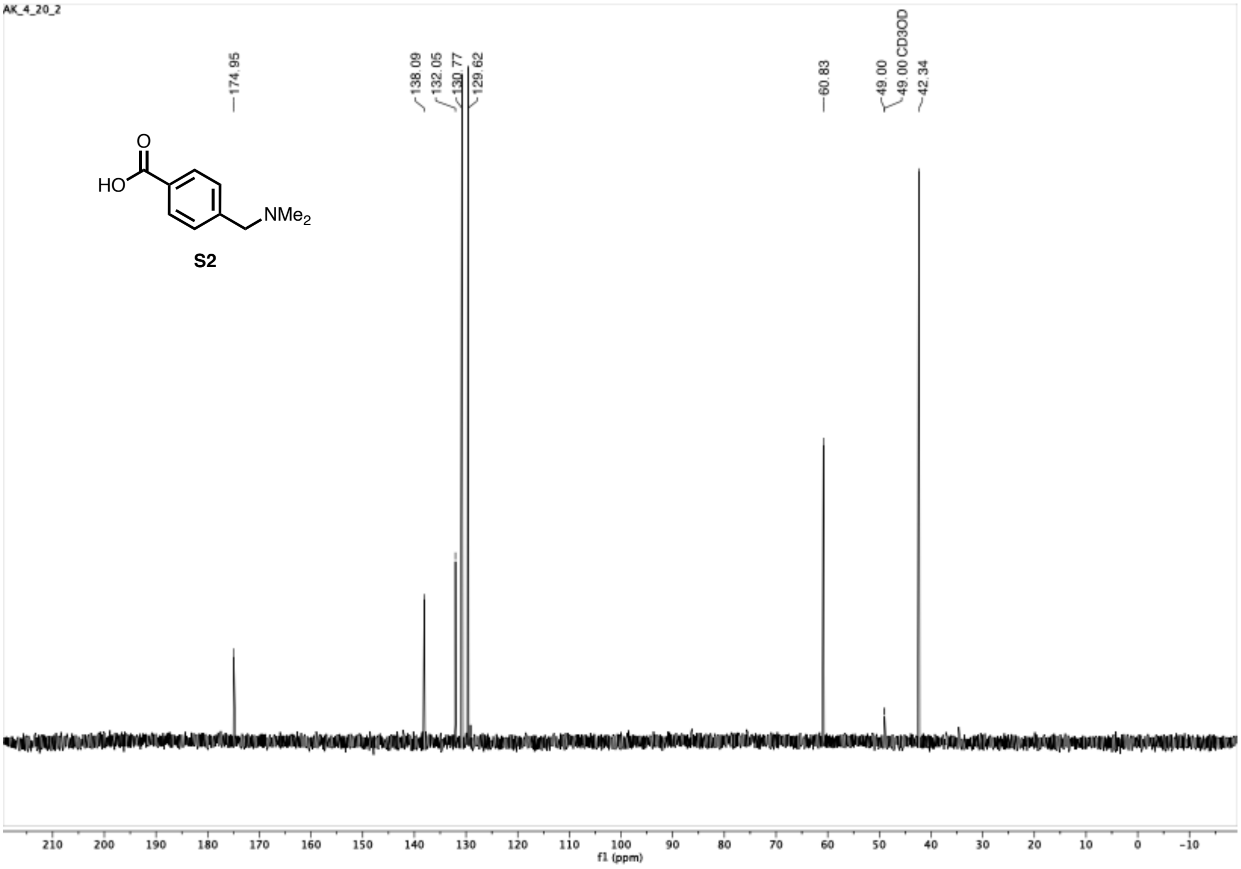

^{1}H-NMR of **S9**

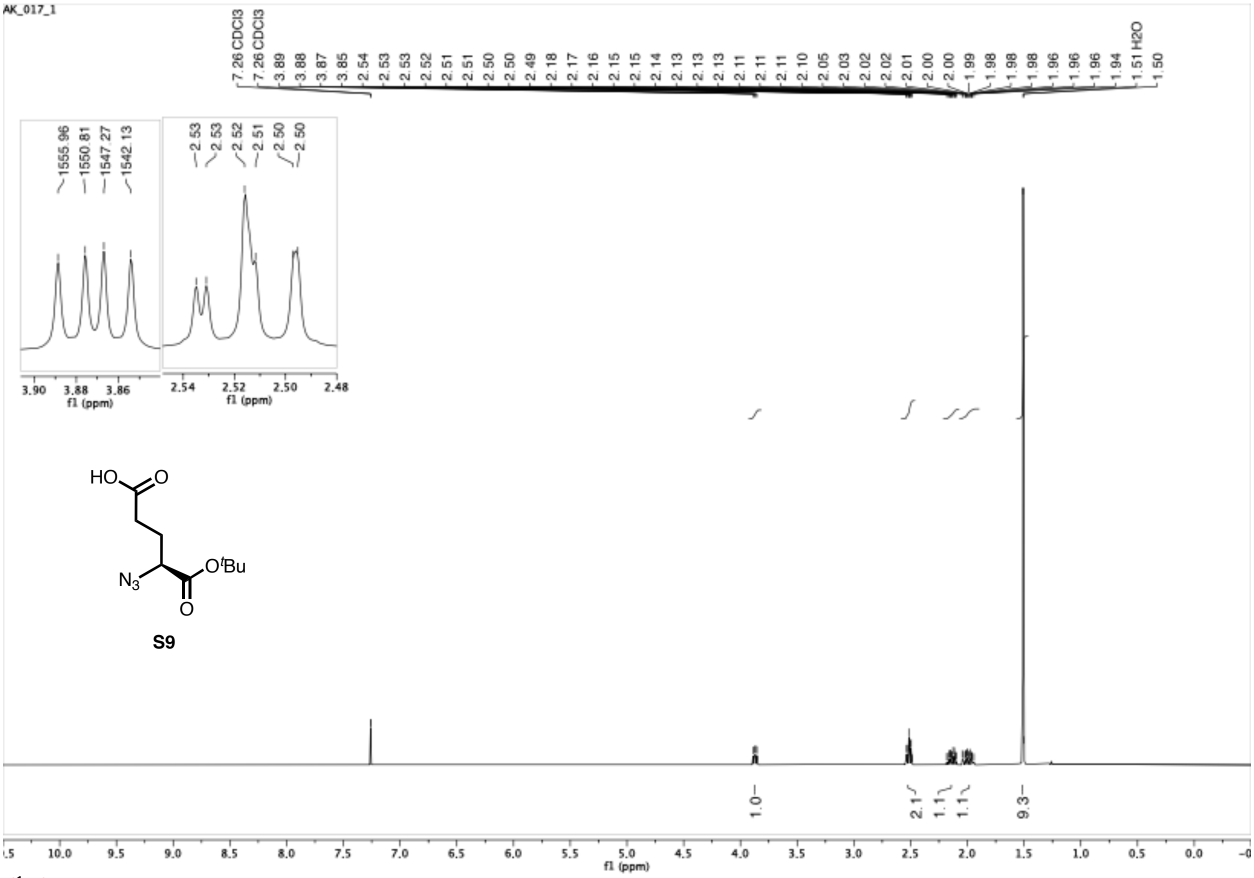

$^{13}C\{^{1}H\}$-NMR of **S9**

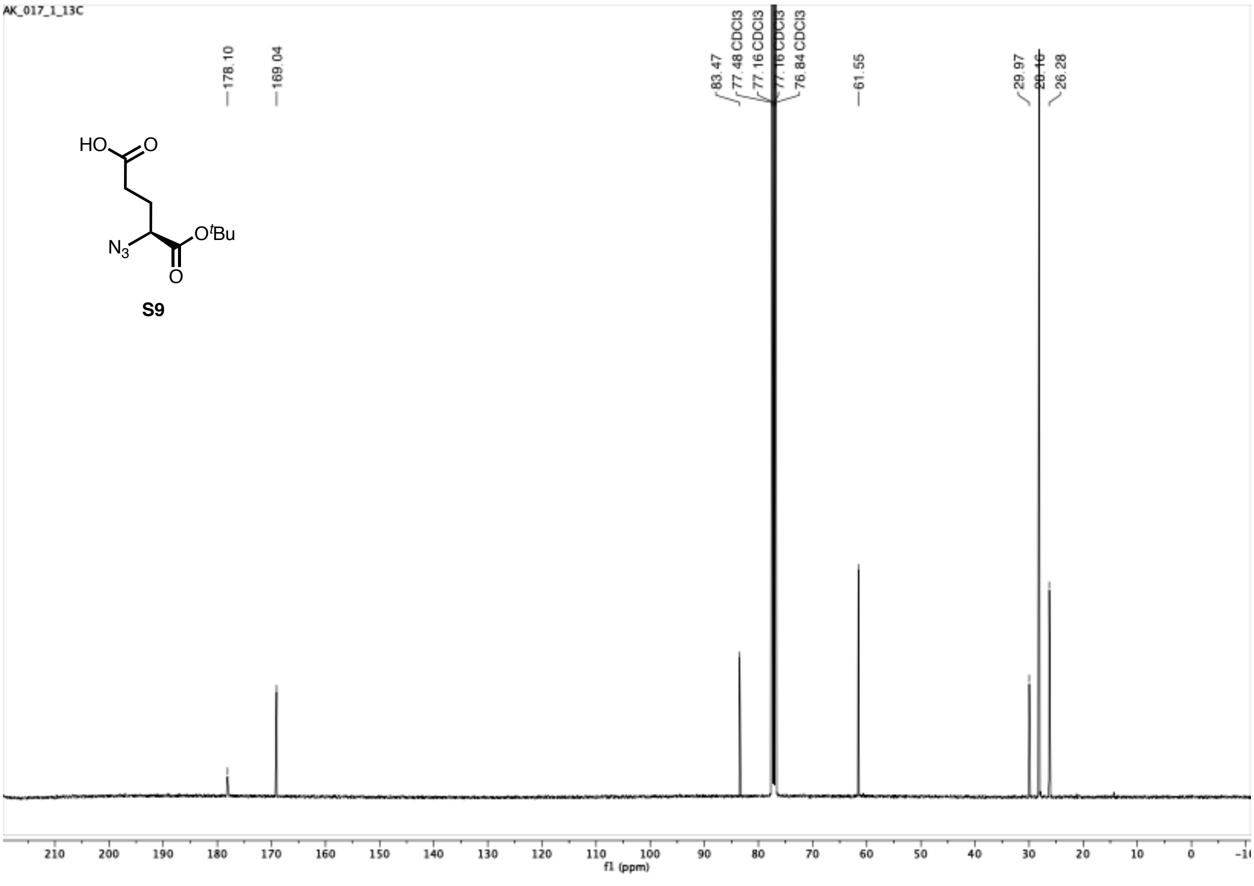

^{1}H-NMR of **S10**

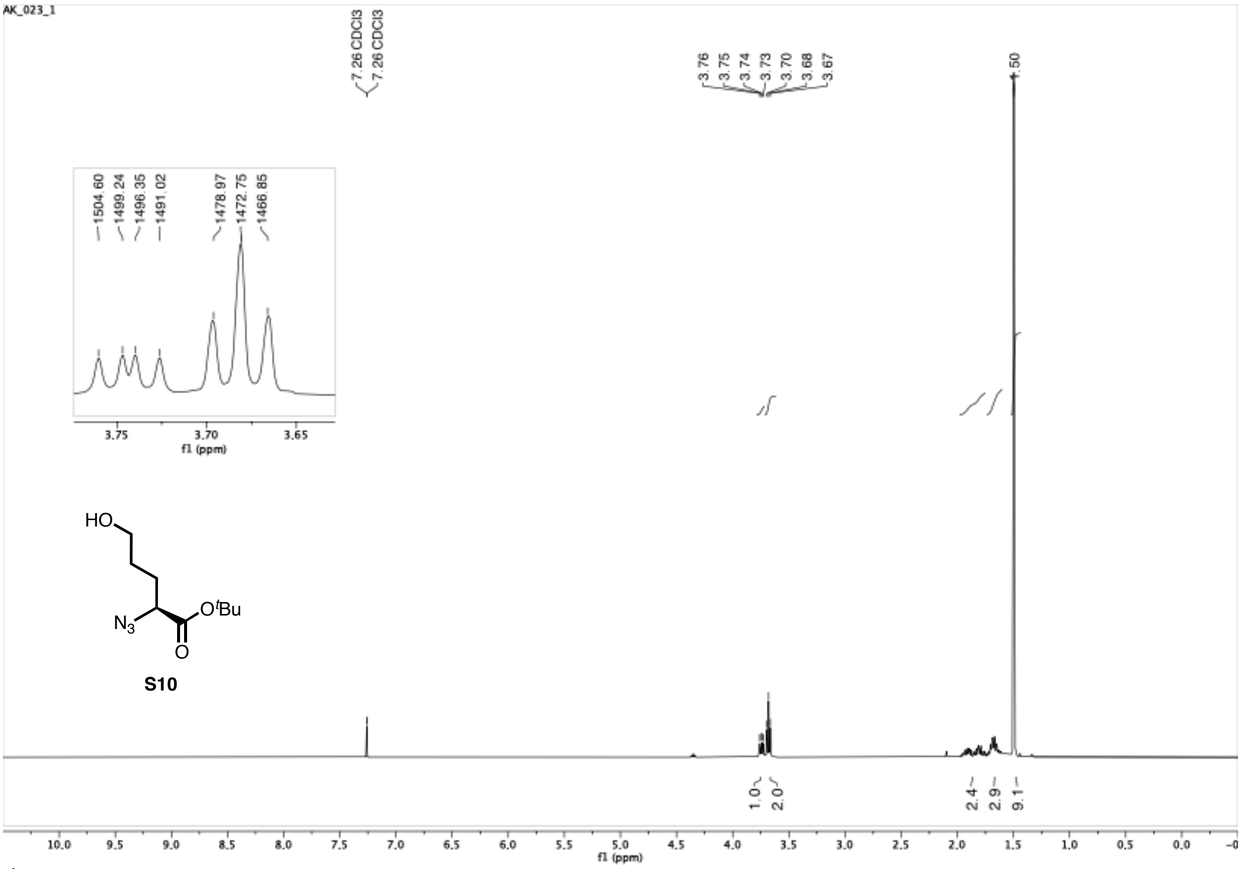

^{13}C{^{1}H}-NMR of **S10**

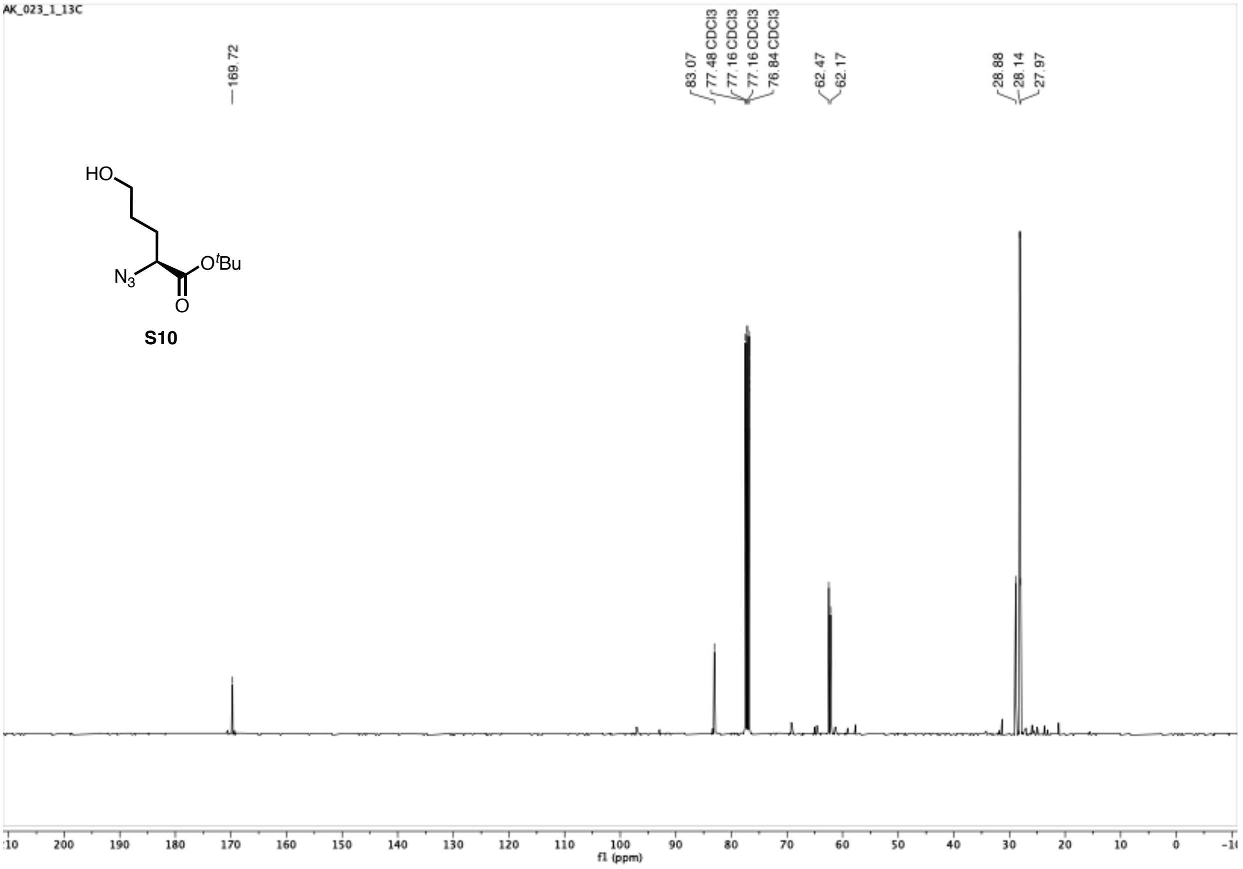

^{1}H-NMR of **S11**

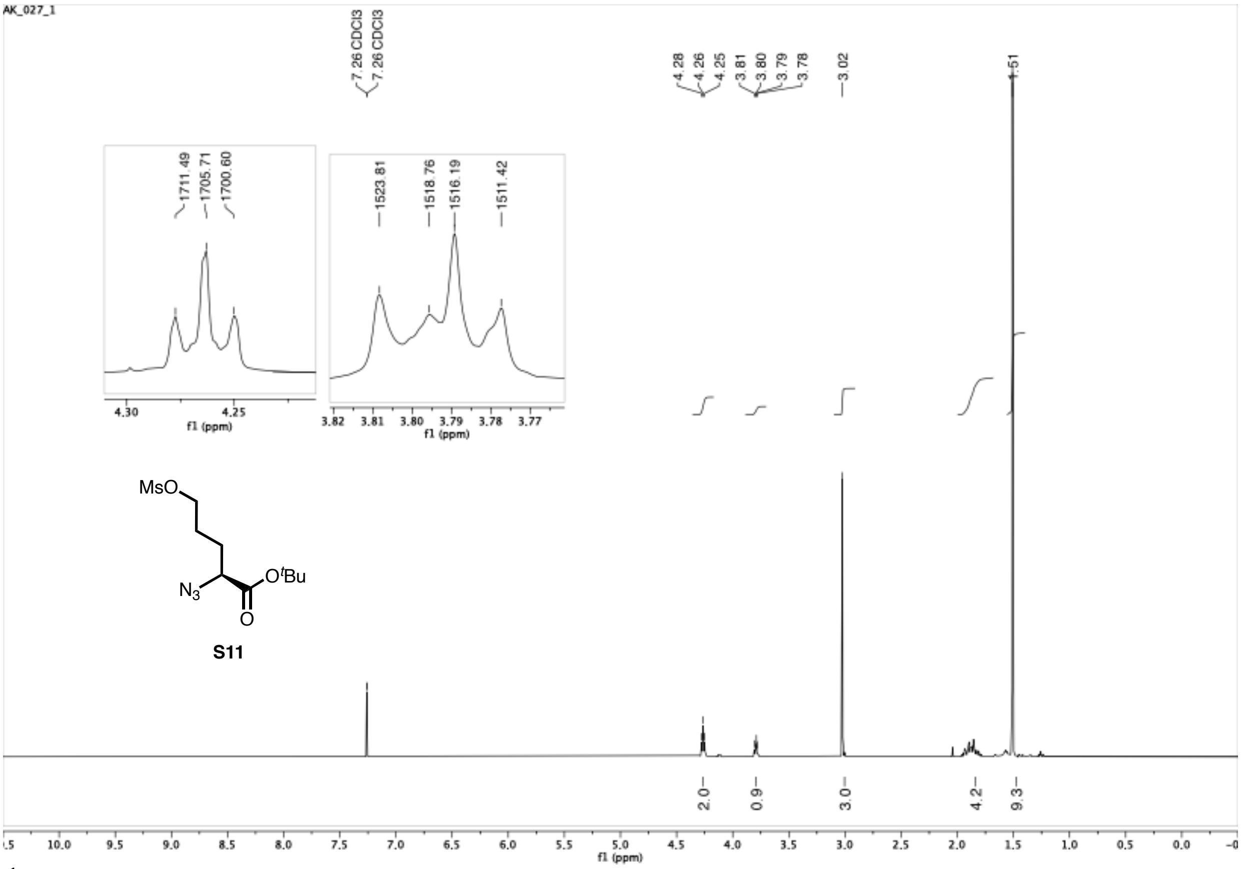

^{13}C{^{1}H}-NMR of **S11**

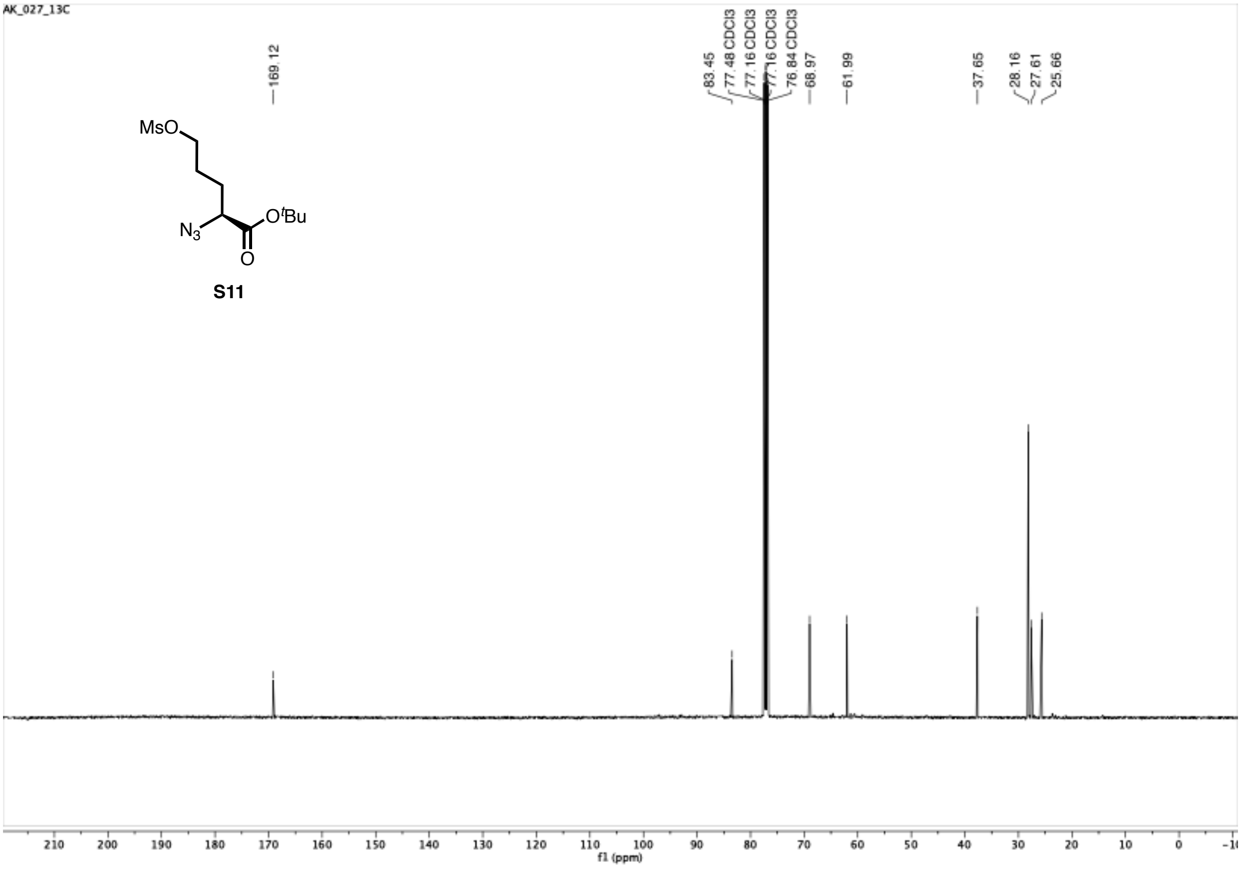

^{1}H-NMR of **S12**

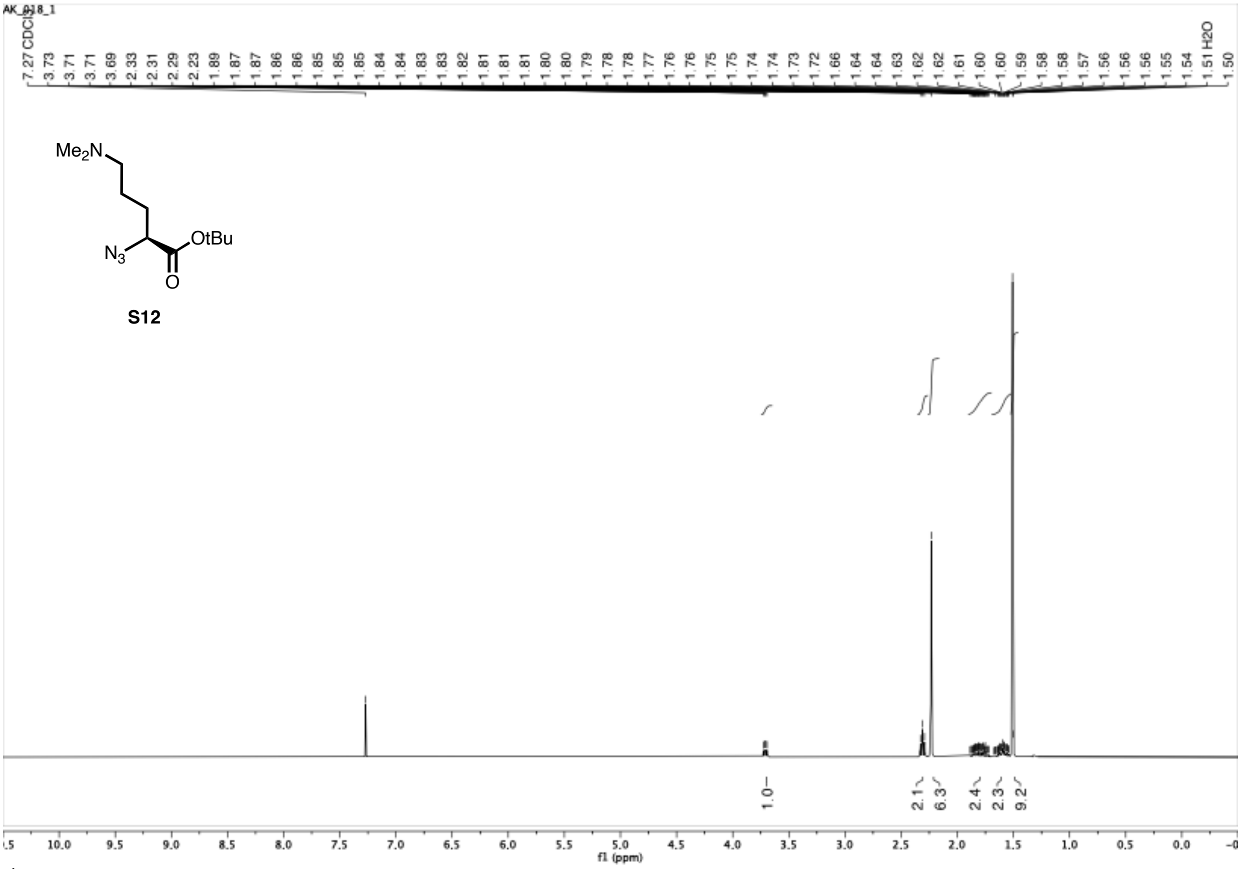

^{13}C{^{1}H}-NMR of **S12**

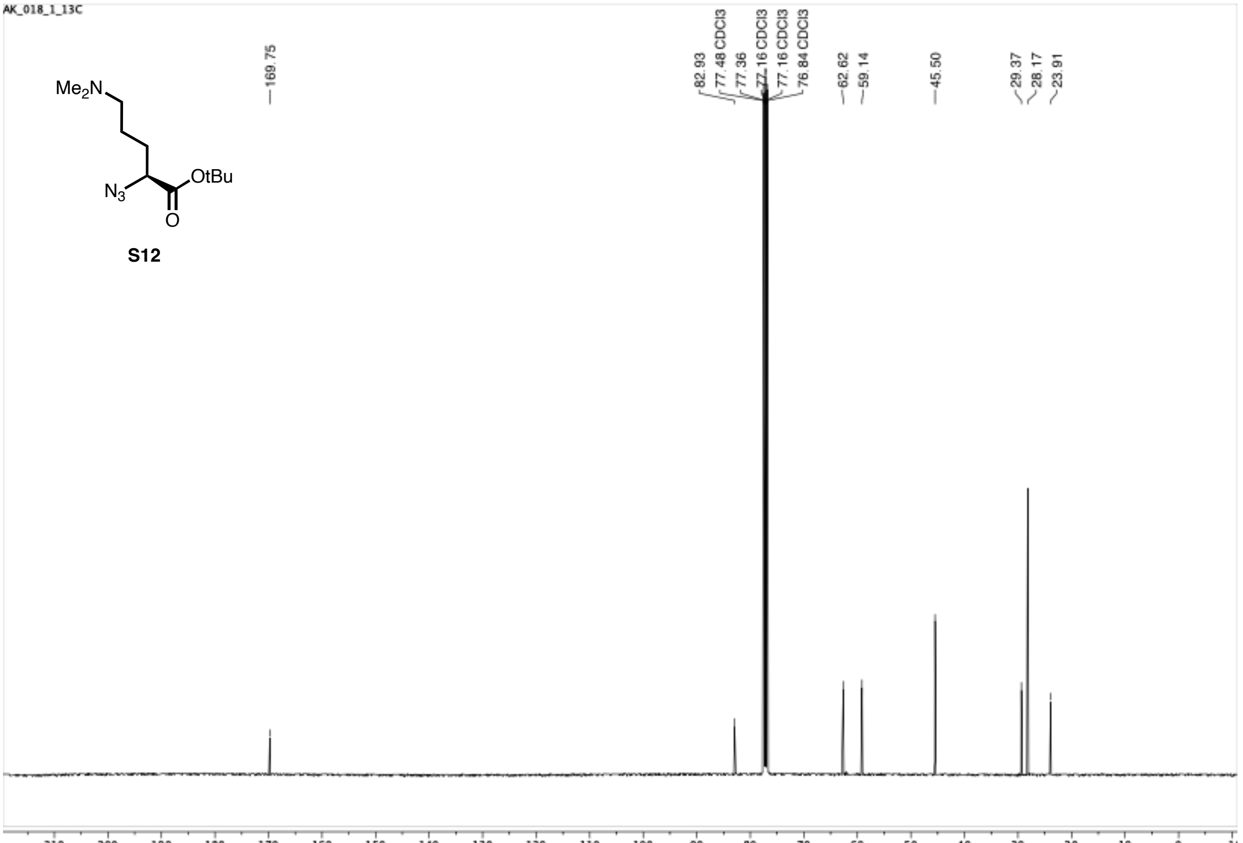

^{1}H-NMR of **S8**

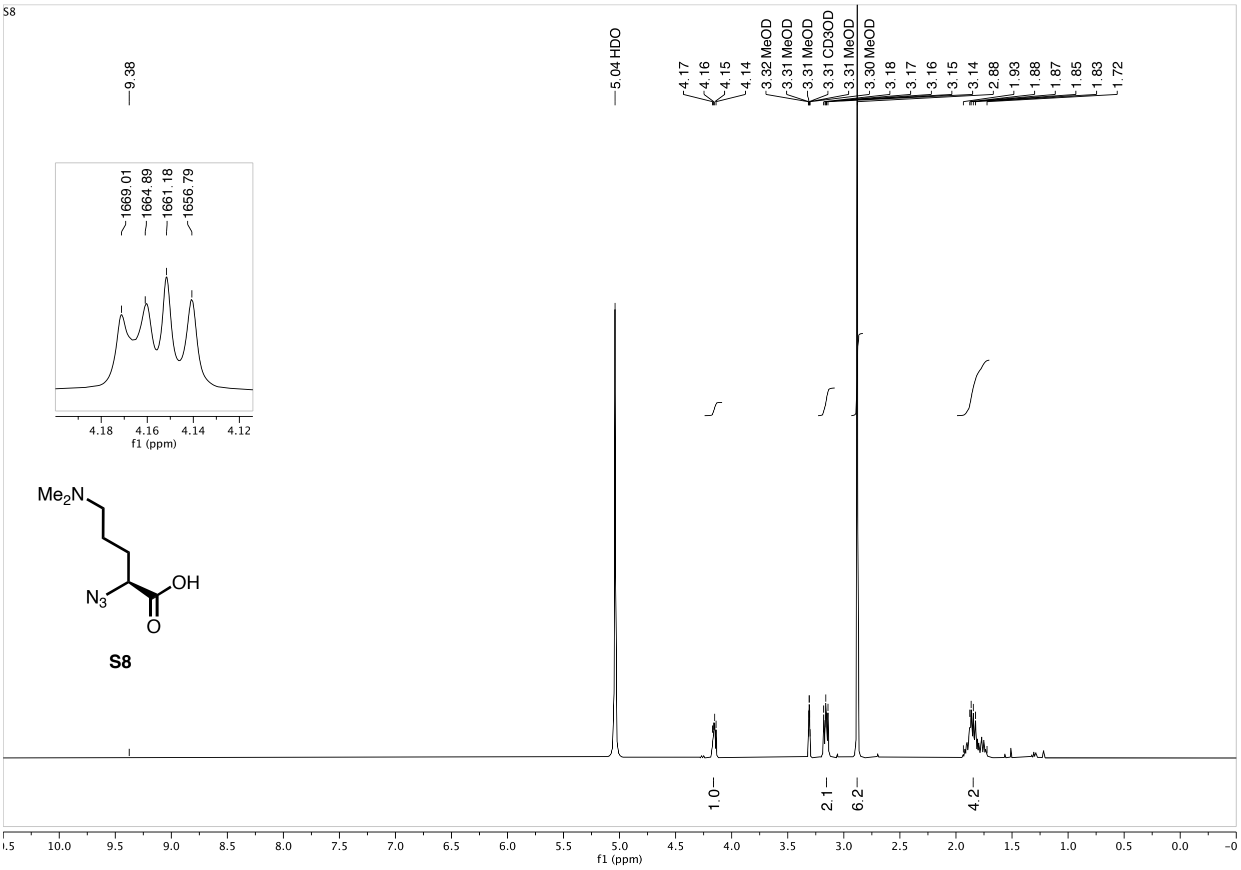

S8

^{1}H-NMR of **S13**

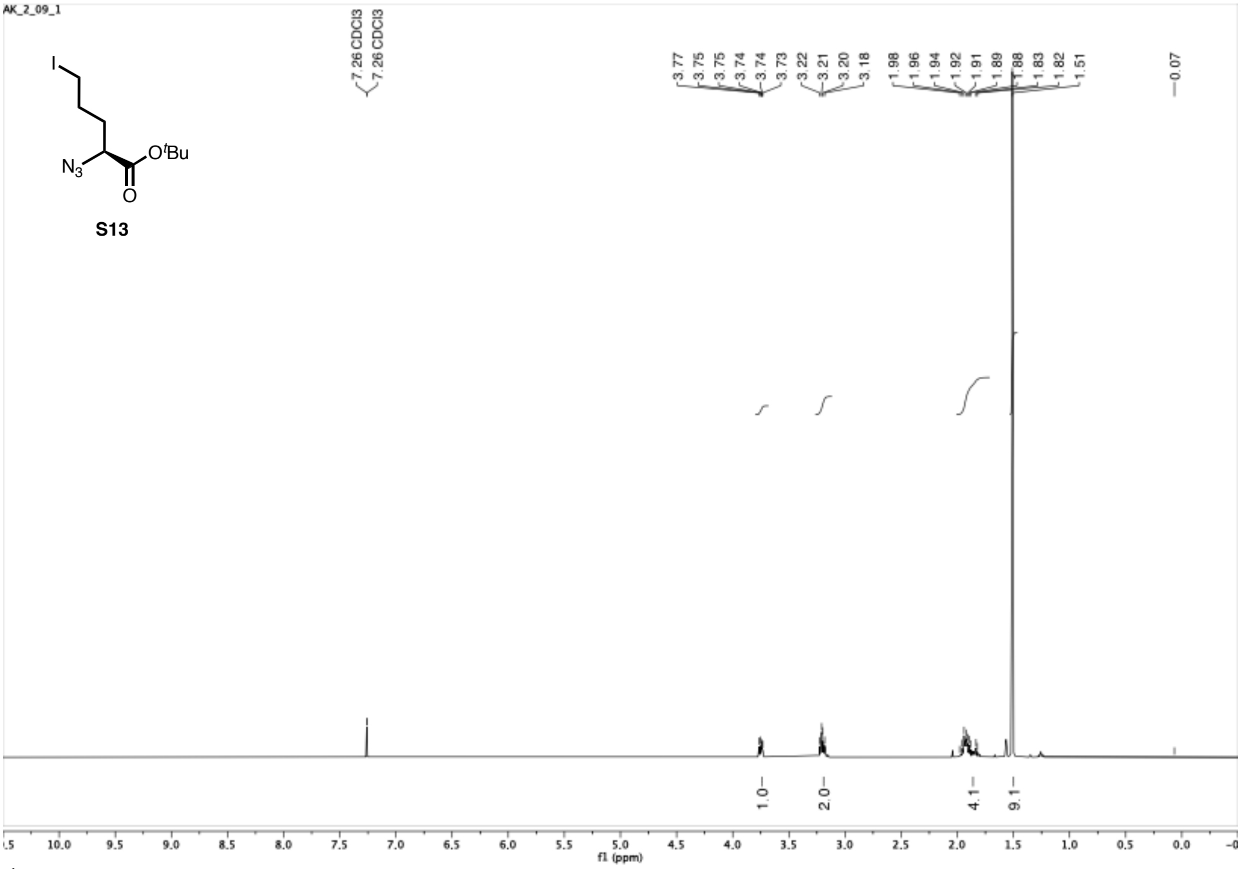

^{13}C{^{1}H}-NMR of **S13**

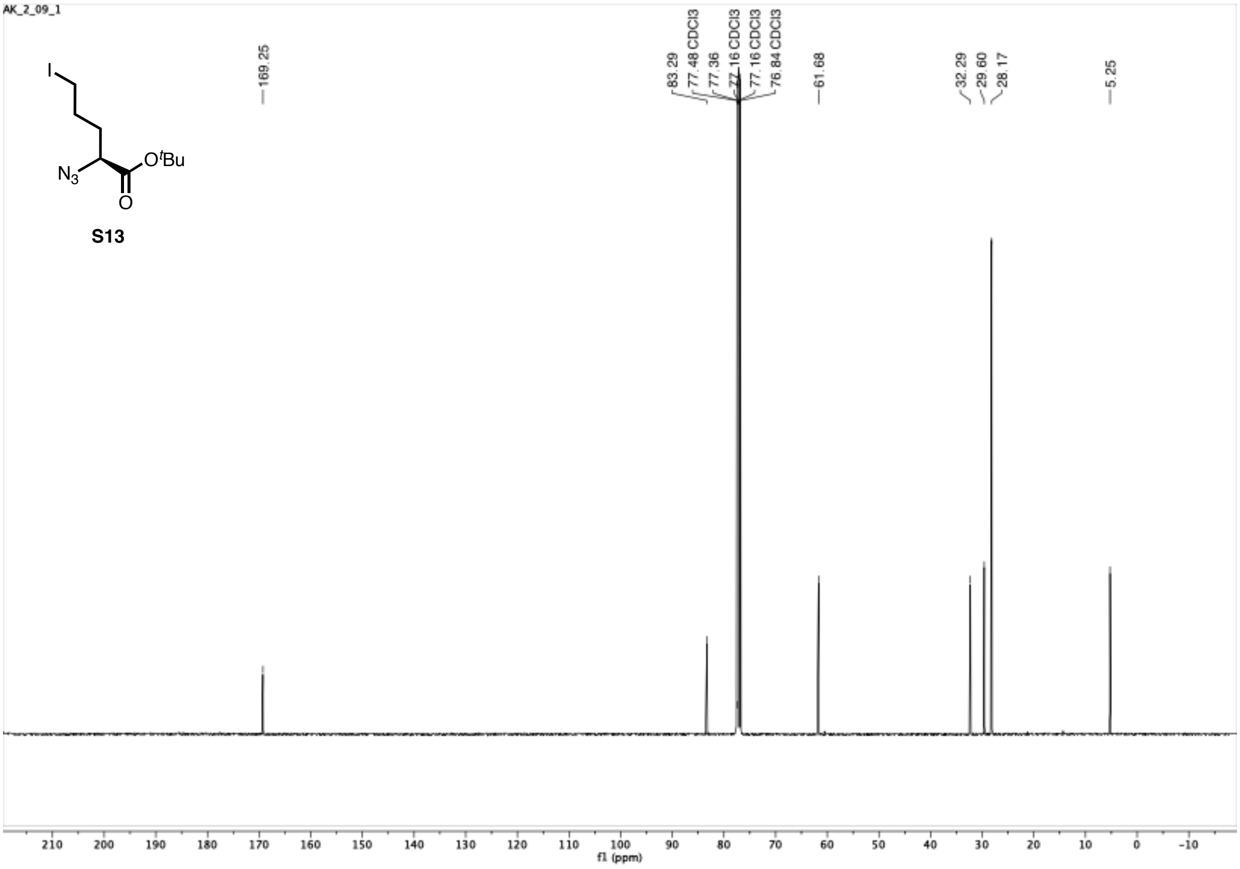

^{1}H-NMR of **S14**

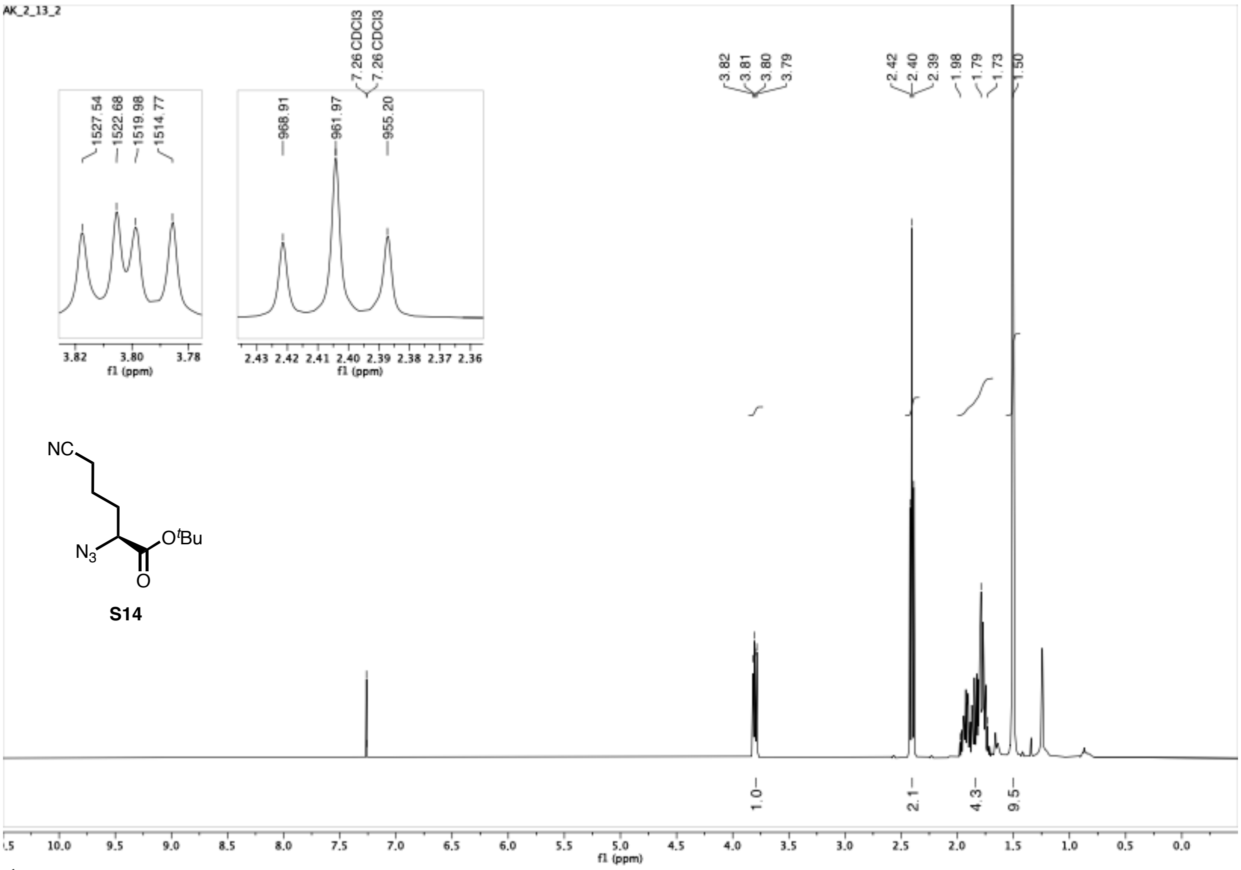

$^{13}C\{^1H\}$-NMR of **S14**

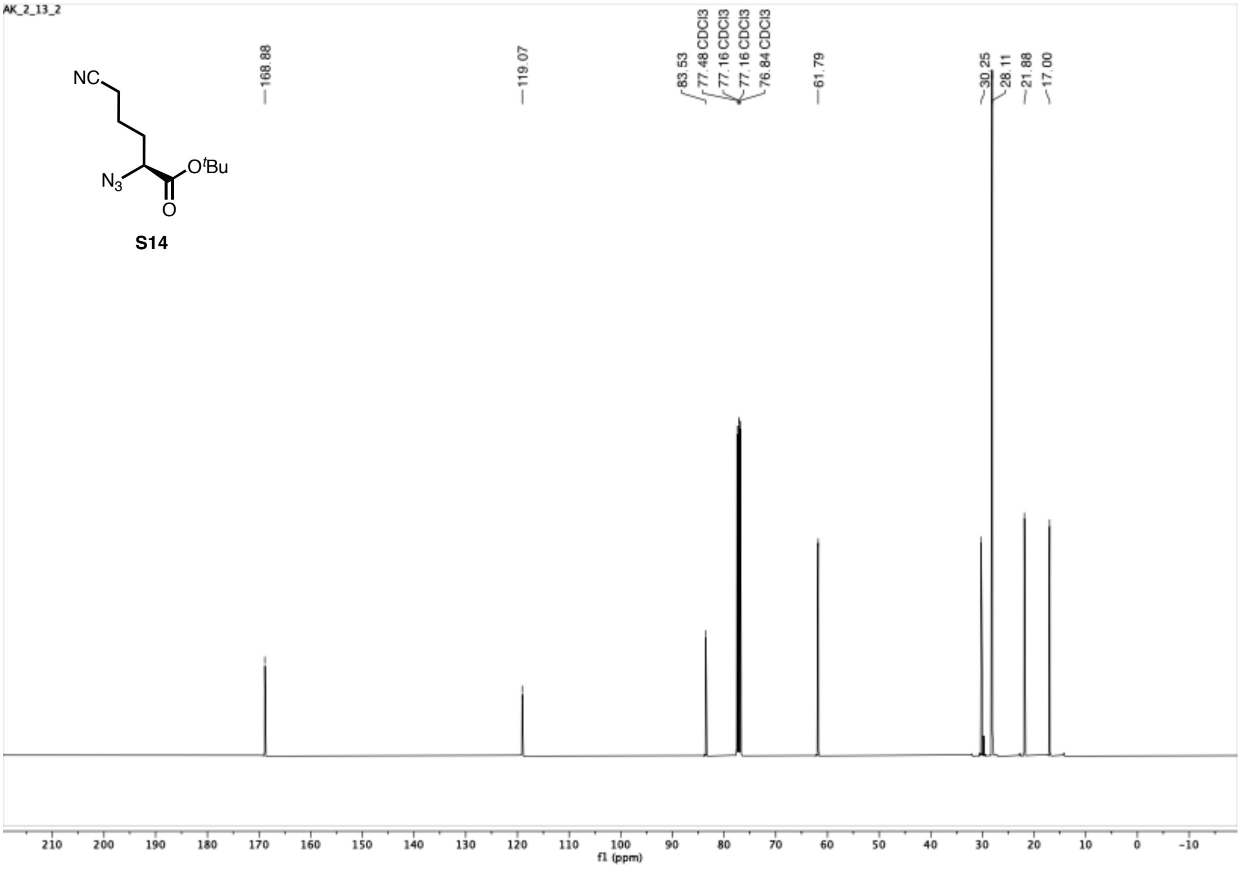

^{1}H-NMR of **S7**

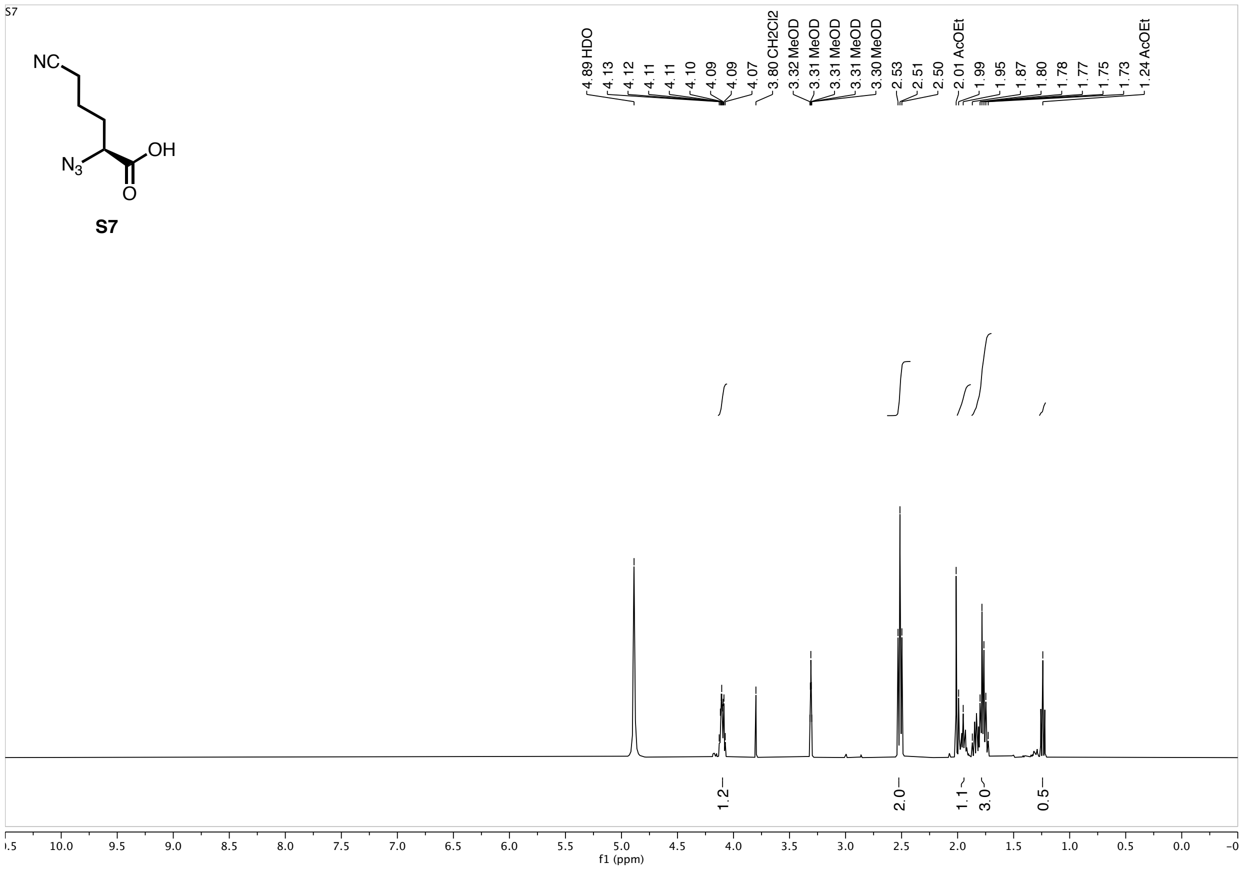
S7
NC
N3
OH
O
S7
4.89 HDO
4.13
4.12
4.11
4.11
4.10
4.09
4.09
4.07
3.80 CH2Cl2
3.32 MeOD
3.31 MeOD
3.31 MeOD
3.31 MeOD
3.30 MeOD
2.53
2.51
2.50
2.01 AcOEt
1.99
1.95
1.87
1.80
1.78
1.77
1.75
1.73
1.24 AcOEt
1.2
2.0
1.1
3.0
0.5
f1 (ppm)

^{1}H-NMR of **S16**

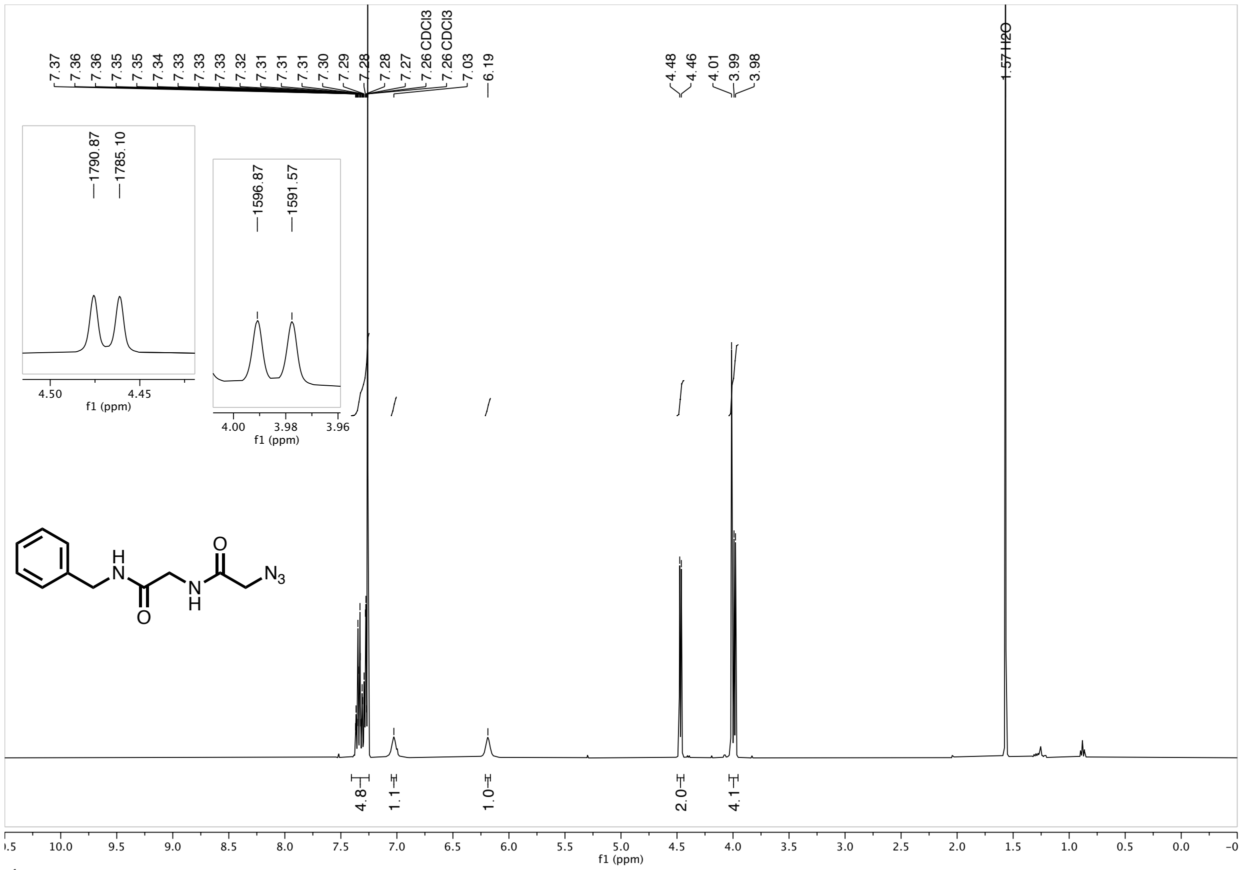

^{13}C{^{1}H}-NMR of **S16**

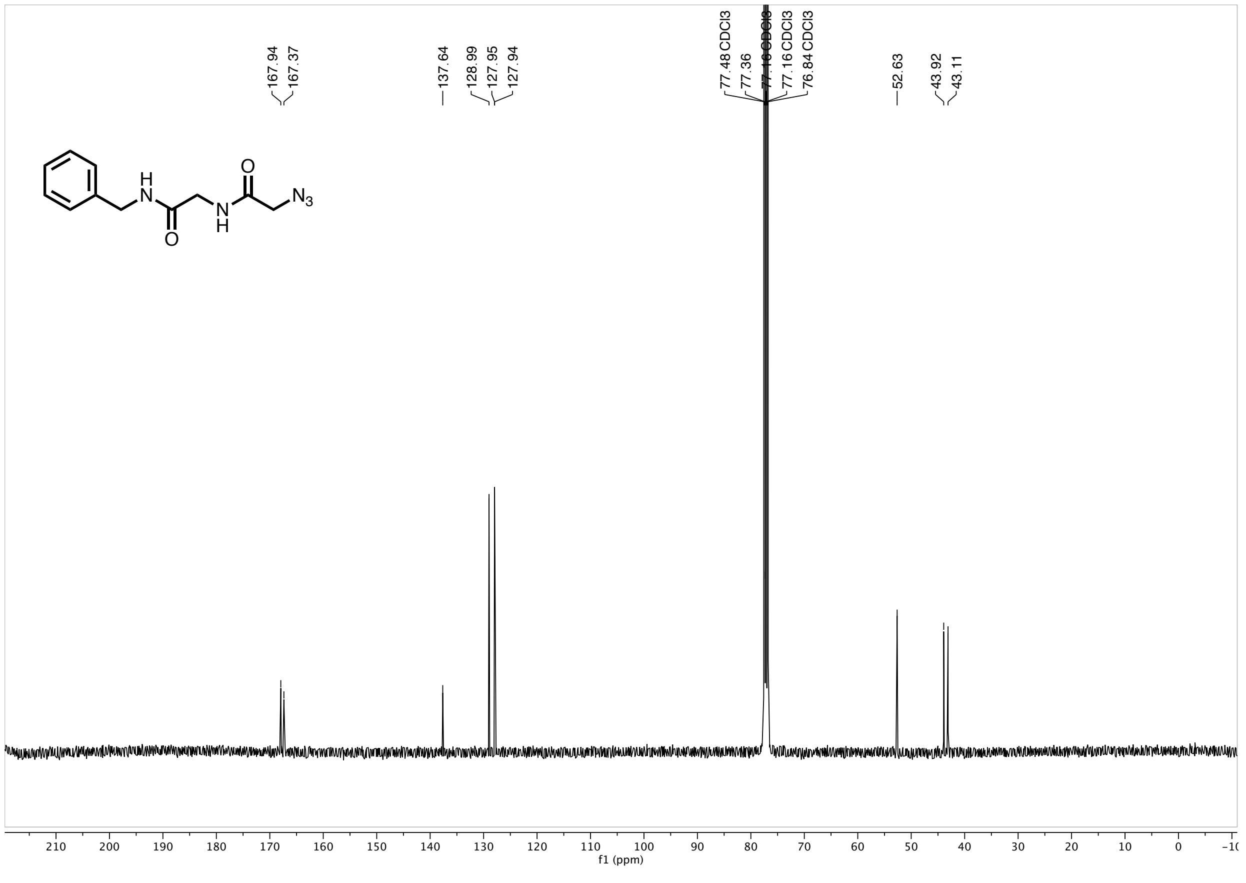

^{1}H-NMR of **S17**

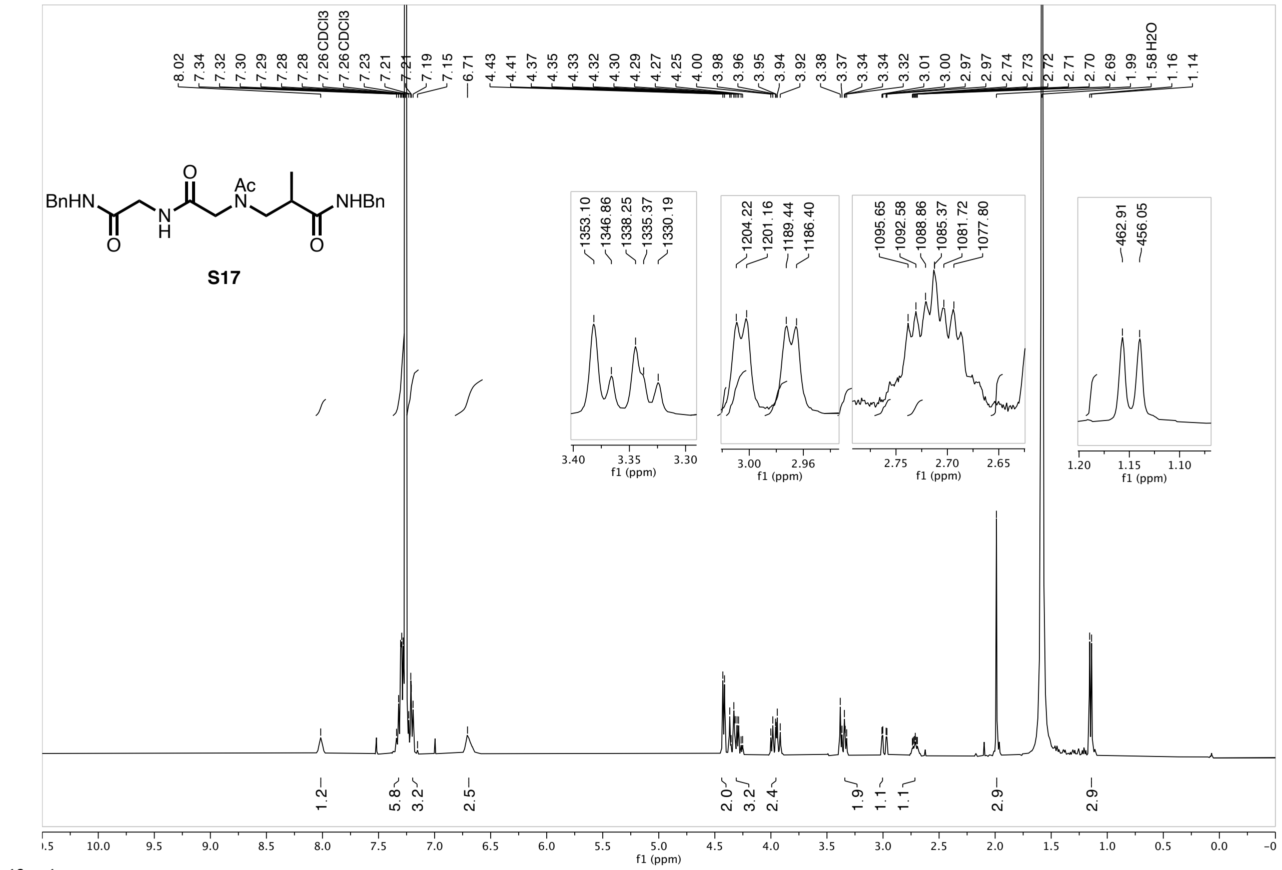

^{13}C{^{1}H}-NMR of **S17**

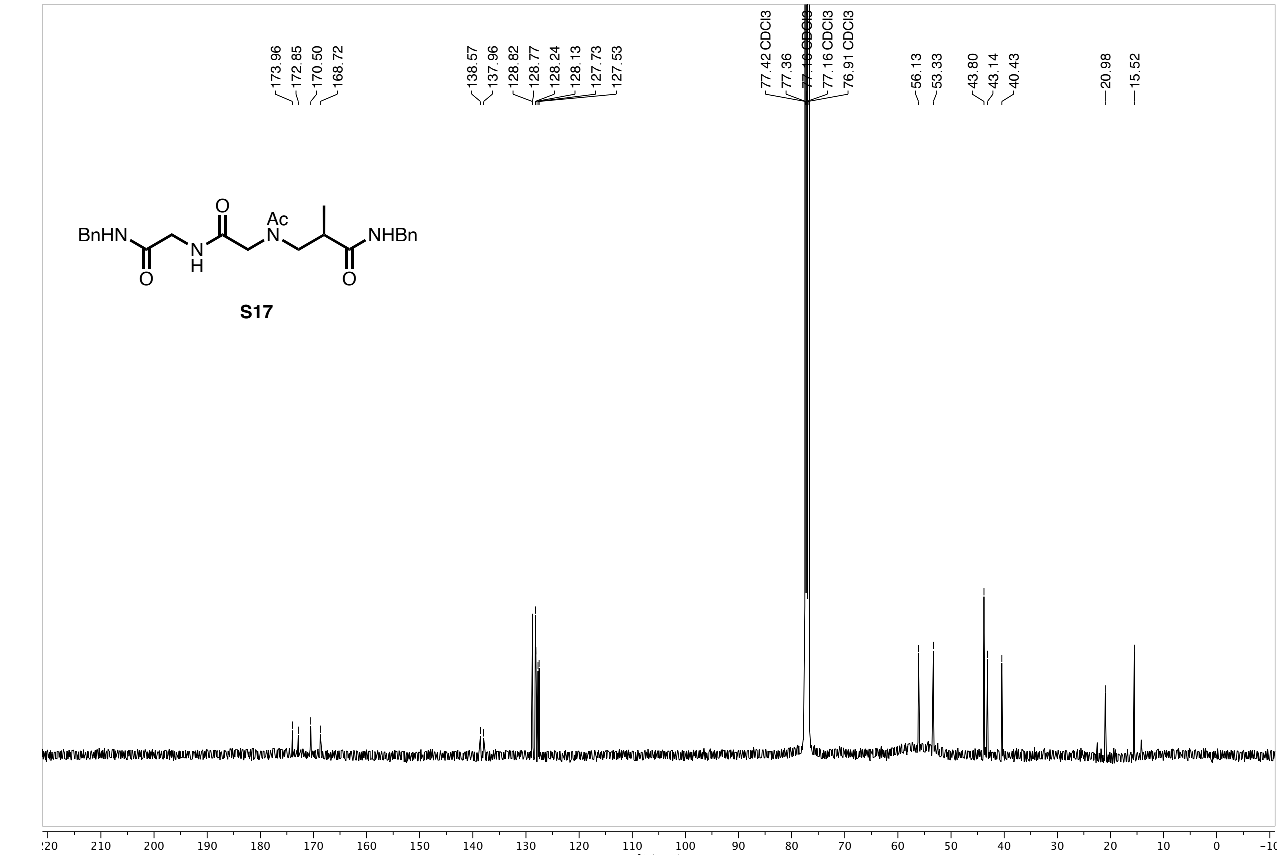

^{1}H-^{1}H COSY of **S17**

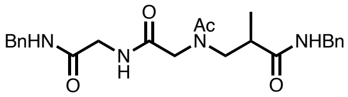

S17

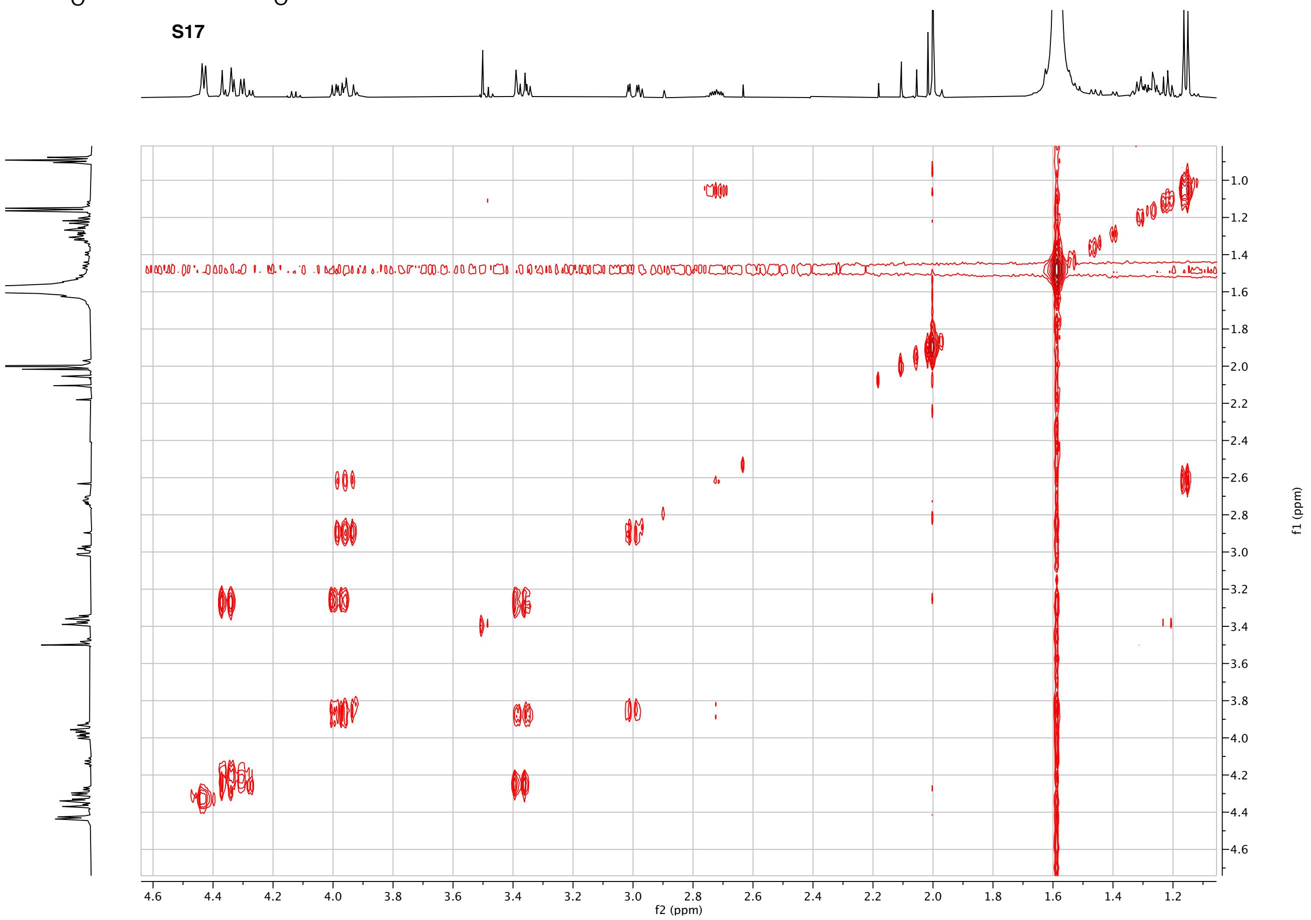

^{1}H-NMR of **3**

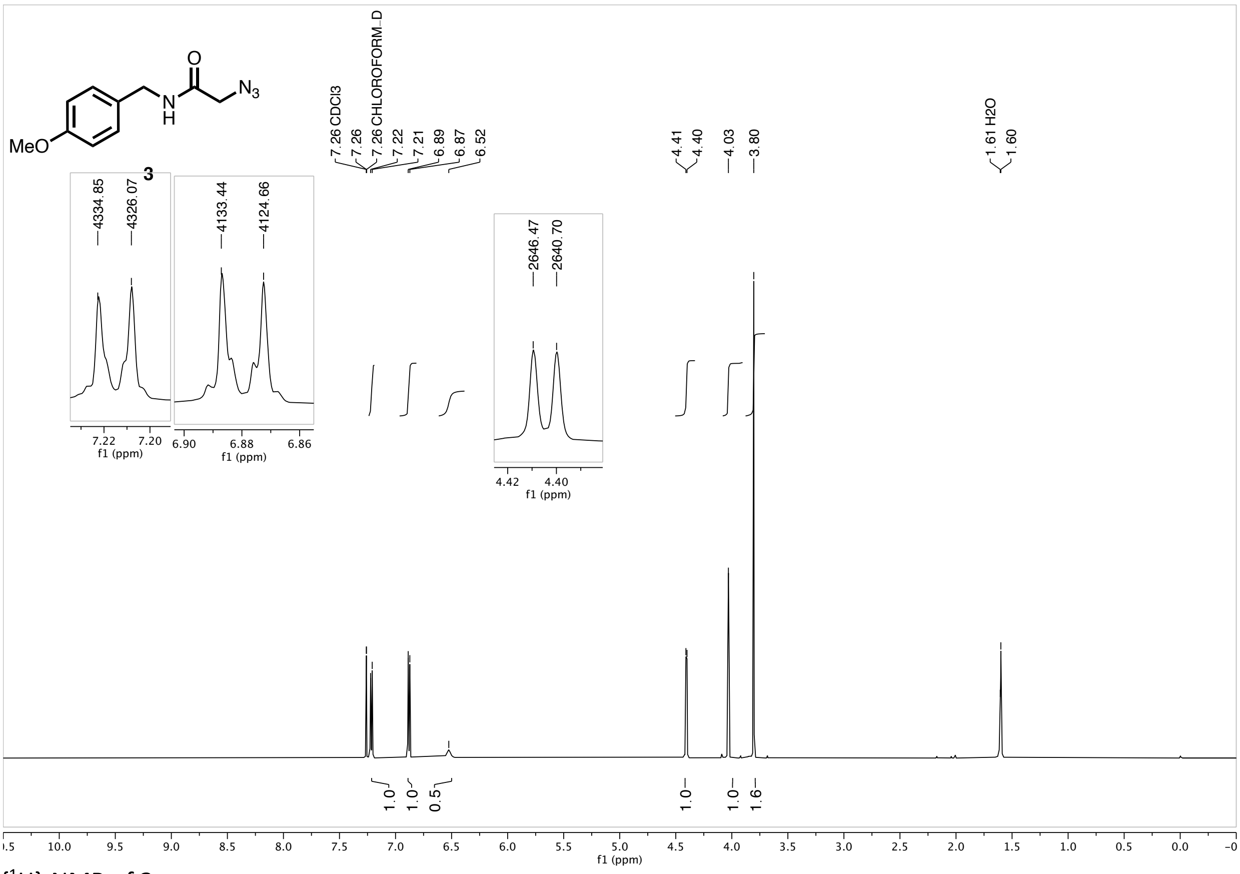

$^{13}C\{^{1}H\}$-NMR of **3**

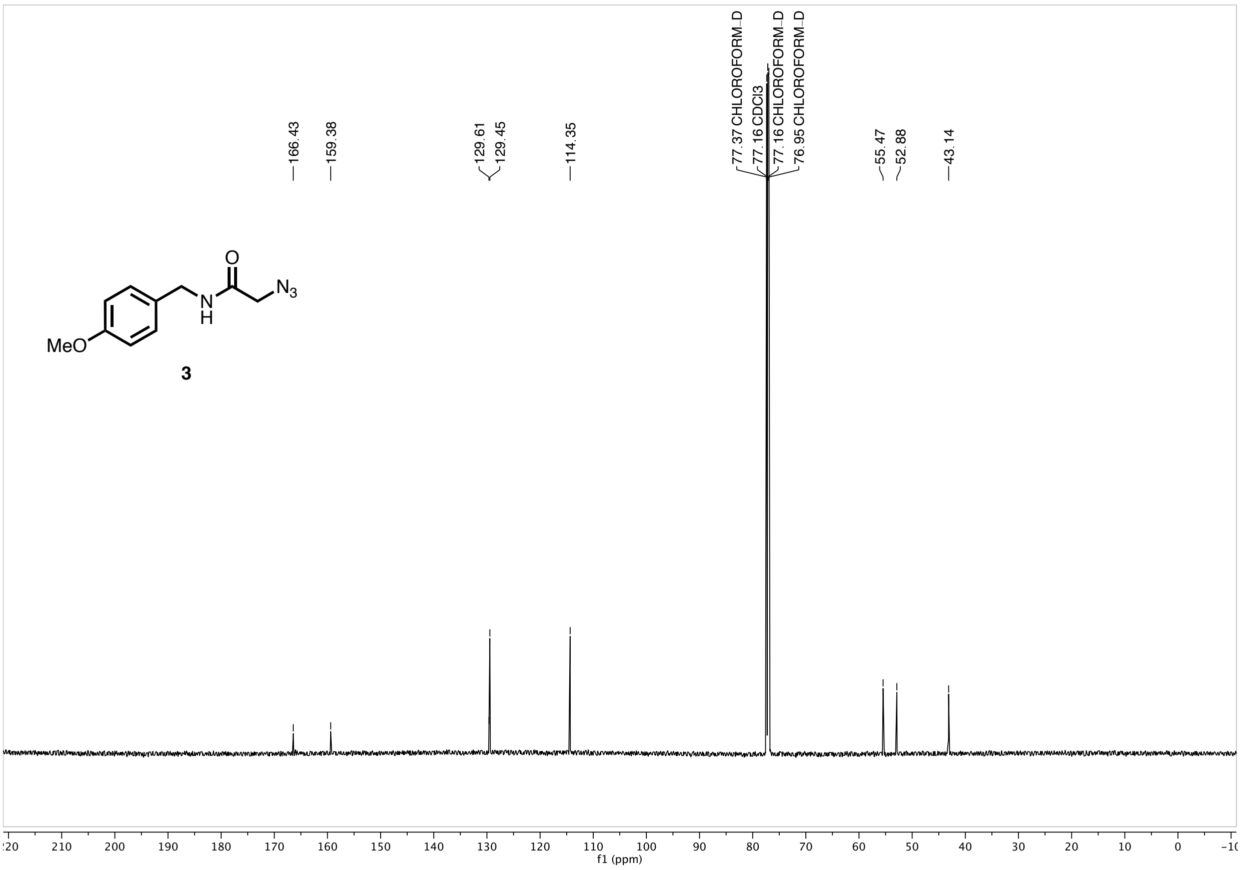

^{1}H-NMR of **8**

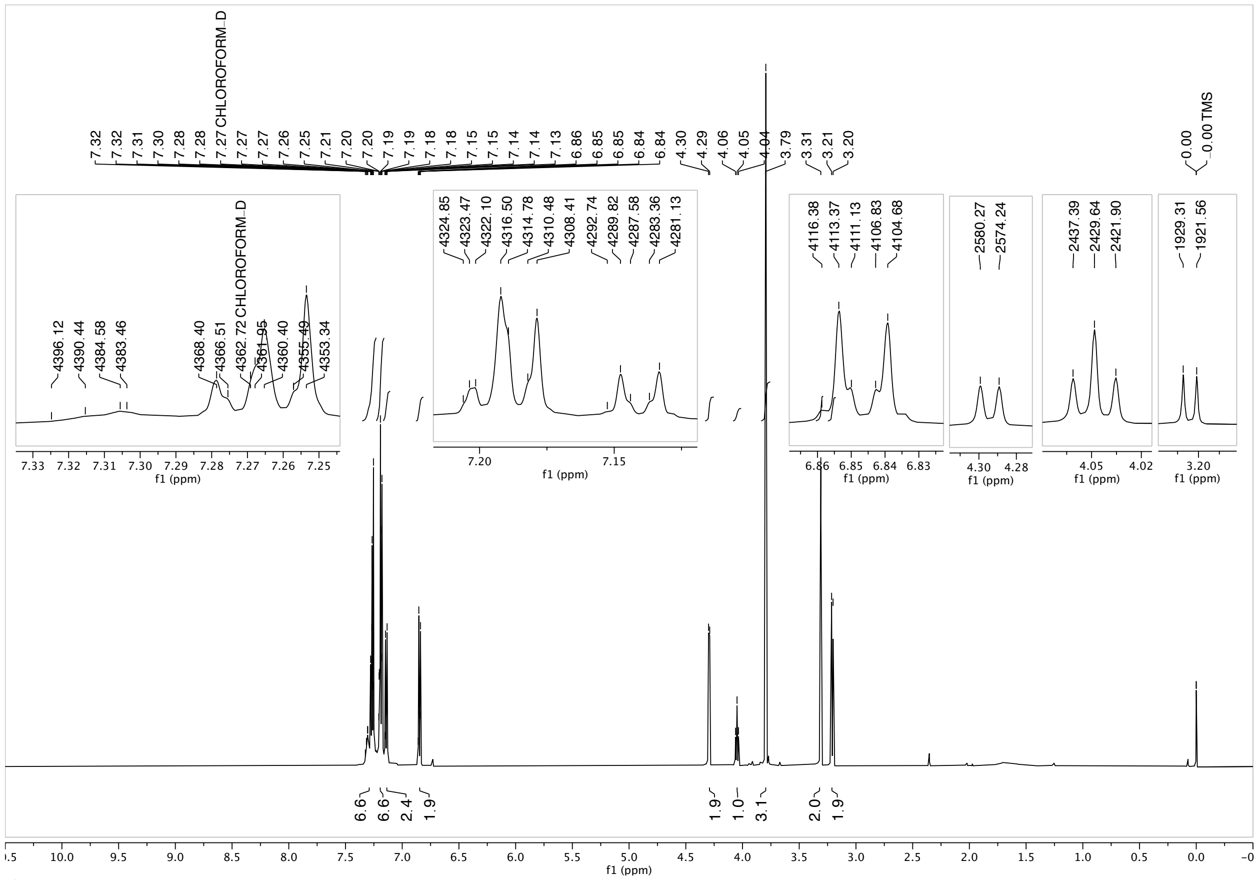

^{13}C{^{1}H}-NMR of **8**

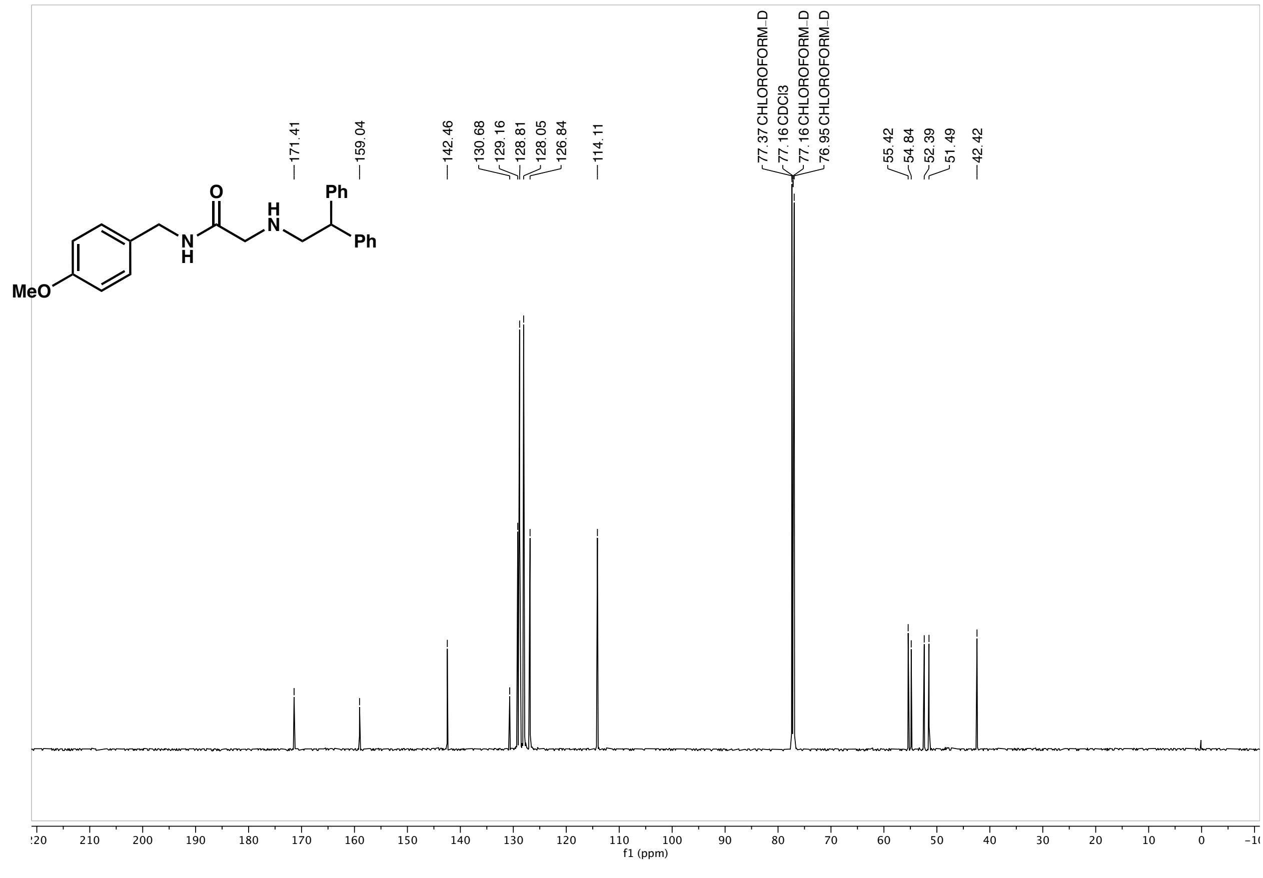

^{1}H-^{1}H COSY of **8**

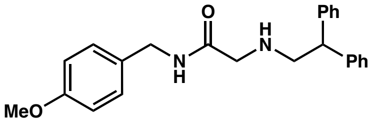

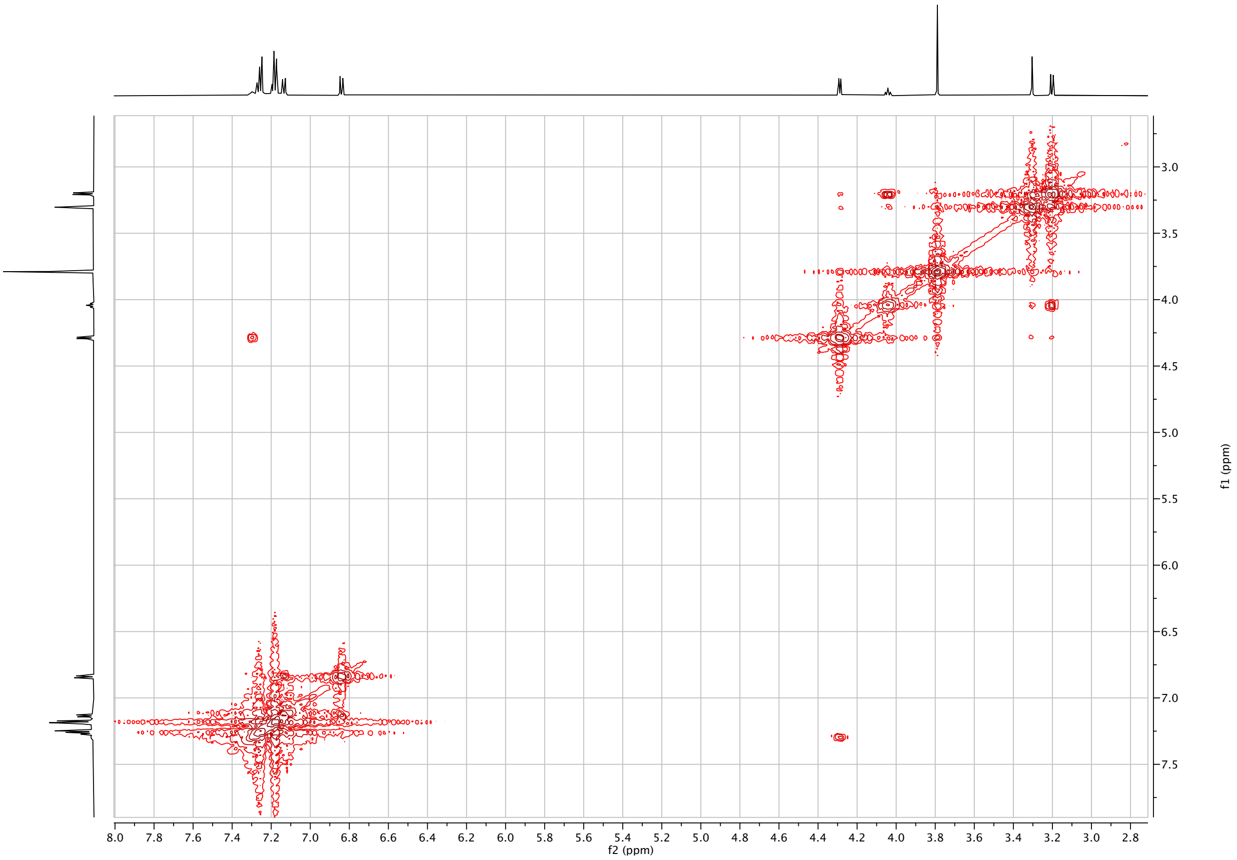

Supplement: SC-017-D5SC08732E-s001 [file SC-017-D5SC08732E-s001.pdf]
