# Supplementary material for: One-pot multi-substrate screening of ligation reactions using PNA tags
Source: Chem Sci. 2026 Feb 20;17(15):7756–65. doi: 10.1039/d5sc08732e (PMC12937129; doi:10.1039/d5sc08732e)
Supplement: SC-017-D5SC08732E-s002 [file SC-017-D5SC08732E-s002.pdf]

Supplementary Information (SI) for Chemical Science.
This journal is © The Royal Society of Chemistry 2026

X-GCGGCG

X1

Sequence: 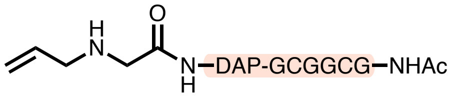

Chemical Formula: $C_{74}H_{96}N_{42}O_{21}$, Exact Mass: 1908.77

LC-MS (ESI) RT = 1.03 min, m/z found: 956.00 $[M+2H]^{2+}$, 637.83 $[M+3H]^{3+}$; calc.*1 955.39 $[M+2H]^{2+}$, 637.27 $[M+3H]^{3+}$

MALDI-TOF m/z found*2 1910.165 $[M+H]^{+}$, 1932.122 $[M+Na]^{+}$; calc.*3 1909.7808 $[M+H]^{+}$, 1931.7627 $[M+Na]^{+}$

*1 It was calculated with enviPad using centroid mode.

*2 Reflector mode was used.

*3 It was calculated with enviPad using profile mode.

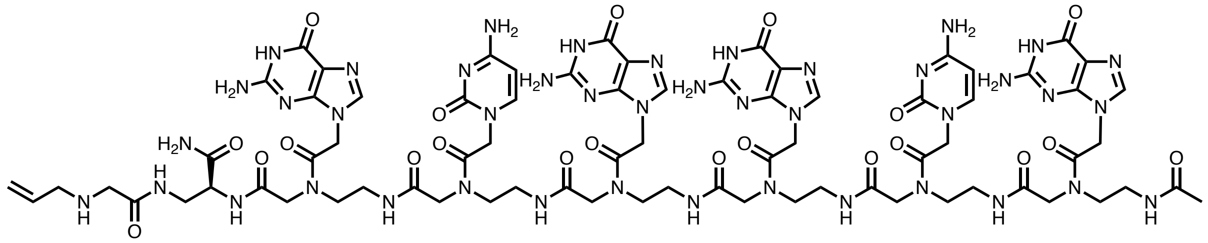

m/z: 1908.77 (100.0%), 1909.78 (81.9%), 1910.78 (37.6%)

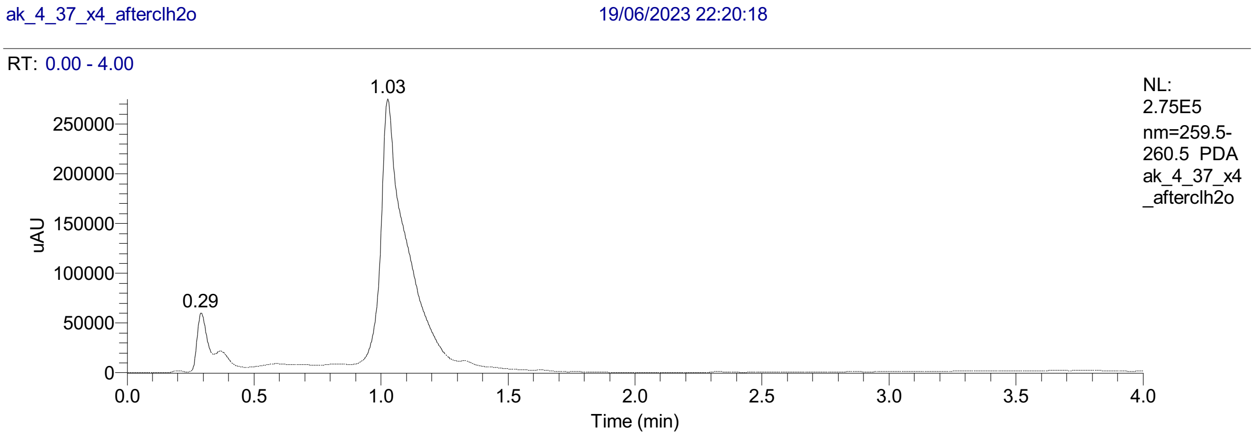

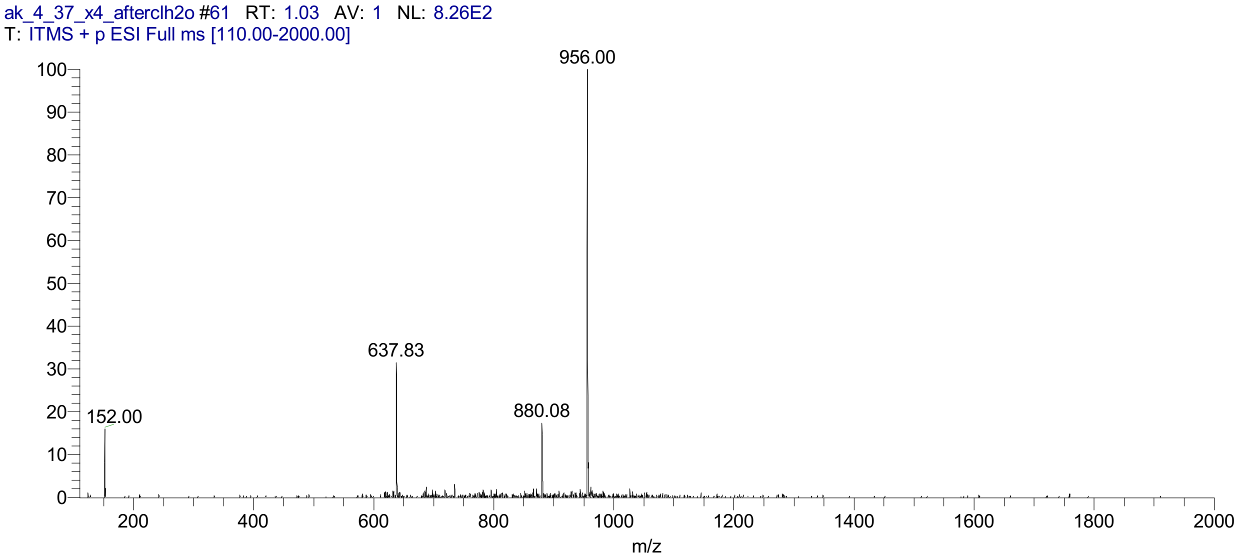

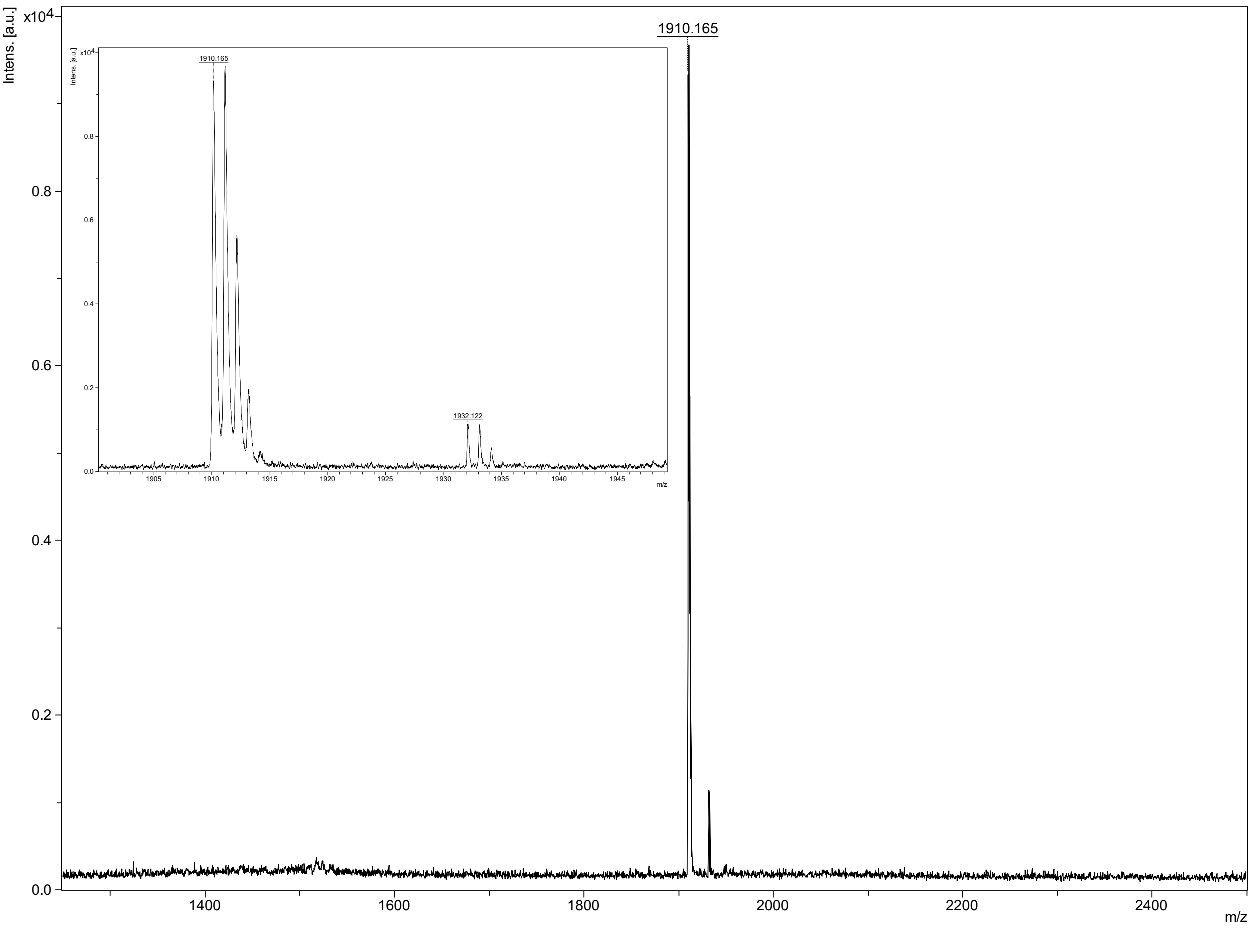

1910.165
Intens. [a.u.]
x10⁴
0.8
0.6
0.4
0.2
0.0
1400
1600
1800
2000
2200
2400
m/z
1932.122

X2

Sequence: 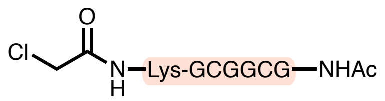

Chemical Formula: $C_{74}H_{96}ClN_{41}O_{21}$, Exact Mass: 1929.73

LC-MS (ESI) RT = 1.15 min, m/z found: 966.33 $[M+2H]^{2+}$, 644.58 $[M+3H]^{3+}$; 965.88 $[M+2H]^{2+}$, 644.25 $[M+3H]^{3+}$

MALDI-TOF m/z found 1931.16 $[M+H]^{+}$, 1953.13 $[M+Na]^{+}$; calc. 1930.75 $[M+H]^{+}$, 1952.73 $[M+Na]^{+}$

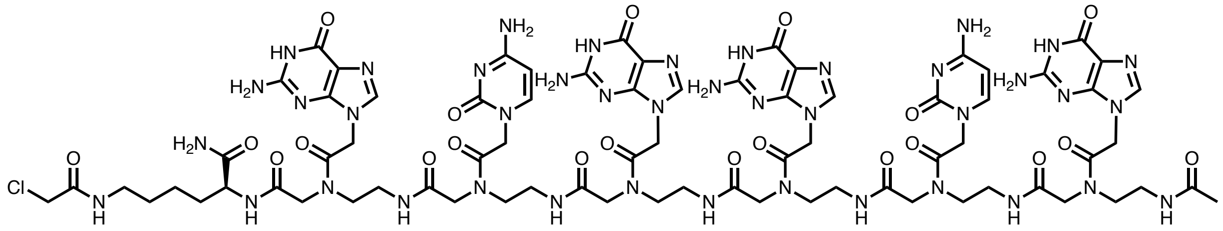

m/z: 1929.74 (100.0%), 1930.74 (96.0%), 1931.74 (48.7%), 1931.75 (33.1%), 1932.74 (32.6%)

ak_4_35_x9_aftercl_h2o 19/06/2023 22:58:04

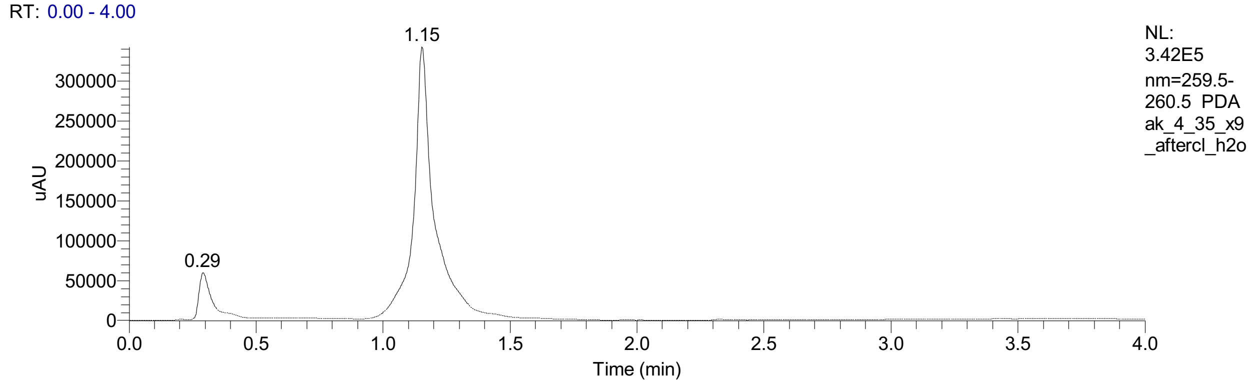

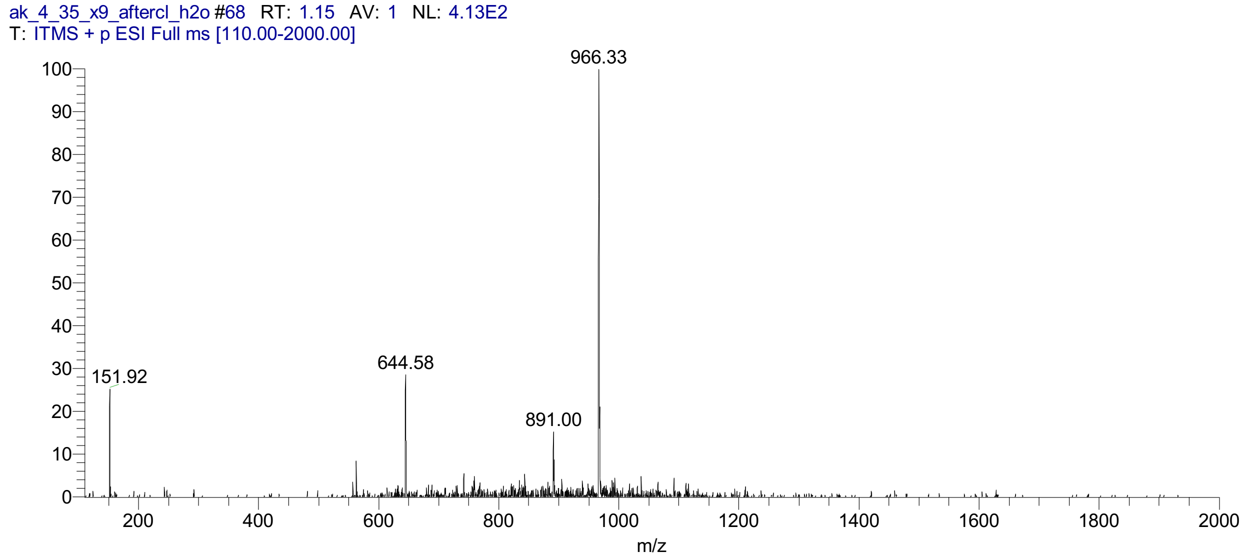

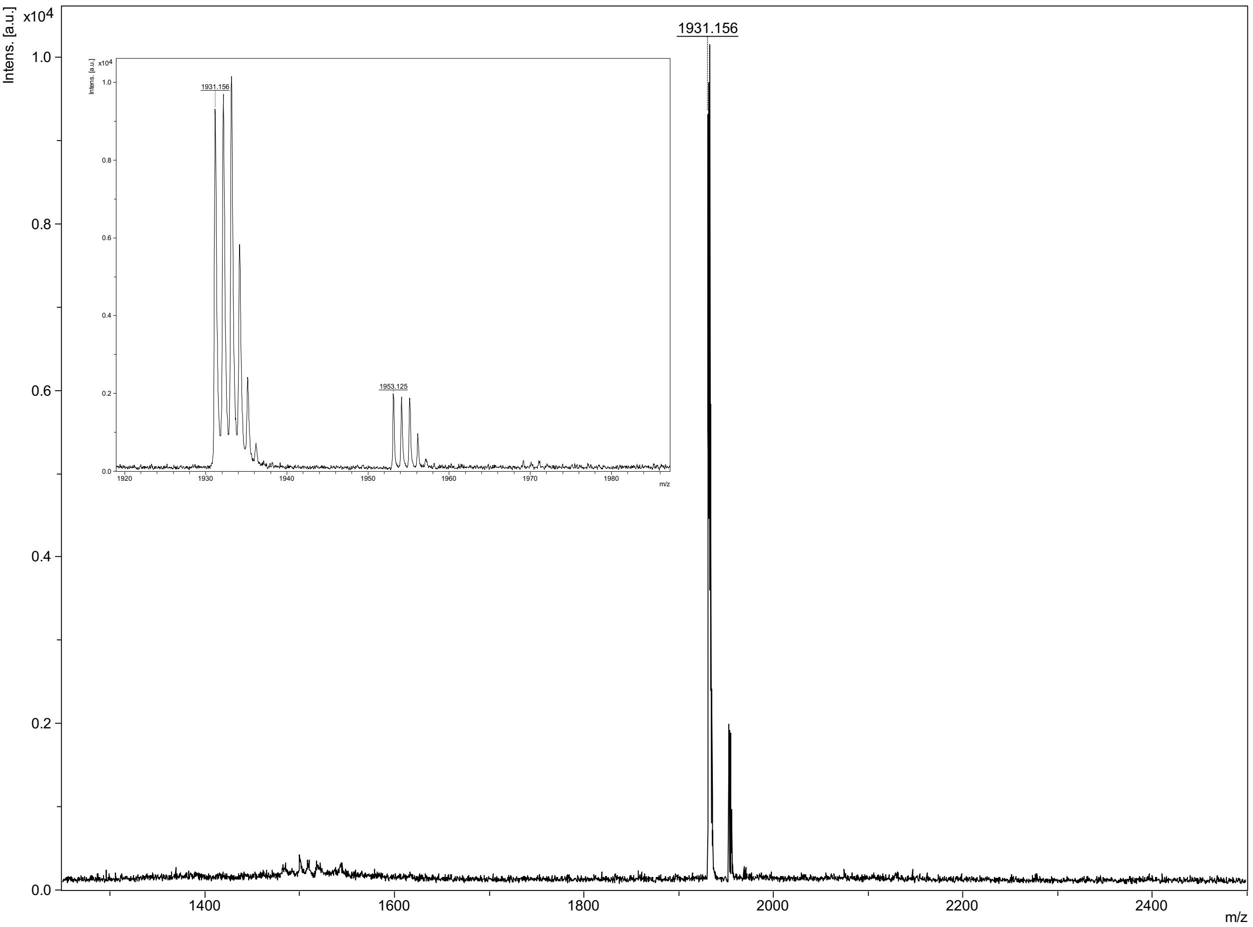

x10^4
Intens. [a.u.]
1931.156
1953.125
m/z

X3

Sequence: 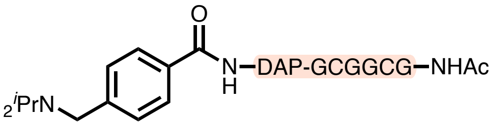

Chemical Formula: $C_{83}H_{108}N_{42}O_{21}$, Exact Mass: 2028.86

LC-MS (ESI) RT = 1.12 min, m/z found: 1016.00 $[M+2H]^{2+}$, 677.75 $[M+3H]^{3+}$; 1015.94 $[M+2H]^{2+}$, 677.63 $[M+3H]^{3+}$

MALDI-TOF m/z found 2030.39 $[M+H]^{+}$, 2052.31 $[M+Na]^{+}$; calc. 2029.88 $[M+H]^{+}$, 2051.86 $[M+Na]^{+}$

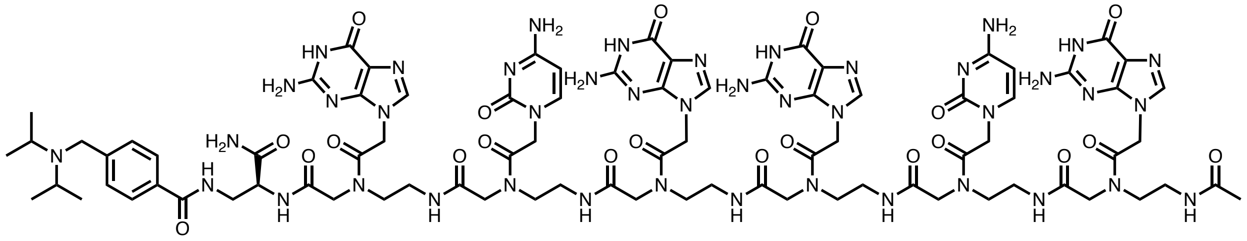

m/z: 2028.87 (100.0%), 2029.87 (91.8%), 2030.87 (59.1%)

ak_4_34_x8_aftercl_h2o 19/06/2023 23:10:43

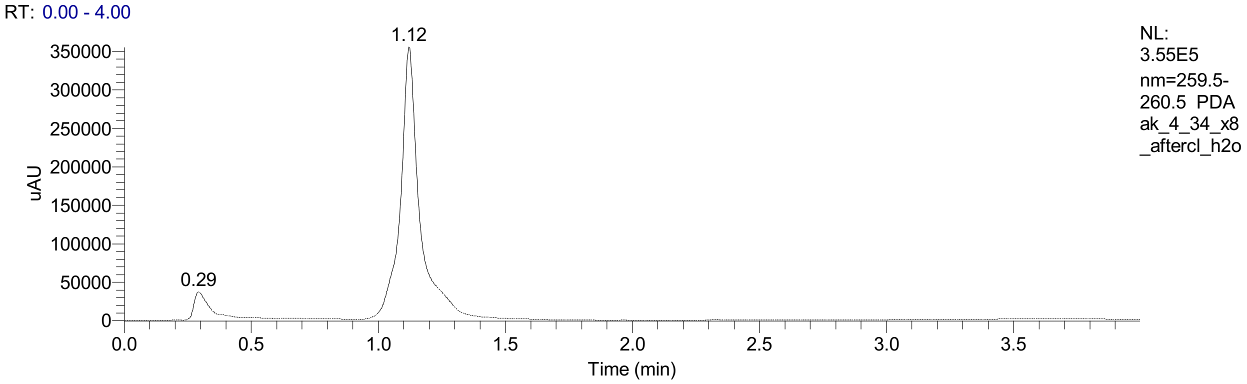

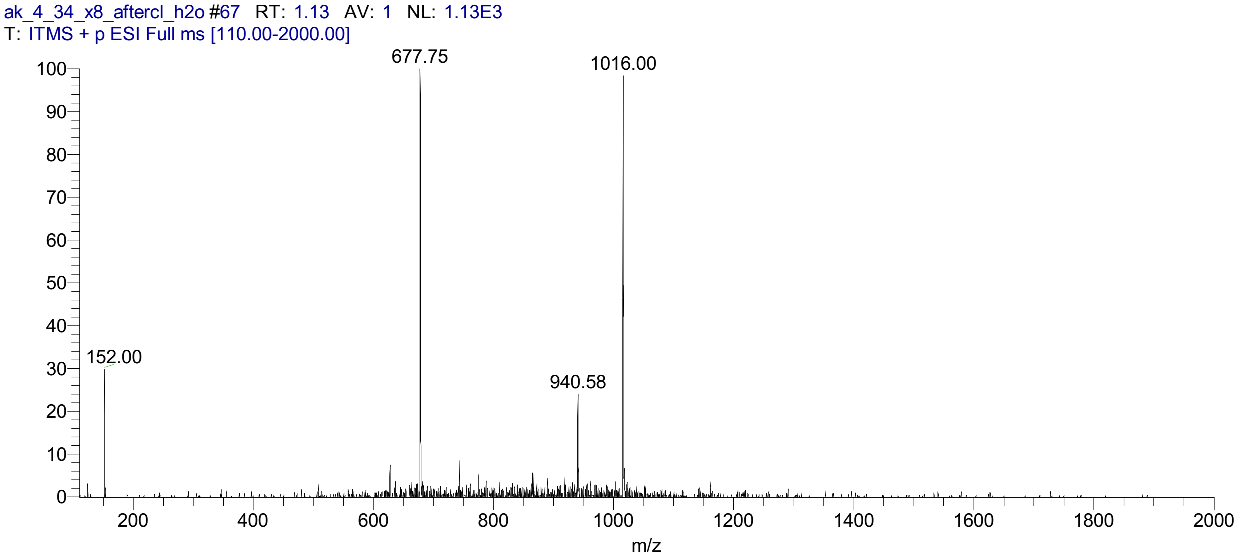

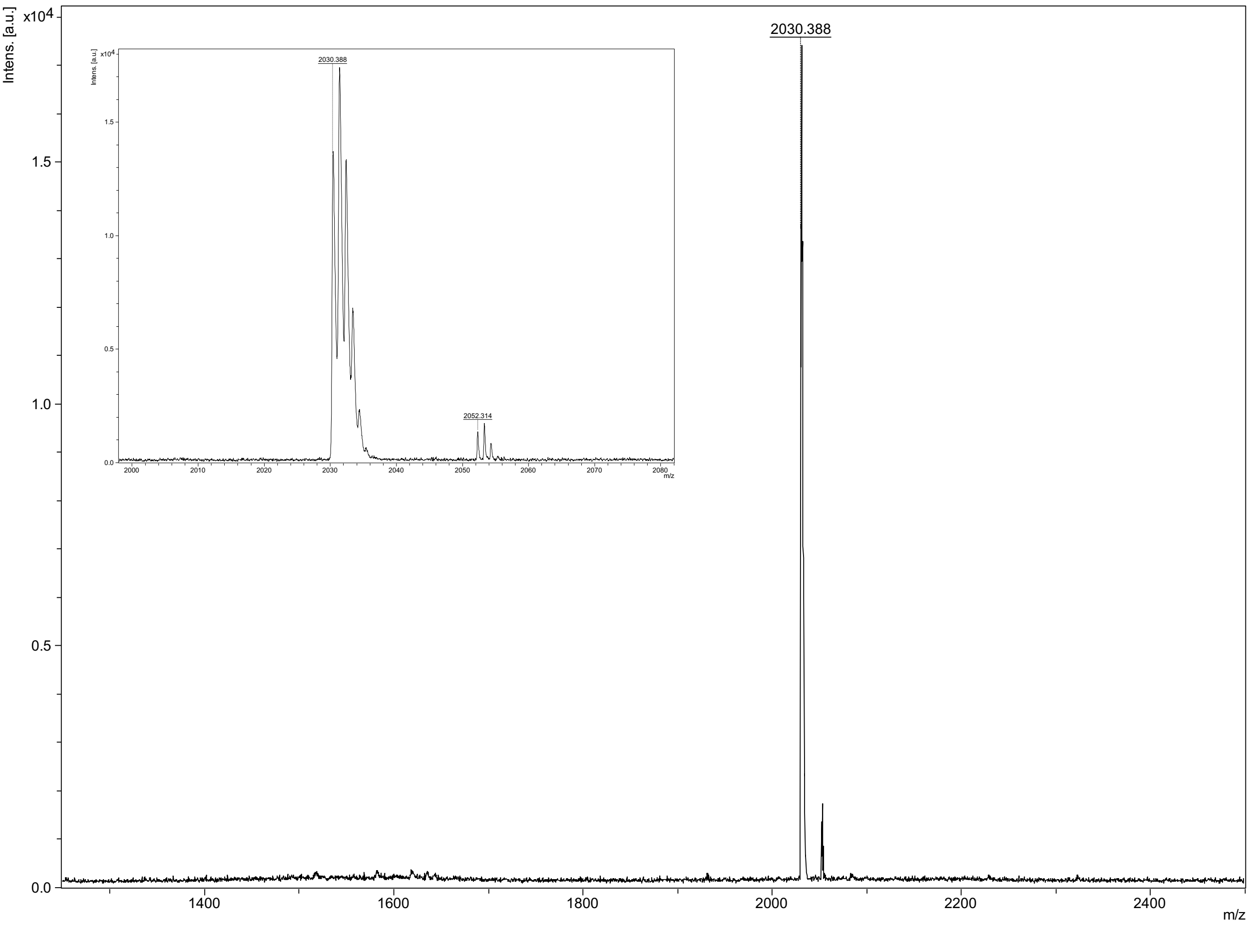

Intens. [a.u.]
x10^4
1.5
1.0
0.5
0.0
2030.388
1400
1600
1800
2000
2200
2400
m/z
2052.314

X4

Sequence: 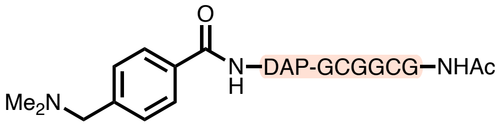

Chemical Formula: $C_{79}H_{100}N_{42}O_{21}$, Exact Mass: 1972.8048

LC-MS (ESI) RT = 1.05 min, m/z found: 988.00 $[M+2H]^{2+}$, 658.92 $[M+3H]^{3+}$; 987.91 $[M+2H]^{2+}$, 658.94 $[M+3H]^{3+}$

MALDI-TOF m/z found 1974.81 $[M+H]^{+}$, 1996.60 $[M+Na]^{+}$; calc. 1973.81 $[M+H]^{+}$, 1995.79 $[M+Na]^{+}$

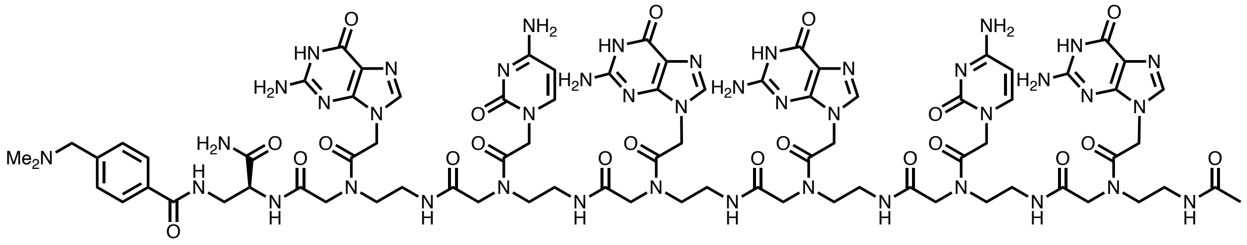

m/z: 1972.80 (100.0%), 1973.81 (87.4%), 1974.81 (55.6%), 1975.81 (20.2%)

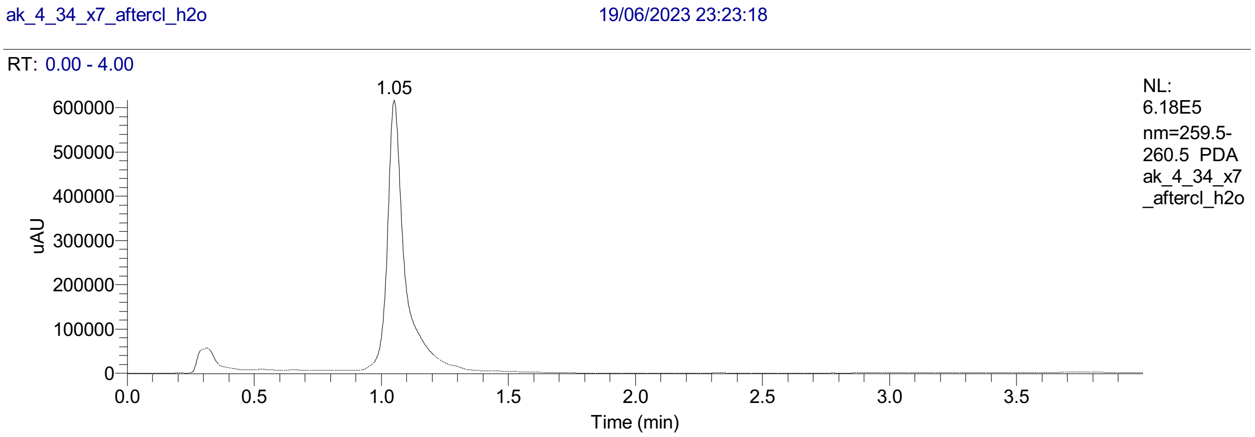

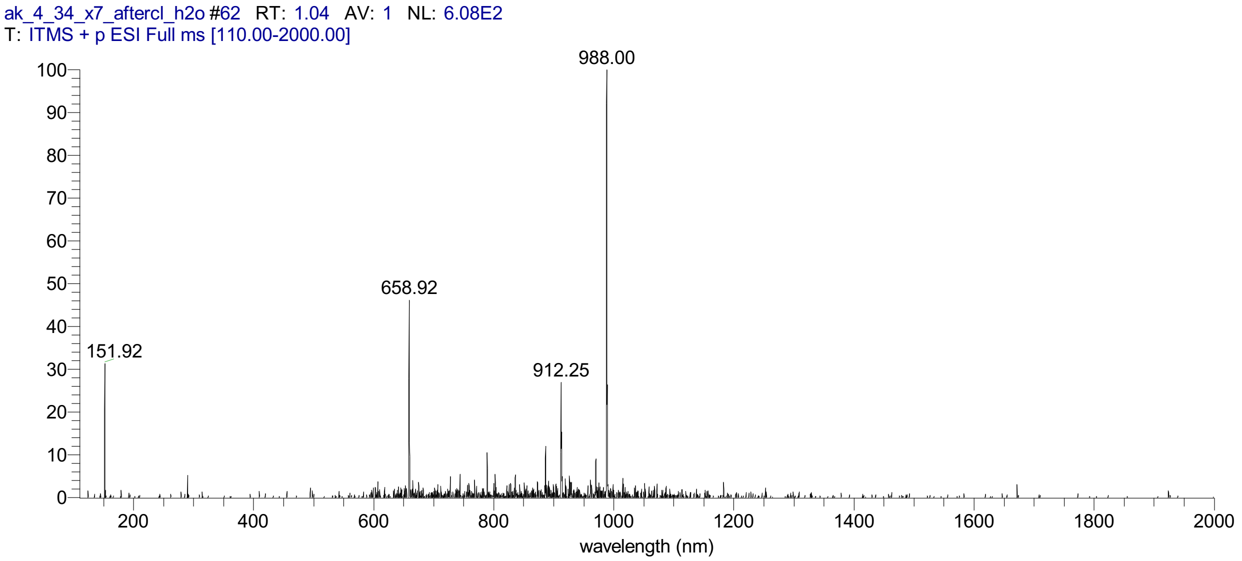

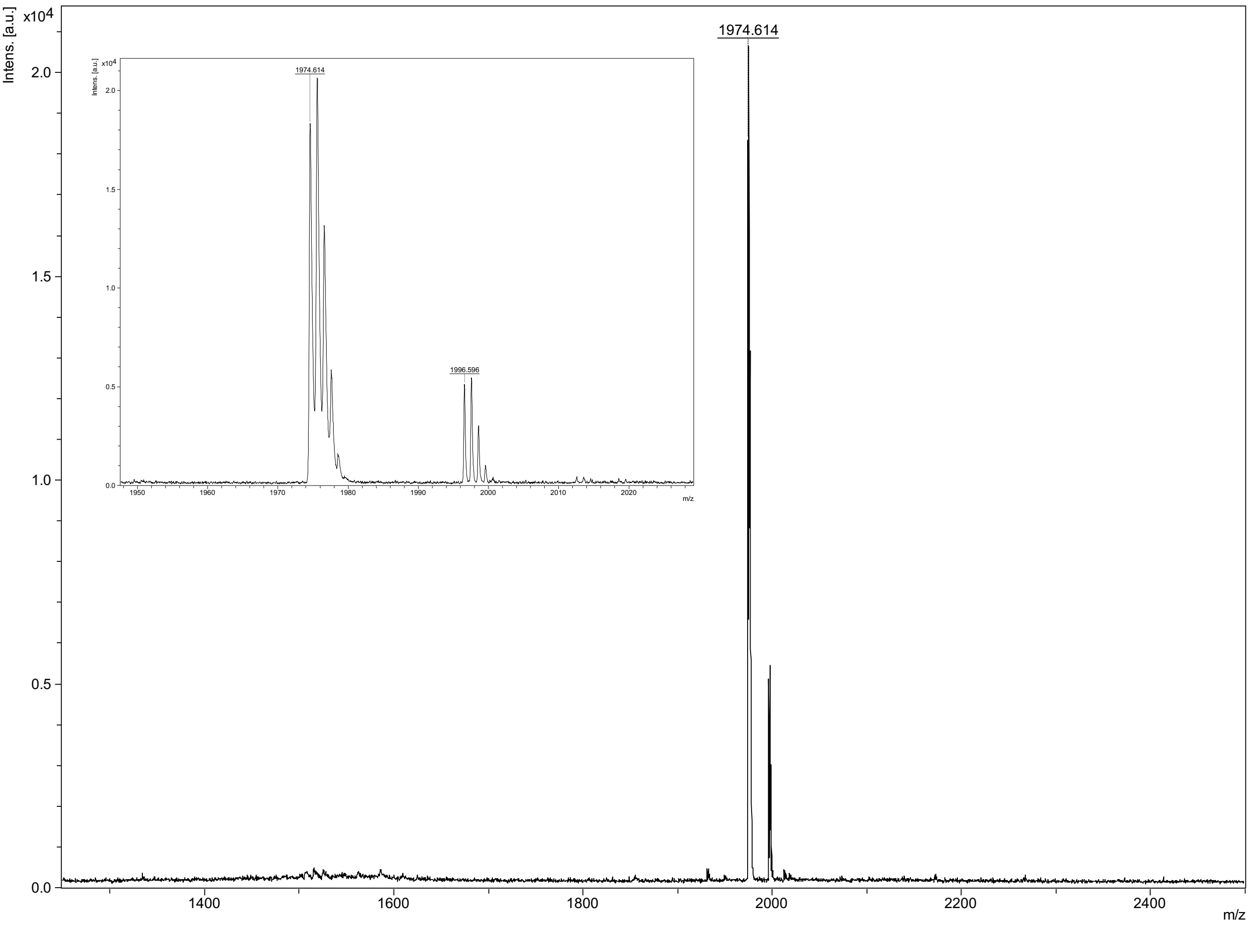

x10^4
Intens. [a.u.]
2.0
1.5
1.0
0.5
0.0
1974.614
1996.596
1400
1600
1800
2000
2200
2400
1950
1960
1970
1980
1990
2000
2010
2020
m/z

X5

Sequence: 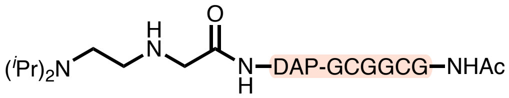

Chemical Formula: $C_{79}H_{109}N_{43}O_{21}$ Exact Mass: 1995.88

LC-MS (ESI) RT = 1.02 min, m/z found: 999.25 $[M+2H]^{2+}$, 666.75 $[M+3H]^{3+}$; calc. 999.44 $[M+2H]^{2+}$, 666.63 $[M+3H]^{3+}$

MALDI-TOF m/z found 1997.34 $[M+H]^{+}$, 2019.35 $[M+Na]^{+}$; calc. 1996.89 $[M+H]^{+}$, 2018.87 $[M+Na]^{+}$

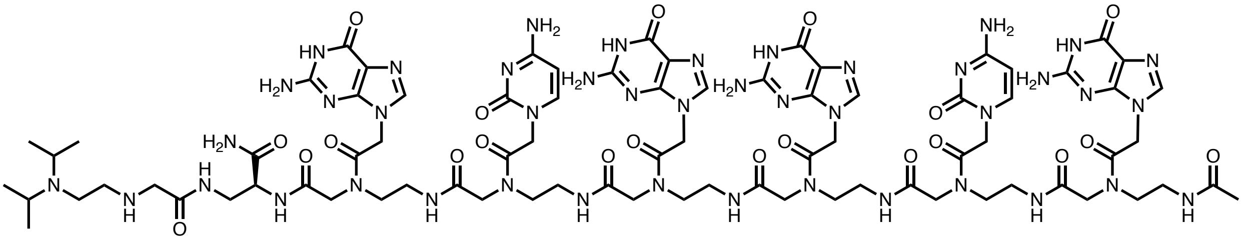

m/z: 1996.88 (100.0%), 1995.88 (96.7%), 1997.89 (36.6%)

ak_4_37_pr_aftercl_230619155413 19/06/2023 15:54:13

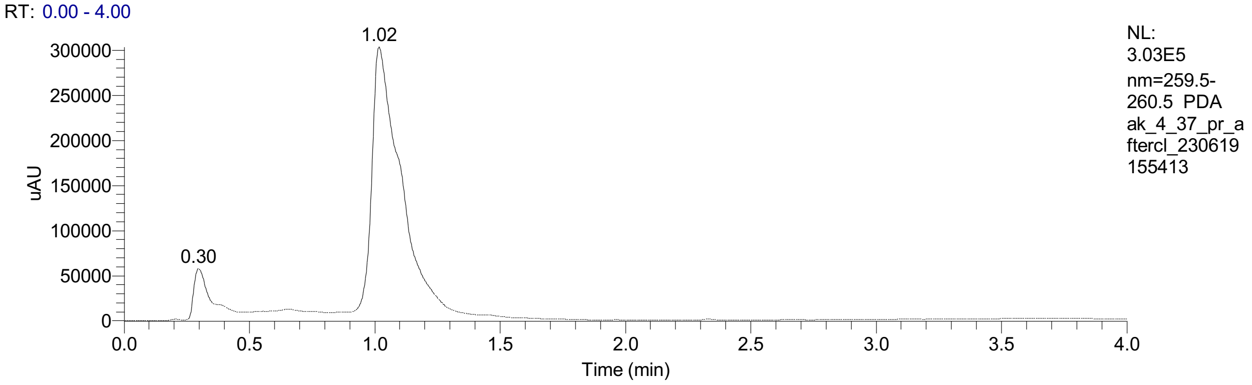

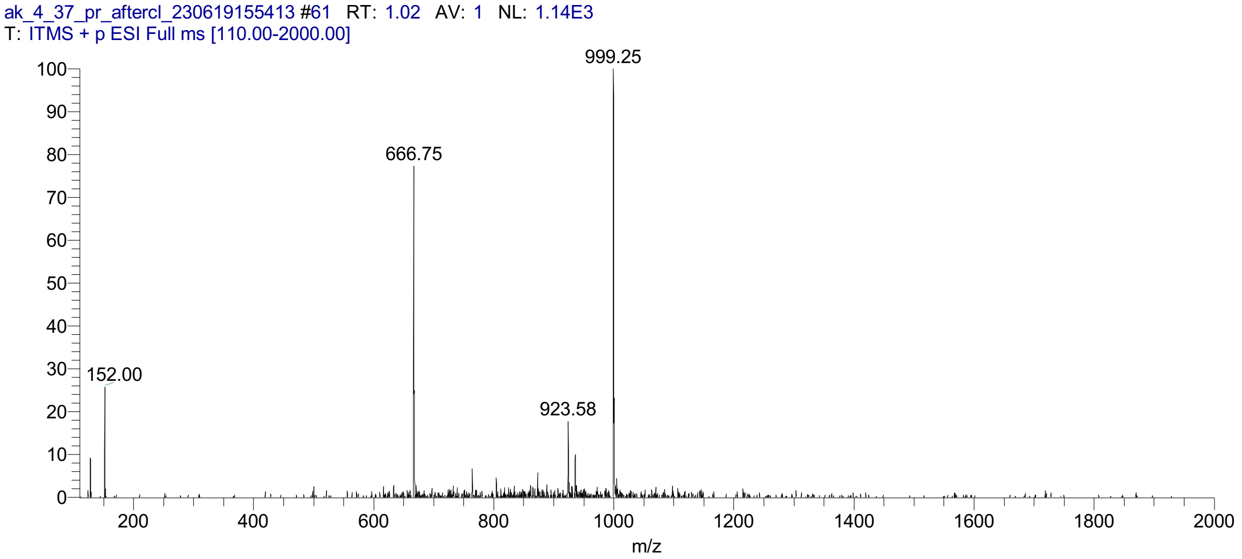

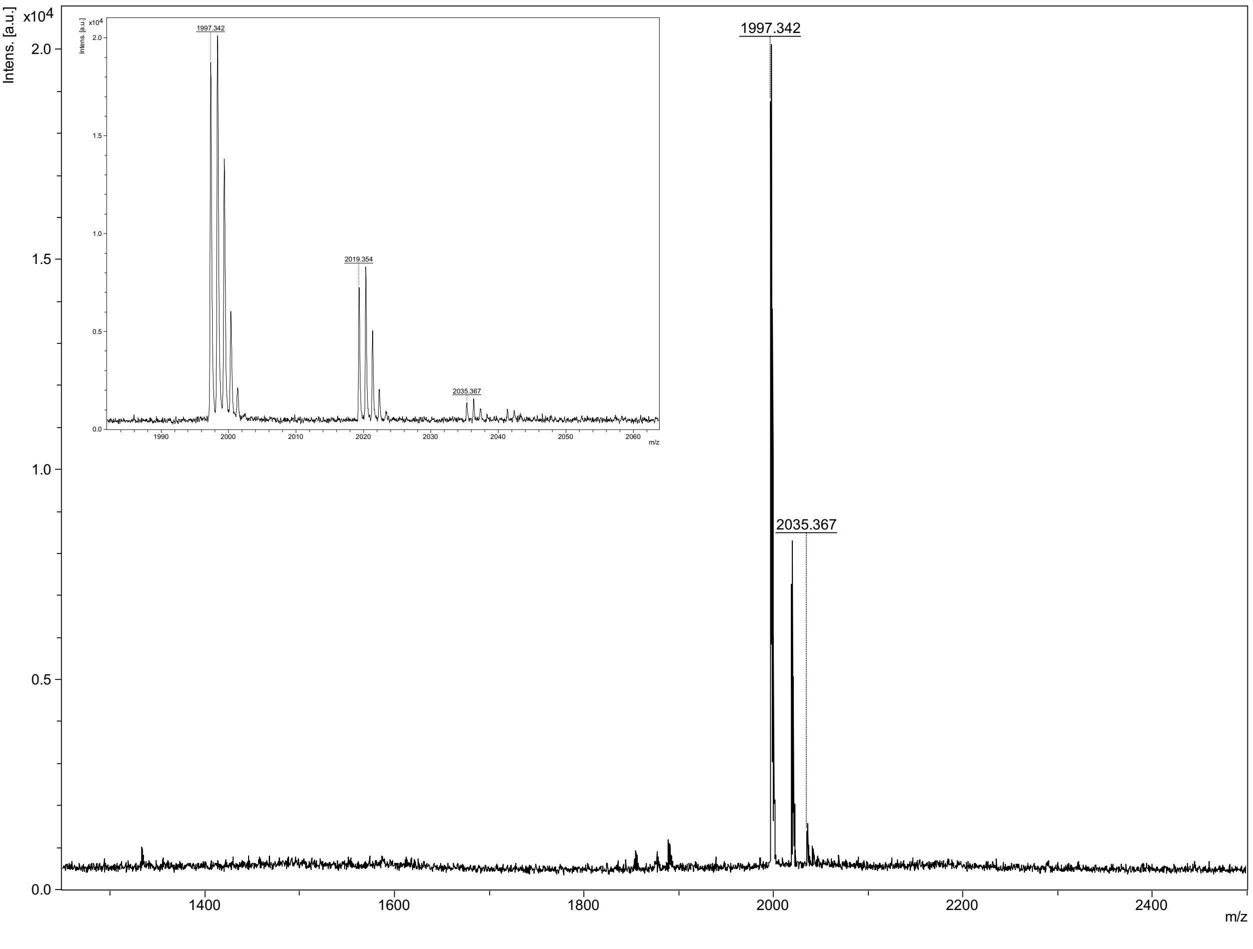

x10^4
Intens. [a.u.]
2.0
1.5
1.0
0.5
0.0
1997.342
2035.367
1400
1600
1800
2000
2200
2400
m/z
1997.342
2019.354
2035.367
1990
2000
2010
2020
2030
2040
2050
2060

X6

Sequence: 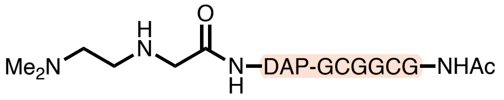

Chemical Formula: $C_{75}H_{101}N_{43}O_{21}$ Exact Mass: 1939.82

LC-MS (ESI) RT = 1.07 min, m/z found: 971.50 $[M+2H]^{2+}$, 647.83 $[M+3H]^{3+}$; calc. 970.92 $[M+2H]^{2+}$, 647.61 $[M+3H]^{3+}$

MALDI-TOF m/z found 1941.21 $[M+H]^{+}$, 1963.24 $[M+Na]^{+}$; calc. 1940.82 $[M+H]^{+}$, 1962.80 $[M+Na]^{+}$

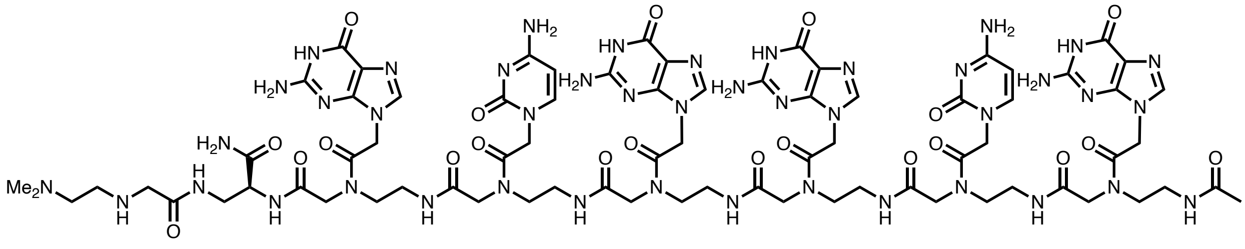

m/z: 1939.82 (100.0%), 1940.82 (83.1%), 1941.82 (50.6%)

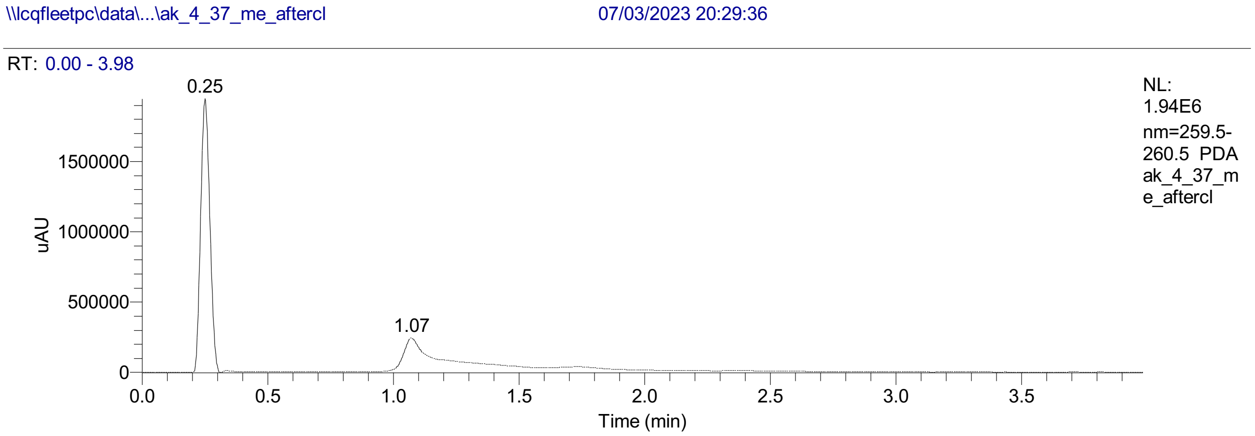

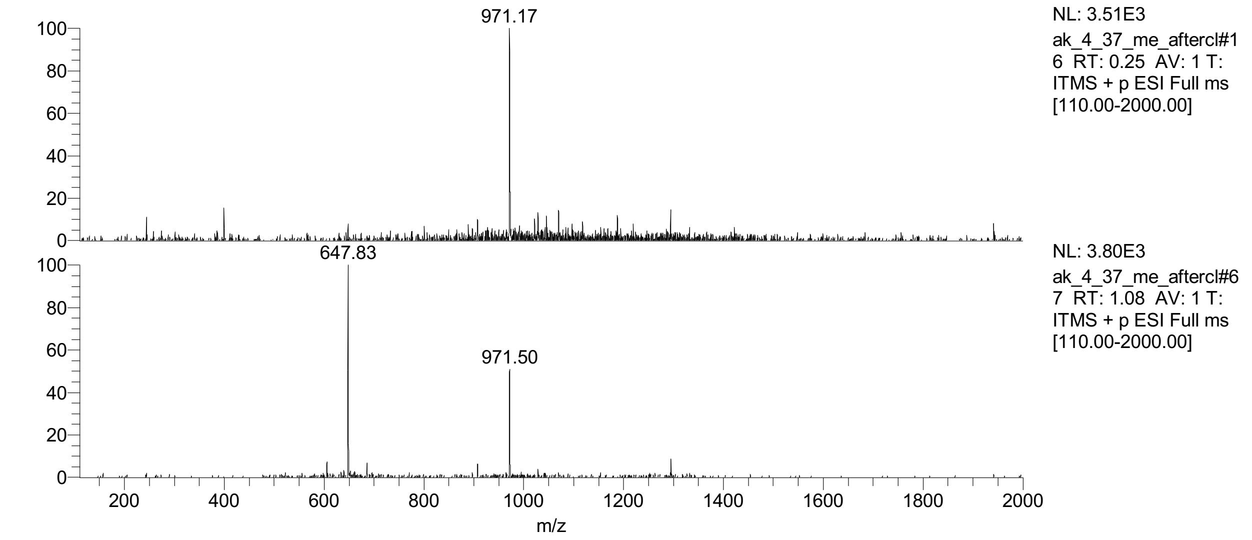

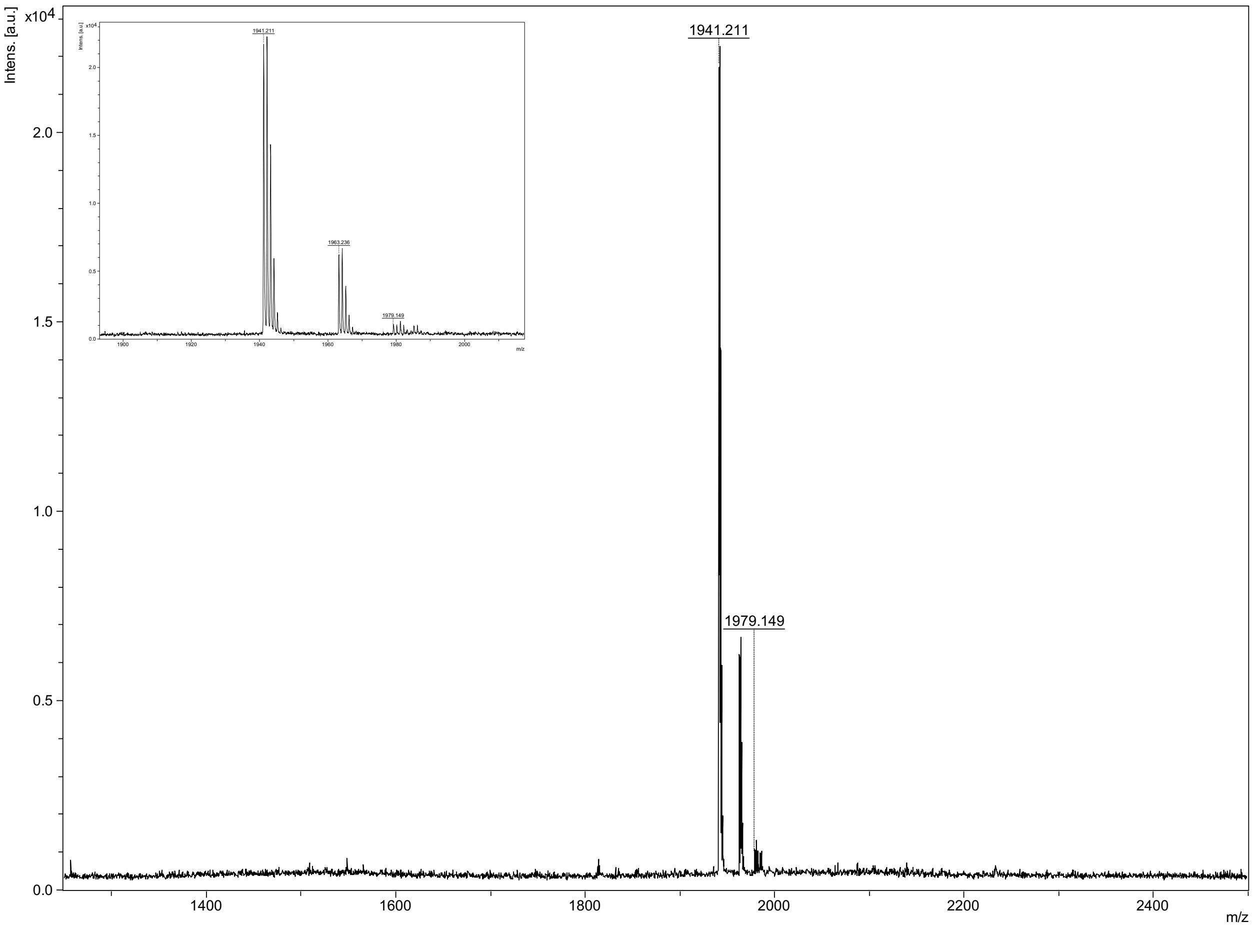

Intens. [a.u.]
x10^4
1941.211
1979.149
1963.236
m/z

X7

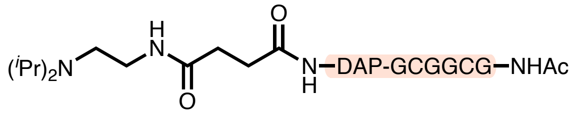

Sequence:

Chemical Formula: C81H111N43O22, Exact Mass: 2037.8889, LC-MS (ESI) RT = 1.08 min, m/z found: 1020.17 $[M+2H]^{2+}$, 680.75 $[M+3H]^{3+}$;

MALDI-TOF m/z found 2039.331 $[M+H]^{+}$, 2061.337 $[M+Na]^{+}$

Calc. Monoisotopic Mass: 2038.8961 $[M+H]^{+}$, 2060.8781 $[M+Na]^{+}$

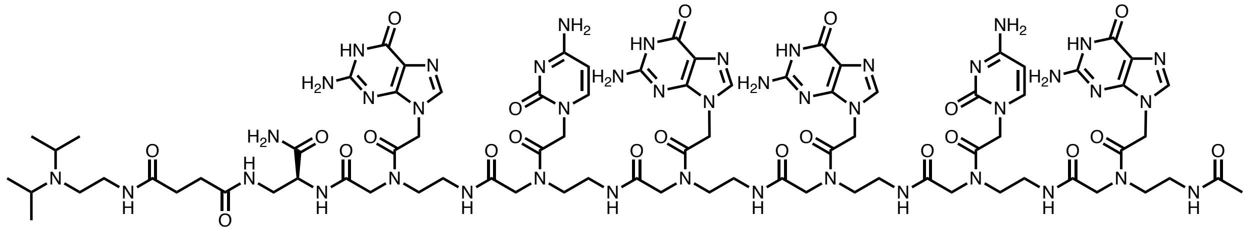

m/z: 2038.89 (100.0%), 2037.89 (95.9%), 2039.90 (38.1%)

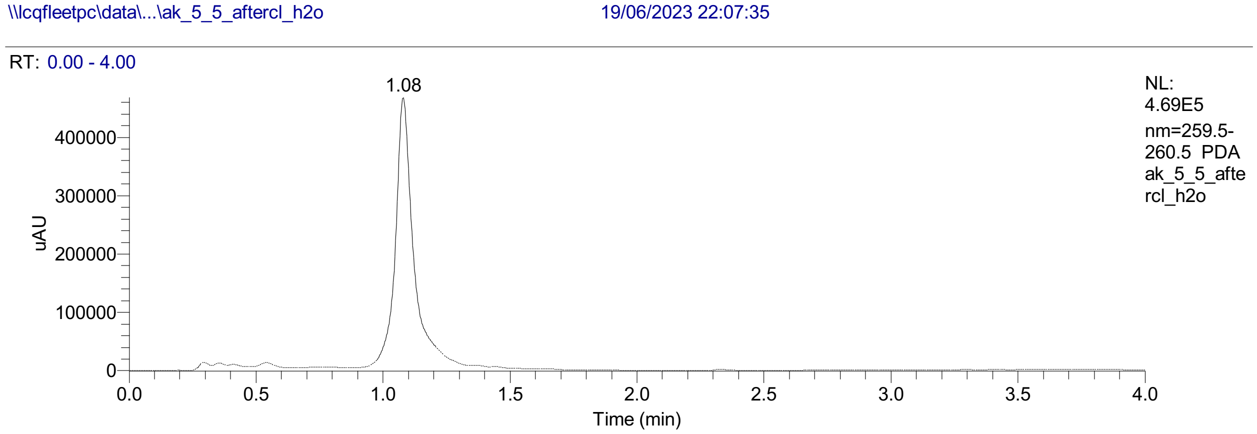

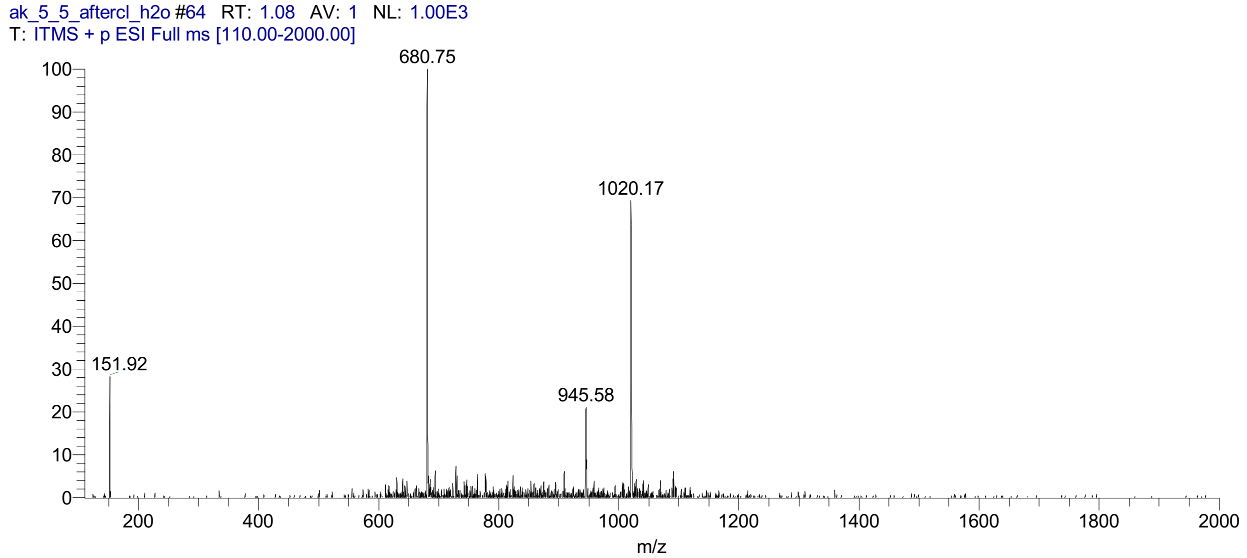

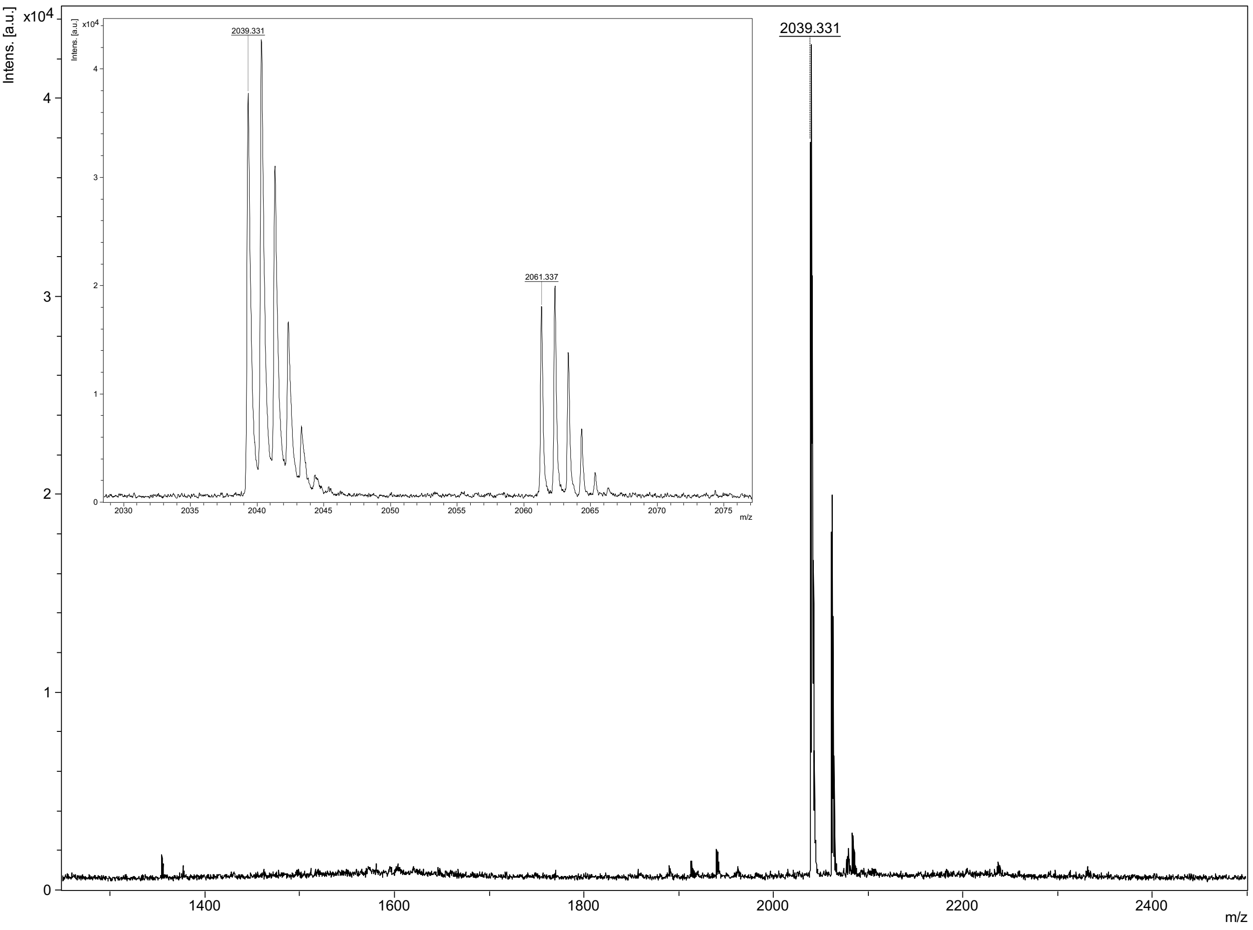

Intens. [a.u.]
x10^4
2039.331
2061.337
m/z

X8

Sequence: 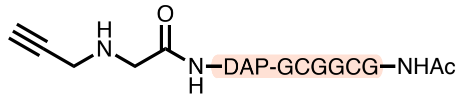

Chemical Formula: $C_{74}H_{94}N_{42}O_{21}$, Exact Mass: 1906.76

LC-MS (ESI) RT = 1.01 min, m/z found: 954.75 $[M+2H]^{2+}$, 637.17 $[M+3H]^{3+}$; calc. 954.38 $[M+2H]^{2+}$, 636.59 $[M+3H]^{3+}$

MALDI-TOF m/z found 1908.03 $[M+H]^{+}$, 1930.01 $[M+Na]^{+}$; calc. 1907.77 $[M+H]^{+}$, 1929.75 $[M+Na]^{+}$

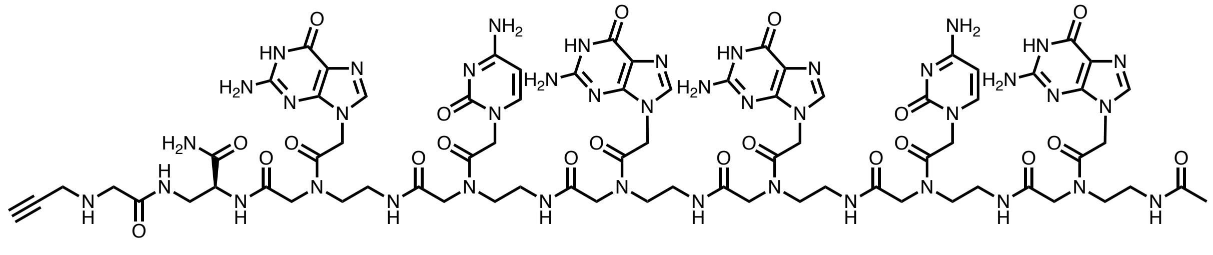

m/z: 1906.76 (100.0%), 1907.76 (81.9%), 1908.76 (48.6%)

ak_4_37_x5_aftercl_h2o 19/06/2023 22:32:55

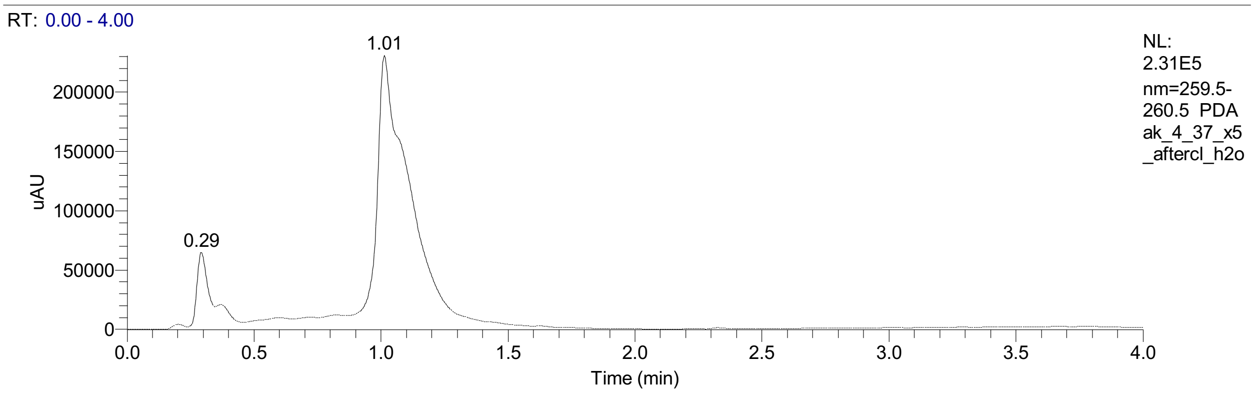

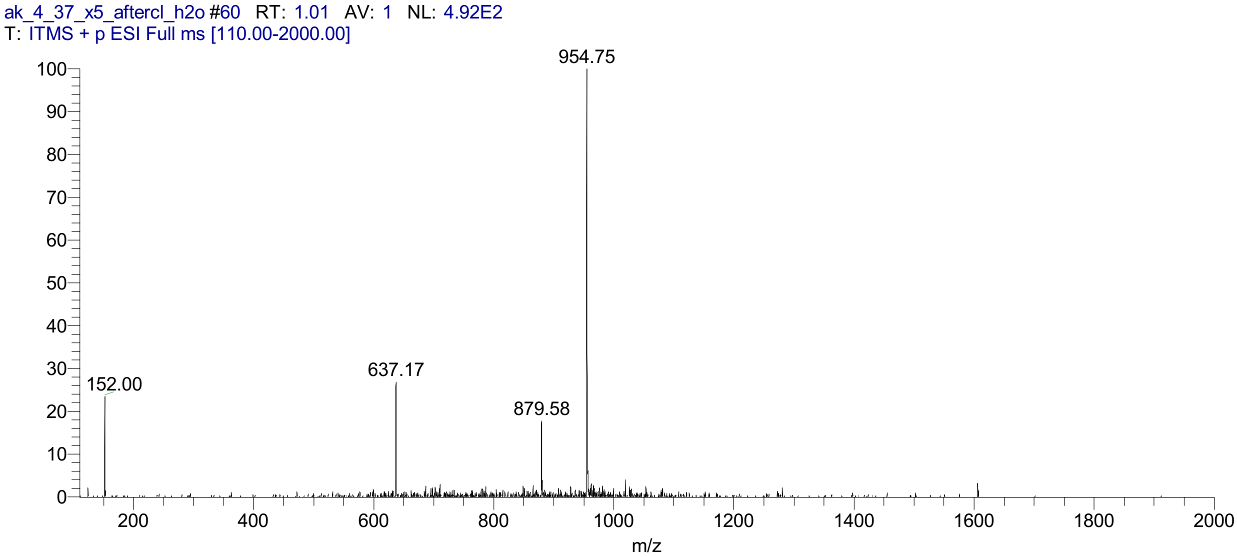

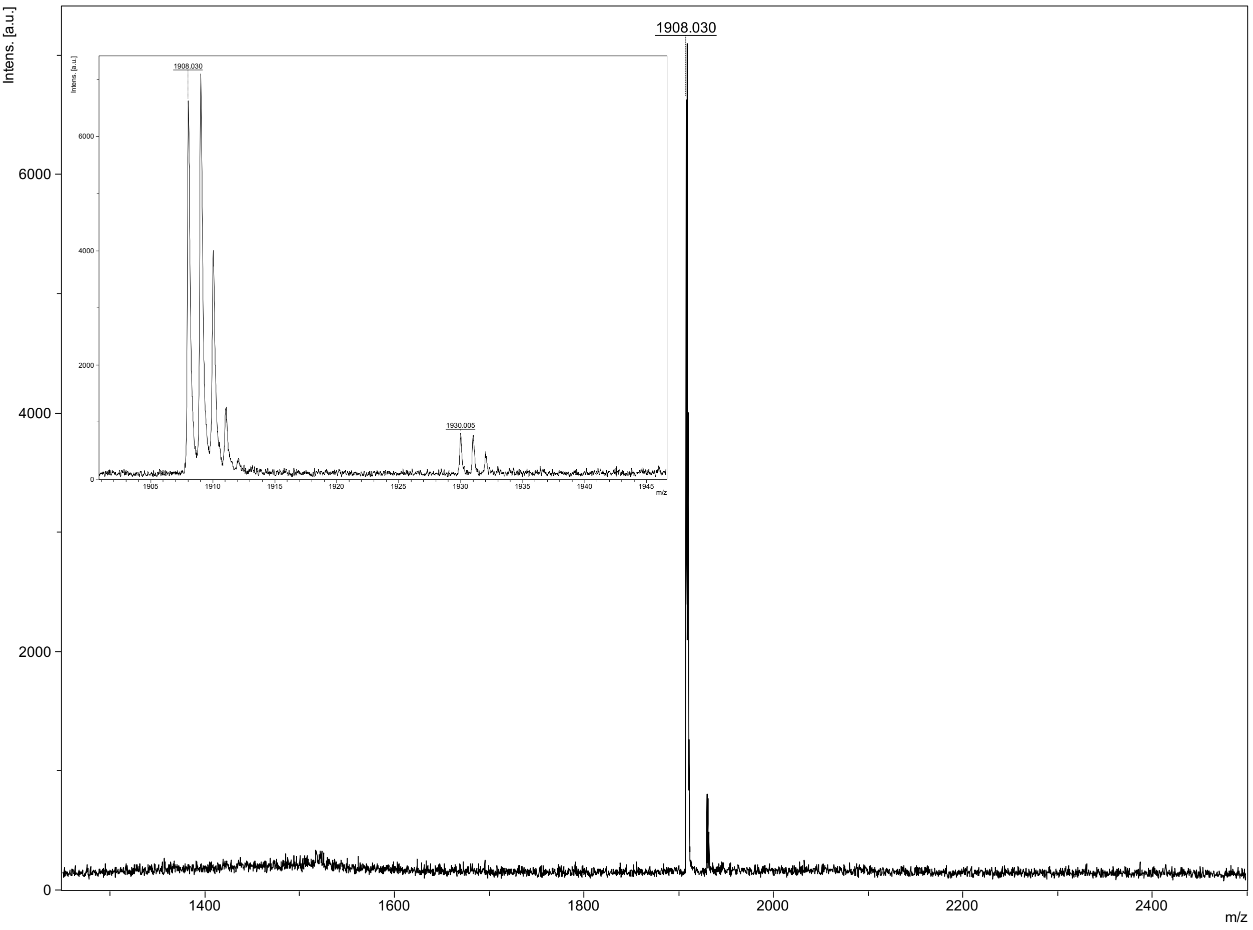

Intens. [a.u.]
1908.030
6000
4000
2000
0
1400
1600
1800
2000
2200
2400
m/z
1930.005
1905
1910
1915
1920
1925
1930
1935
1940
1945

X9

Sequence: 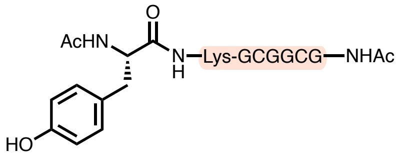

Chemical Formula: $C_{83}H_{106}N_{42}O_{23}$, Exact Mass: 2058.84

LC-MS (ESI) RT = 1.26 min, m/z found: 1030.75 $[M+2H]^{2+}$, 687.75 $[M+3H]^{3+}$; calc. 1030.93 $[M+2H]^{2+}$, 687.62 $[M+3H]^{3+}$

MALDI-TOF m/z found 2060.291 $[M+H]^{+}$, 2082.83 $[M+Na]^{+}$; calc. 2059.85 $[M+H]^{+}$, 2081.83 $[M+Na]^{+}$

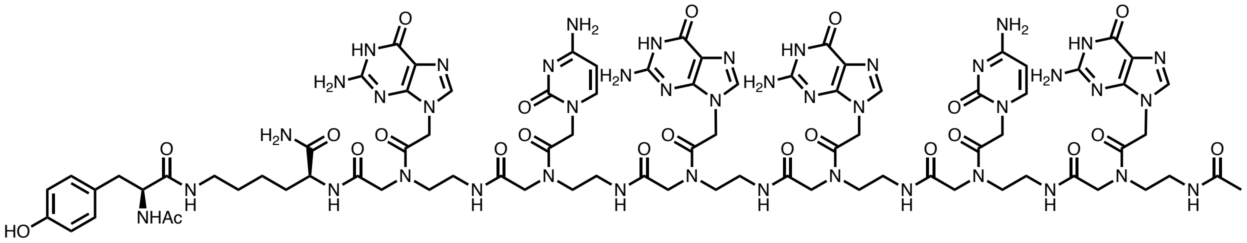

m/z: 2059.84 (100.0%), 2058.84 (95.0%), 2060.85 (44.1%)

AK_5_7_Tyr_aftercl_H2O 02/05/2023 21:09:08

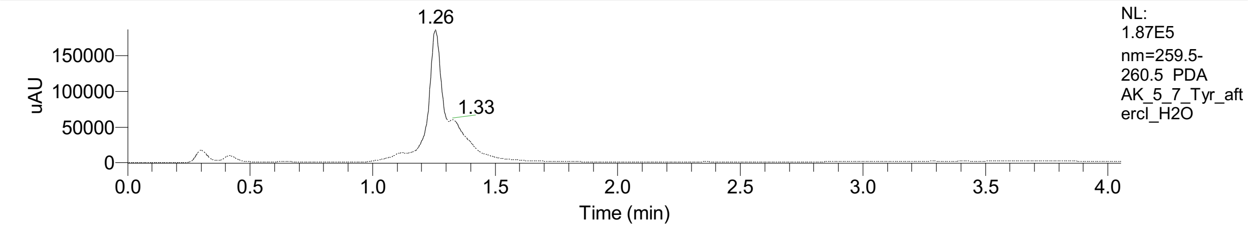

AK_5_7_Tyr_aftercl_H2O #75 RT: 1.27 AV: 1 NL: 7.20E2
T: ITMS + p ESI Full ms [110.00-2000.00]

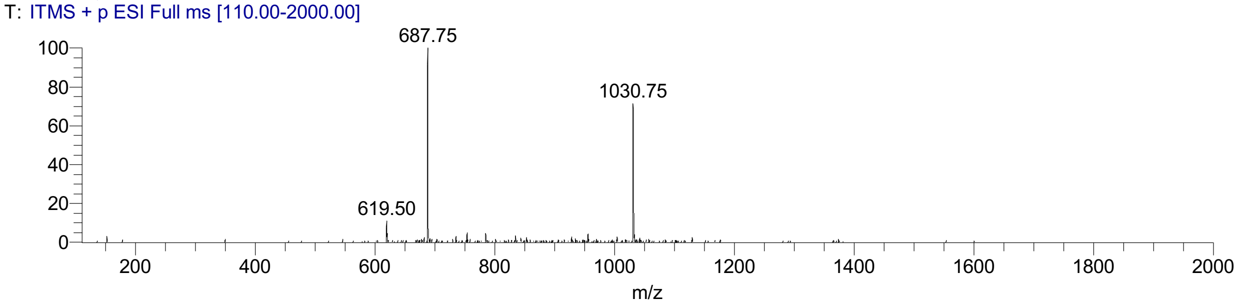

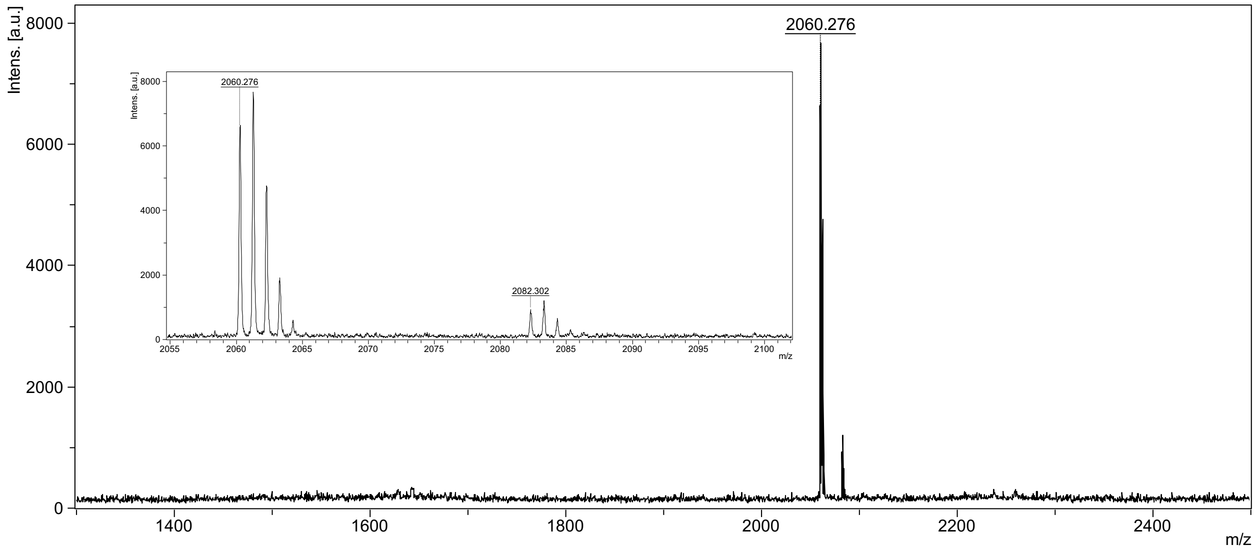

X10

Sequence: 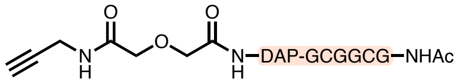

Chemical Formula: $C_{76}H_{96}N_{42}O_{23}$, Exact Mass: 1964.7633

LC-MS (ESI) RT = 1.12 min, m/z found: 983.75 $[M+2H]^{2+}$, 656.50 $[M+3H]^{3+}$; calc. 983.39 $[M+2H]^{2+}$, 655.93 $[M+3H]^{3+}$

MALDI-TOF m/z found 1965.91 $[M+H]^{+}$, 1987.90 $[M+Na]^{+}$; calc. 1965.77 $[M+H]^{+}$, 1987.75 $[M+Na]^{+}$

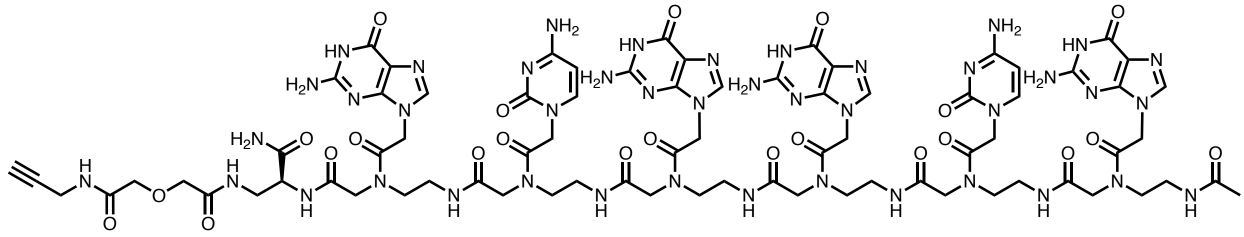

m/z: 1964.76 (100.0%), 1965.77 (84.2%), 1966.77 (39.9%)

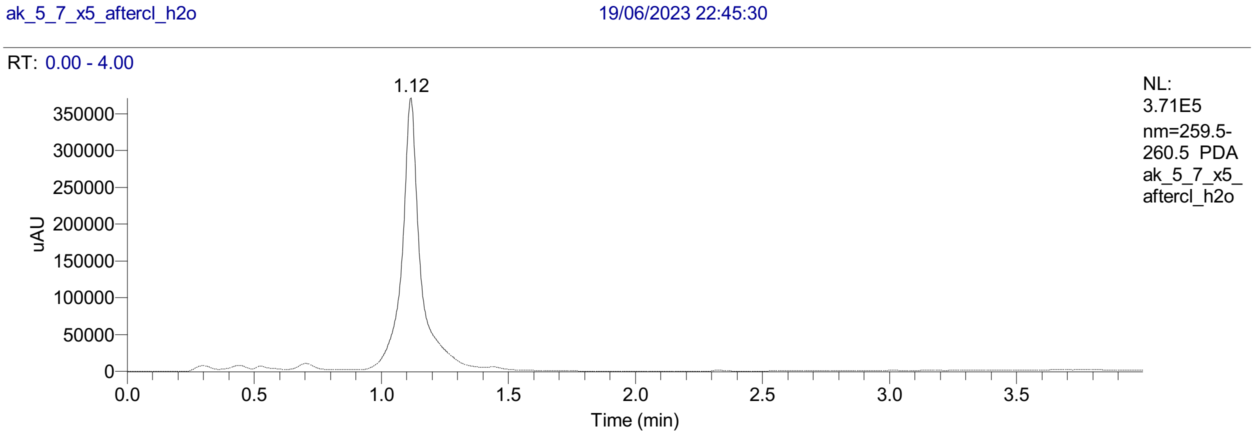

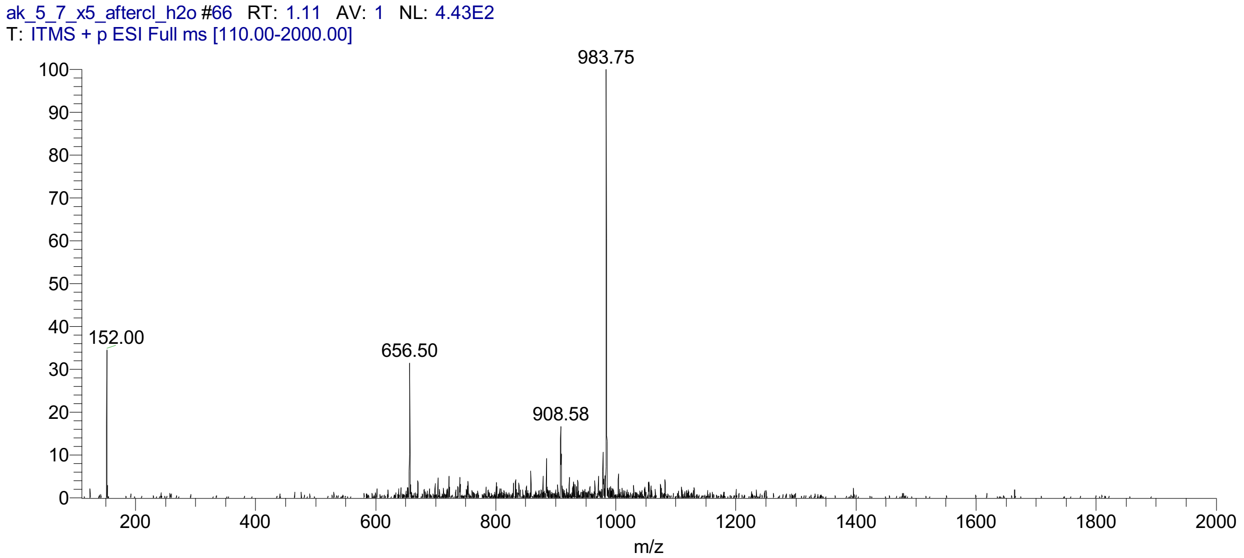

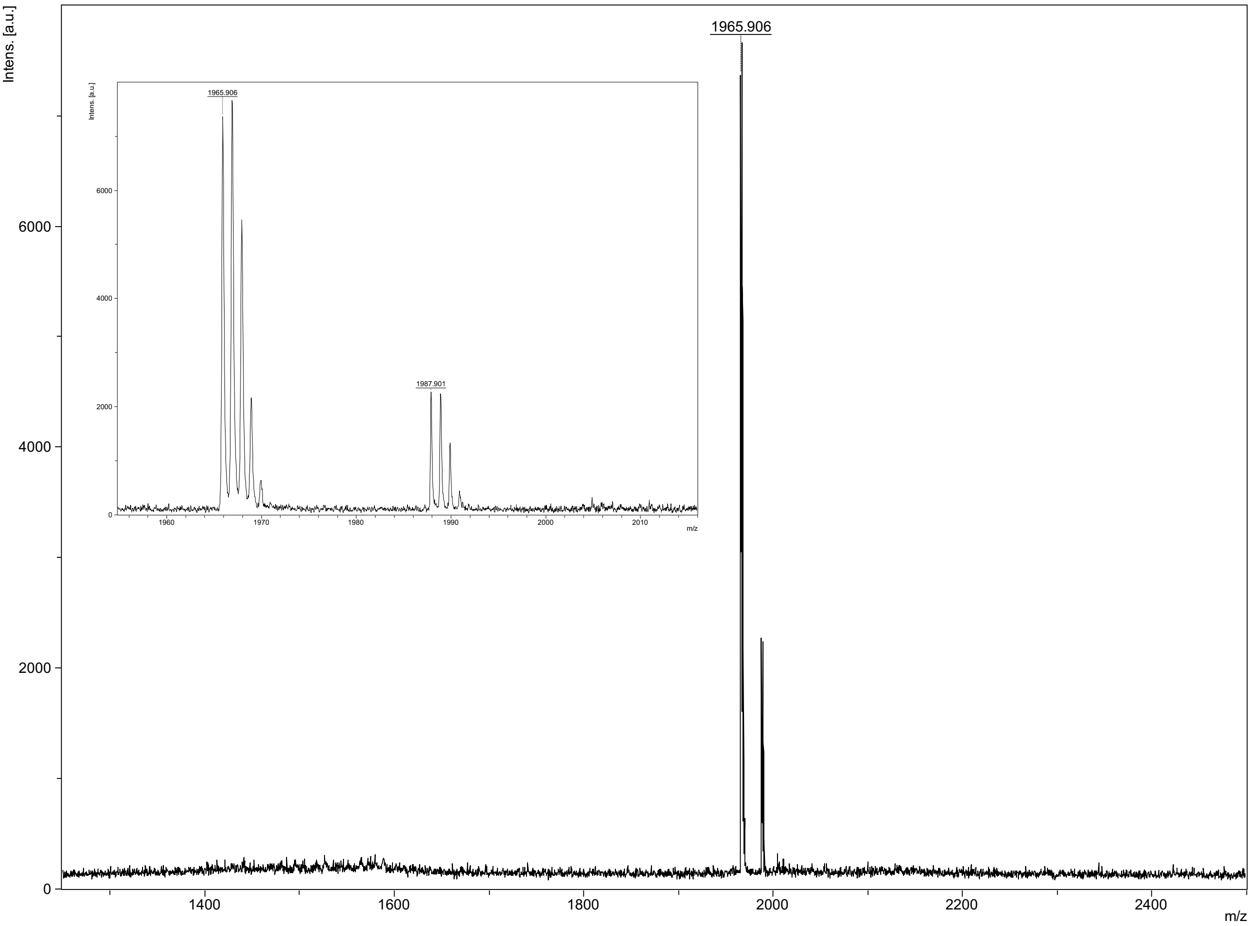

1965.906
Intens. [a.u.]
6000
4000
2000
0
1400
1600
1800
2000
2200
2400
m/z
1965.906
1987.901
1960
1970
1980
1990
2000
2010

X11

Sequence: 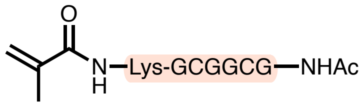

Chemical Formula: $C_{76}H_{99}N_{41}O_{21}$, Exact Mass: 1921.79

LC-MS (ESI) RT = 1.25 min, m/z found: 962.42 $[M+2H]^{2+}$, 642.00 $[M+3H]^{3+}$; 961.90 $[M+2H]^{2+}$, 641.61 $[M+3H]^{3+}$

MALDI-TOF m/z found 1923.48 $[M+H]^{+}$, 1945.48 $[M+Na]^{+}$; calc 1922.80 $[M+H]^{+}$, 1944.78 $[M+Na]^{+}$

AK_4_35_X6_aftercl_H2O 02/05/2023 19:09:20

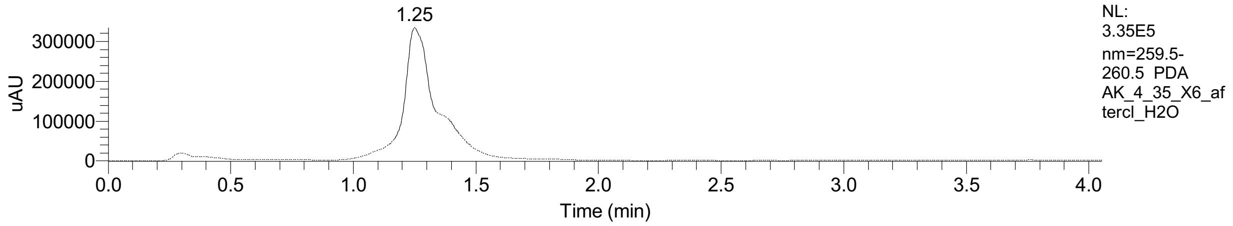

AK_4_35_X6_aftercl_H2O #74 RT: 1.25 AV: 1 NL: 1.10E3
T: ITMS + p ESI Full ms [110.00-2000.00]

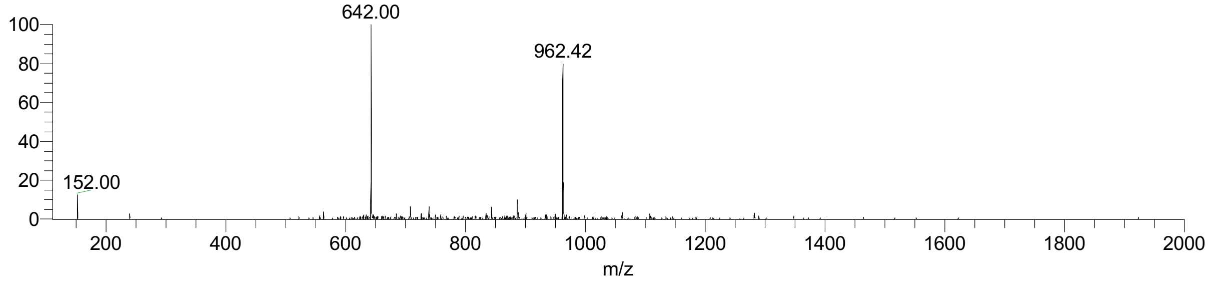

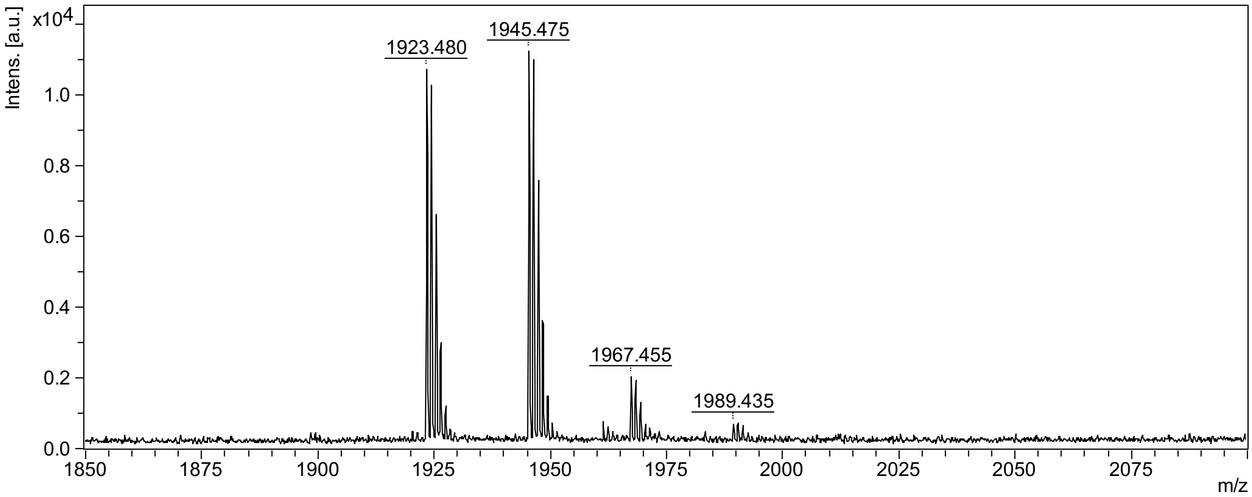

X12

Sequence: 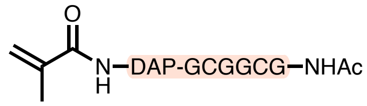

Chemical Formula: $C_{73}H_{93}N_{41}O_{21}$, Exact Mass: 1879.7470,

LC-MS (ESI) RT = 1.20 min, m/z found: 941.08 $[M+2H]^{2+}$, 628.08 $[M+3H]^{3+}$; calc. 940.88 $[M+2H]^{2+}$, 627.59 $[M+3H]^{3+}$

MALDI-TOF m/z found 1881.25 $[M+H]^{+}$, 1903.27 $[M+Na]^{+}$; calc. 1880.75 $[M+H]^{+}$, 1902.74 $[M+Na]^{+}$

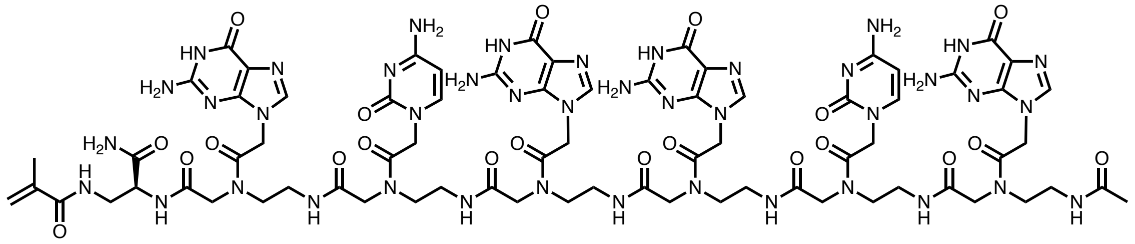

m/z: 1879.75 (100.0%), 1880.75 (80.8%), 1881.75 (47.9%)

AK_4_28_X6_aftercl_H2O 02/05/2023 19:03:01

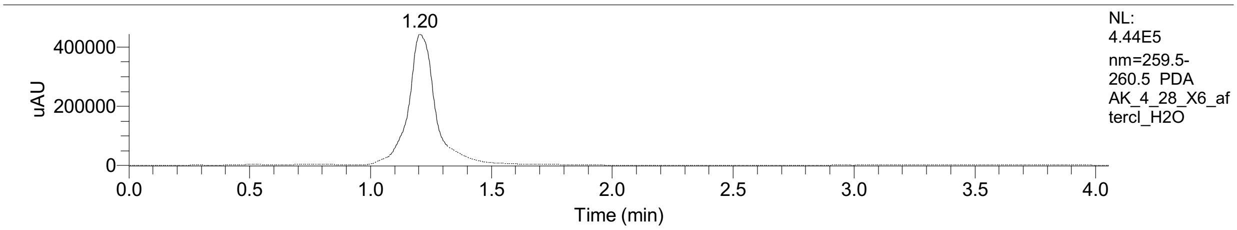

AK_4_28_X6_aftercl_H2O #71 RT: 1.20 AV: 1 NL: 7.75E2
T: ITMS + p ESI Full ms [110.00-2000.00]

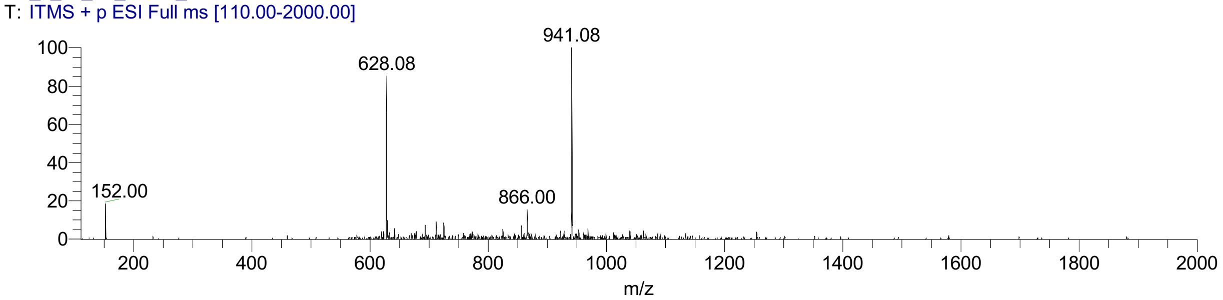

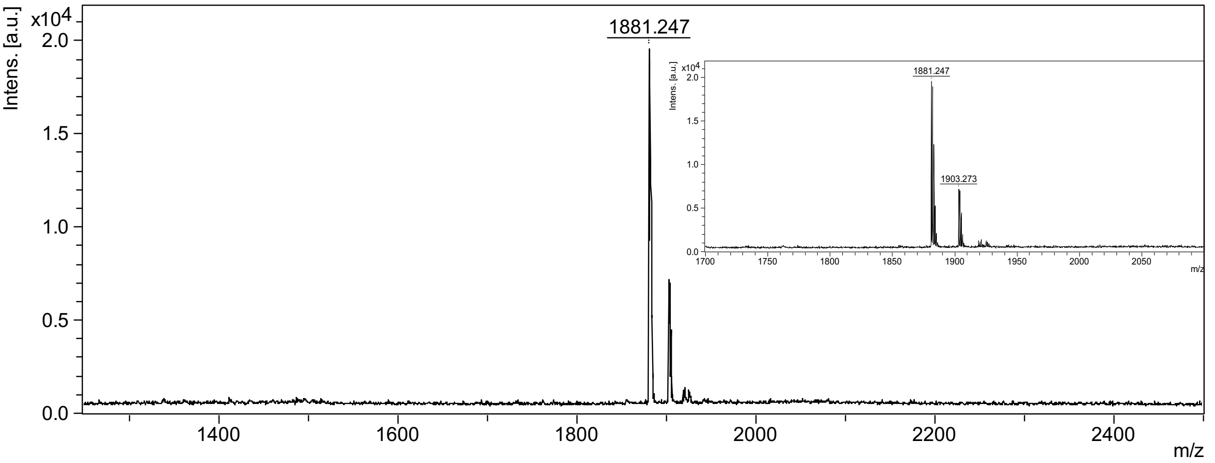

X13

Sequence: 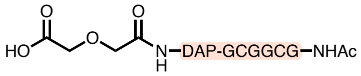

Chemical Formula: $C_{73}H_{93}N_{41}O_{24}$ Exact Mass: 1927.73,

LC-MS (ESI) RT = 1.13 min, m/z found: 965.17 $[M+2H]^{2+}$, 643.92 $[M+3H]^{3+}$; calc. 964.87$[M+2H]^{2+}$, 643.58 $[M+3H]^{3+}$

MALDI-TOF m/z found 1929.255 $[M+H]^{+}$, 1951.274 $[M+Na]^{+}$; calc. 1928.739 $[M+H]^{+}$, 1950.721 $[M+Na]^{+}$

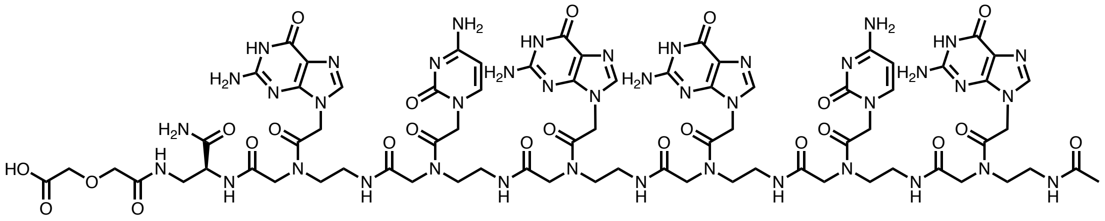

m/z: 1927.73 (100.0%), 1928.74 (80.9%), 1929.74 (37.4%)

AK_3_36_3_aftercl_H2o 02/05/2023 22:48:07

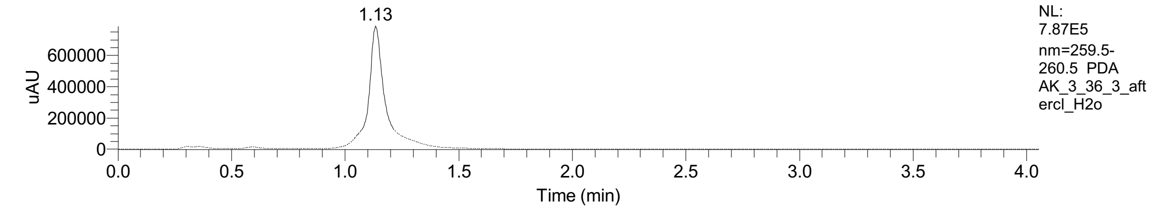

AK_3_36_3_aftercl_H2o #67 RT: 1.13 AV: 1 NL: 7.15E2
T: ITMS + p ESI Full ms [110.00-2000.00]

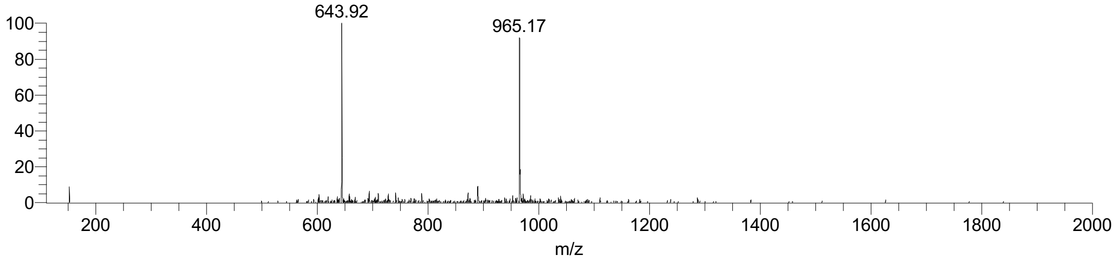

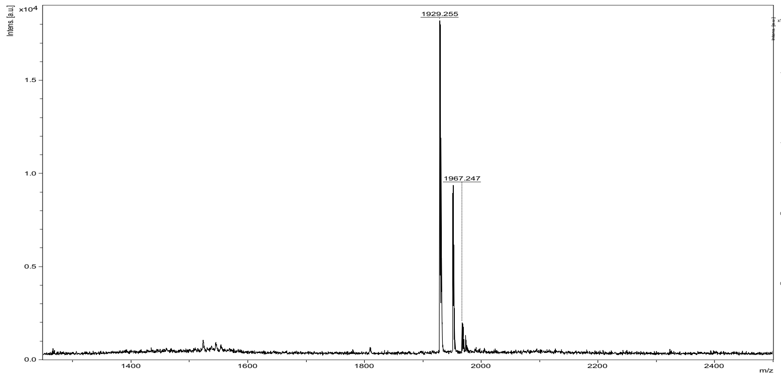

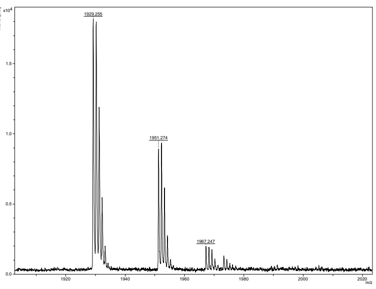

X14

Sequence: 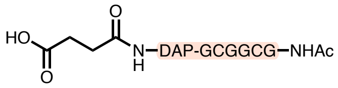

Chemical Formula: $C_{73}H_{93}N_{41}O_{23}$ Exact Mass: 1911.74

LC-MS (ESI) RT = 1.08 min, m/z found: 957.17 $[M+2H]^{2+}$, 638.67 $[M+3H]^{3+}$; calc. 956.88 $[M+2H]^{2+}$, 638.25 $[M+3H]^{3+}$

MALDI-TOF m/z found 1912.827 $[M+H]^{+}$, 1934.844 $[M+Na]^{+}$; calc. 1912.744 $[M+H]^{+}$, 1934.726 $[M+Na]^{+}$

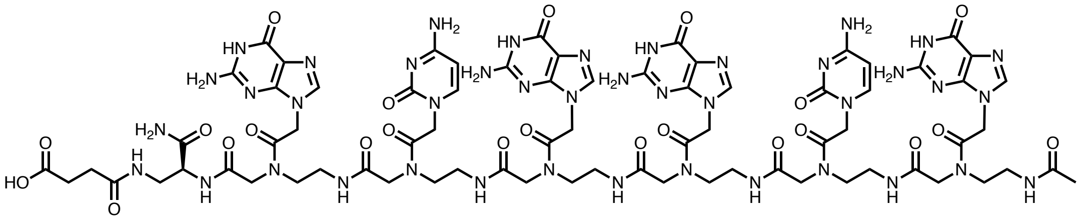

m/z: 1911.74 (100.0%), 1912.74 (80.9%), 1913.74 (48.4%)

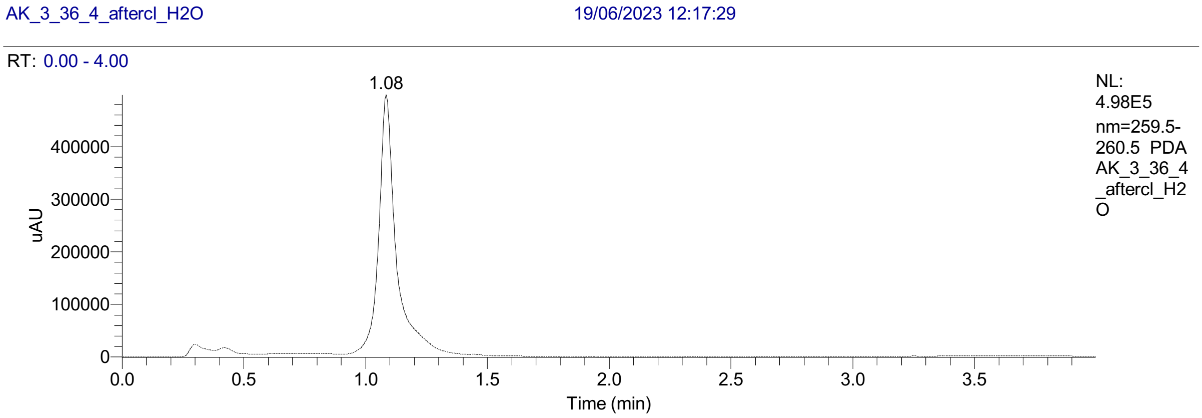

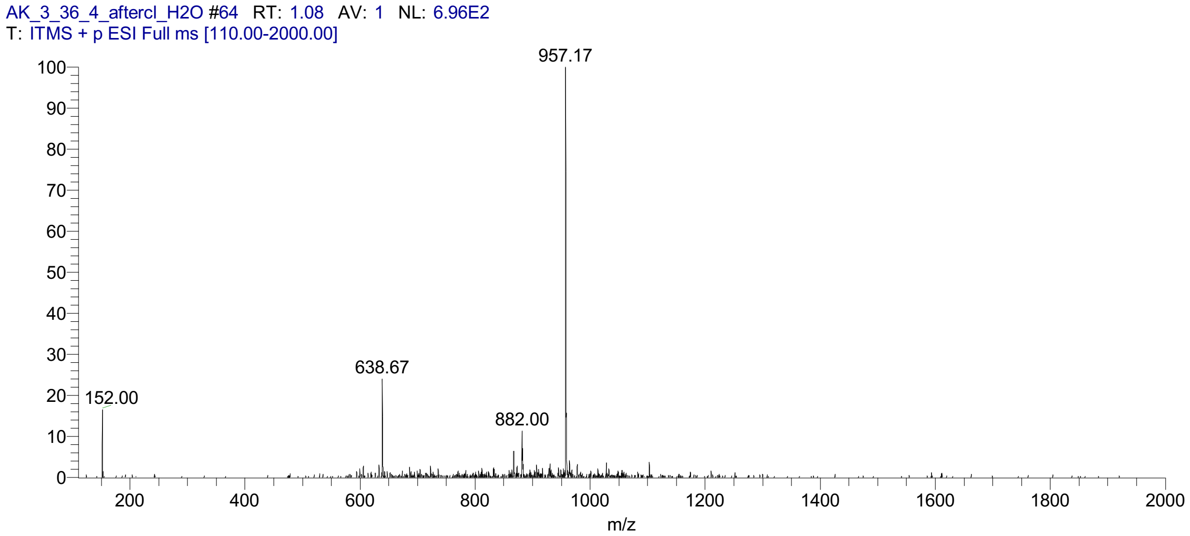

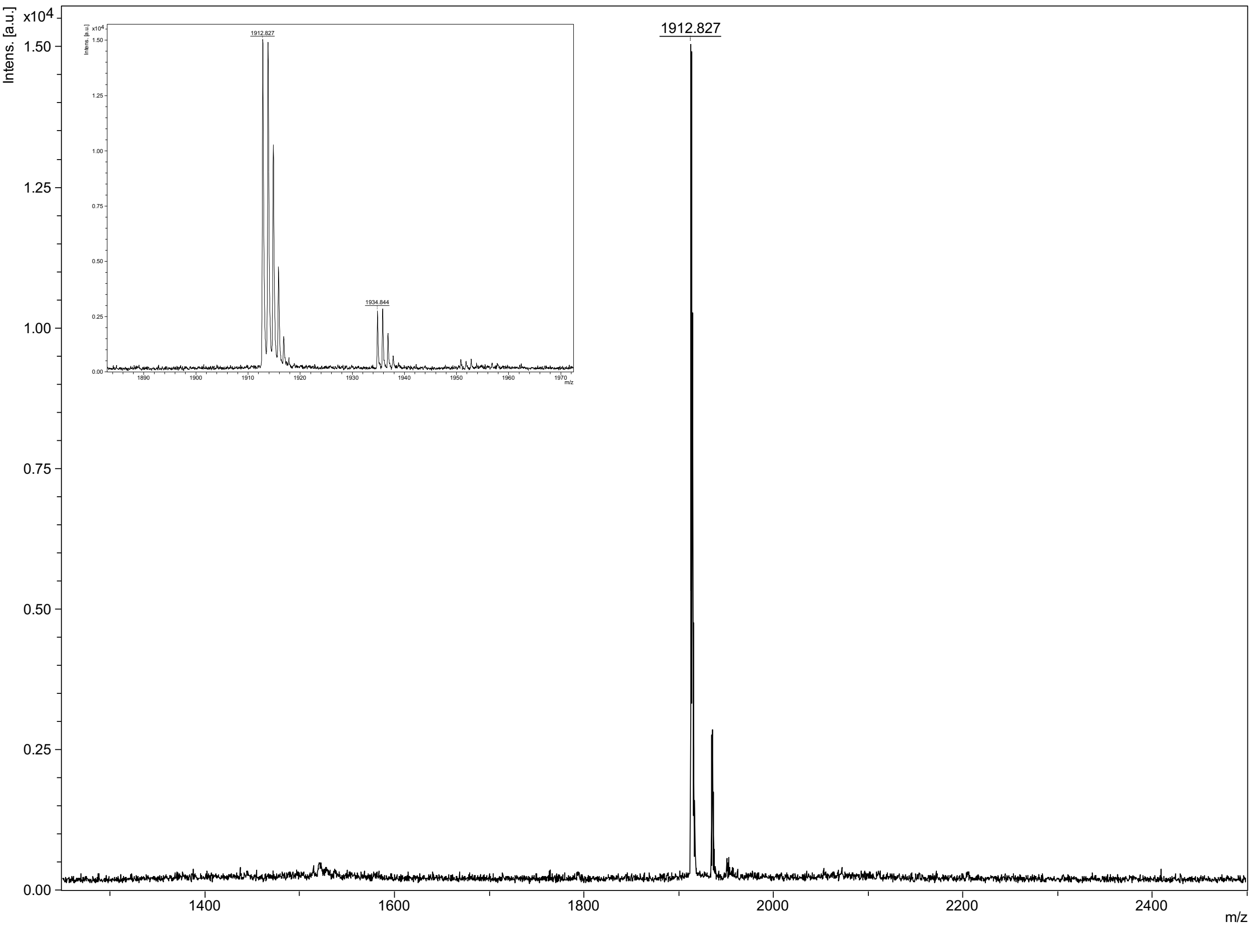

x10^4
Intens. [a.u.]
1912.827
1934.844
m/z

X15

Sequence: 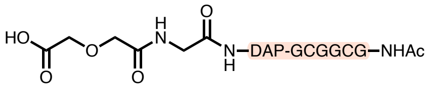

Chemical Formula: $C_{75}H_{96}N_{42}O_{25}$ Exact Mass: 1984.75

LC-MS (ESI) RT = 1.08 min, m/z found: 994.00 $[M+2H]^{2+}$, 662.92 $[M+3H]^{3+}$; calc. 993.38 $[M+2H]^{2+}$, 662.59 $[M+3H]^{3+}$

MALDI-TOF m/z found 1985.93 $[M+H]^{+}$, 2007.94 $[M+Na]^{+}$; calc. 1985.76 $[M+H]^{+}$, 2007.74 $[M+Na]^{+}$

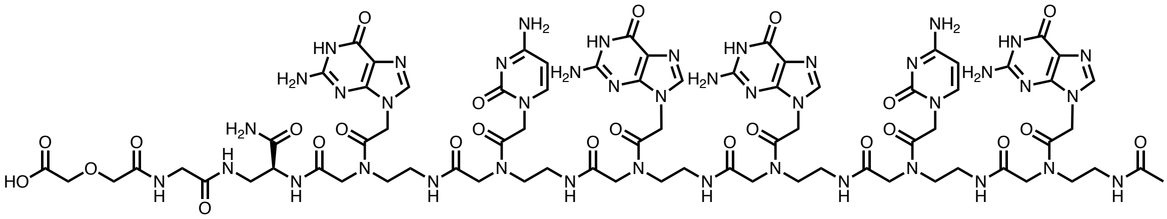

m/z: 1984.75 (100.0%), 1985.76 (83.2%), 1986.76 (39.5%)

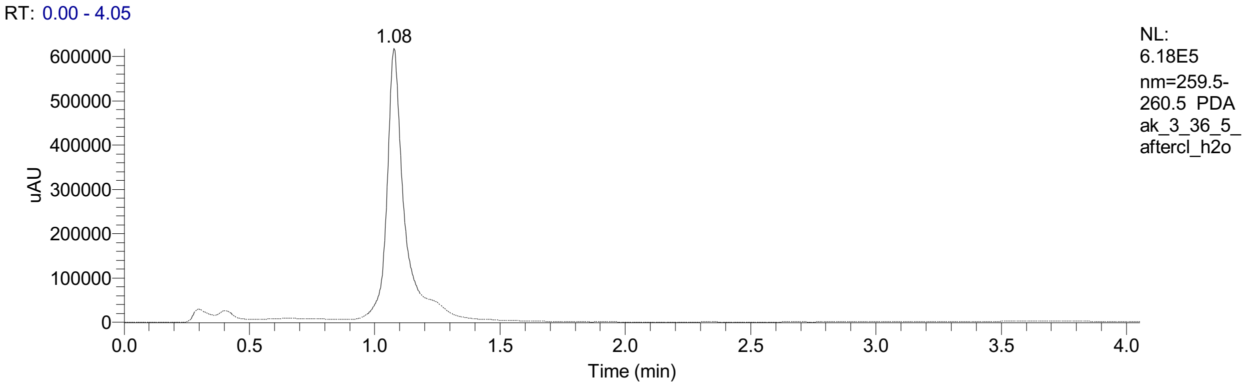

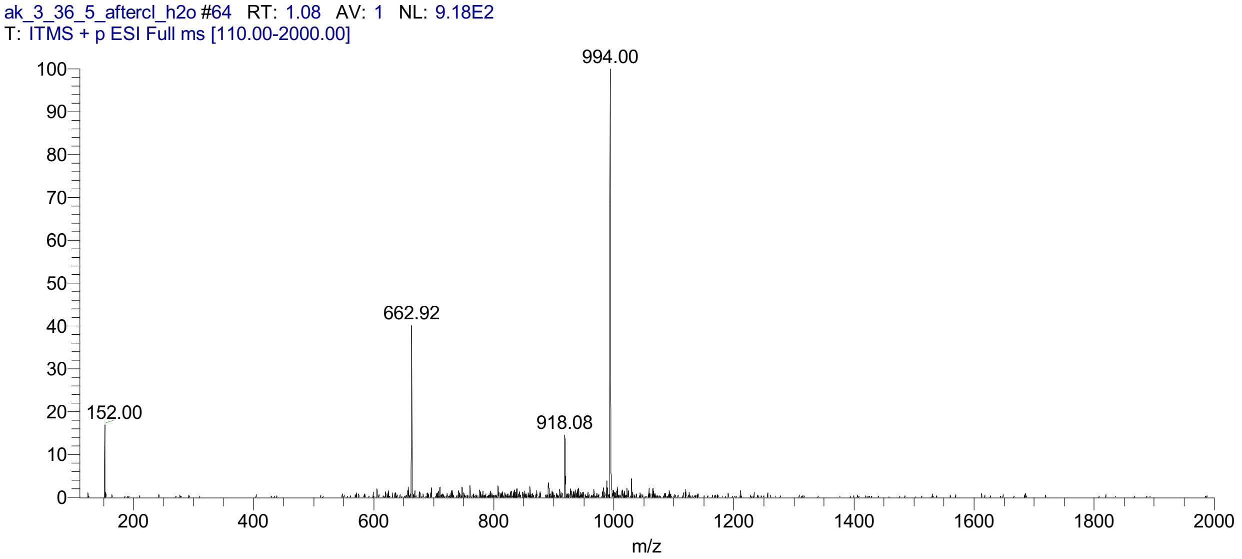

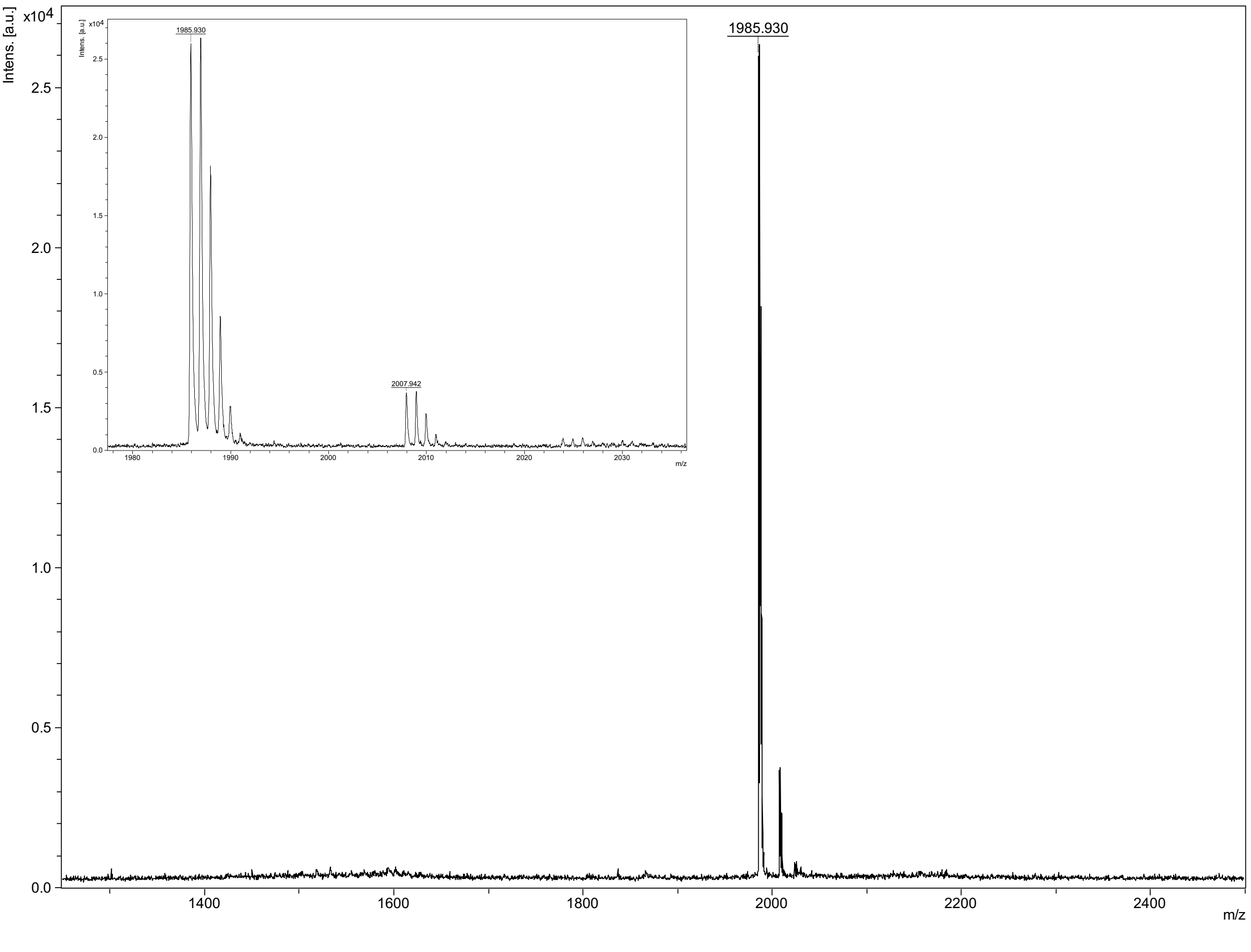

x10^4
Intens. [a.u.]
1985.930
2007.942
m/z

X16

Sequence: 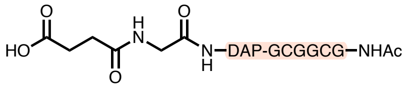

Chemical Formula: $C_{75}H_{96}N_{42}O_{24}$ Exact Mass: 1968.76

LC-MS (ESI) RT = 1.08 min, m/z found: 985.67 $[M+2H]^{2+}$, 657.58 $[M+3H]^{3+}$; calc. 985.39 $[M+2H]^{2+}$, 657.26 $[M+3H]^{3+}$

MALDI-TOF m/z found 1969.99 $[M+H]^{+}$, 1991.99 $[M+Na]^{+}$; calc. 1969.77 $[M+H]^{+}$, 1991.75 $[M+Na]^{+}$

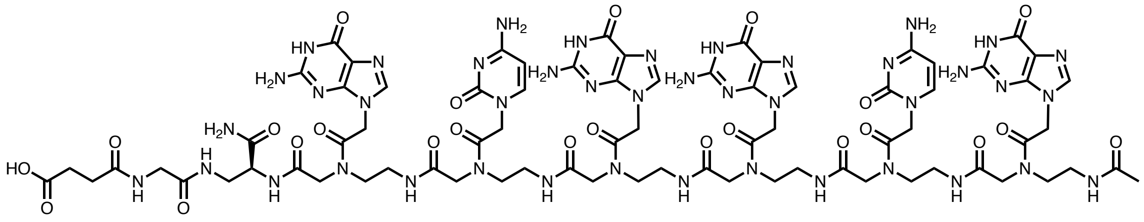

m/z: 1968.76 (100.0%), 1969.76 (98.6%), 1970.76 (50.3%)

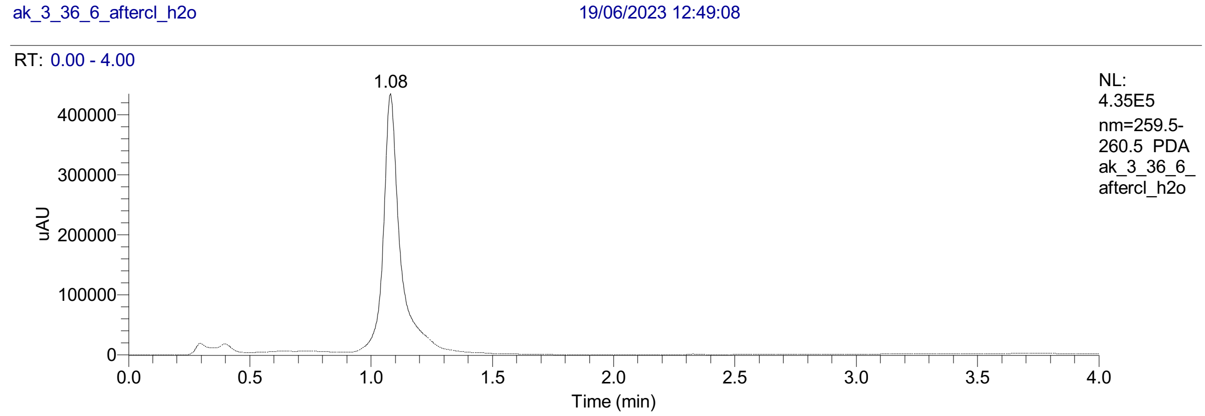

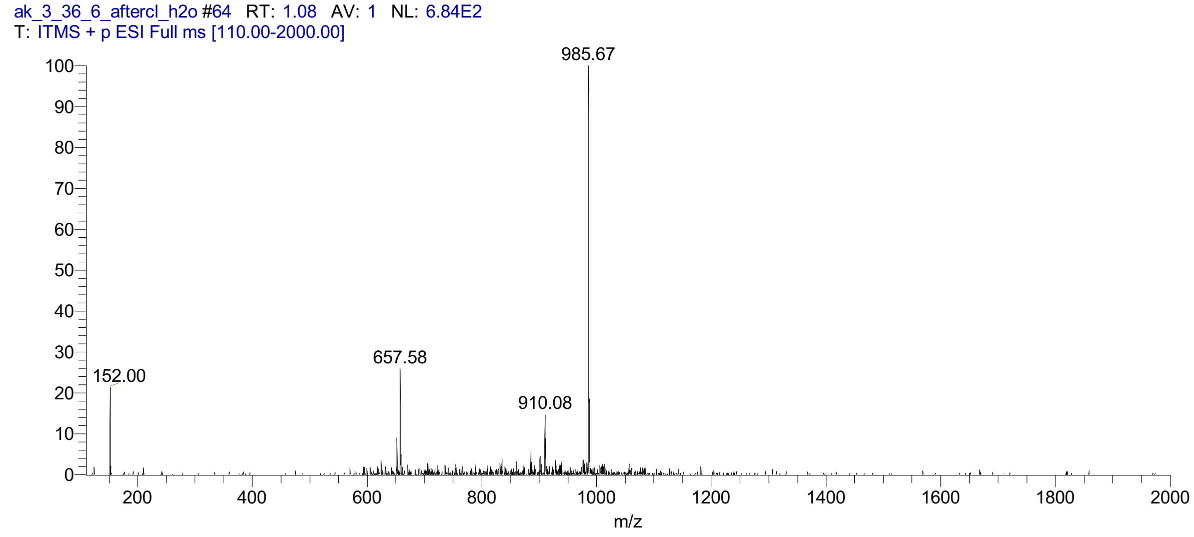

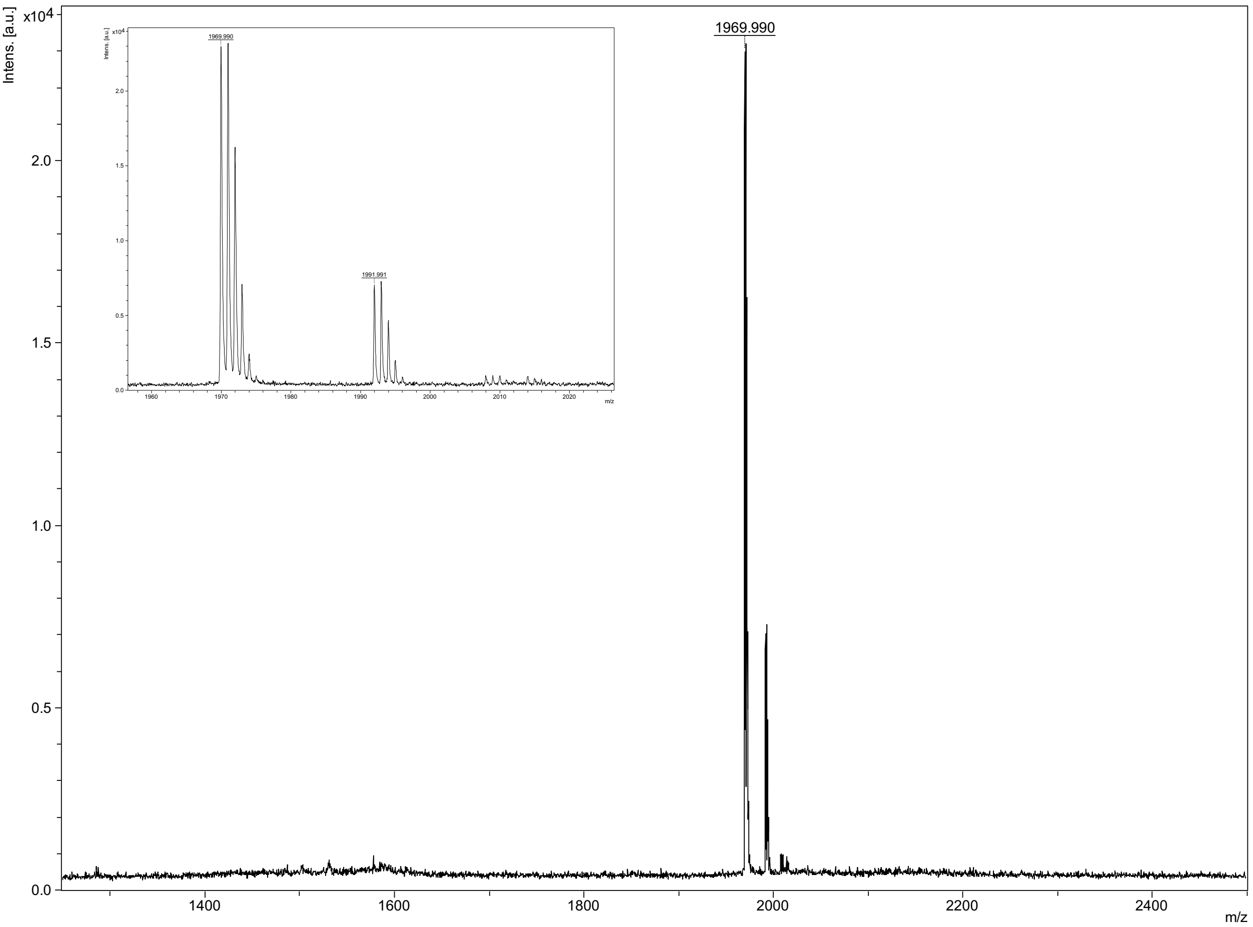

Intens. [a.u.]
x10^4
1969.990
1991.991
m/z

X17

Sequence: H_2N—Lys-GCGGCG—NHAc

Chemical Formula: $C_{72}H_{95}N_{41}O_{20}$ Exact Mass: 1853.77

LC-MS (ESI) RT = 0.99 min, m/z found: 928.25 $[M+2H]^{2+}$, 619.50 $[M+3H]^{3+}$; calc. 927.89 $[M+2H]^{2+}$, 618.93 $[M+3H]^{3+}$

MALDI-TOF m/z found 1855.13 $[M+H]^{+}$, 1877.11 $[M+Na]^{+}$; calc. 1854.78 $[M+H]^{+}$, 1876.76 $[M+Na]^{+}$

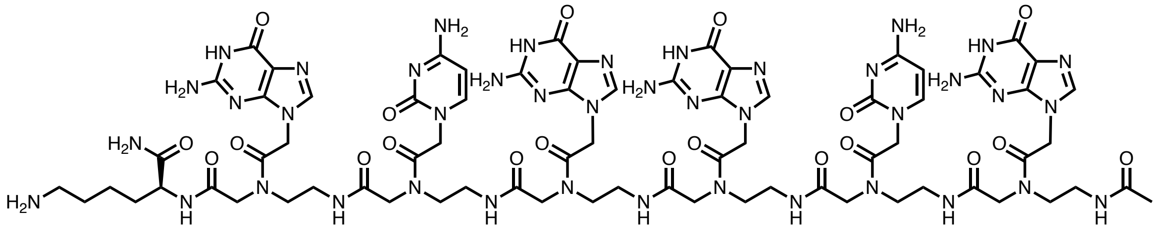

m/z: 1853.77 (100.0%), 1854.77 (79.7%), 1855.77 (46.1%),.76 (1.1%), 1858.78 (1.0%)

ak_4_31_b'_aftercl_h2o 19/06/2023 14:05:37

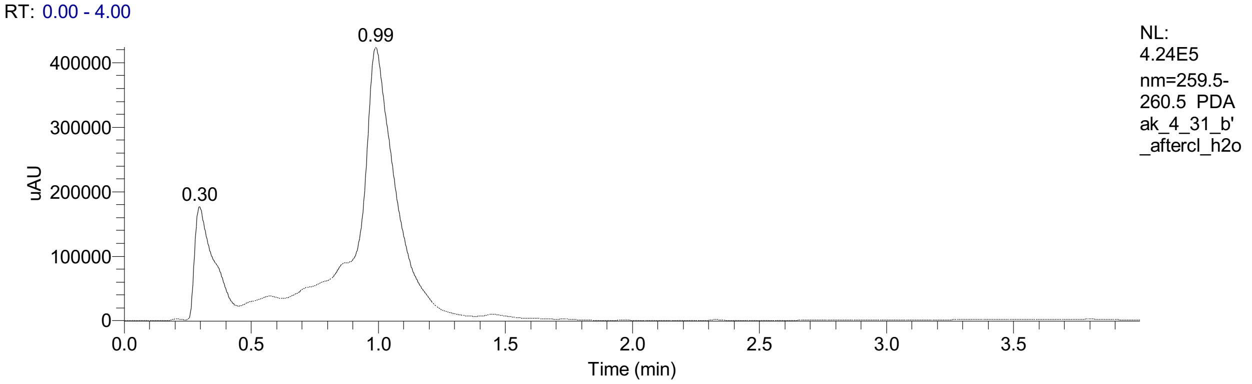

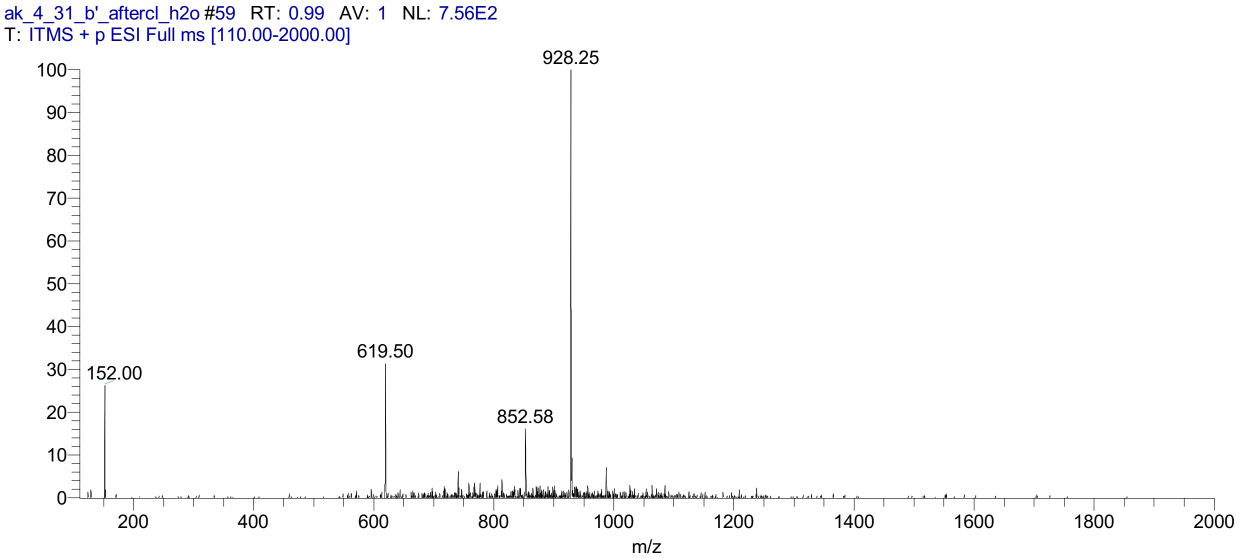

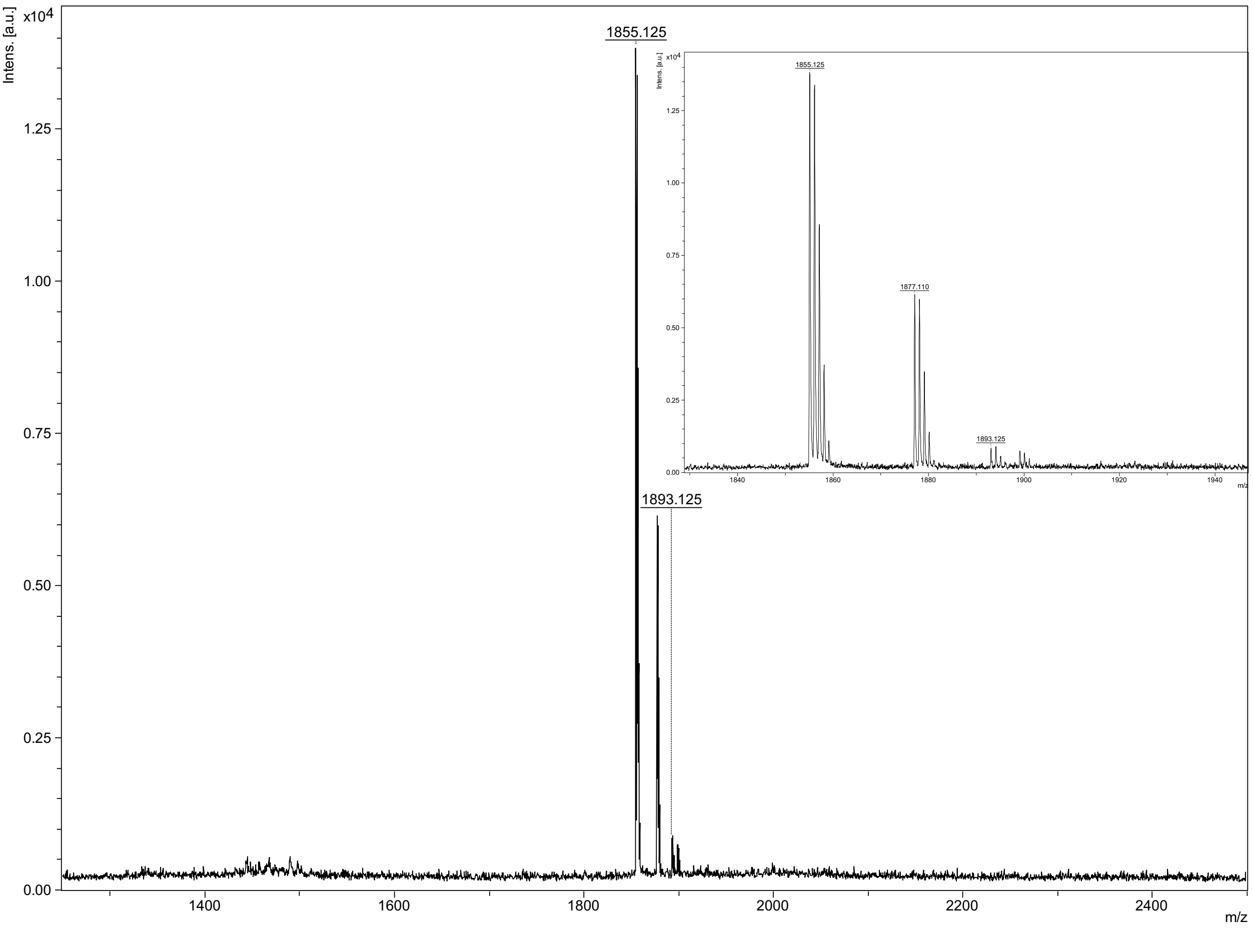

x10^4
Intens. [a.u.]
1855.125
1893.125
1877.110
1.25
1.00
0.75
0.50
0.25
0.00
1400
1600
1800
2000
2200
2400
m/z
1840
1860
1880
1900
1920
1940

Data of PNA_Y

Y1

Sequence: 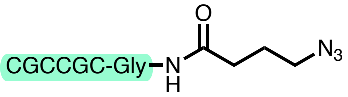

Chemical Formula: $C_{68}H_{89}N_{39}O_{20}$, Exact Mass: 1771.715
LC-MS (ESI) RT = 1.14 min, m/z found: 887.08 $[M+2H]^{2+}$, 591.92 $[M+3H]^{3+}$; calc. 886.86 $[M+2H]^{2+}$, 591.59 $[M+3H]^{3+}$
MALDI-TOF m/z found 1772.81 $[M+H]^{+}$, calc. 1772.72 $[M+H]^{+}$

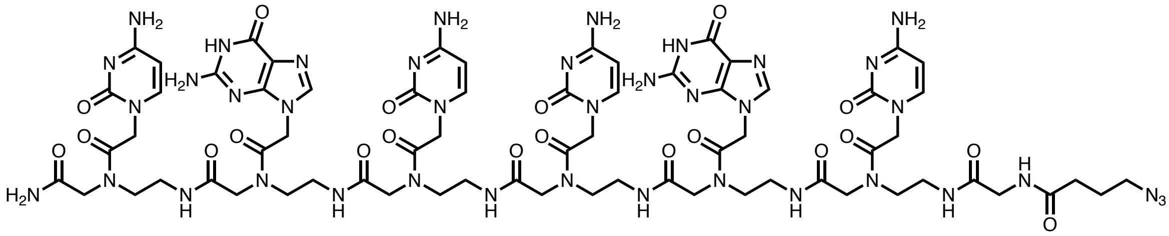

m/z: 1771.71 (100.0%), 1772.72 (75.3%)

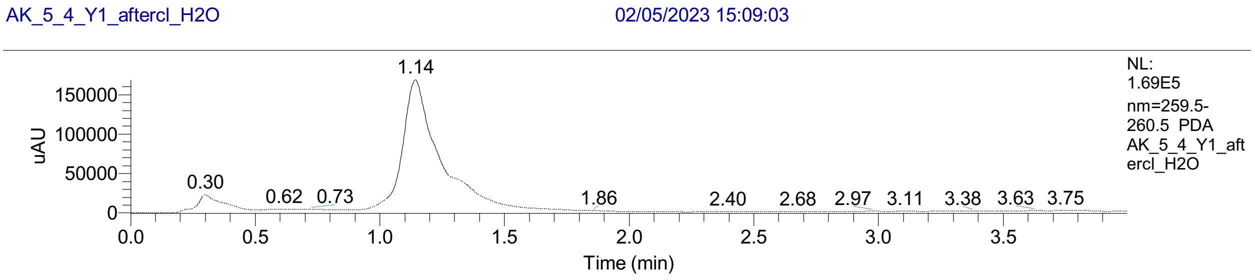

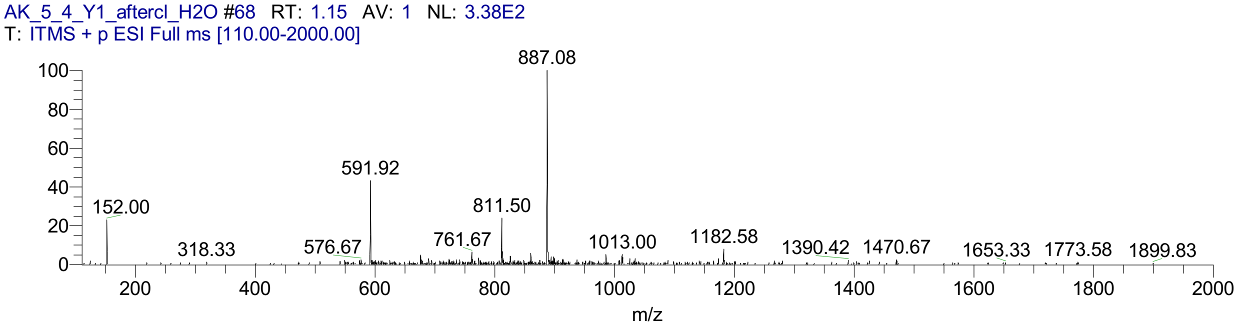

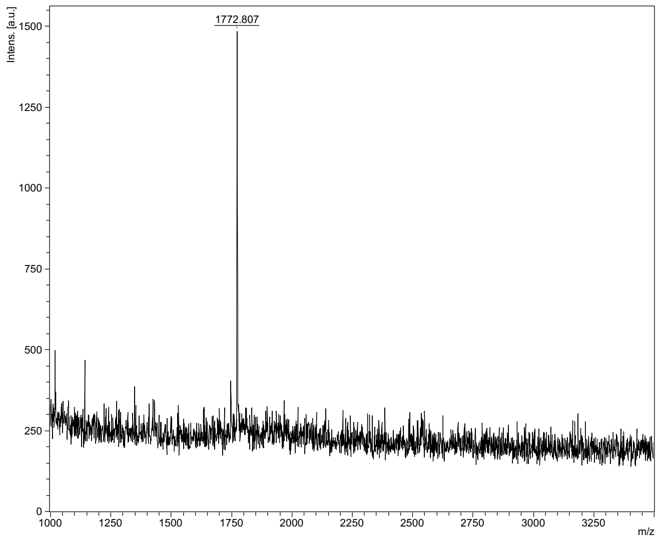

Y2

Sequence: 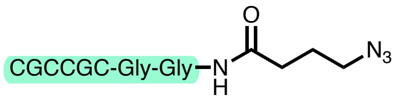

Chemical Formula: $C_{72}H_{95}N_{41}O_{22}$, Exact Mass: 1885.76

LC-MS (ESI) RT = 1.05 min, m/z found: 944.25 $[M+2H]^{2+}$; calc. 943.89 $[M+2H]^{2+}$

MALDI-TOF m/z found 1860.80 $[M-N_2+3H]^{+}$, 1886.74 $[M+H]^{+}$, 1908.76 $[M+Na]^{+}$, 1924.73 $[M+K]^{+}$; calc. 1860.77 $[M-N_2+3H]^{+}$, 1886.76 $[M+H]^{+}$, 1908.75 $[M+Na]^{+}$, 1924.72 $[M+K]^{+}$

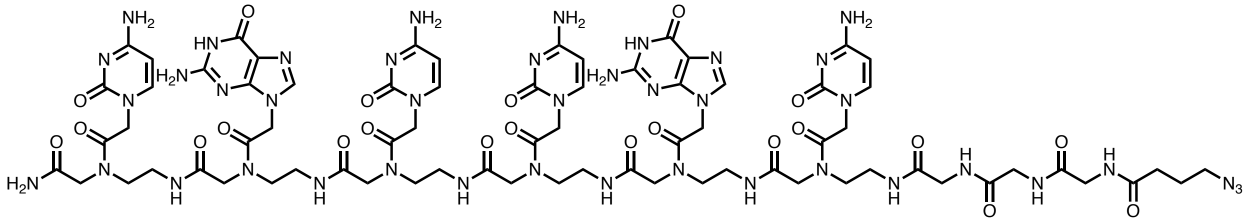

m/z: 1885.76 (100.0%), 1886.76 (79.8%), 1887.76 (46.5%)

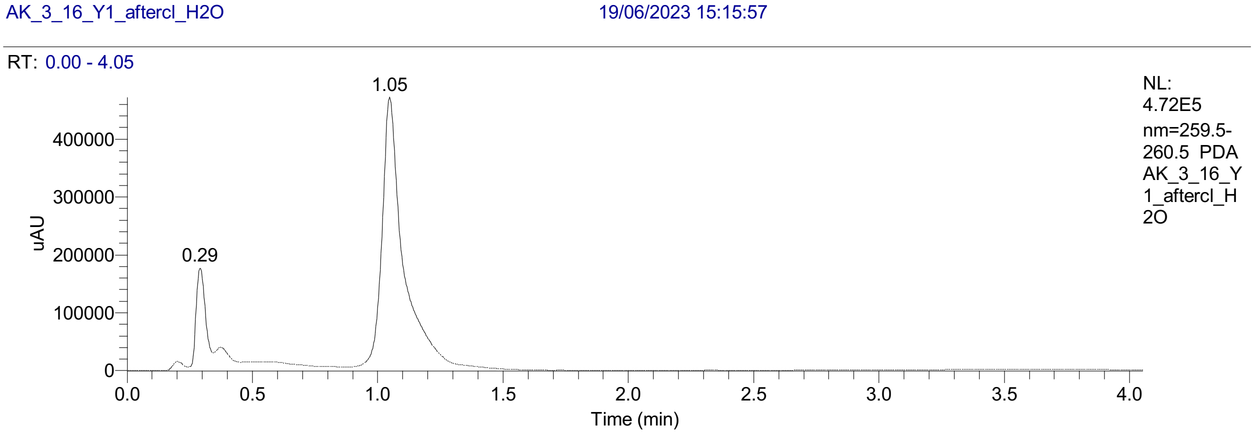

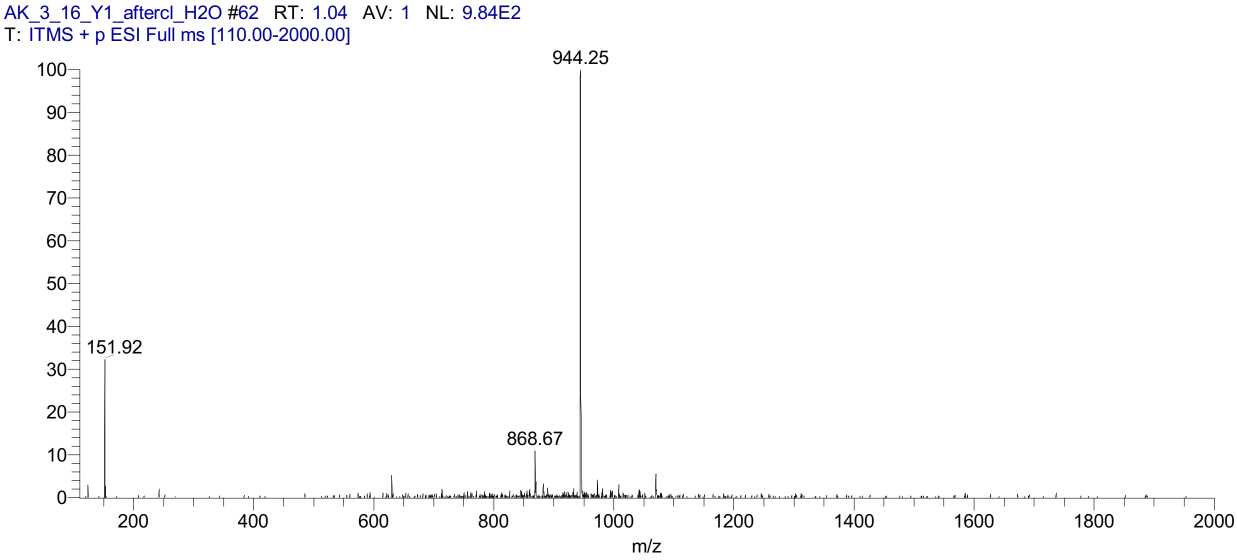

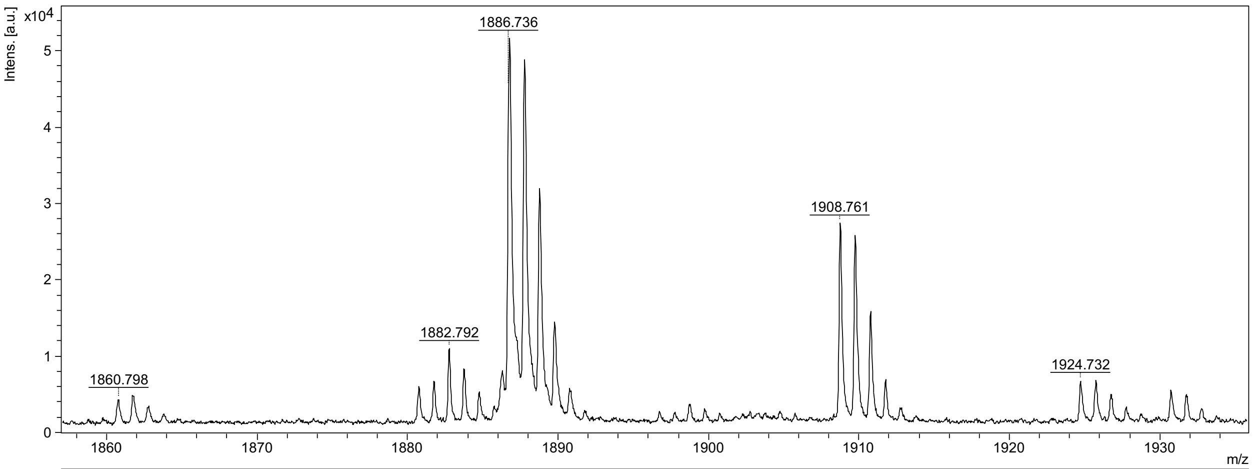
x10^4
Intens. [a.u.]
1860.798
1882.792
1886.736
1908.761
1924.732
m/z

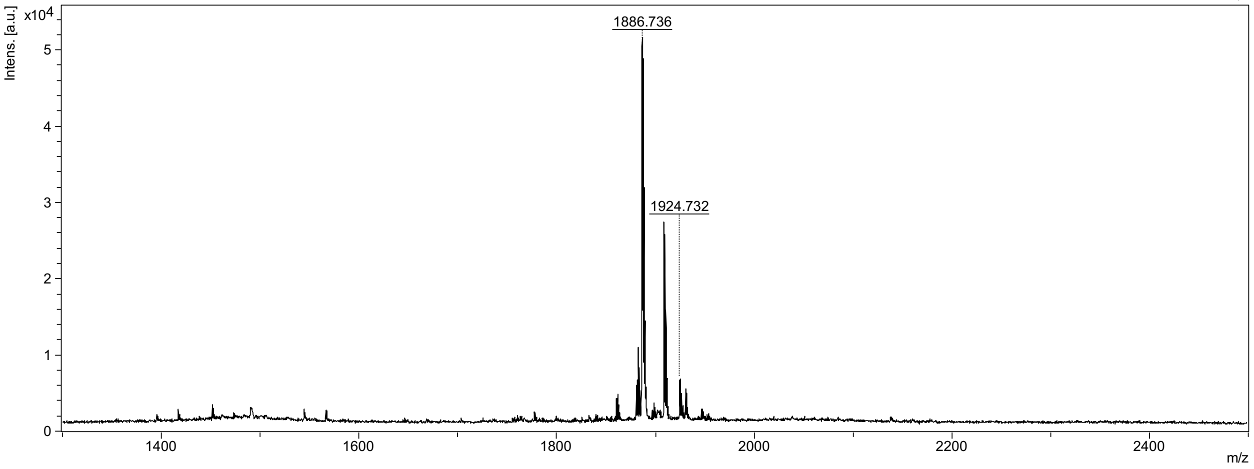
x10^4
Intens. [a.u.]
1886.736
1924.732
m/z

Y3

Sequence: 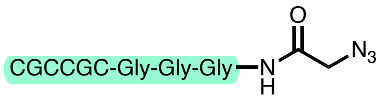

Chemical Formula: $C_{70}H_{91}N_{41}O_{22}$, Exact Mass: 1857.73

LC-MS (ESI) RT = 1.07 min, m/z found: 930.08 $[M+2H]^{2+}$, 620.58 $[M+3H]^{3+}$; calc. 929.87 $[M+2H]^{2+}$, 620.25 $[M+3H]^{3+}$

MALDI-TOF m/z found 1857.55 $[M+H]^{+}$; calc. 1857.73 $[M+H]^{+}$

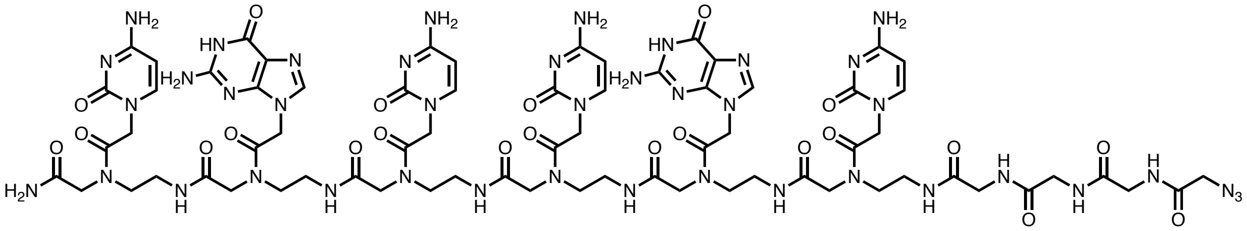

m/z: 1857.73 (100.0%), 1858.73 (77.6%), 1859.73 (45.2%)

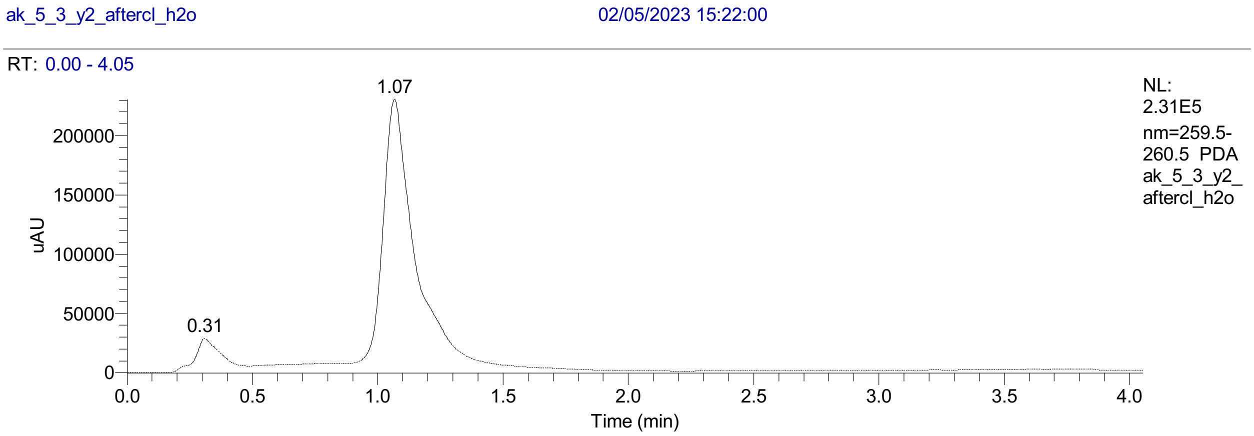

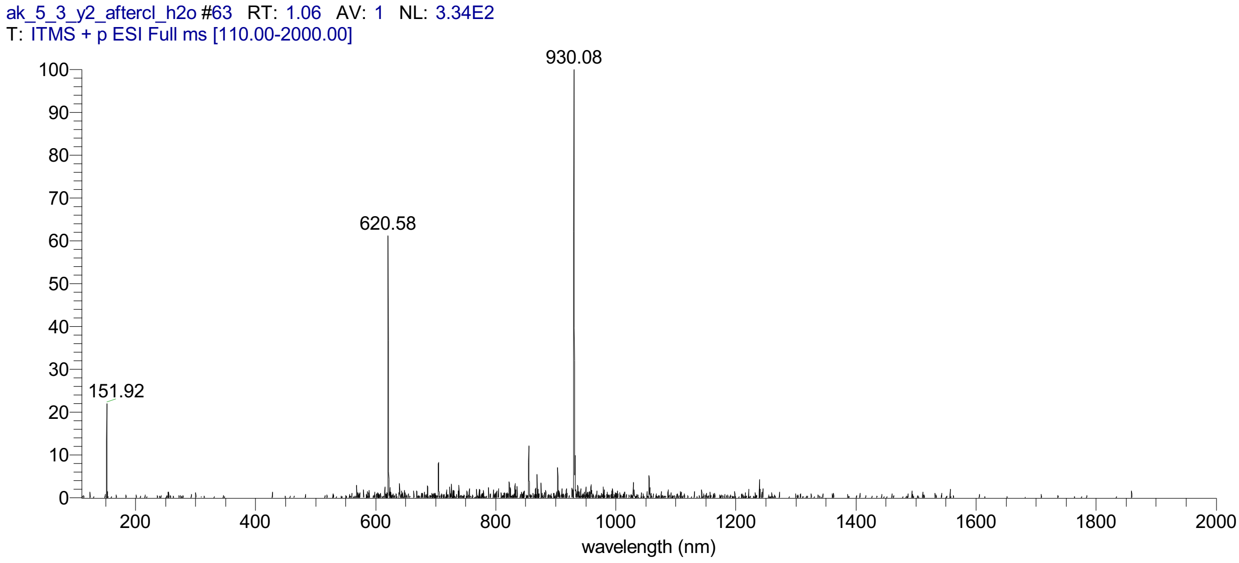

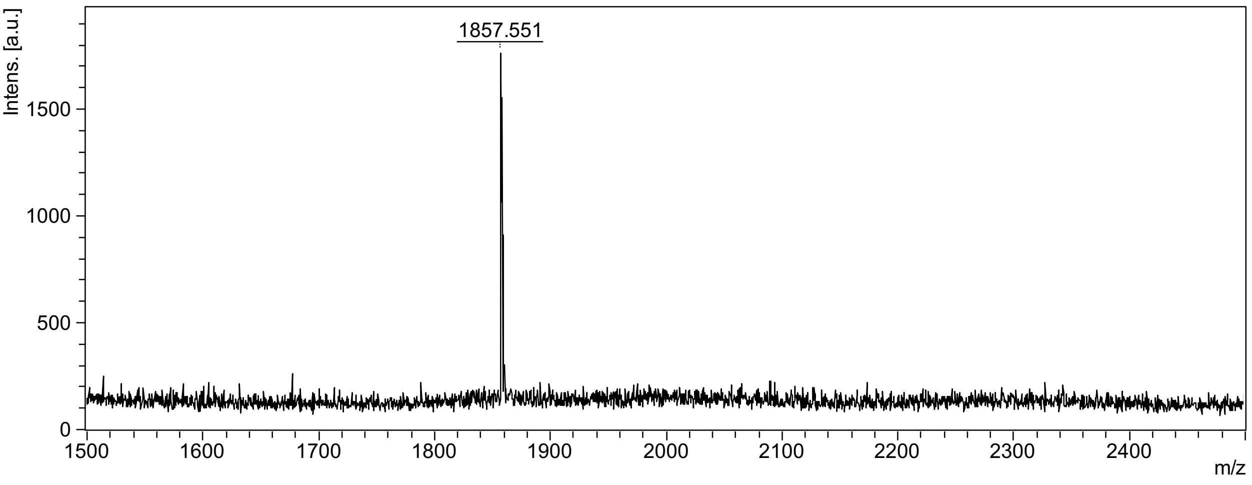

1857.551
Intens. [a.u.]
1500
1000
500
0
1500
1600
1700
1800
1900
2000
2100
2200
2300
2400
m/z

Y4

Sequence: 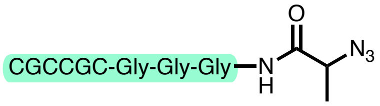

Chemical Formula: $C_{71}H_{93}N_{41}O_{22}$, Exact Mass: 1871.74
LC-MS (ESI) RT = 1.11 min, m/z found: 937.33 $[M+2H]^{2+}$, 625.33 $[M+3H]^{3+}$; calc. 936.88 $[M+2H]^{2+}$, 624.92 $[M+3H]^{3+}$
MALDI-TOF m/z found 1872.90 $[M+H]^{+}$, 1846.90 $[M-N_2+3H]^{+}$, 1894.89 $[M+Na]^{+}$; calc. 1872.75 $[M+H]^{+}$, 1846.76 $[M-N_2+3H]^{+}$, 1894.73 $[M+Na]^{+}$

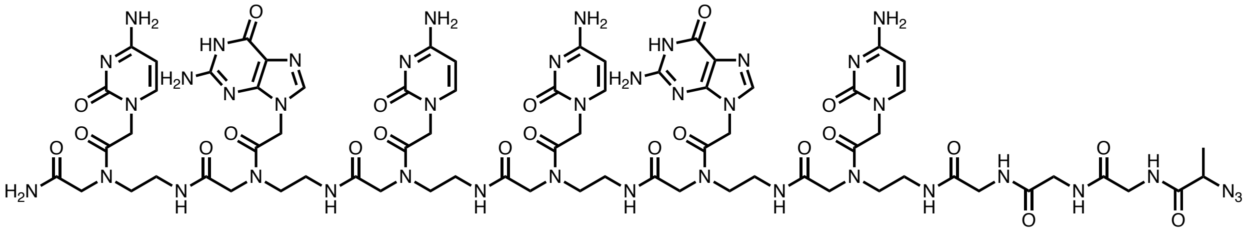

m/z: 1871.74 (100.0%), 1872.75 (78.7%)

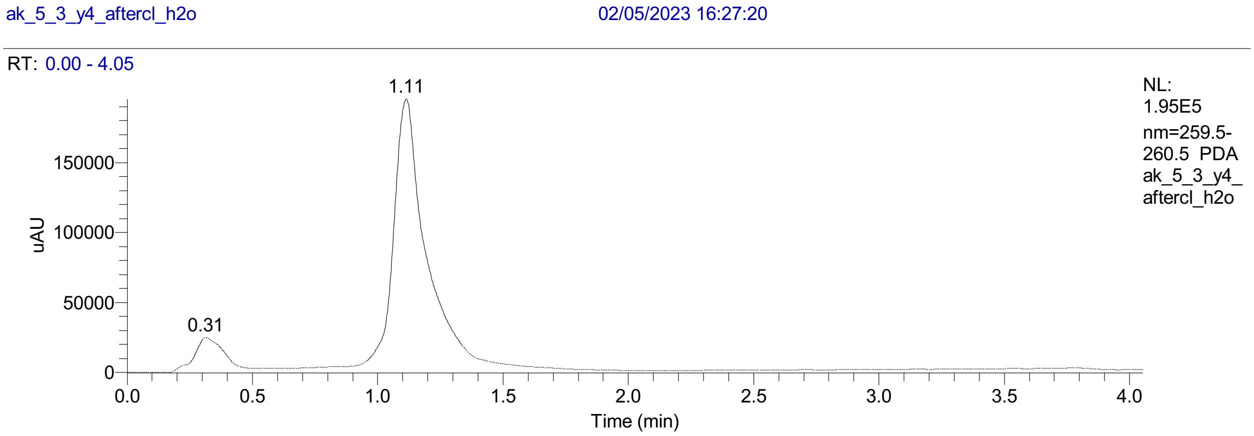

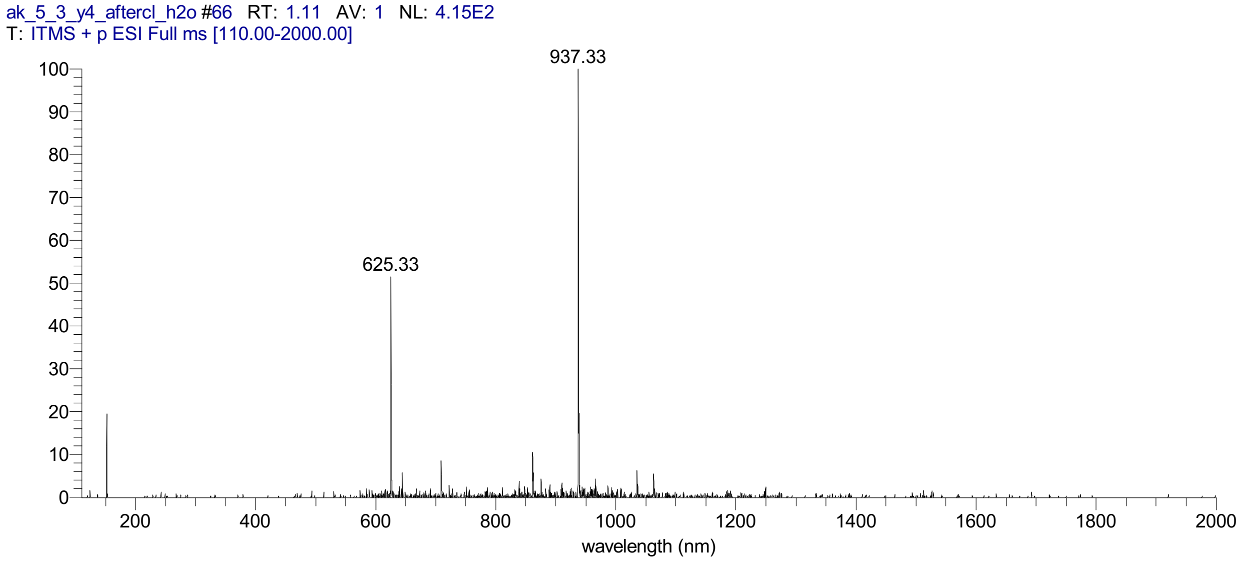

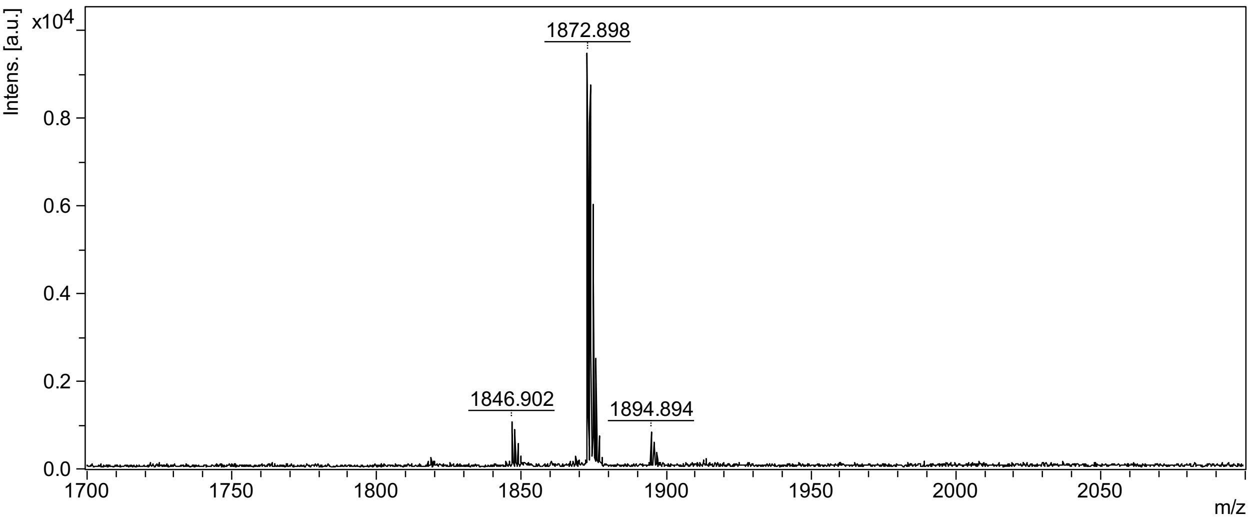
x10^4
Intens. [a.u.]
0.8
0.6
0.4
0.2
0.0
1872.898
1846.902
1894.894
1700
1750
1800
1850
1900
1950
2000
2050
m/z

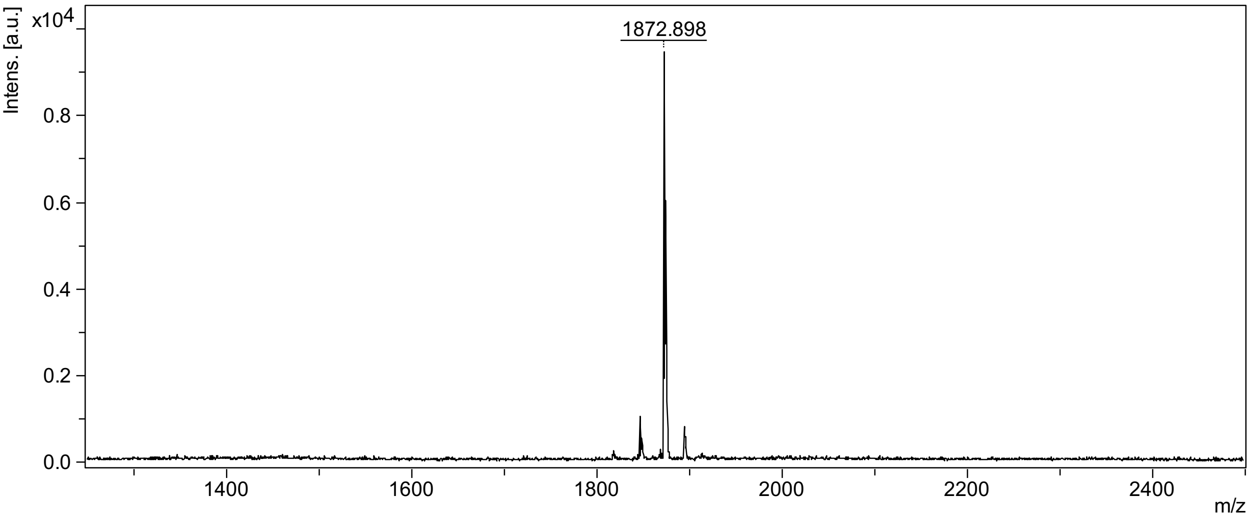
x10^4
Intens. [a.u.]
0.8
0.6
0.4
0.2
0.0
1872.898
1400
1600
1800
2000
2200
2400
m/z

Y5

Sequence: 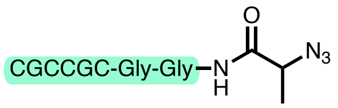

Chemical Formula: $C_{69}H_{90}N_{40}O_{21}$, Exact Mass: 1814.7204
LC-MS (ESI) RT = 1.06 min, m/z found: 908.67 $[M+2H]^{2+}$; calc. 908.37 $[M+2H]^{2+}$
MALDI-TOF m/z found 1815.50 $[M+H]^{+}$; calc. 1815.73 $[M+H]^{+}$

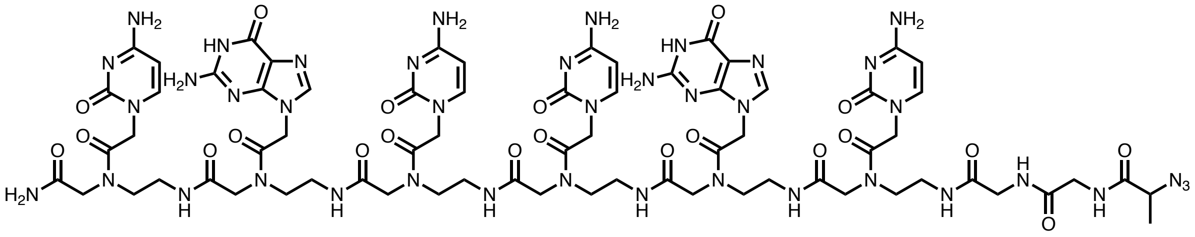

m/z: 1814.72 (100.0%), 1815.72 (90.2%), 1816.73 (28.8%)

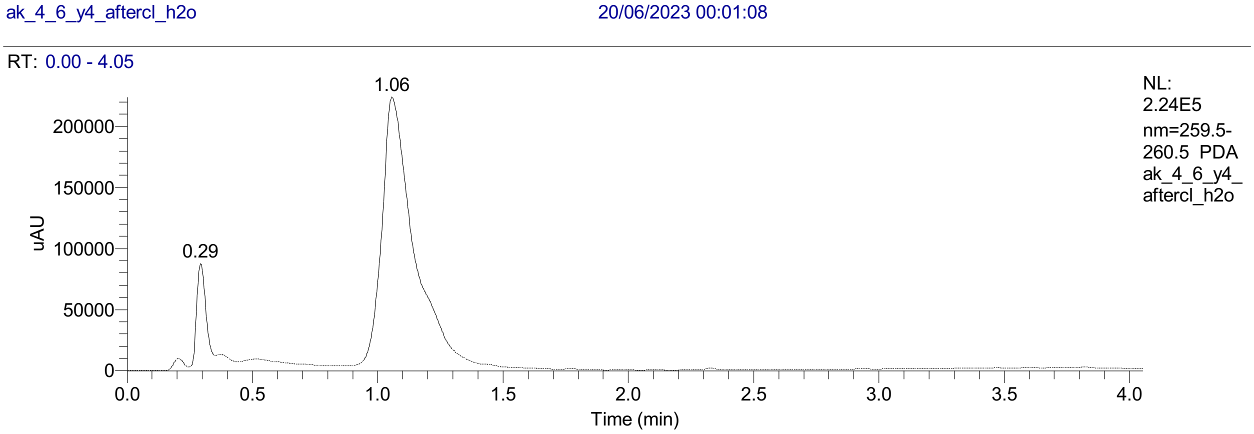

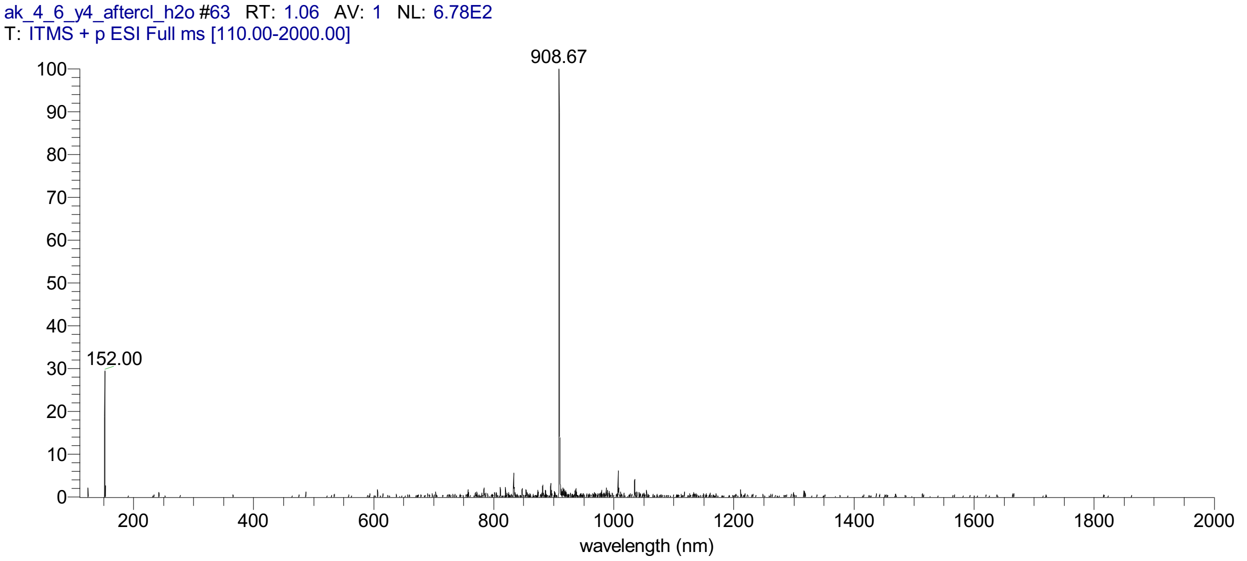

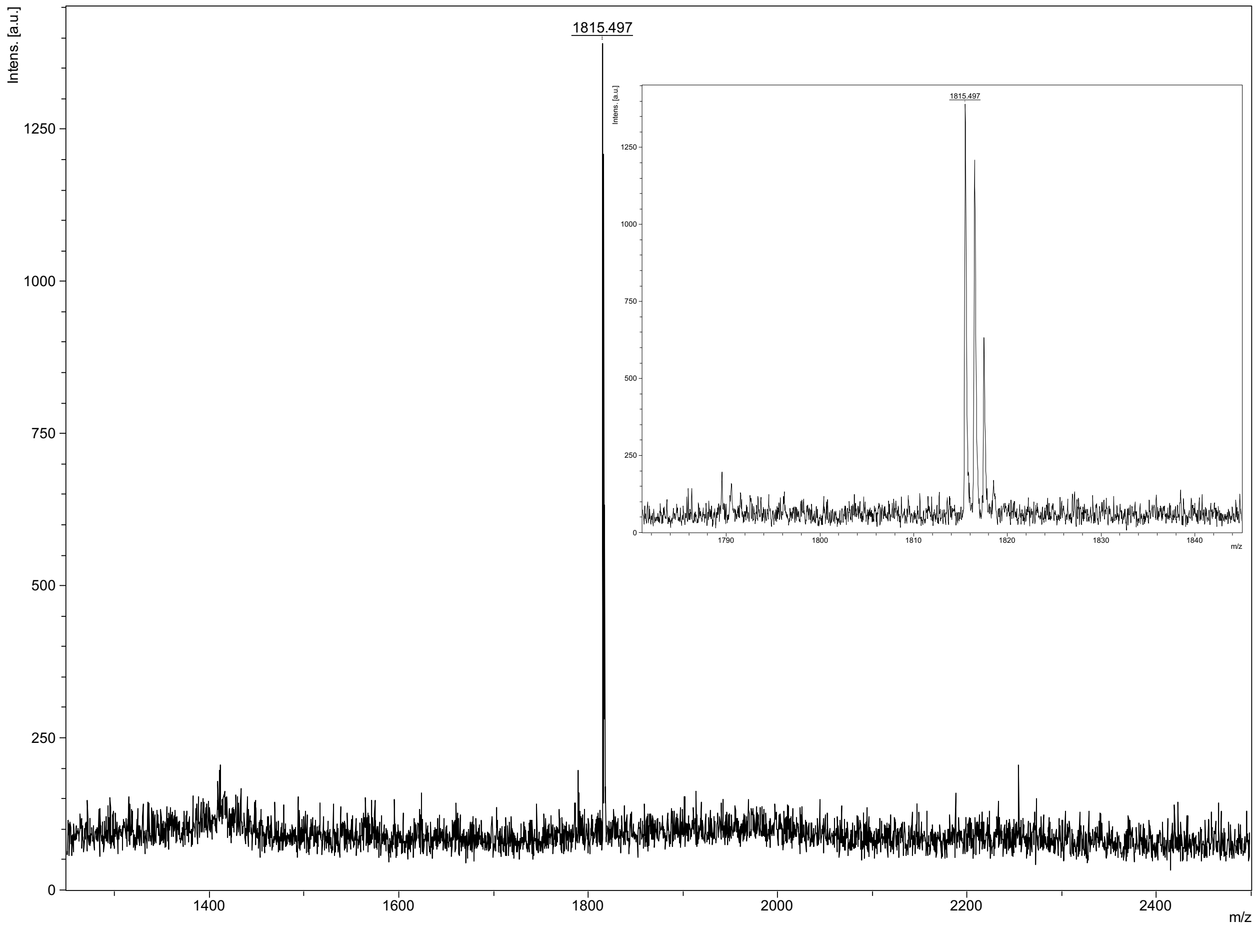

1815.497
Intens. [a.u.]
1250
1000
750
500
250
0
1400
1600
1800
2000
2200
2400
m/z
1815.497
Intens. [a.u.]
1250
1000
750
500
250
0
1790
1800
1810
1820
1830
1840
m/z

Y6

Sequence: 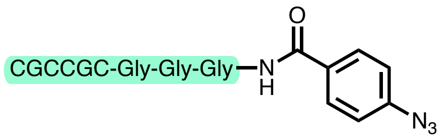

Chemical Formula: $C_{75}H_{93}N_{41}O_{22}$, Exact Mass: 1919.74
LC-MS (ESI) RT = 1.26 min, m/z found: 961.25 $[M+2H]^{2+}$, 641.08 $[M+3H]^{3+}$; calc. 960.88 $[M+2H]^{2+}$, 640.92 $[M+3H]^{3+}$
MALDI-TOF m/z found 1895.12 $[M-N2+3H]^{+}$; calc. 1894.76 $[M-N2+3H]^{+}$

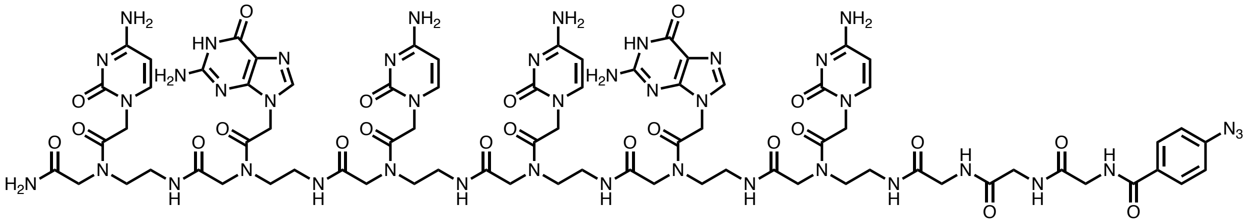

m/z: 1919.74 (100.0%), 1920.75 (83.0%)

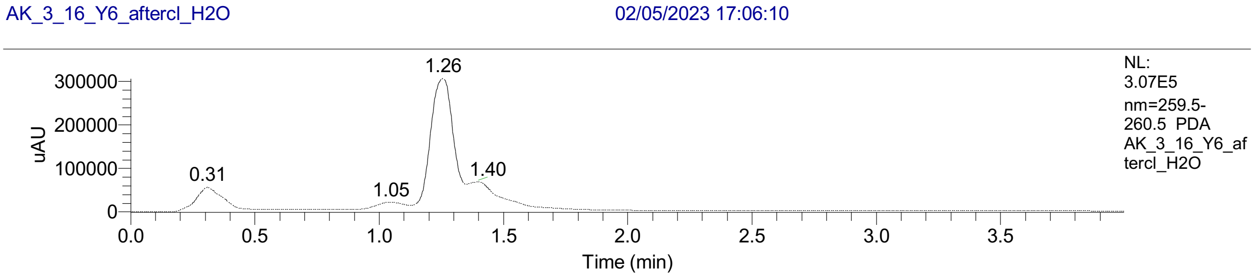

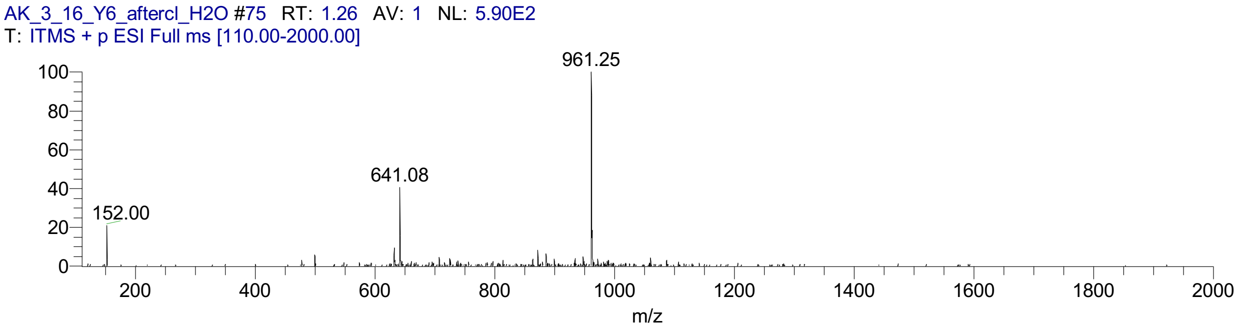

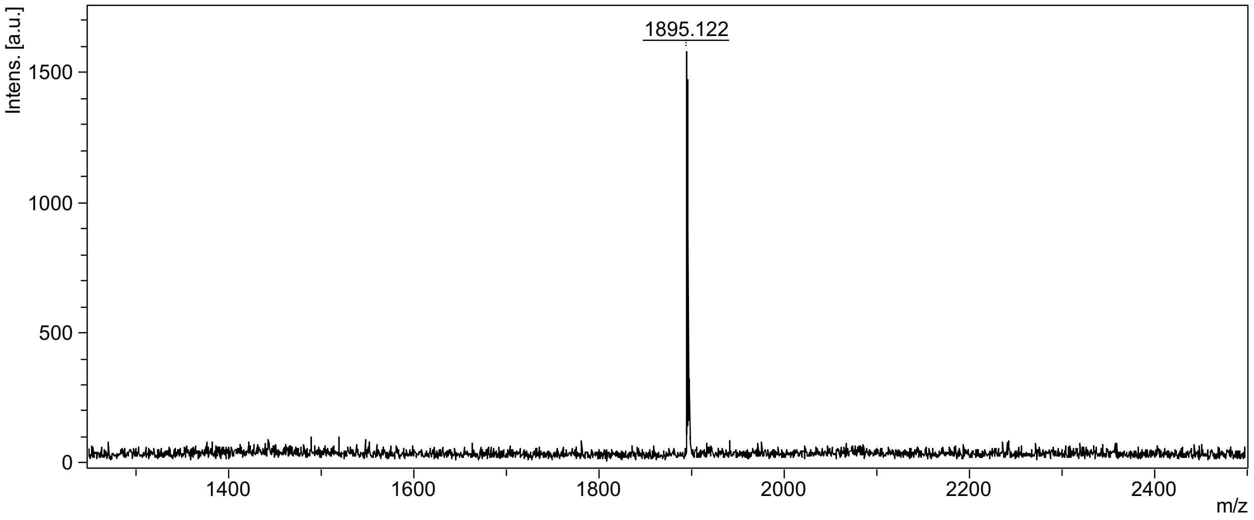

Y7

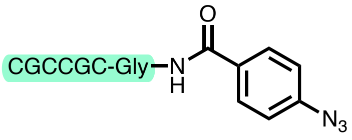

Sequence:

Chemical Formula: $C_{71}H_{87}N_{39}O_{20}$, Exact Mass: 1805.70

LC-MS (ESI) RT = 1.19 min, m/z found: 904.00 $[M+2H]^{2+}$, 889.92 $[M-N_2+2H]^{2+}$; calc. 903.86 $[M+2H]^{2+}$, 889.85 $[M-N_2+2H]^{2+}$

MALDI-TOF m/z found 1780.77 $[M-N_2+3H]^{+}$; calc. 1780.72 $[M-N_2+3H]^{+}$

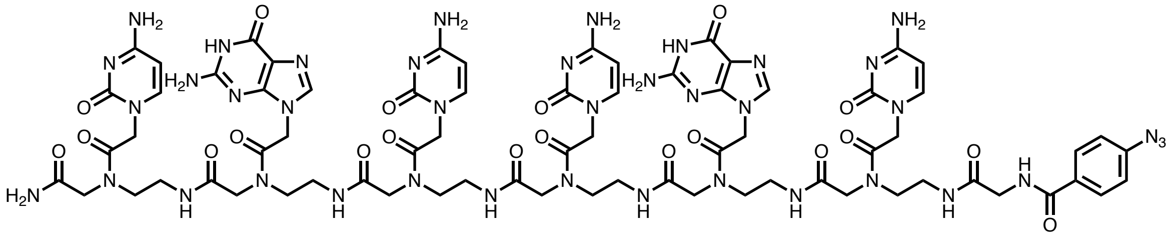

m/z: 1805.70 (100.0%), 1806.70 (92.0%), 1807.71 (30.4%)

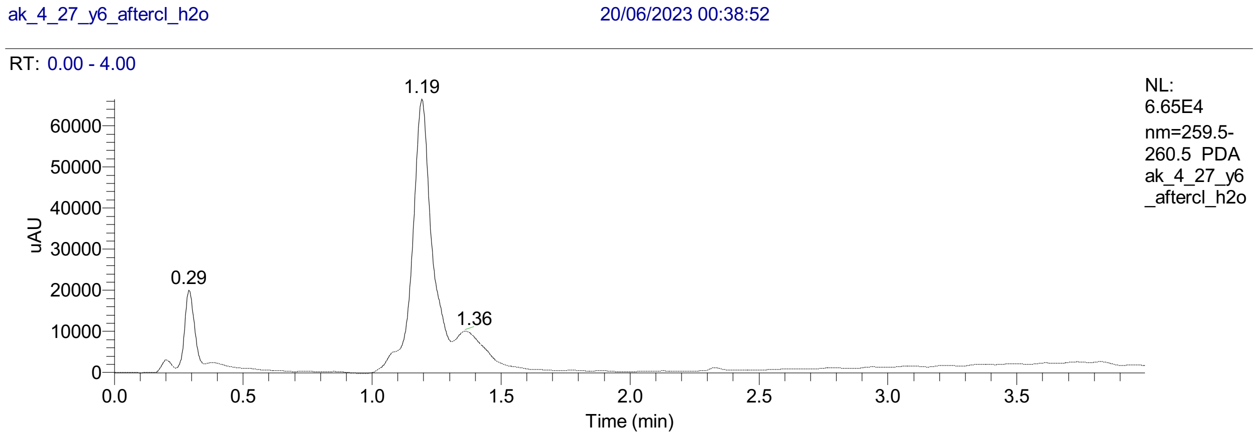

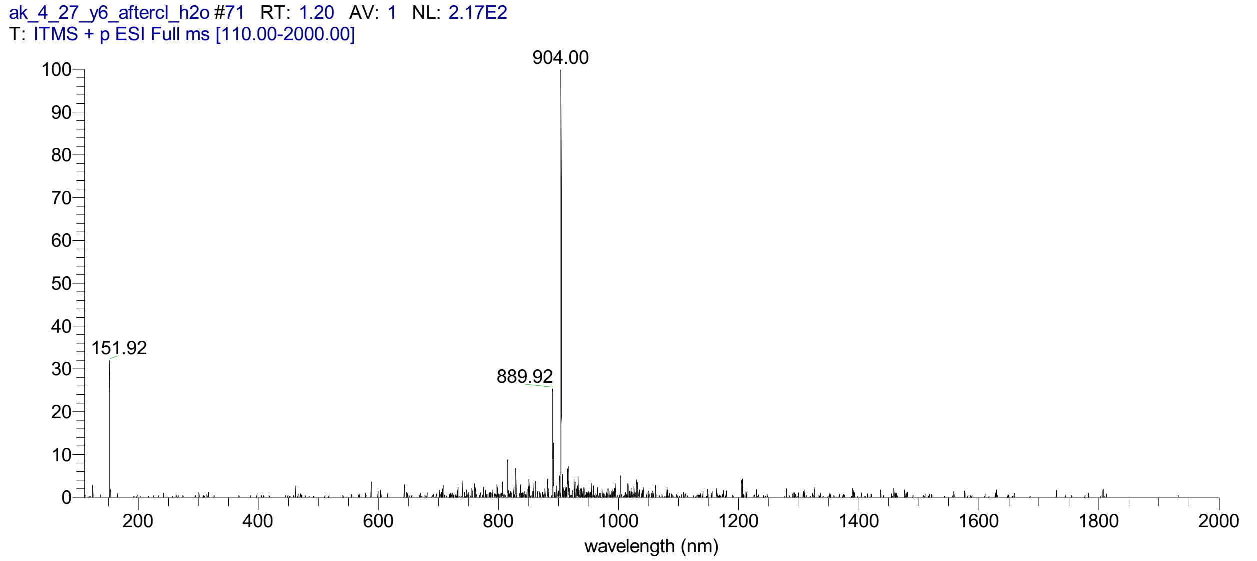

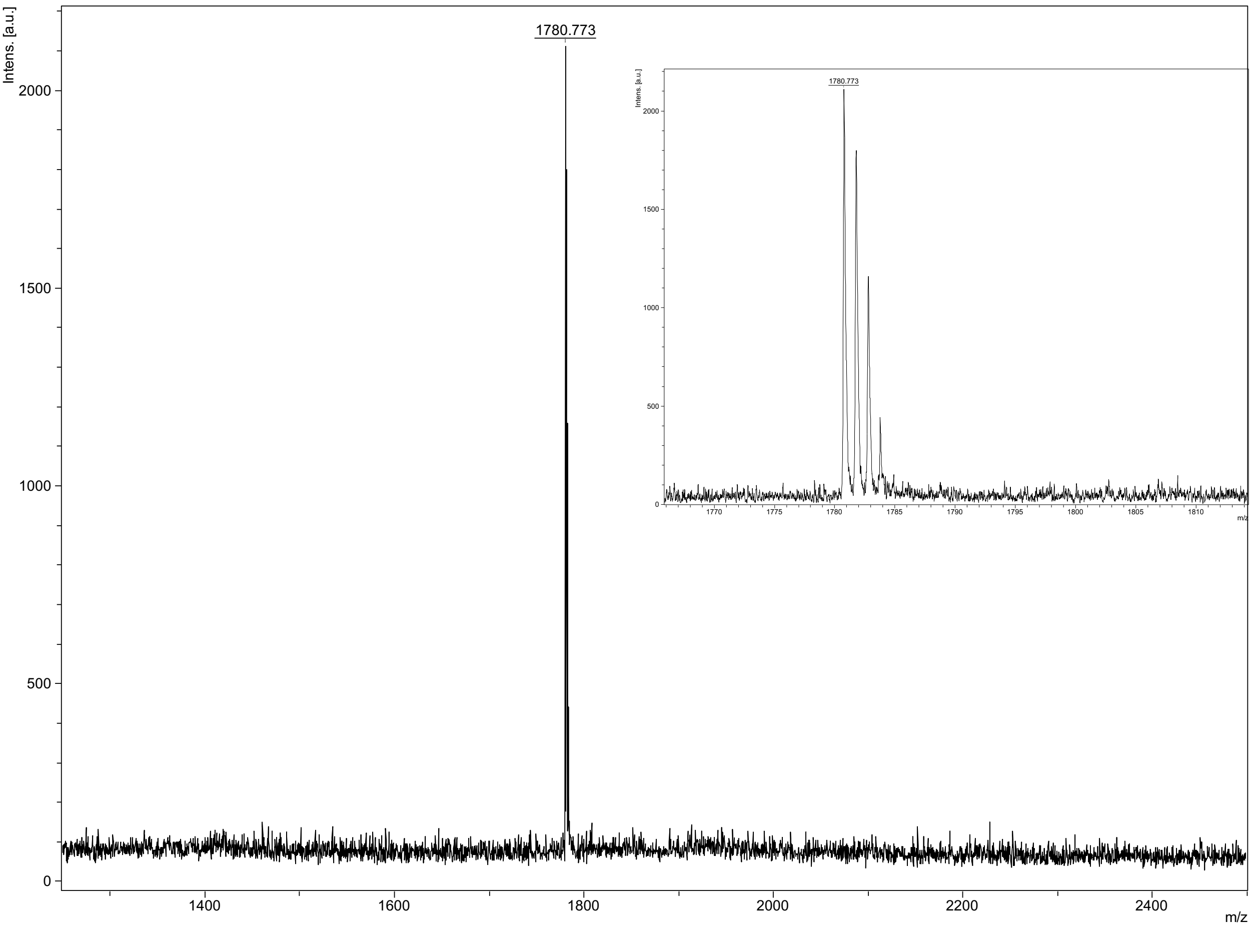

1780.773
Intens. [a.u.]
2000
1500
1000
500
0
1400
1600
1800
2000
2200
2400
m/z

Y8

Sequence:

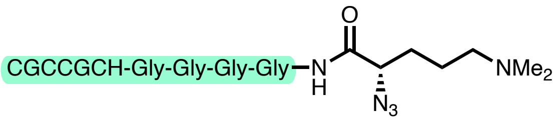

Chemical Formula: $C_{77}H_{105}N_{43}O_{23}$, Exact Mass: 1999.84

LC-MS (ESI) RT = 1.03 min, m/z found: 1001.50 $[M+2H]^{2+}$, 667.92 $[M+3H]^{3+}$; 1001.43 $[M+2H]^{2+}$, 667.62 $[M+3H]^{3+}$

MALDI-TOF m/z found 1974.77 $[M-N_2+3H]^{+}$, 2001.78 $[M+H]^{+}$; calc. 1974.85 $[M-N_2+3H]^{+}$, 2000.84 $[M+H]^{+}$

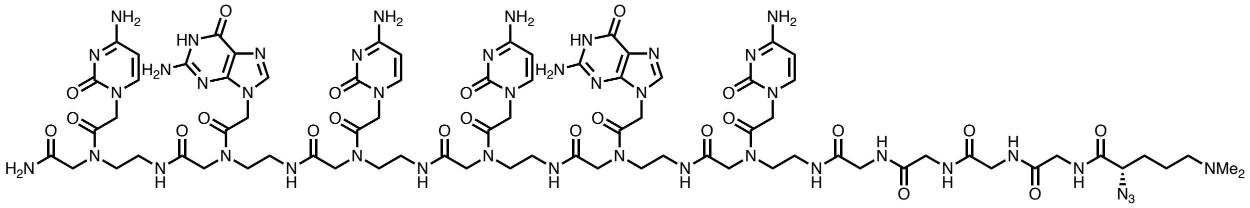

m/z: 1999.84 (100.0%), 2000.84 (85.4%), 2001.84 (53.2%)

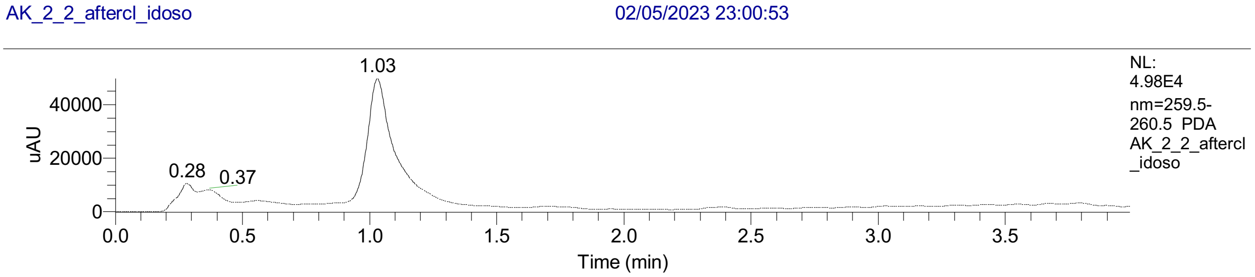

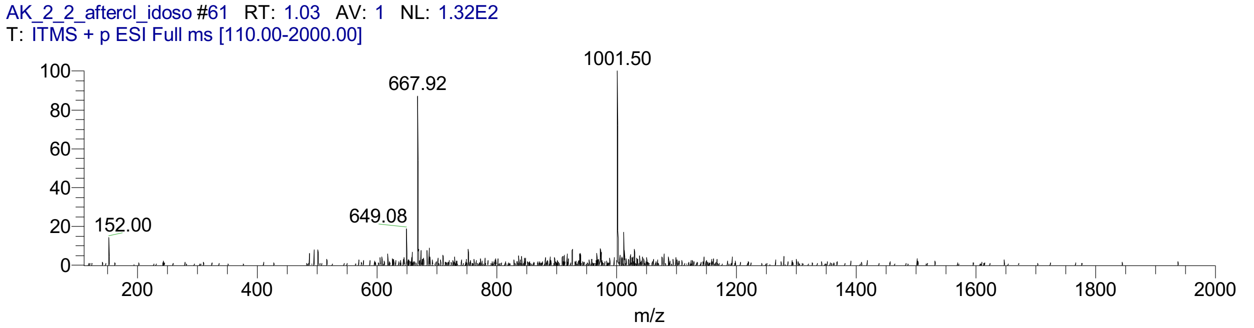

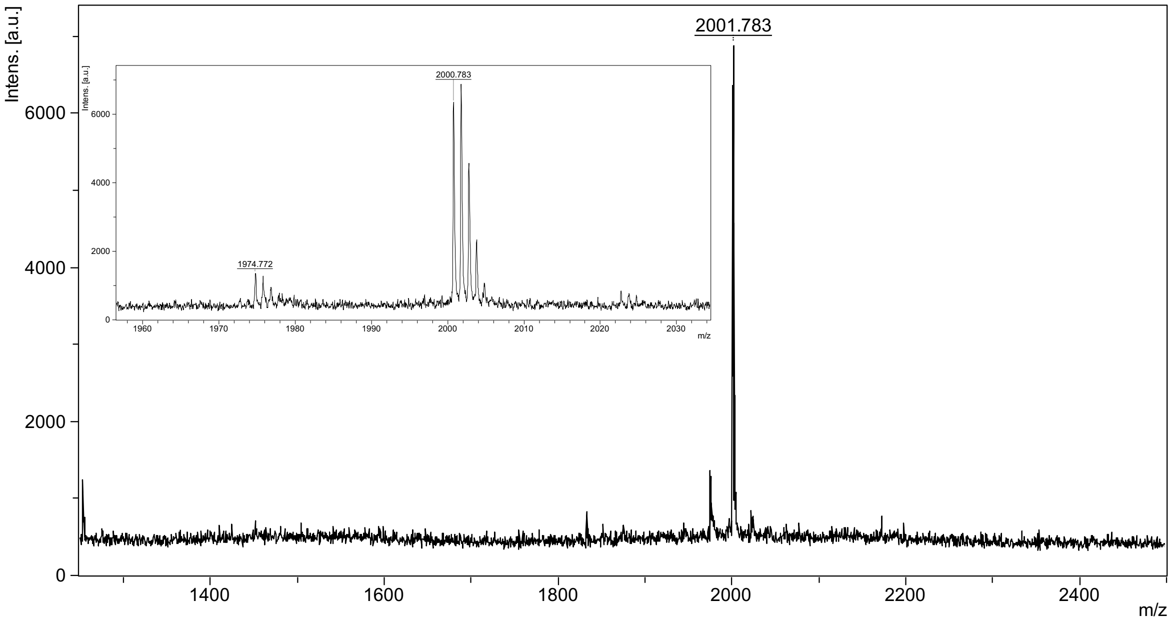

Y9

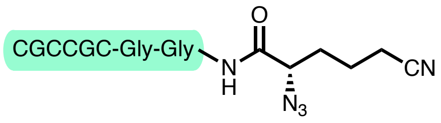

Sequence:

Chemical Formula: $C_{72}H_{93}N_{41}O_{21}$, Exact Mass: 1867.75
LC-MS (ESI) RT = 1.16 min, m/z found: 935.08 $[M+2H]^{2+}$, 624.17 $[M+3H]^{3+}$; calc. 934.88 $[M+2H]^{2+}$, 623.59 $[M+3H]^{3+}$
MALDI-TOF m/z found 1868.79 $[M+H]^{+}$, 1890.75 $[M+Na]^{+}$; calc. 1868.75 $[M+H]^{+}$, 1890.74 $[M+Na]^{+}$

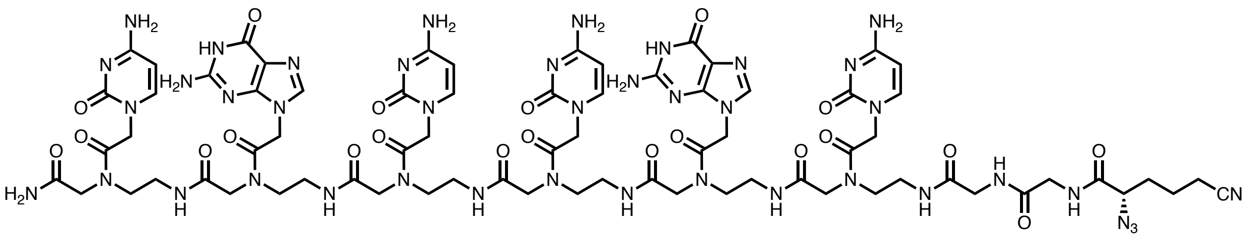

m/z: 1867.75 (100.0%), 1868.75 (79.7%), 1869.75 (46.9%)

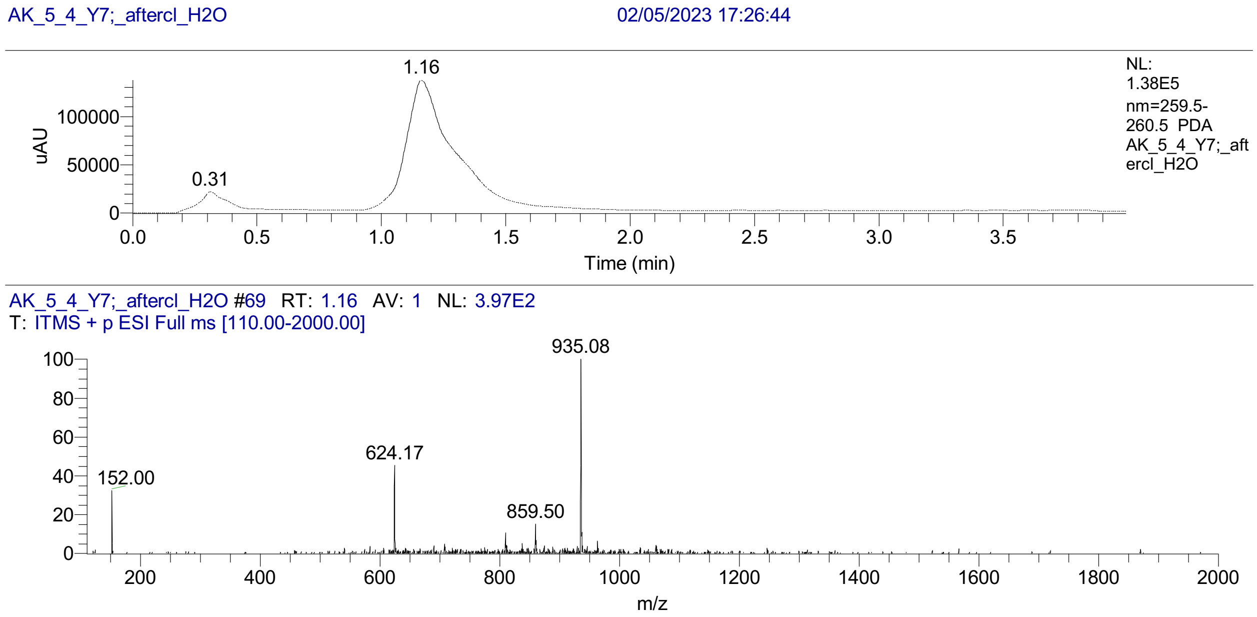

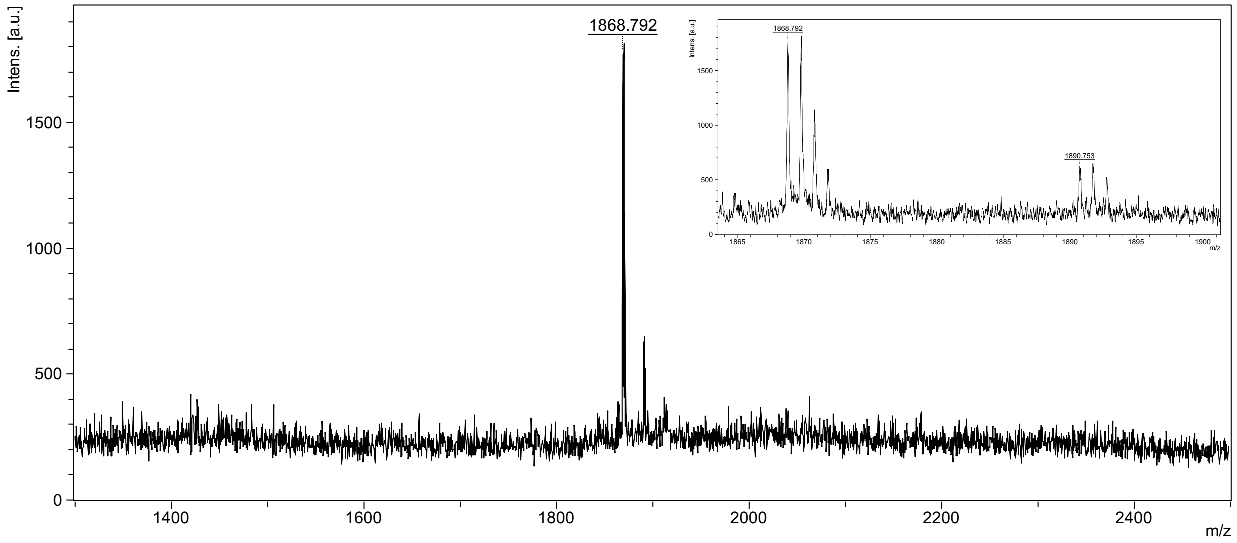

Y10

Sequence: CGCCGC-Gly-Gly-Gly-Gly–NH_2

Chemical Formula: $C_{70}H_{93}N_{39}O_{22}$, Exact Mass: 1831.74

LC-MS (ESI) RT = 0.30 min, m/z found: 917.42 $[M+2H]^{2+}$; calc. 916.88 $[M+2H]^{2+}$

MALDI-TOF m/z found 1833.66 $[M+H]^+$, 1855.61 $[M+Na]^+$, 1871.58 $[M+K]^+$; calc. 1832.74 $[M+H]^+$, 1854.73 $[M+Na]^+$, 1870.70 $[M+K]^+$

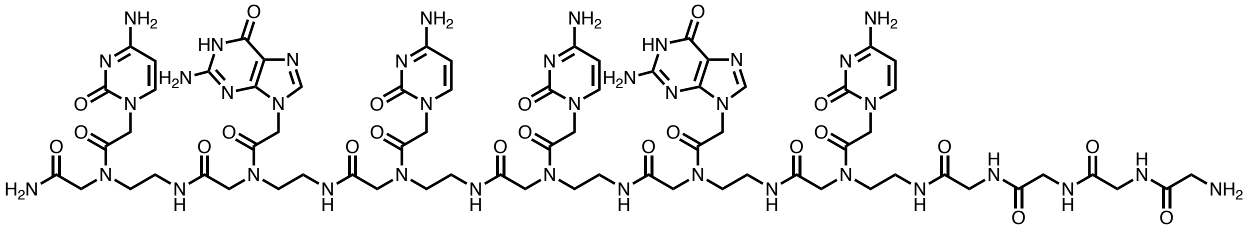

m/z: 1831.74 (100.0%), 1832.74 (77.6%), 1833.74 (44.6%)

ak_4_26_3_afterck_h2o 19/06/2023 23:35:56

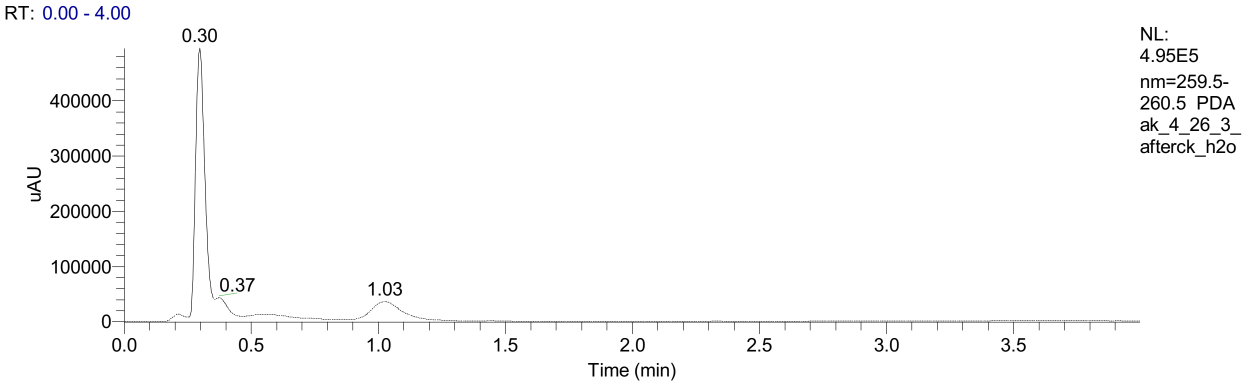

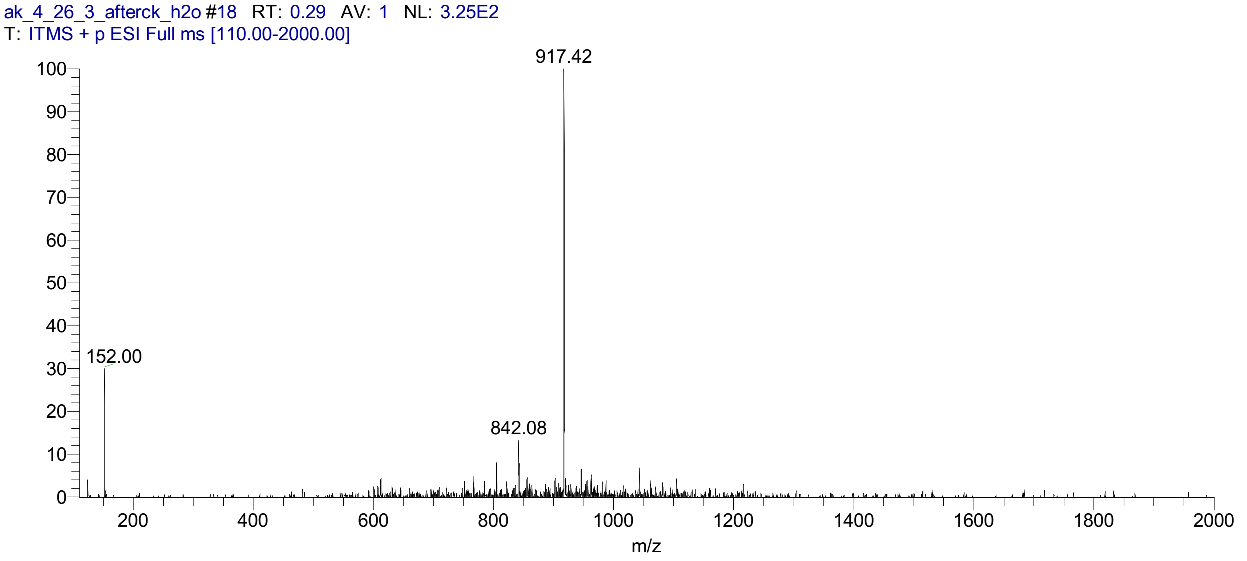

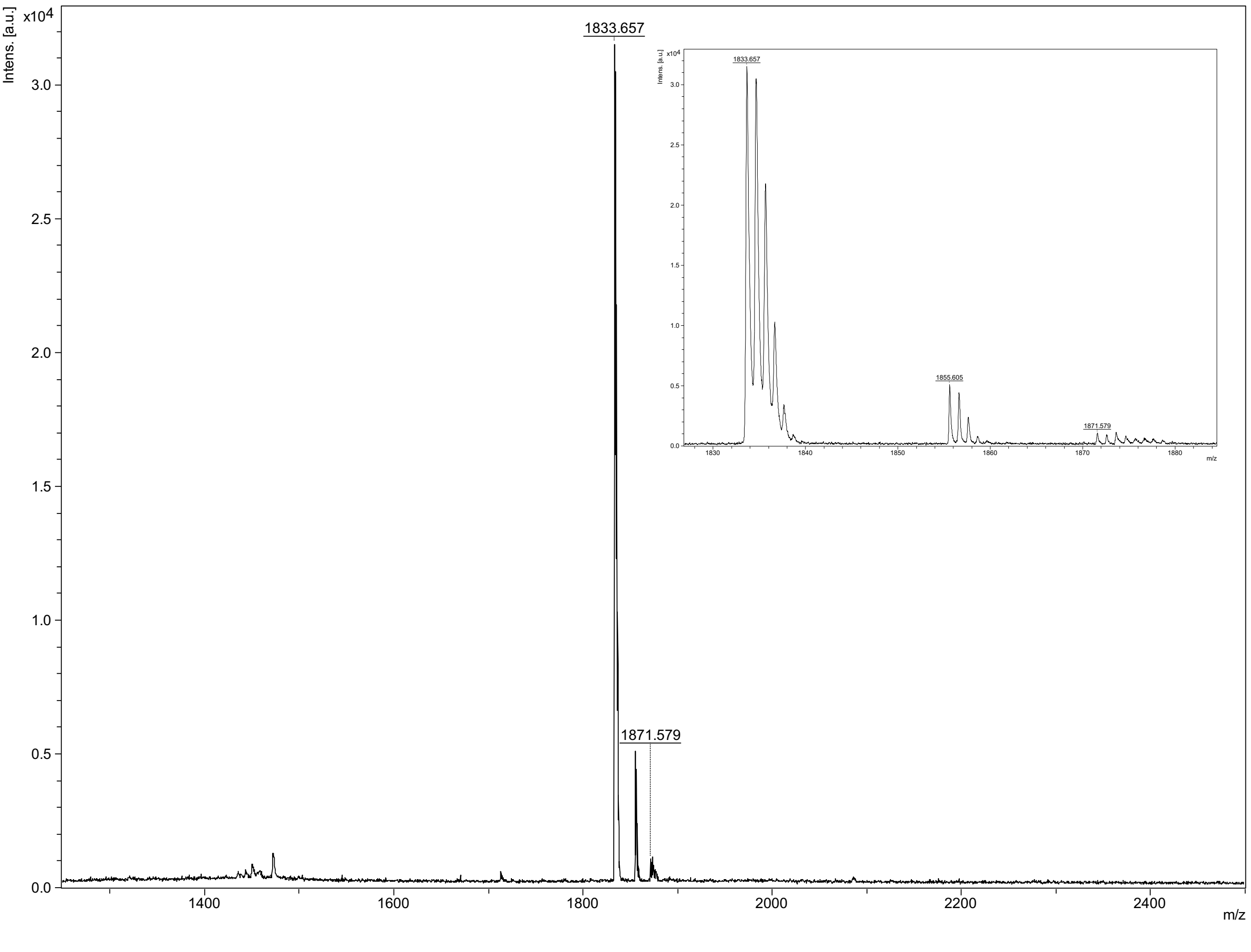

x10^4
Intens. [a.u.]
1833.657
1871.579
1855.605
m/z

Y11

Sequence: 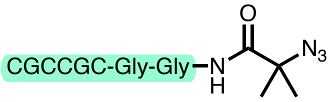

Chemical Formula: $C_{70}H_{92}N_{40}O_{21}$, Exact Mass: 1828.74
LC-MS (ESI) RT = 1.12 min, m/z found: 915.67 $[M+2H]^{2+}$; calc. 915.38 $[M+2H]^{2+}$
MALDI-TOF m/z found 1829.847 $[M+H]^{+}$; calc. 1829.74 $[M+H]^{+}$

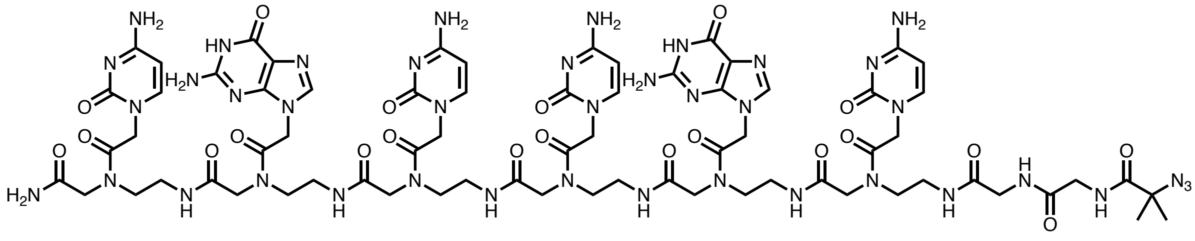

m/z: 1828.74 (100.0%), 1829.74 (77.6%), 1830.74 (44.6%)

Sequence of impurity 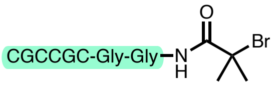

Chemical Formula: $C_{70}H_{92}BrN_{37}O_{21}$, Exact Mass: 1865.65
LC-MS (ESI) RT = 1.12 min, m/z found: 935.00 $[M+2H]^{2+}$; calc. 934.83 $[M+2H]^{2+}$
MALDI-TOF m/z found 1866.84 $[M+H]^{+}$; calc. 1866.65 $[M+H]^{+}$

impurity

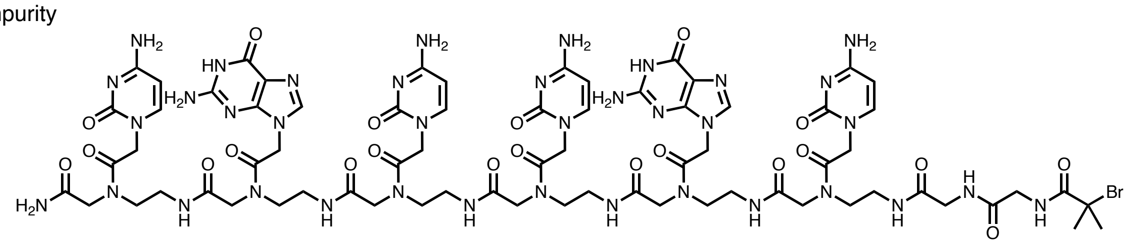

m/z: 1865.65 (100.0%), 1867.64 (98.2%), 1868.65 (83.3%), 1866.65 (77.6%)

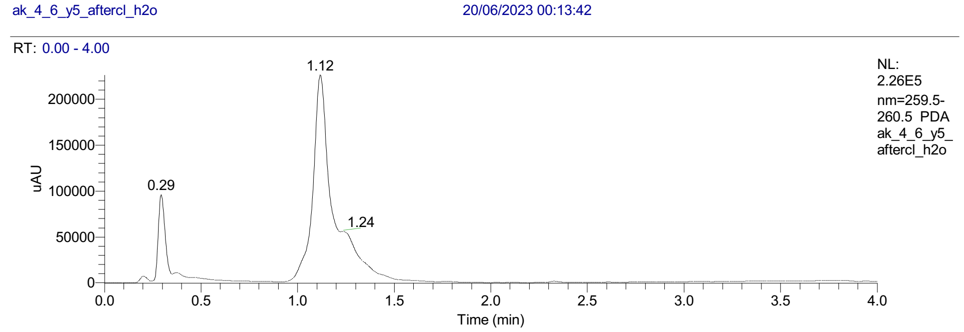

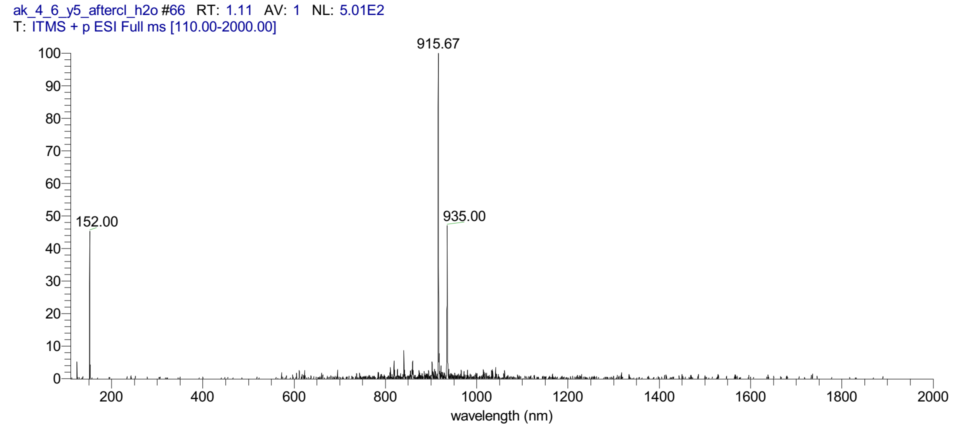

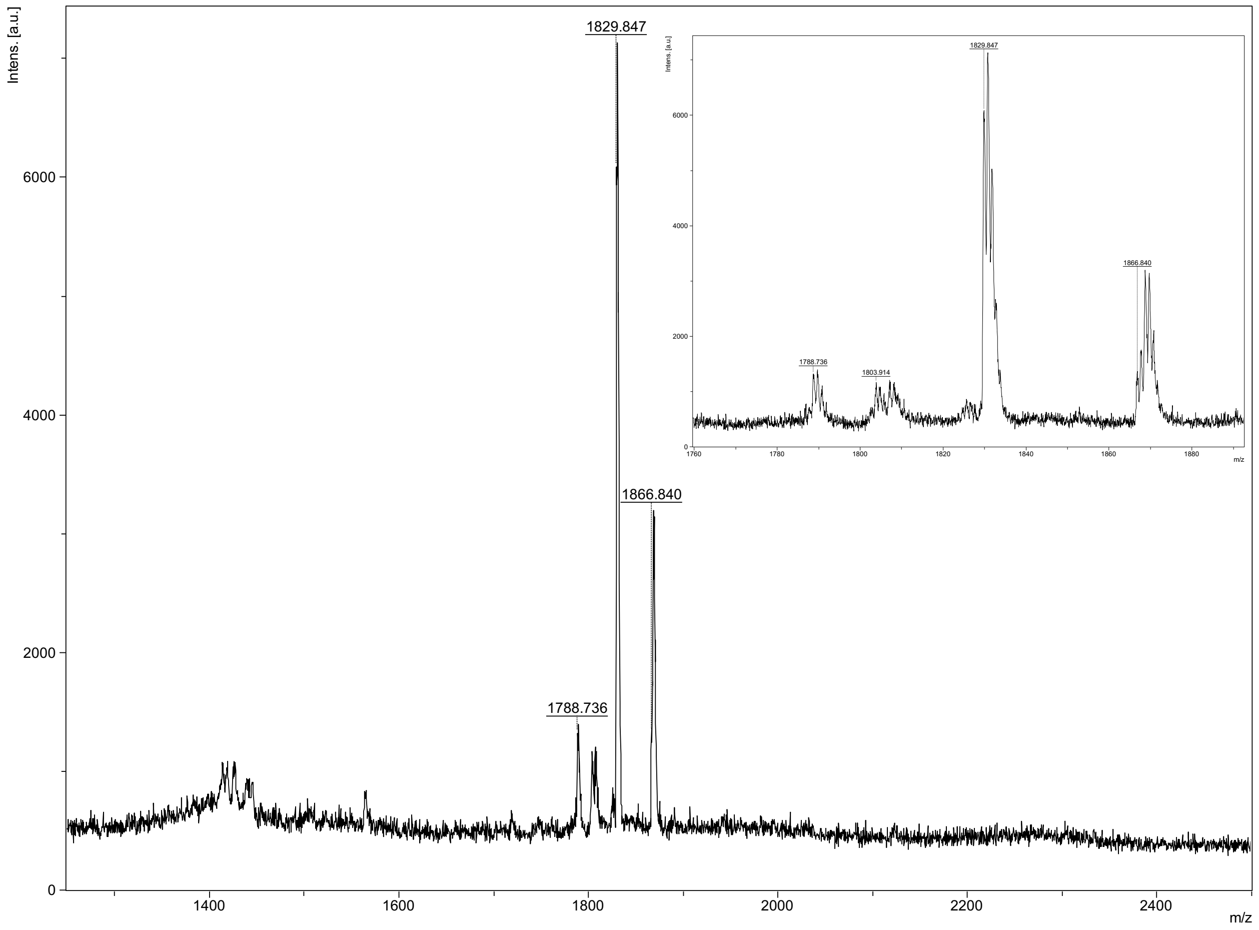

Intens. [a.u.]
6000
4000
2000
0
1400
1600
1800
2000
2200
2400
m/z
1829.847
1866.840
1788.736
1803.914
1760
1780
1800
1820
1840
1860
1880

Y12

Sequence: CGCCGC-Gly-Gly-Gly-NH_2

Chemical Formula: $C_{68}H_{90}N_{38}O_{21}$, Exact Mass: 1774.71

LC-MS (ESI) RT = 0.22 min, m/z found: 888.67 $[M+2H]^{2+}$; calc. 888.36 $[M+2H]^{2+}$

MALDI-TOF m/z found 1775.70 $[M+H]^{+}$, 1797.69 $[M+Na]^{+}$; calc. 1775.72 $[M+H]^{+}$, 1797.70 $[M+Na]^{+}$

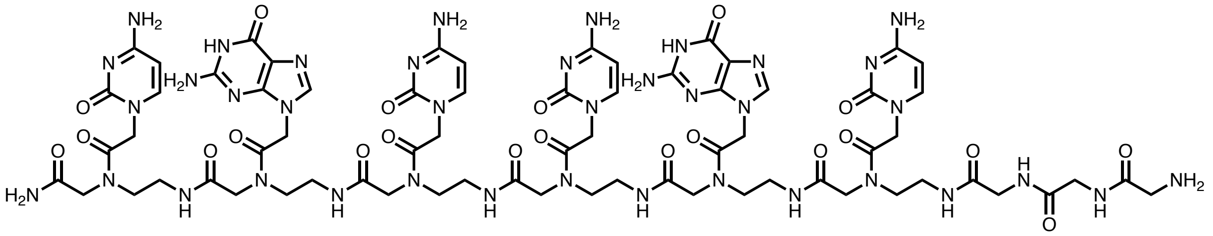

m/z: 1774.71 (100.0%), 1775.72 (75.4%), 1776.72 (32.6%)

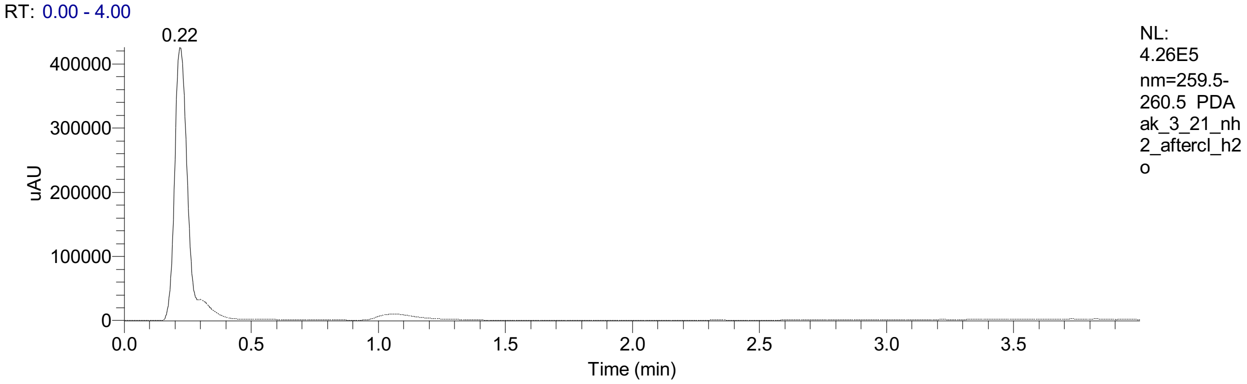

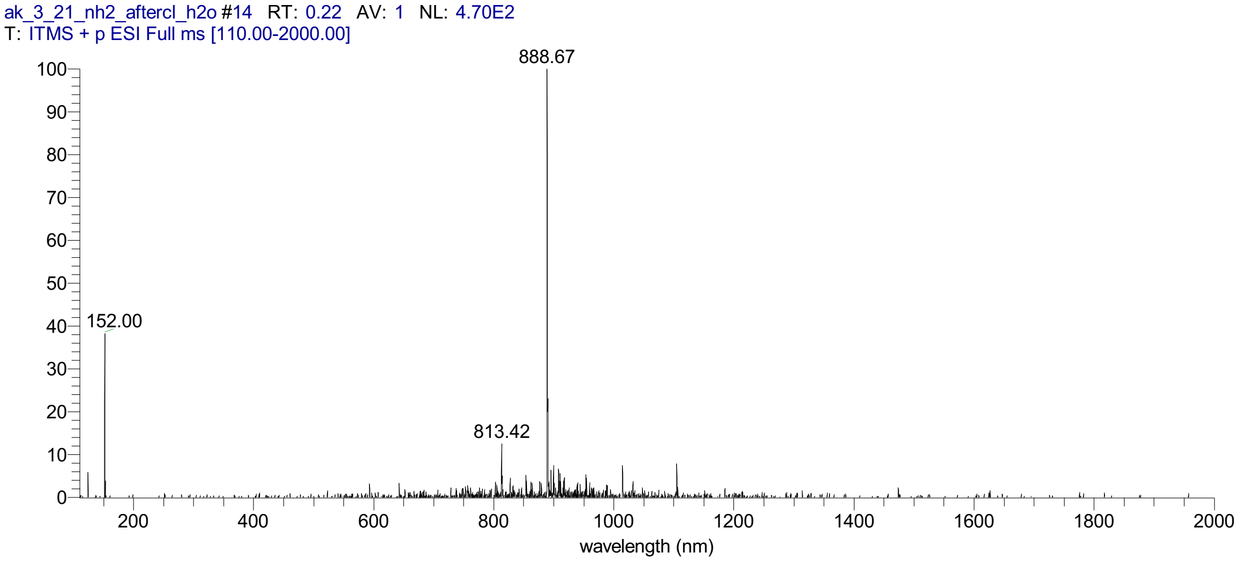

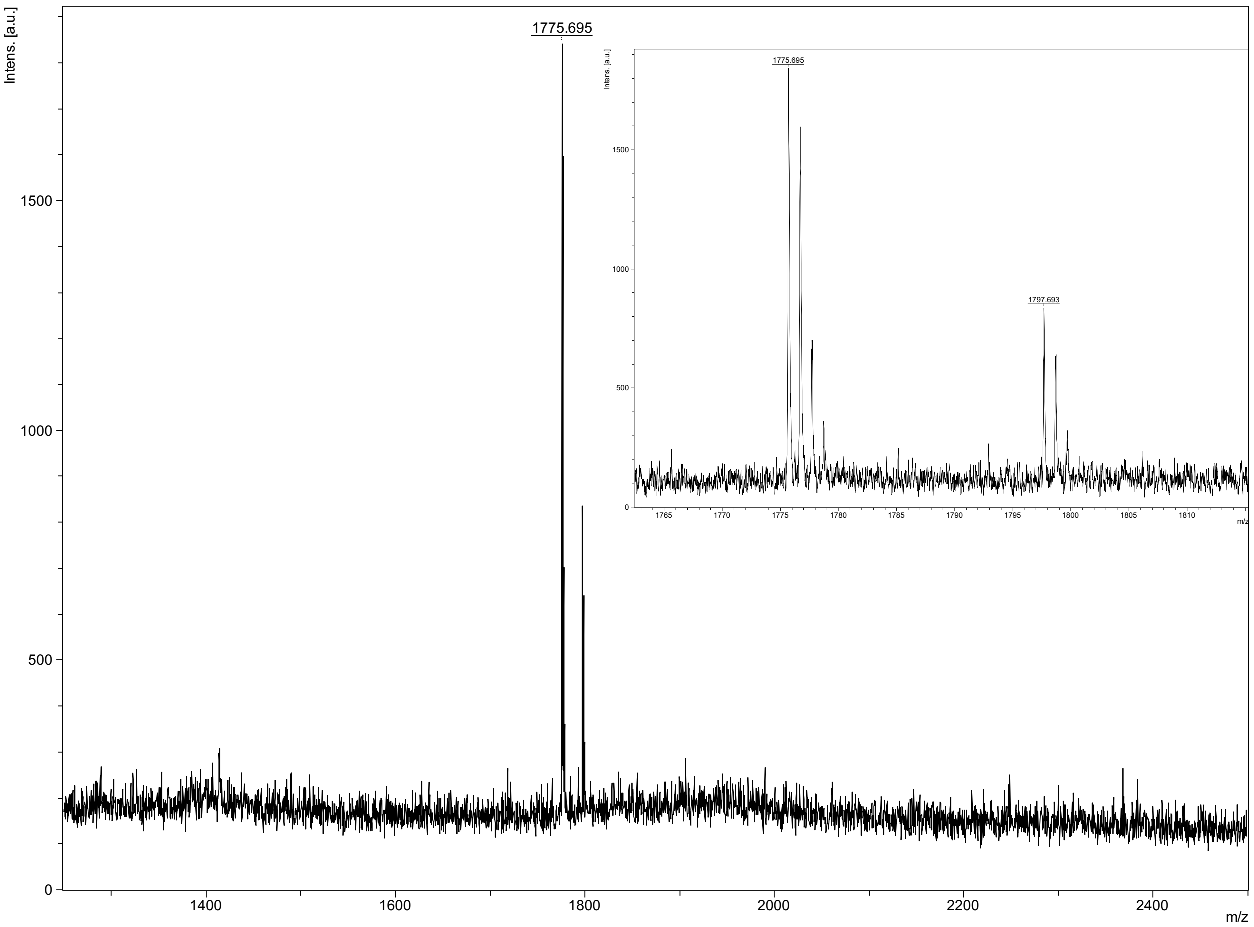
Intens. [a.u.]
1775.695
1500
1000
500
0
1400
1600
1800
2000
2200
2400
m/z
1797.693
1765
1770
1775
1780
1785
1790
1795
1800
1805
1810

Y13

Sequence: 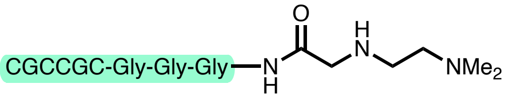

Chemical Formula: $C_{74}H_{102}N_{40}O_{22}$, Exact Mass: 1902.81
LC-MS (ESI) RT = 0.29 min, m/z found: 952.67 $[M+2H]^{2+}$; calc. 952.41 $[M+2H]^{2+}$
MALDI-TOF m/z found 1903.82 $[M+H]^{+}$; calc. 1903.82 $[M+H]^{+}$

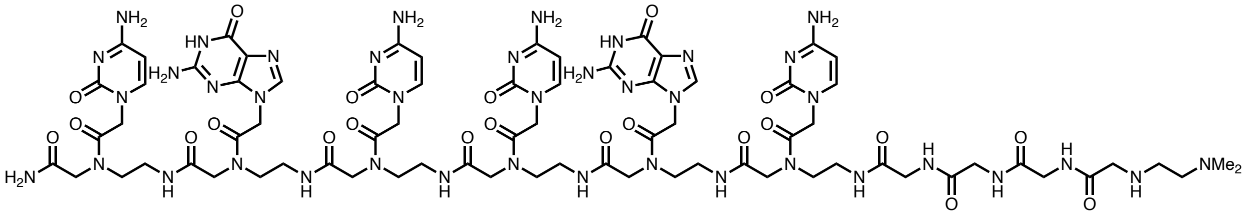

m/z: 1902.81 (100.0%), 1903.81 (95.6%), 1904.82 (33.2%)

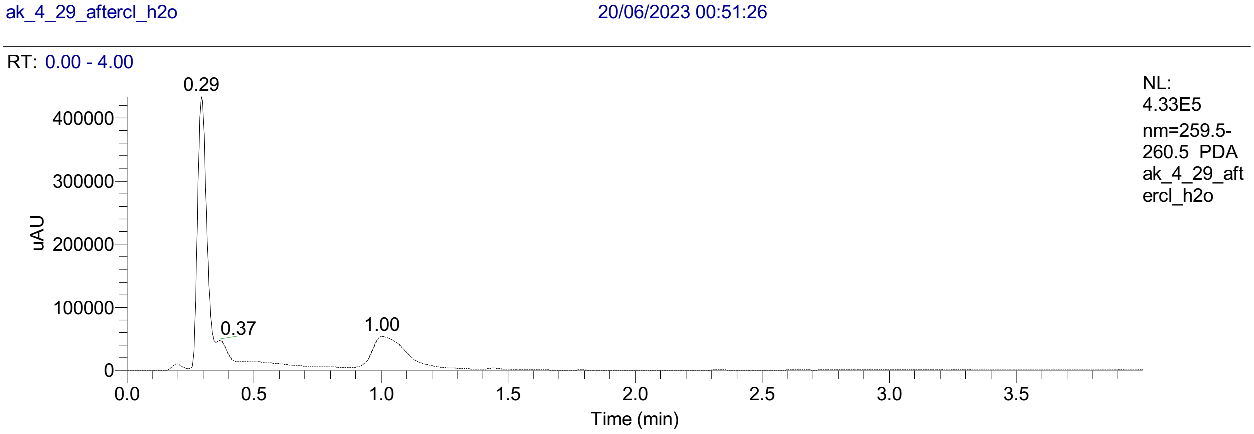

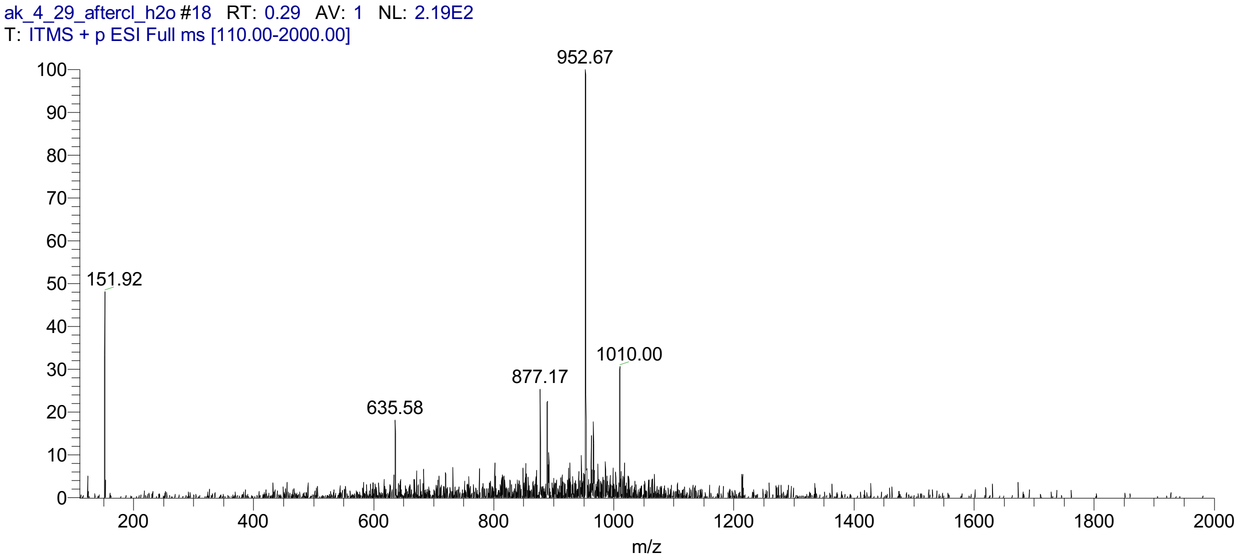

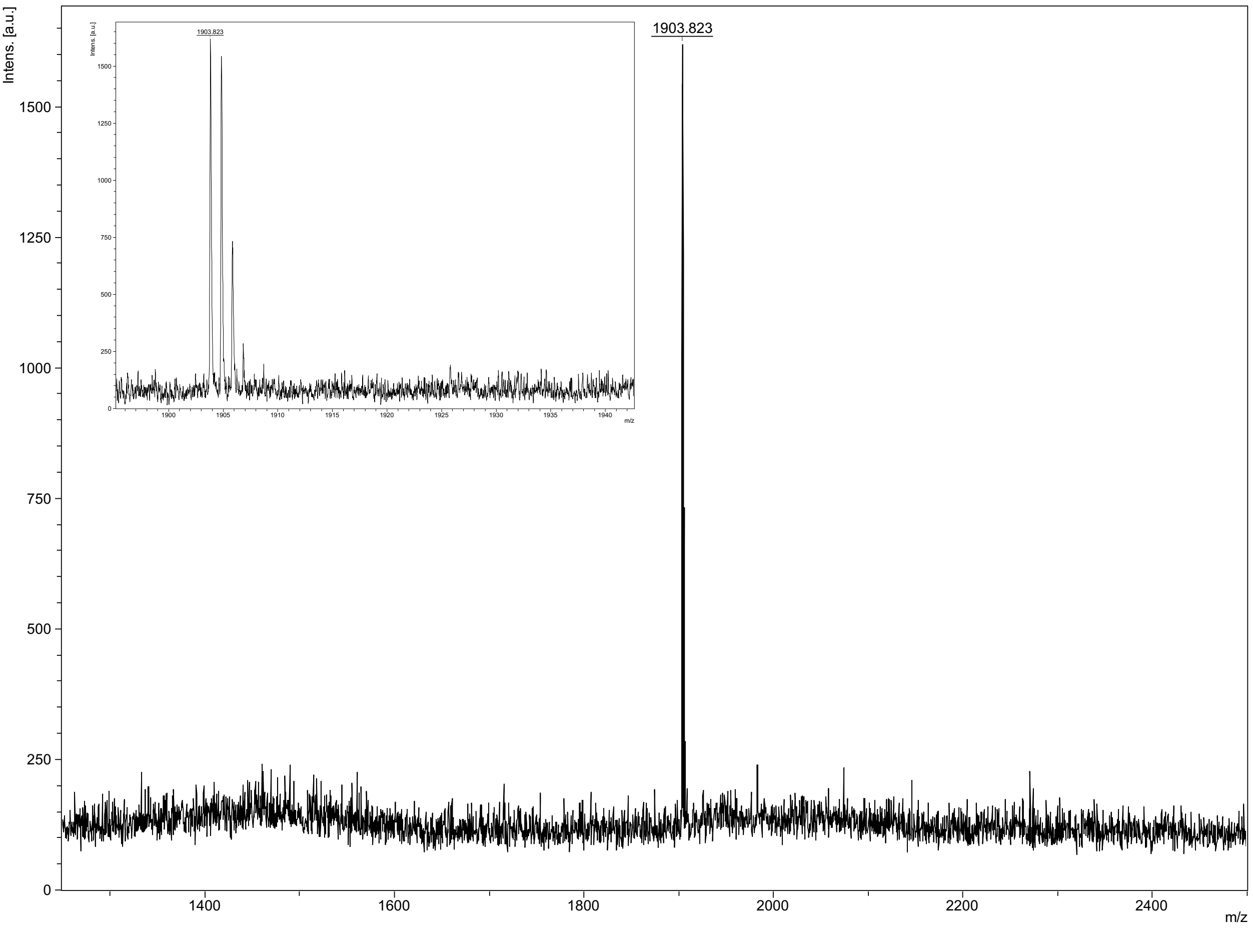

Intens. [a.u.]
1903.823
1500
1250
1000
750
500
250
0
1400
1600
1800
2000
2200
2400
m/z

Y14

Sequence: CGCCGC-Gly-Gly-Gly-Glu—NHAc

Chemical Formula: $C_{75}H_{99}N_{39}O_{25}$, Exact Mass: 1945.77

LC-MS (ESI) RT = 1.05 min, m/z found: 974.50 $[M+2H]^{2+}$; calc. 973.89 $[M+2H]^{2+}$

MALDI-TOF m/z found 1946.97 $[M+H]^{+}$, 1968.95 $[M+Na]^{+}$; calc. 1946.78 $[M+H]^{+}$, 1968.76 $[M+Na]^{+}$

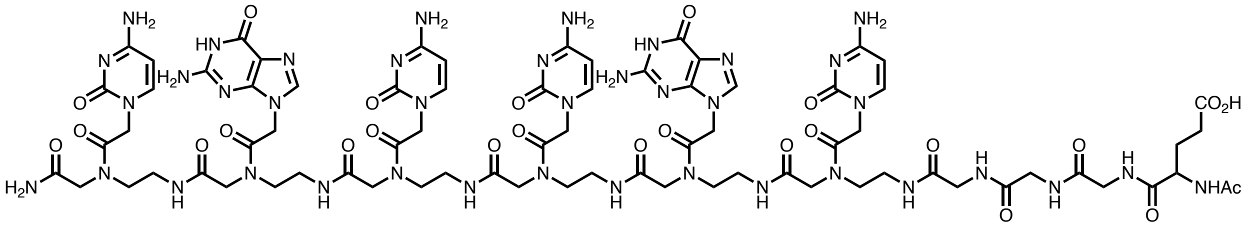

m/z: 1945.77 (100.0%), 1946.77 (83.2%), 1947.77 (49.6%)

\\lcqfleetpc\data\...\ak_5_3_glu_aftercl 31/03/2023 19:28:37

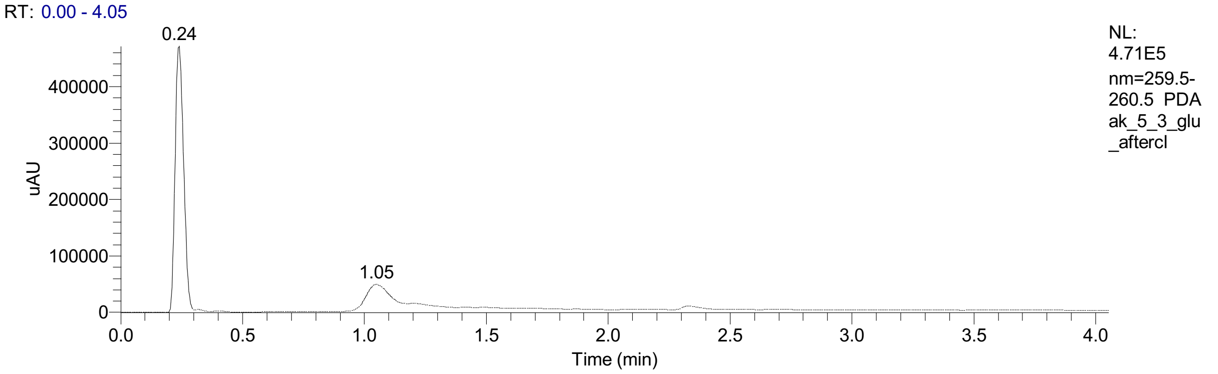

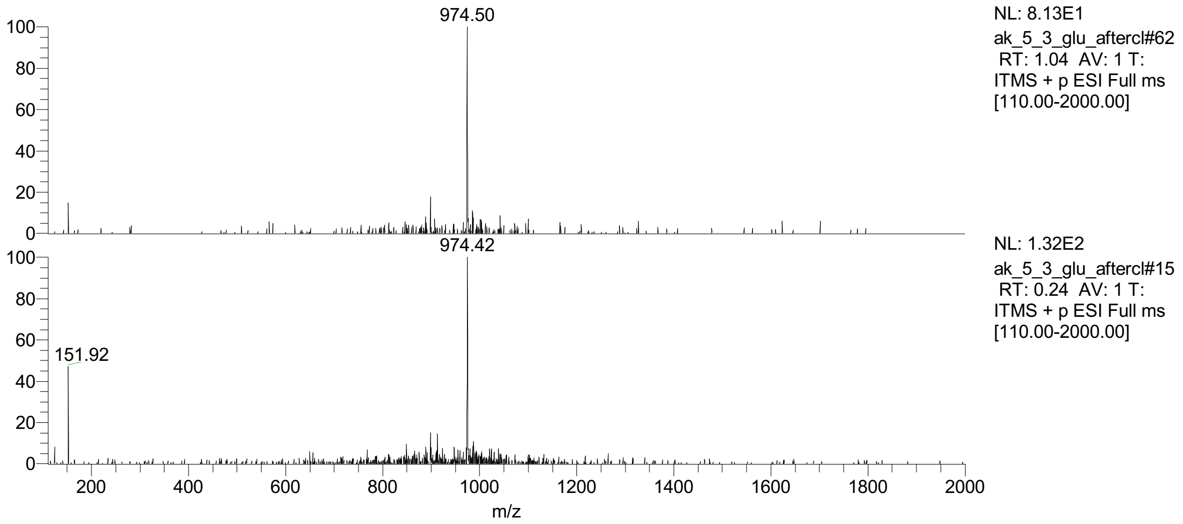

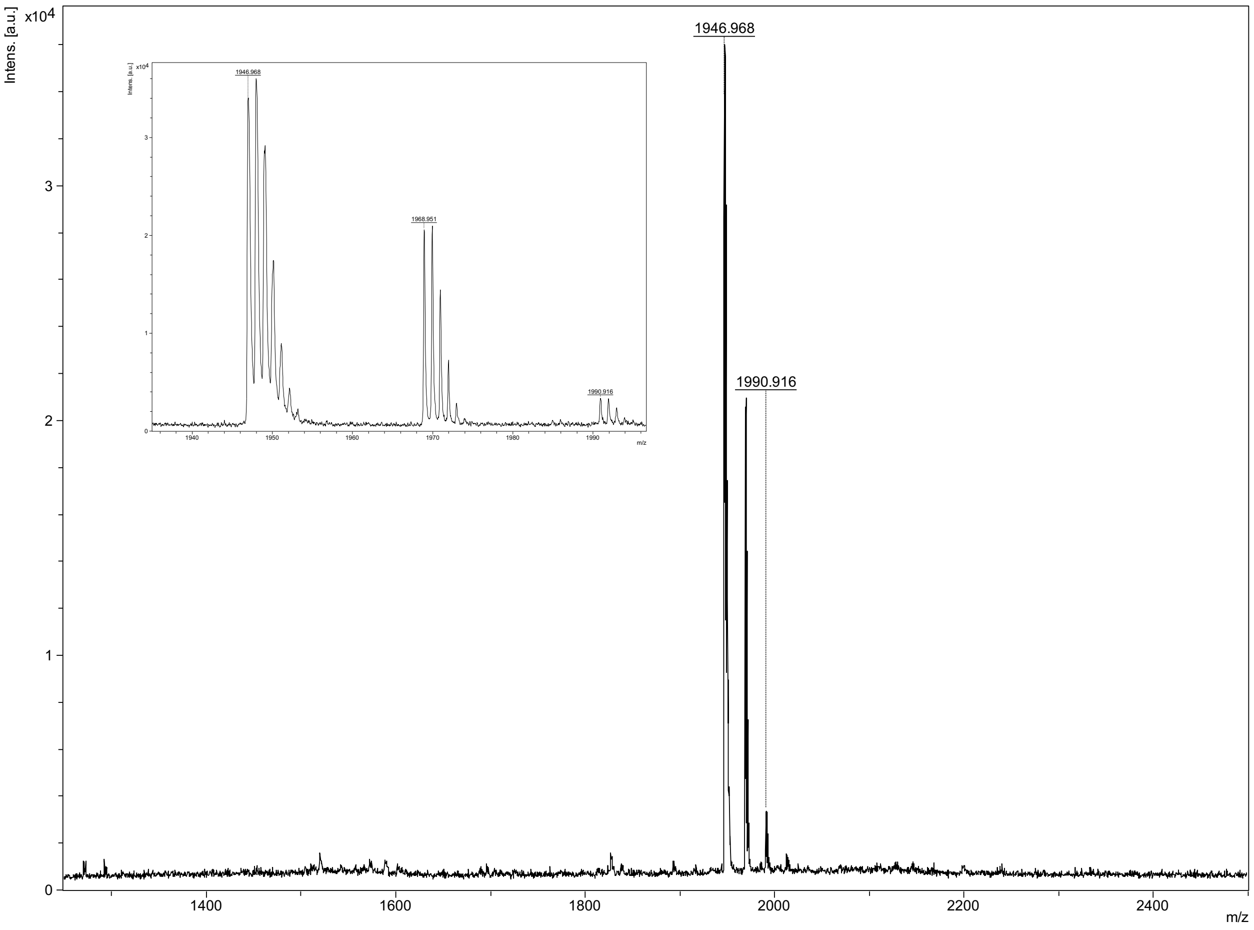

Intens. [a.u.]
x10^4
1946.968
1990.916
1968.951
m/z

Y15

Sequence: CGCCGC-Gly-Gly-Gly-Trp—NHAc

Chemical Formula: $C_{81}H_{102}N_{40}O_{23}$, Exact Mass: 2002.80
LC-MS (ESI) RT = 1.14 min, m/z found: 1002.92 $[M+2H]^{2+}$; 1002.91$[M+2H]^{2+}$
MALDI-TOF m/z found 2004.10 $[M+H]^{+}$, 2026.08 $[M+Na]^{+}$, 2048.05 $[M+HCO_2]^{+}$; calc. 2004.81 $[M+H]^{+}$, 2026.80 $[M+Na]^{+}$, 2047.80 $[M+HCO_2]^{+}$

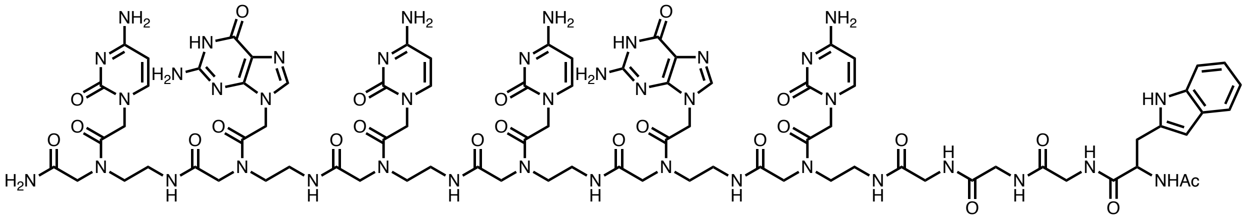

m/z: 2002.80 (100.0%), 2003.81 (89.7%), 2004.81 (44.7%)

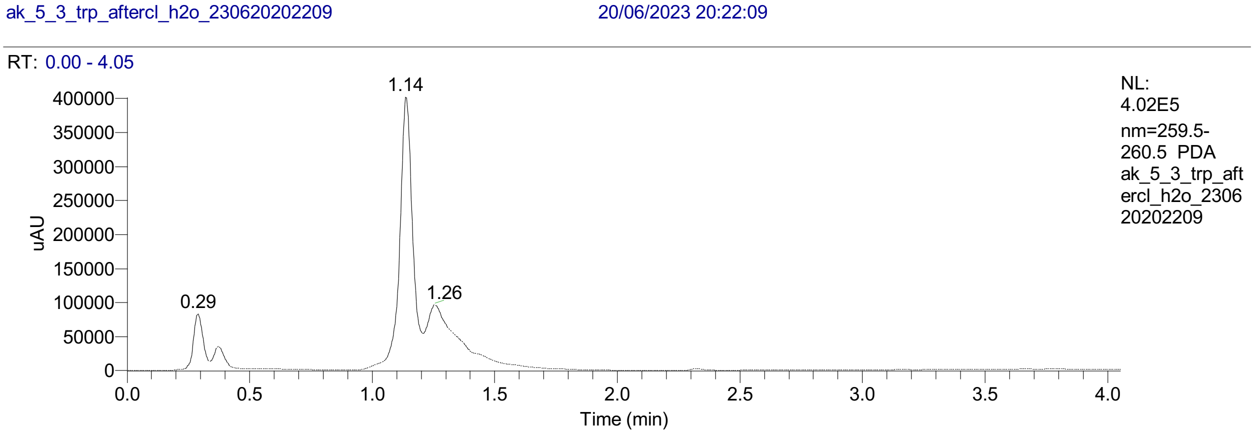

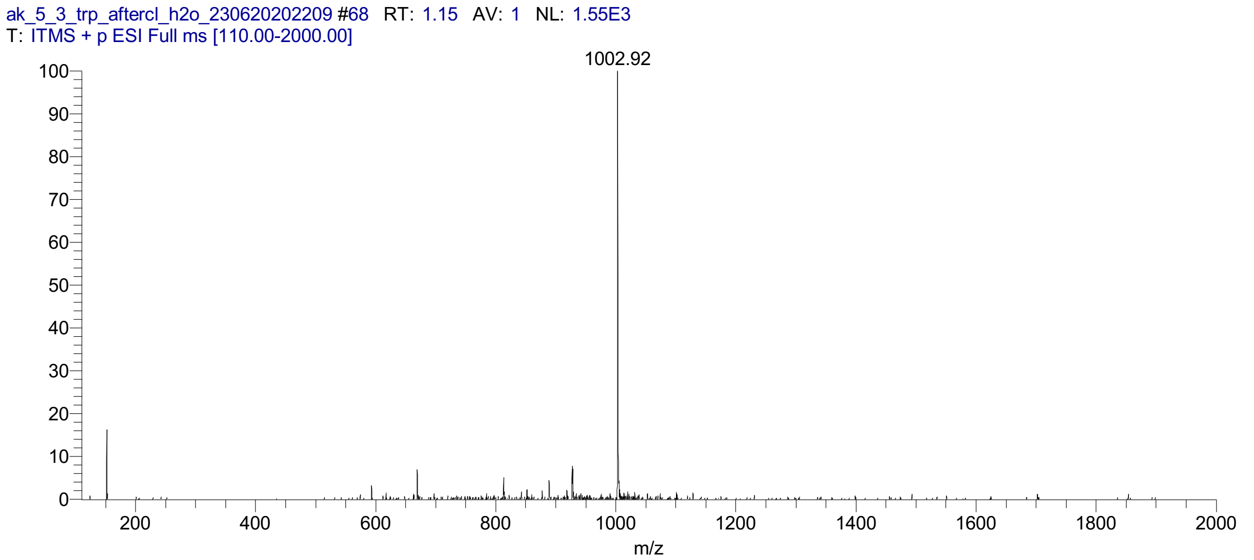

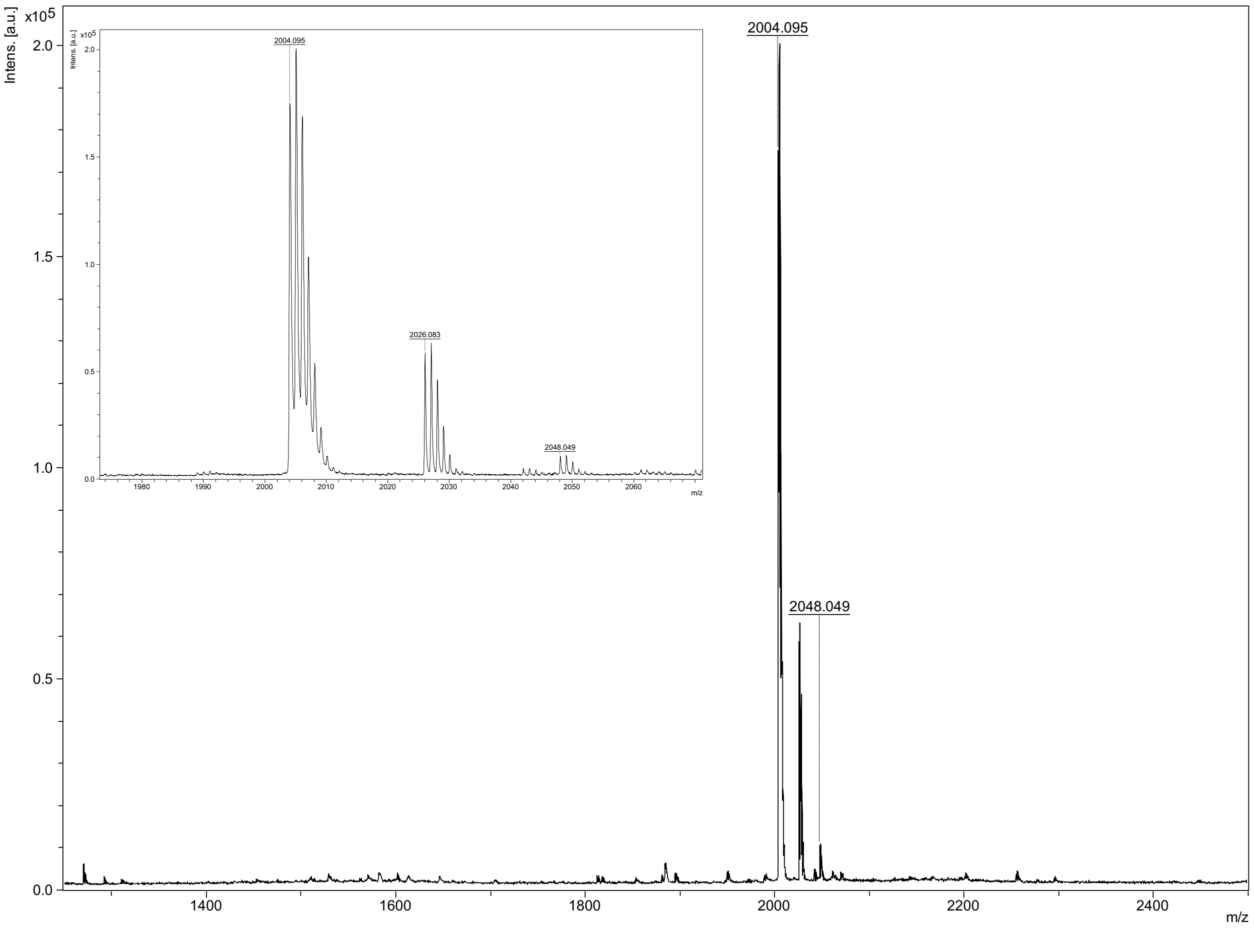
x10^5
Intens. [a.u.]
2004.095
2026.083
2048.049
m/z

Y16

Sequence: CGCCGC-Gly-Gly-Gly-Tyr—NHAc

Chemical Formula: $C_{79}H_{101}N_{39}O_{24}$, Exact Mass: 1979.79

LC-MS (ESI) RT = 1.12 min, m/z found: 991.50 $[M+2H]^{2+}$, 661.42 $[M+3H]^{3+}$; calc. 991.40 $[M+2H]^{2+}$, 660.94 $[M+3H]^{3+}$

MALDI-TOF m/z found 1981.14 $[M+H]^{+}$, 2003.13 $[M+Na]^{+}$, 2025.11 $[M+HCO_2]^{+}$; calc. 1980.80 $[M+H]^{+}$, 2002.78 $[M+Na]^{+}$, 2024.78 $[M+HCO_2]^{+}$

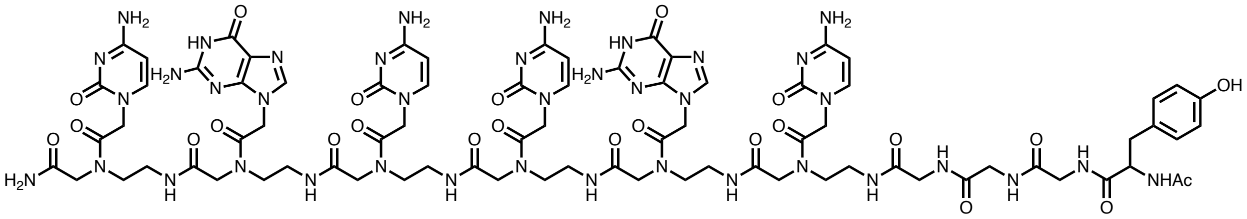

m/z: 1980.79 (100.0%), 1979.79 (98.1%), 1981.79 (52.6%)

\\lcqfleetpc\data\...\ak_5_3_aftercl_h2o 02/05/2023 10:42:16

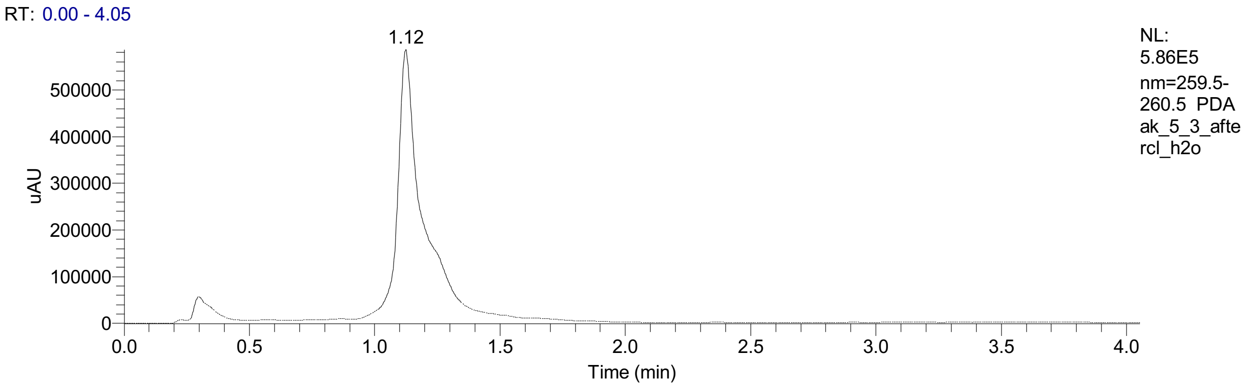

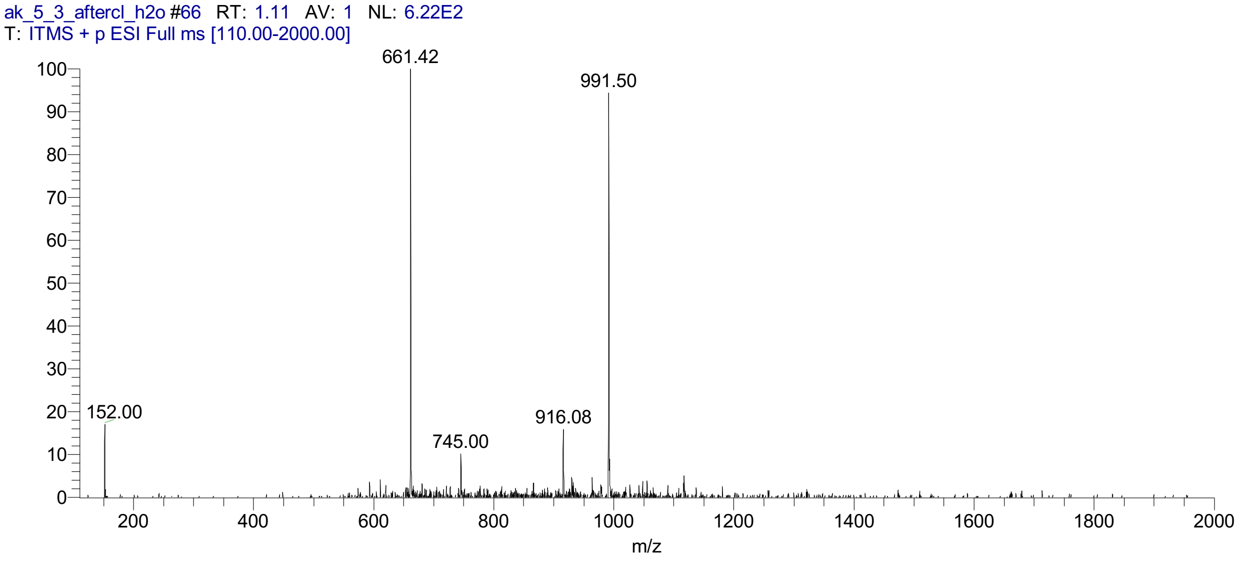

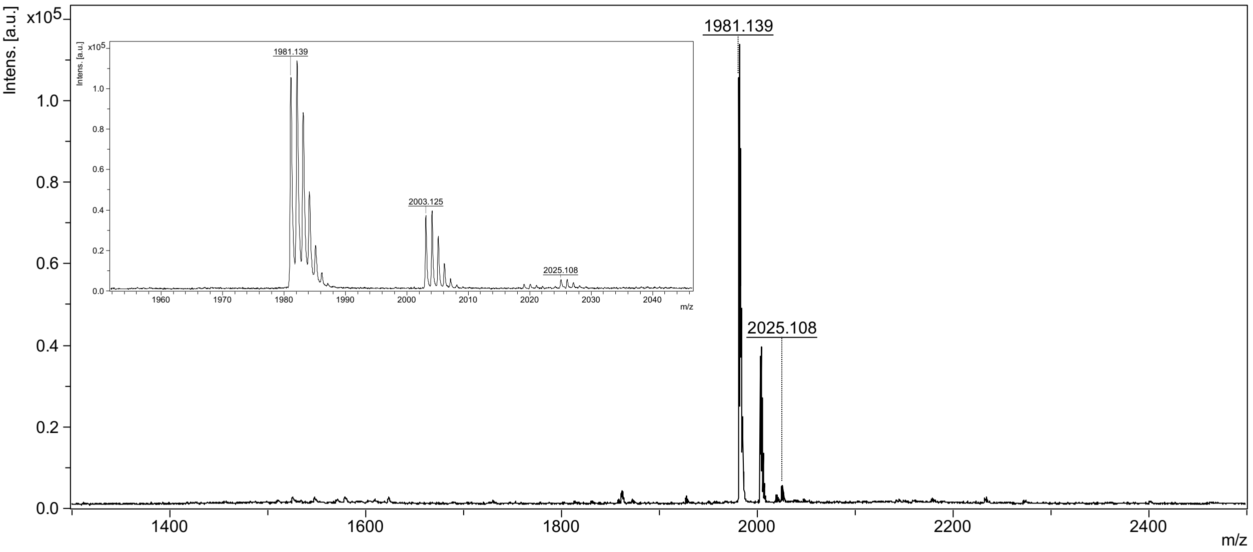

Intens. [a.u.]
x10^5
1.0
0.8
0.6
0.4
0.2
0.0
1981.139
2025.108
2003.125
1400
1600
1800
2000
2200
2400
m/z

Y17

Sequence: 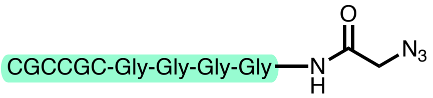

*__Y17__ contains a truncated PNA (missing one glycine, same as **Y3**) as an impurity.

Chemical Formula: $C_{72}H_{94}N_{42}O_{23}$, Exact Mass: 1914.75

LC-MS (ESI) RT = 0.28 min, m/z found: 958.75 $[M+2H]^{2+}$, 639.58 $[M+3H]^{3+}$; calc. 958.38 $[M+2H]^{2+}$, 639. 26 $[M+3H]^{3+}$

MALDI-TOF m/z found 1914.16 $[M+H]^{+}$; calc.: 1915.76 $[M+H]^{+}$

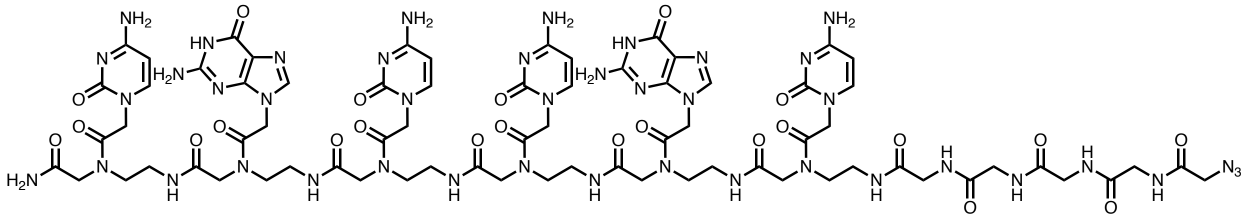

m/z: 1914.75 (100.0%), 1915.75 (79.8%), 1916.75 (46.9%)

F:\20250812\AK_061_221003202704 10/03/22 20:27:04

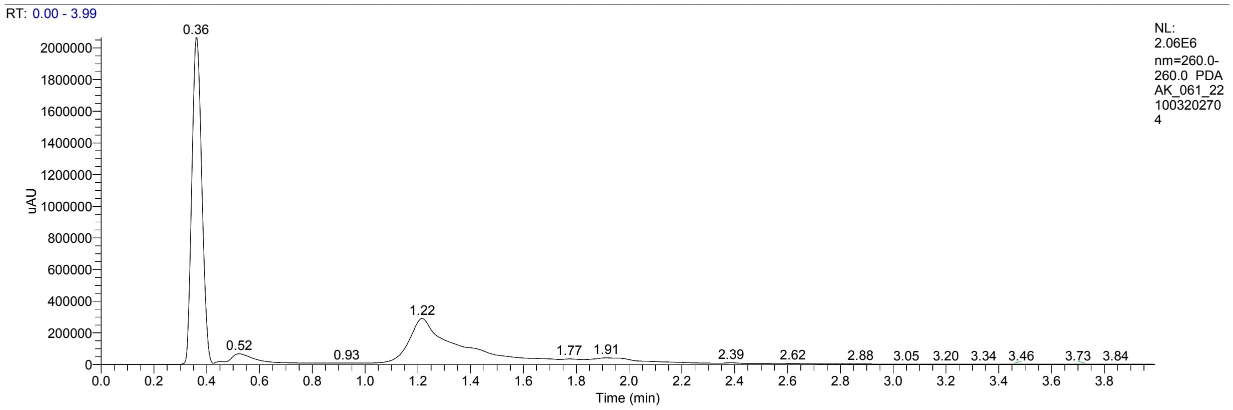

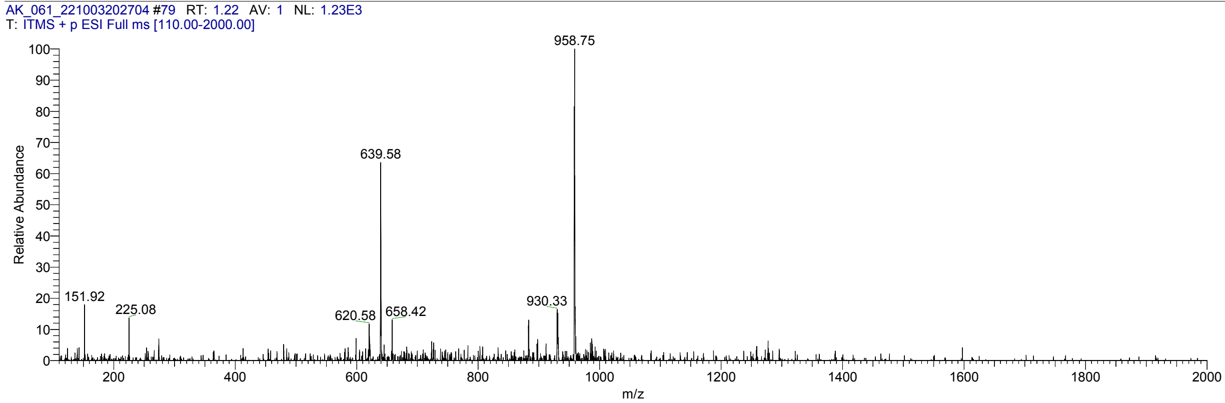

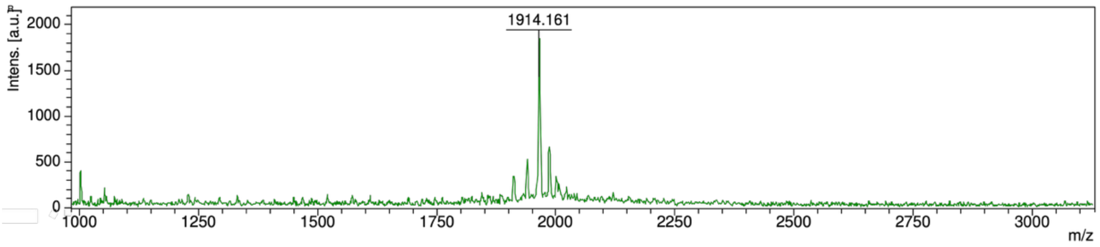

Y18

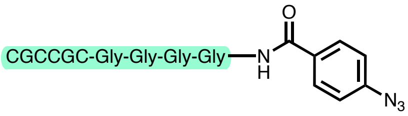

Sequence:

***Y18** contains a truncated PNA (missing one glycine) as an impurity.

Chemical Formula: $C_{77}H_{96}N_{42}O_{23}$, Exact Mass: 1976.76

LC-MS (ESI) RT = 1.45 min, m/z found: 989.67 $[M+2H]^{2+}$, 660.17 $[M+3H]^{3+}$; calc. 989.89 $[M+2H]^{2+}$, 660.26 $[M+3H]^{3+}$

MALDI-TOF m/z found 1950.67 $[M-N_2+2H]^{+}$; calc. 1950.77 $[M-N_2+2H]^{+}$

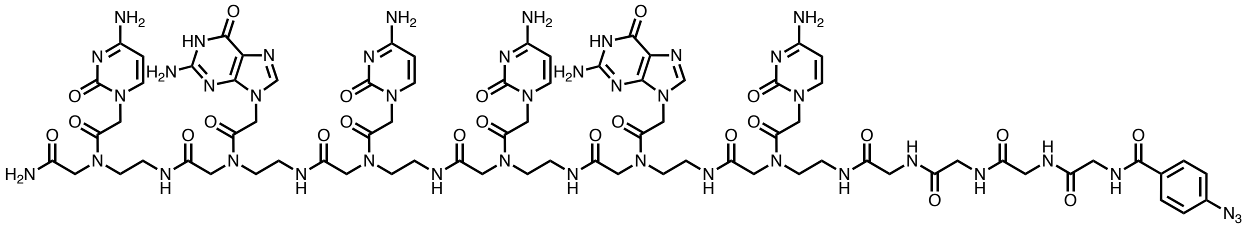

m/z: 1976.76 (100.0%), 1977.77 (85.3%), 1978.77 (40.8%)

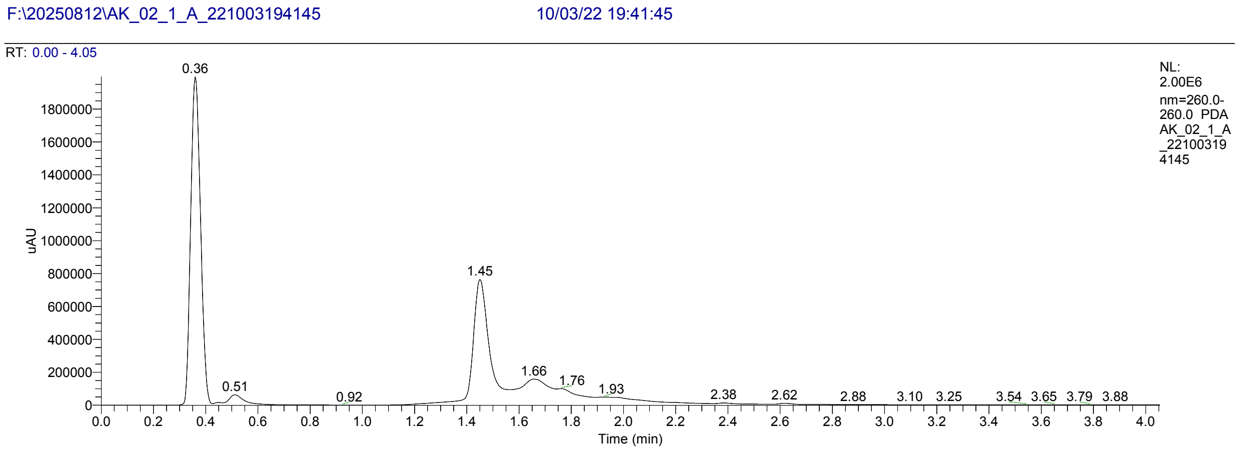

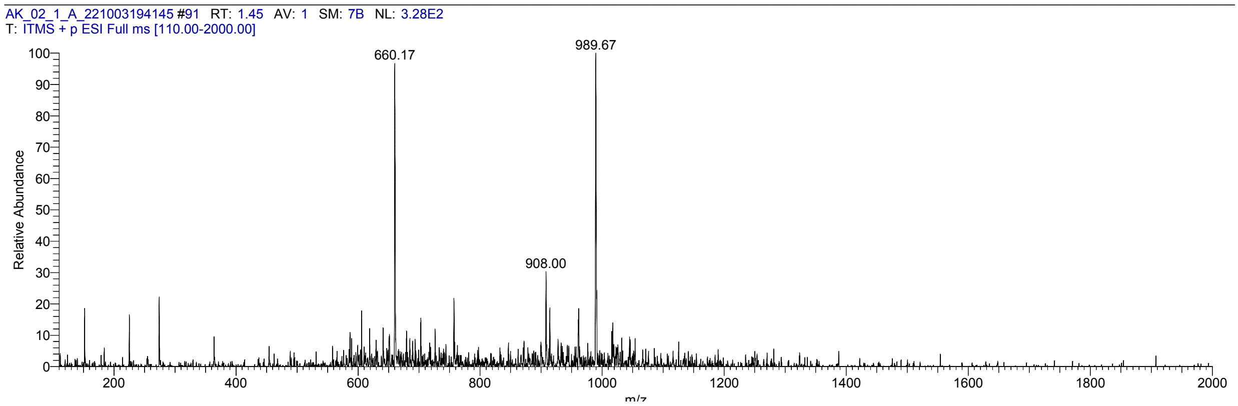

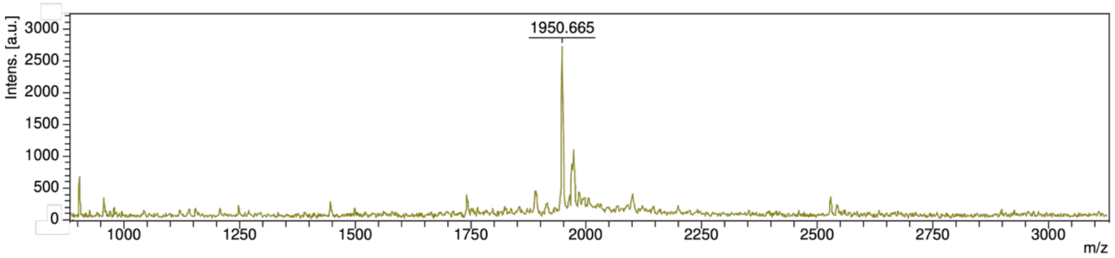

Y19

Sequence: 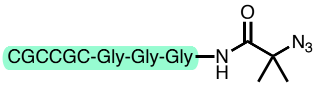

***Y19** contains **Y19’** as an impurity.

Chemical Formula: $C_{72}H_{95}N_{41}O_{22}$, Exact Mass: 1885.76

LC-MS (ESI) RT = 1.19 min, m/z found: 944.06 $[M+2H]^{2+}$, 629.92 $[M+3H]^{3+}$; calc. 943.89 $[M+2H]^{2+}$, 629.59 $[M+3H]^{3+}$

MALDI-TOF m/z found 1887.04 $[M+H]^{+}$; calc. 1886.76 $[M+H]^{+}$

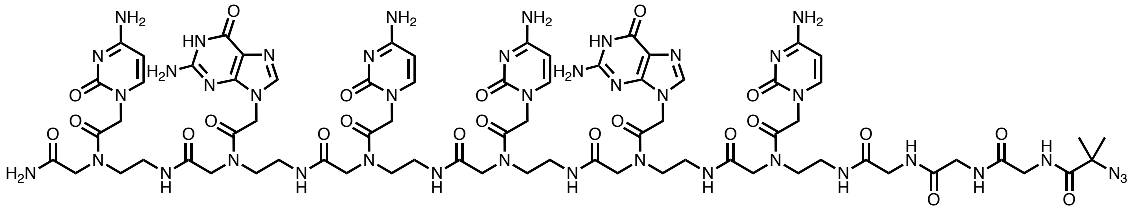

m/z: 1885.76 (100.0%), 1886.76 (79.8%), 1887.76 (46.4%)

Y19’

Sequence: 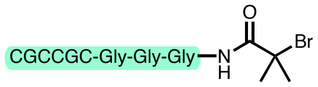

Chemical Formula: $C_{72}H_{95}BrN_{38}O_{22}$, Exact Mass: 1922.67

LC-MS (ESI) RT = 1.19 min, m/z found: 963.42 $[M+2H]^{2+}$, 642.67 $[M+3H]^{2+}$; calc. 963.34 $[M+2H]^{2}$, 642.56 $[M+3H]^{2+}$

MALDI-TOF m/z found 1925.98 $[M+H]^{+}$; calc. 1925.67 $[M+H]^{+}$

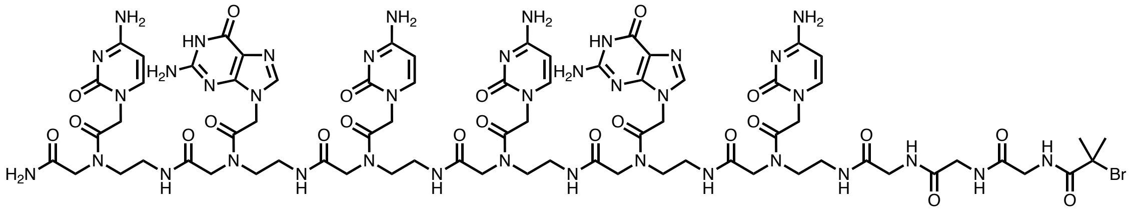

m/z: 1922.67 (100.0%), 1924.66 (98.2%), 1925.67 (86.1%), 1923.67 (79.8%)

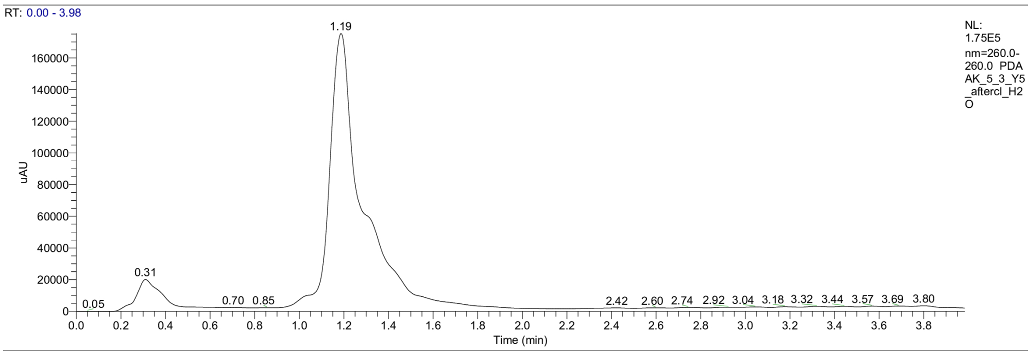

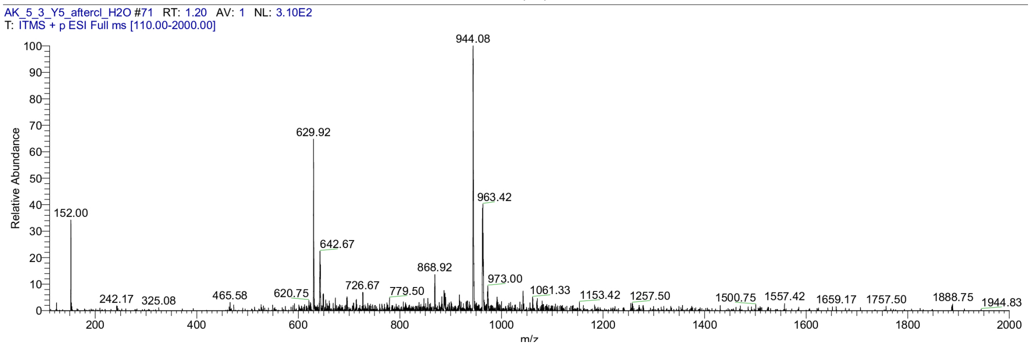

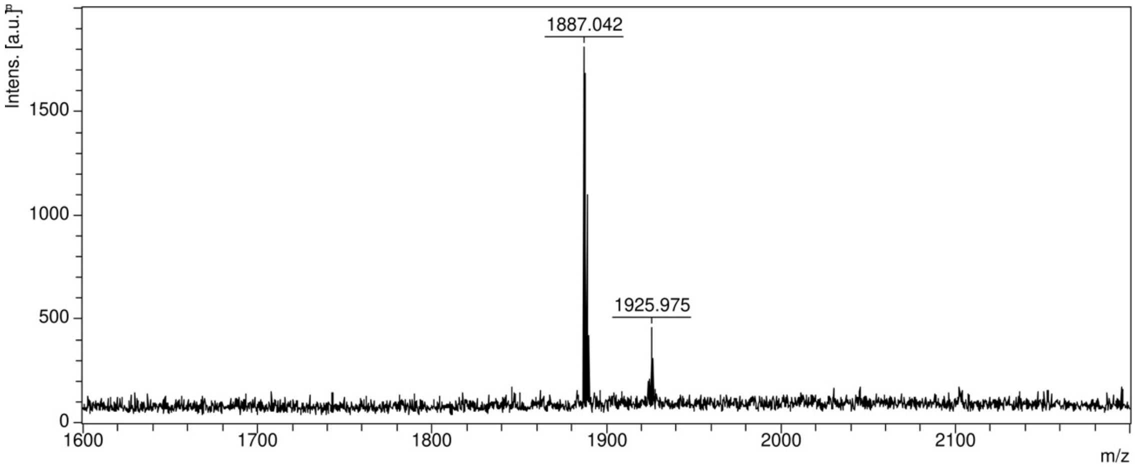

1887.042
1925.975
Intens. [a.u.]
0
500
1000
1500
1600
1700
1800
1900
2000
2100
m/z
