# Supplementary material for: One-pot multi-substrate screening of ligation reactions using PNA tags
Source: Chem Sci. 2026 Feb 20;17(15):7756–65. doi: 10.1039/d5sc08732e (PMC12937129; doi:10.1039/d5sc08732e)

Supplementary Information (SI) for Chemical Science.
This journal is © The Royal Society of Chemistry 2026

7. NMR spectra

^{1}H-NMR of **S1**

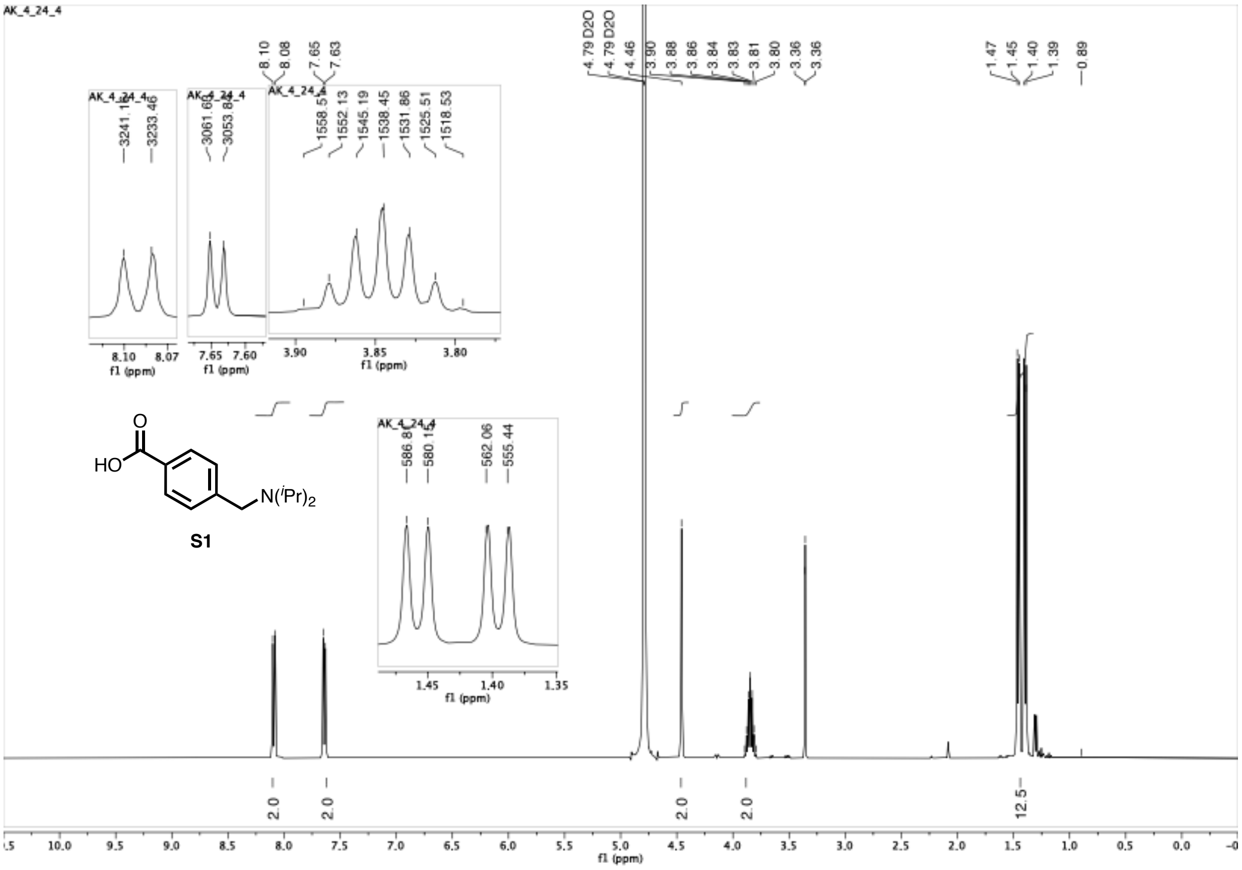

^{13}C{^{1}H}-NMR of **S1**

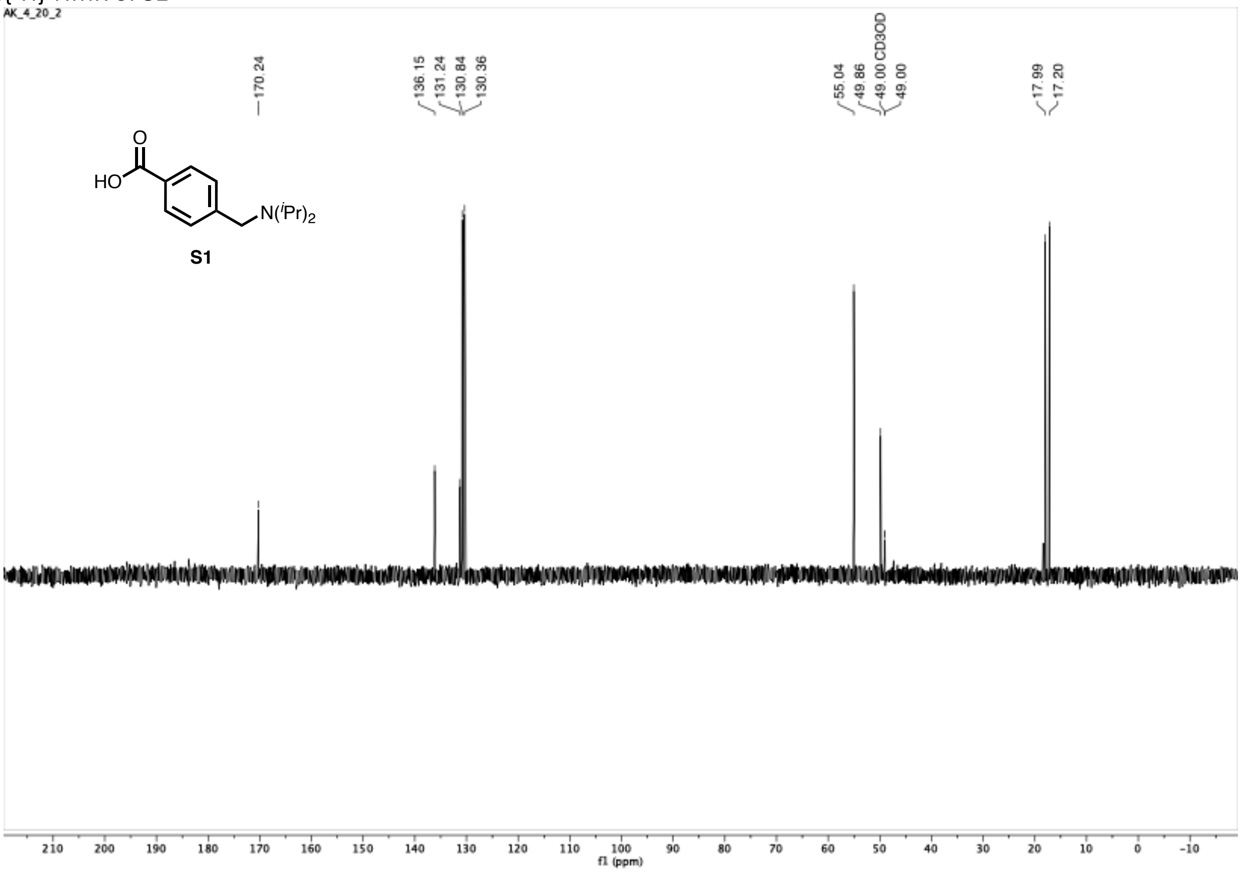

^{1}H-NMR of **S2**

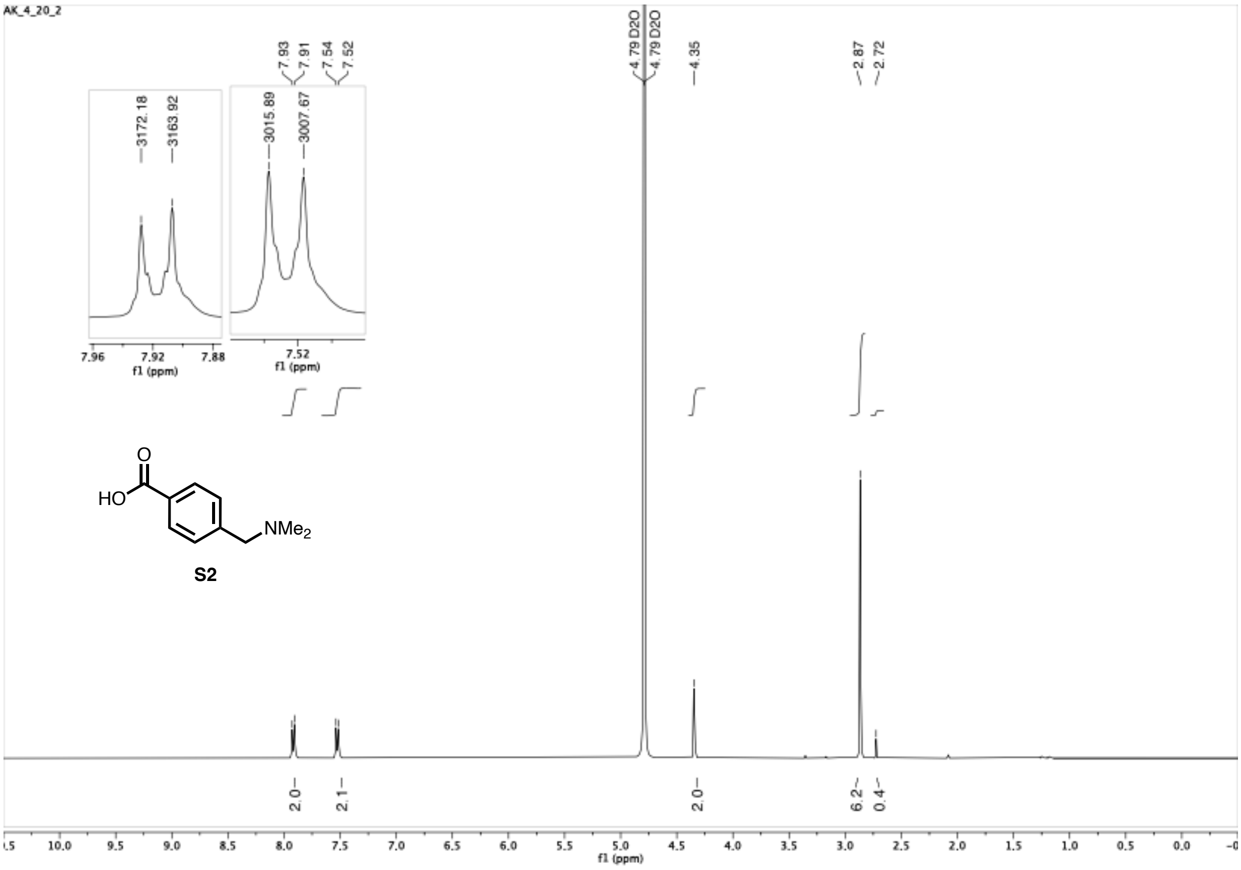

^{13}C{^{1}H}-NMR of **S2**

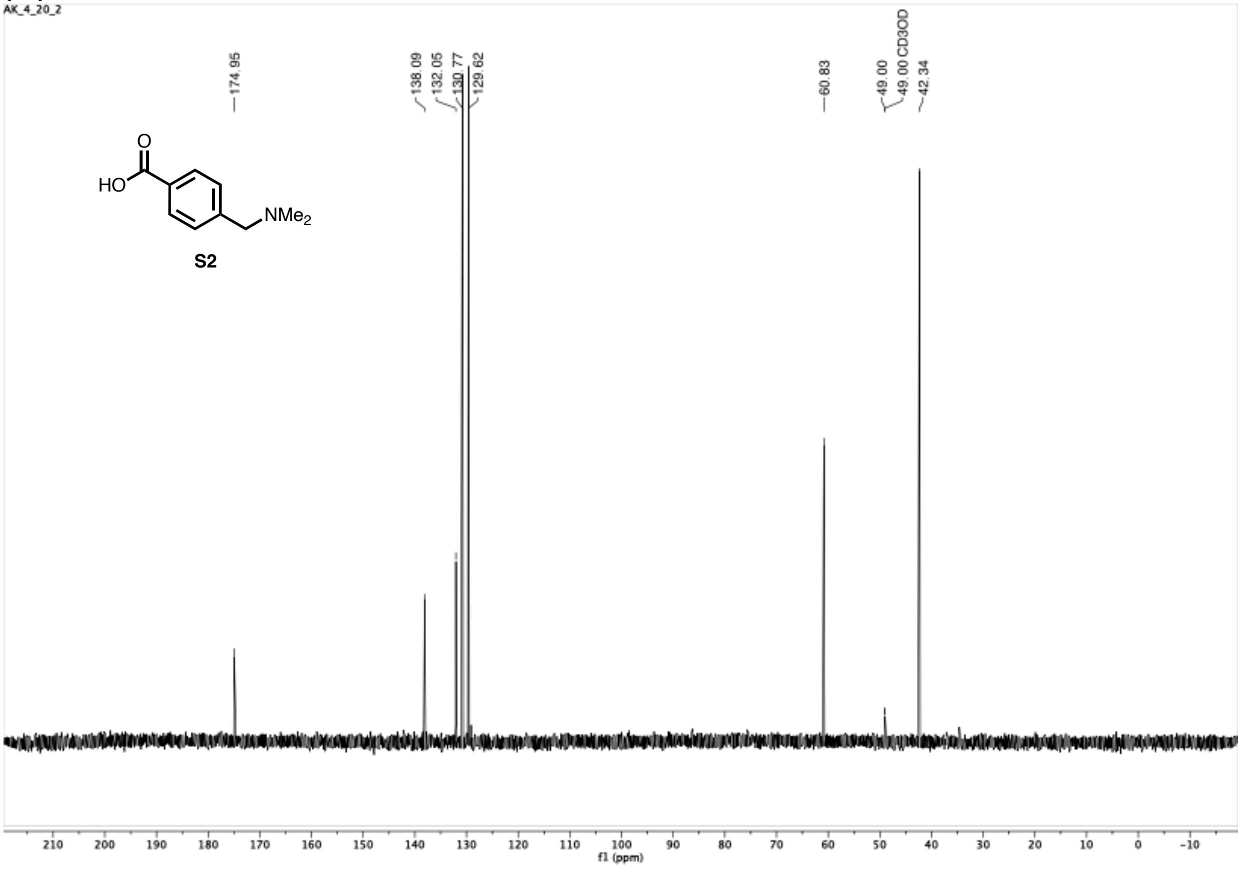

^{1}H-NMR of **S9**

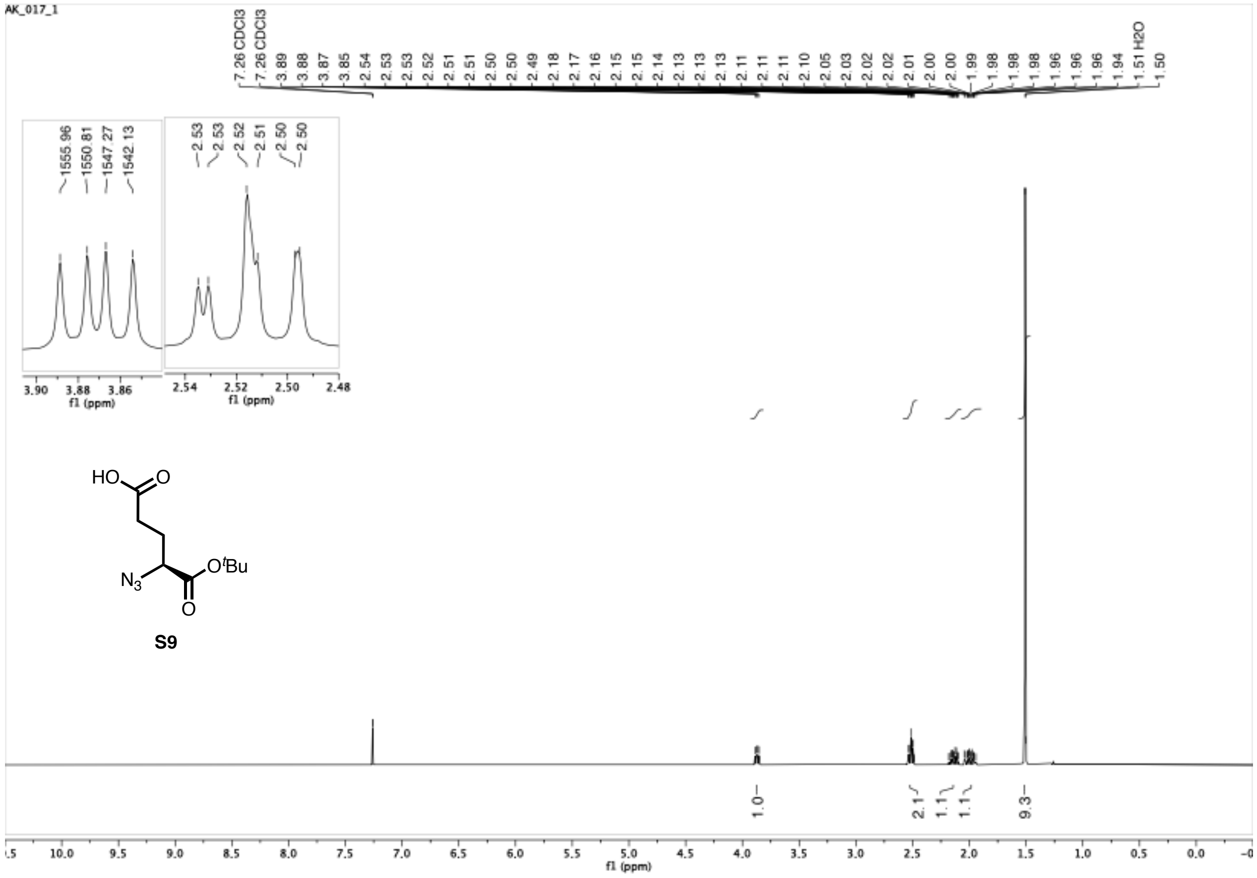

$^{13}C\{^{1}H\}$-NMR of **S9**

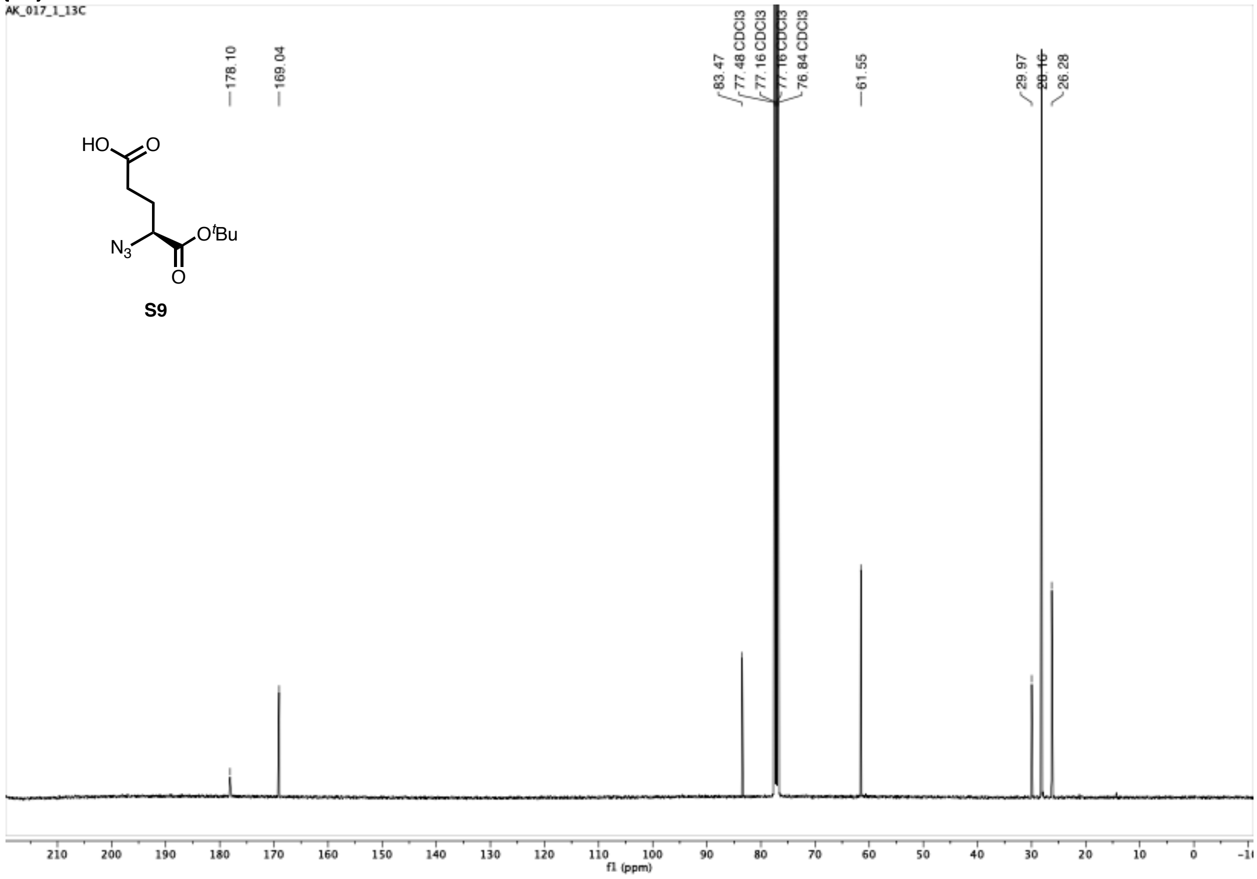

^{1}H-NMR of **S10**

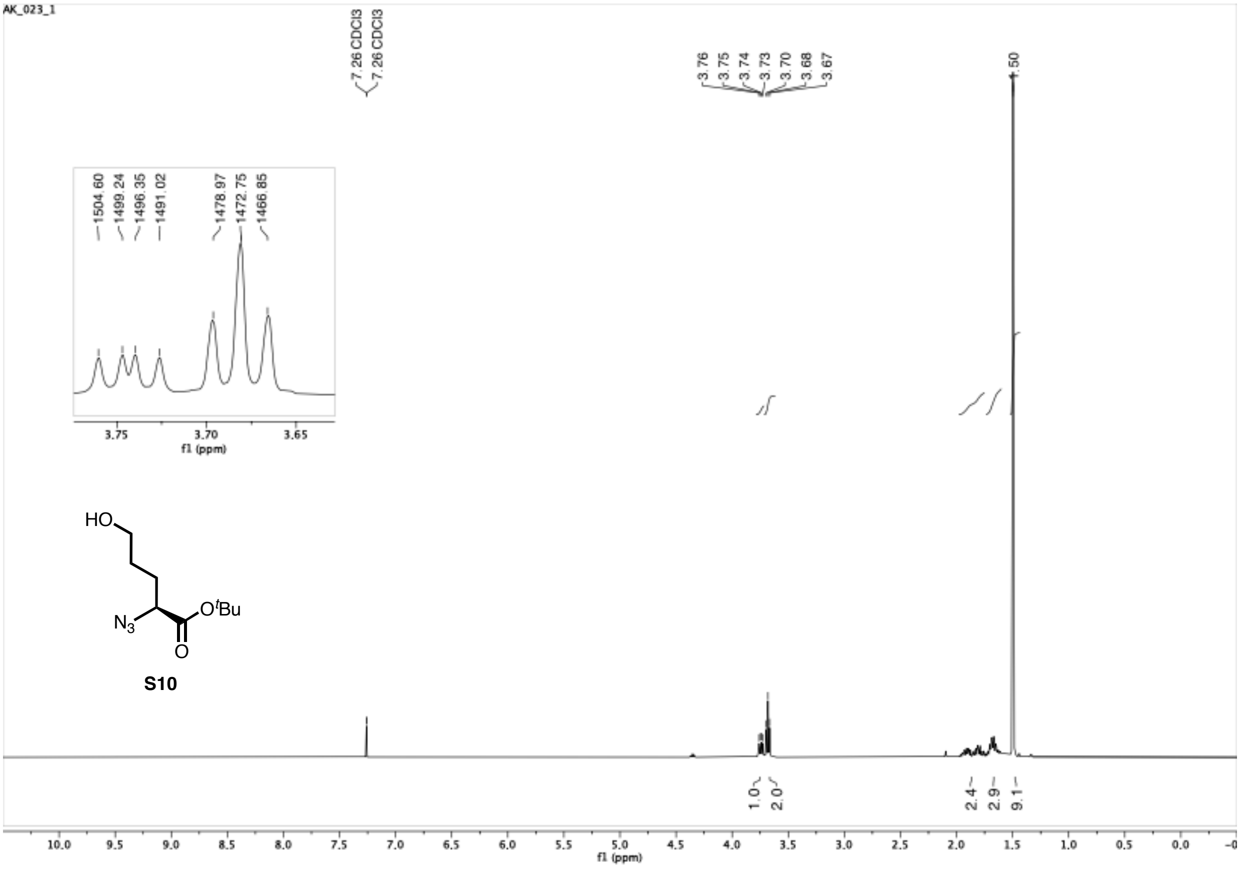

^{13}C{^{1}H}-NMR of **S10**

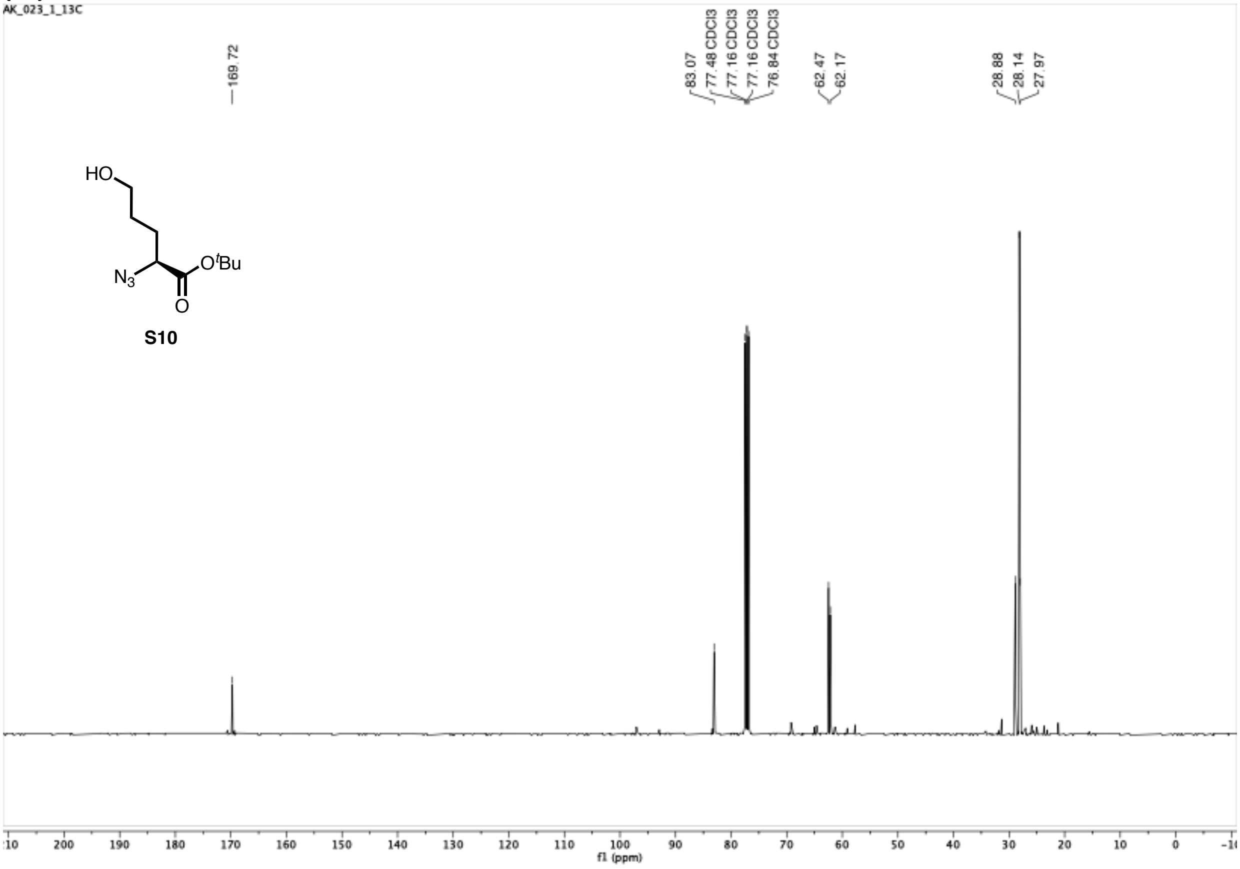

^{1}H-NMR of **S11**

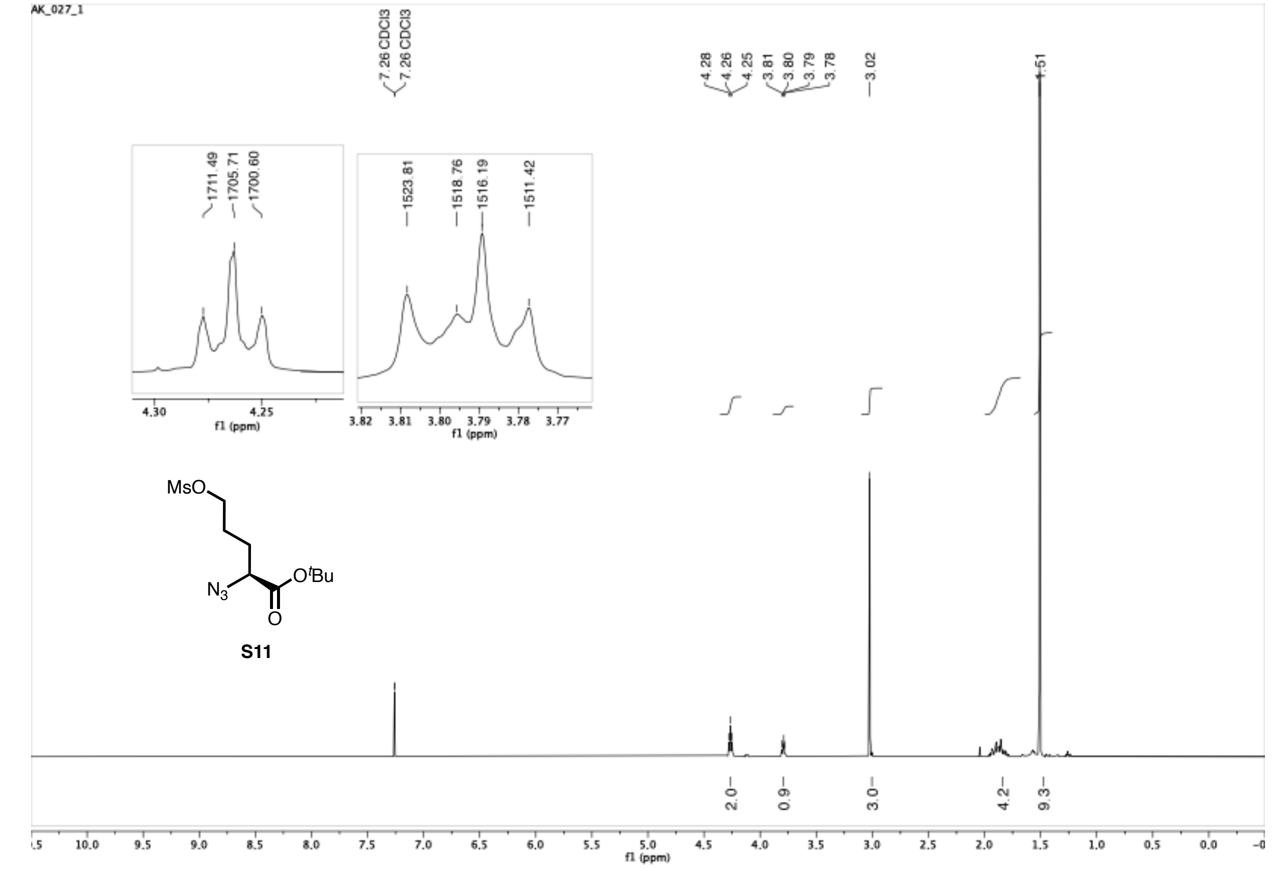

^{13}C{^{1}H}-NMR of **S11**

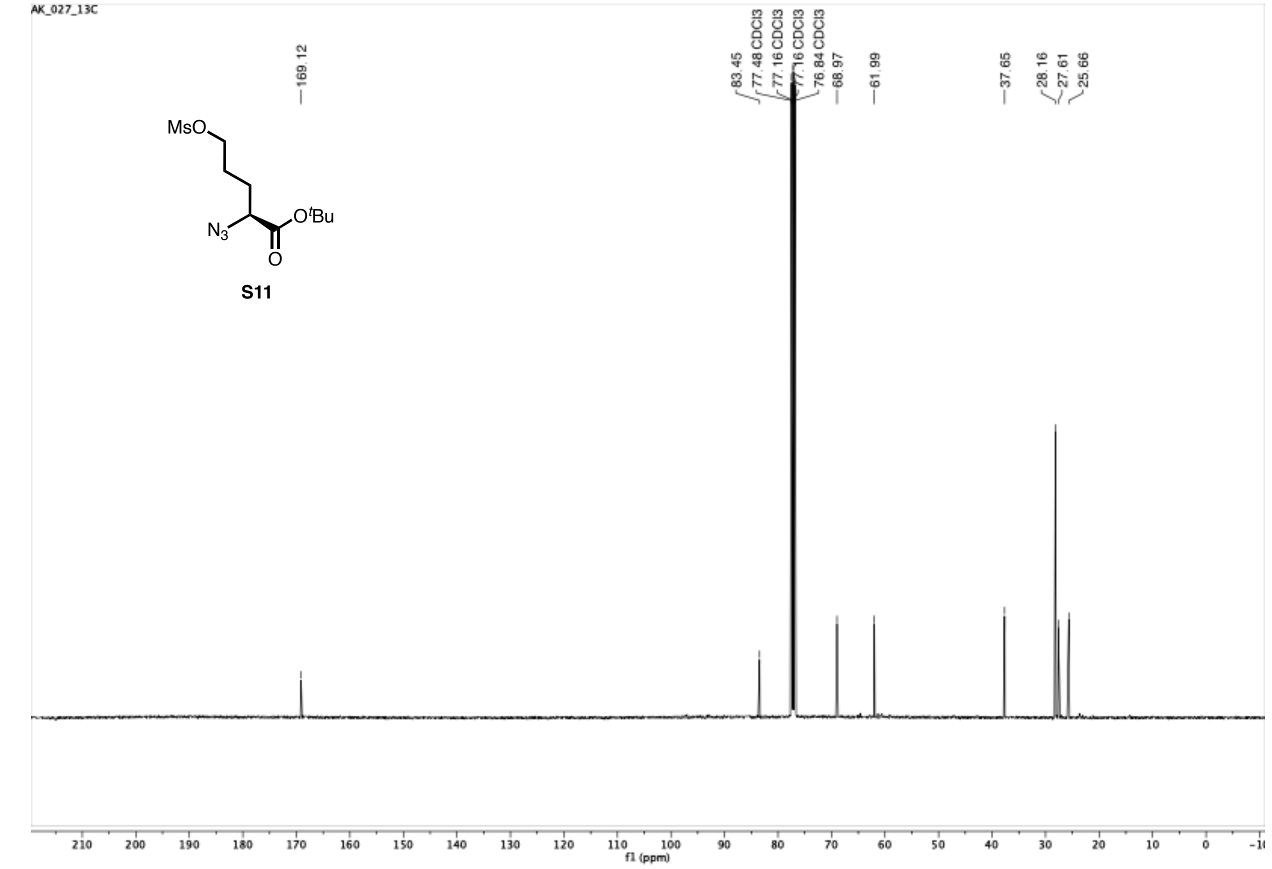

^{1}H-NMR of **S12**

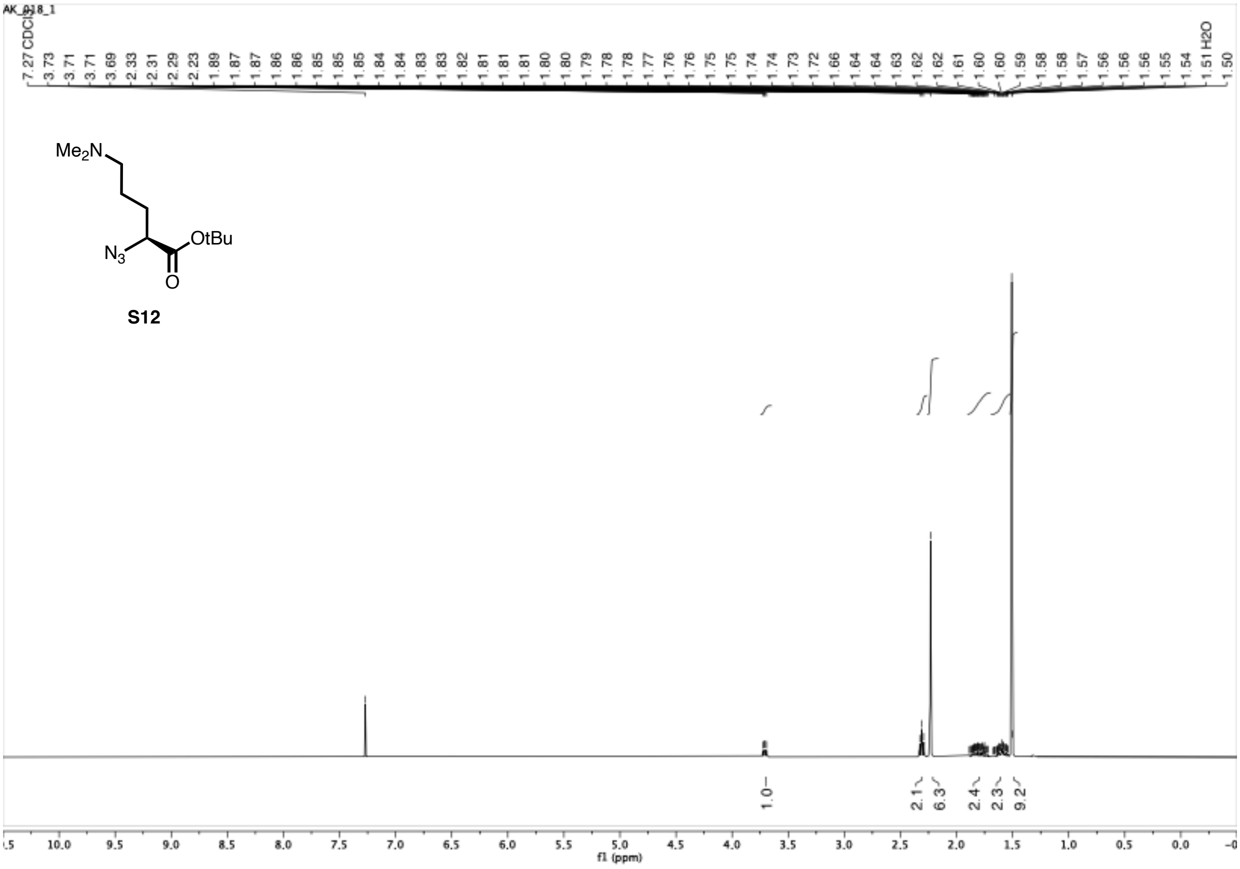

^{13}C{^{1}H}-NMR of **S12**

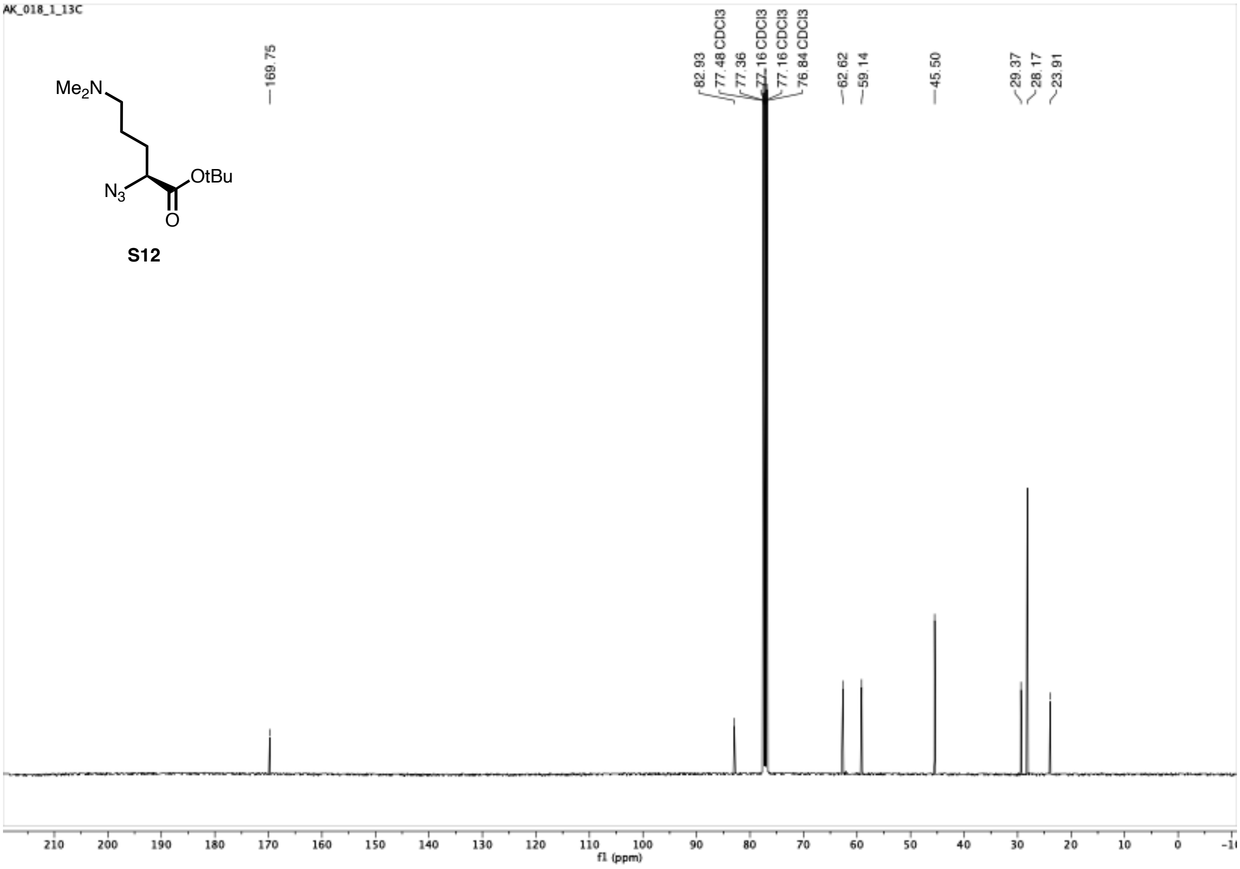

^{1}H-NMR of **S8**

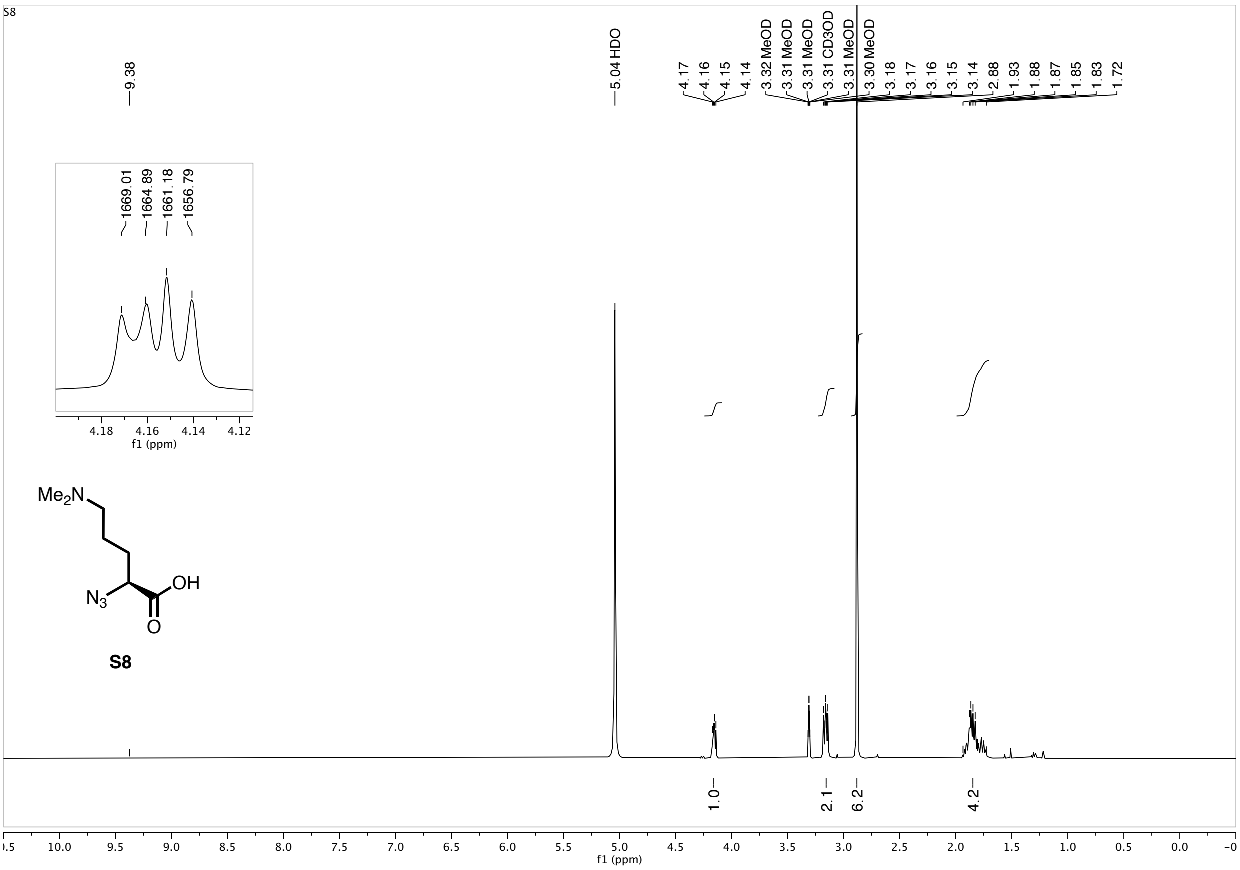
S8
9.38
5.04 HDO
4.17
4.16
4.15
4.14
3.32 MeOD
3.31 MeOD
3.31 MeOD
3.31 CD3OD
3.31 MeOD
3.30 MeOD
3.18
3.17
3.16
3.15
3.14
2.88
1.93
1.88
1.87
1.85
1.83
1.72
1669.01
1664.89
1661.18
1656.79
4.18
4.16
4.14
4.12
f1 (ppm)
Me2N
N3
OH
O
S8
1.0
2.1
6.2
4.2
10.0
9.5
9.0
8.5
8.0
7.5
7.0
6.5
6.0
5.5
5.0
4.5
4.0
3.5
3.0
2.5
2.0
1.5
1.0
0.5
0.0
f1 (ppm)

^{1}H-NMR of **S13**

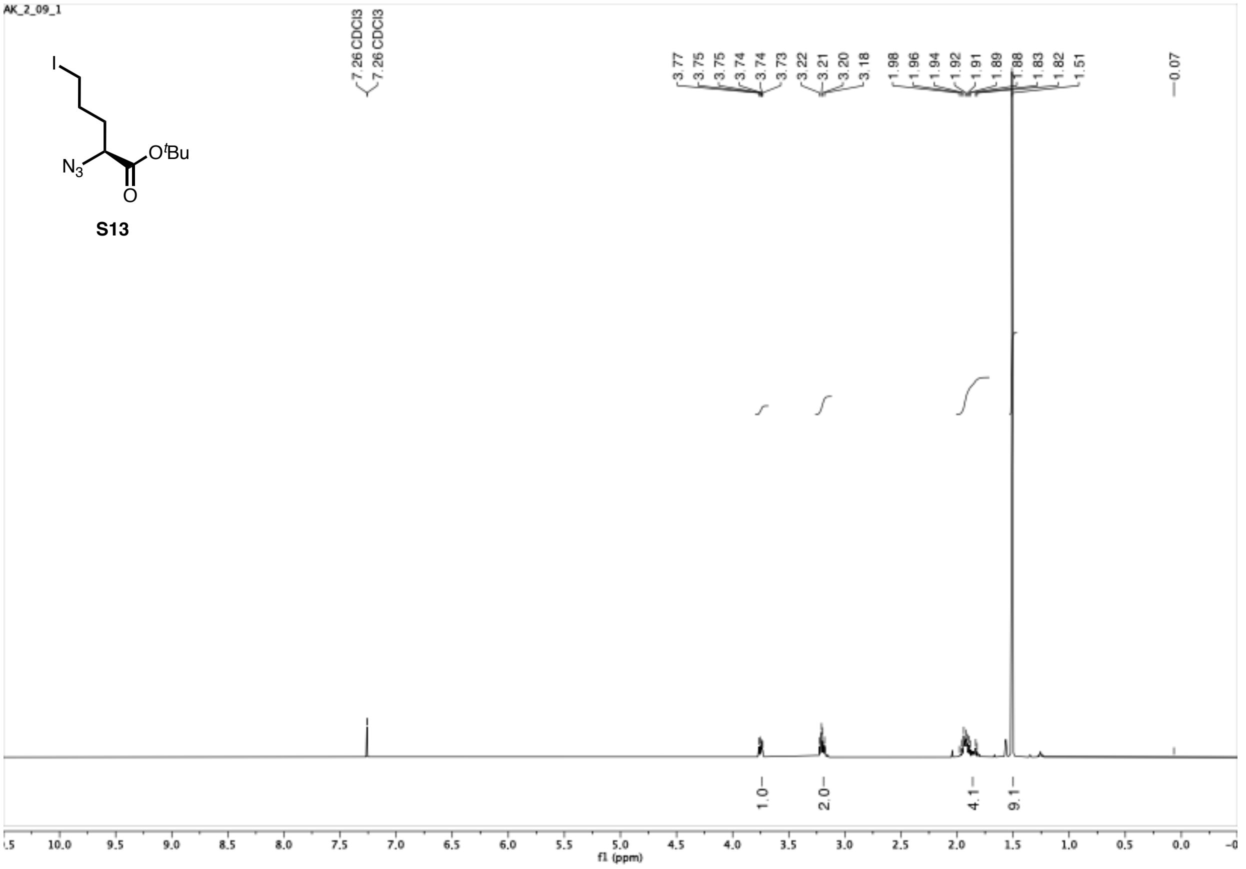

^{13}C{^{1}H}-NMR of **S13**

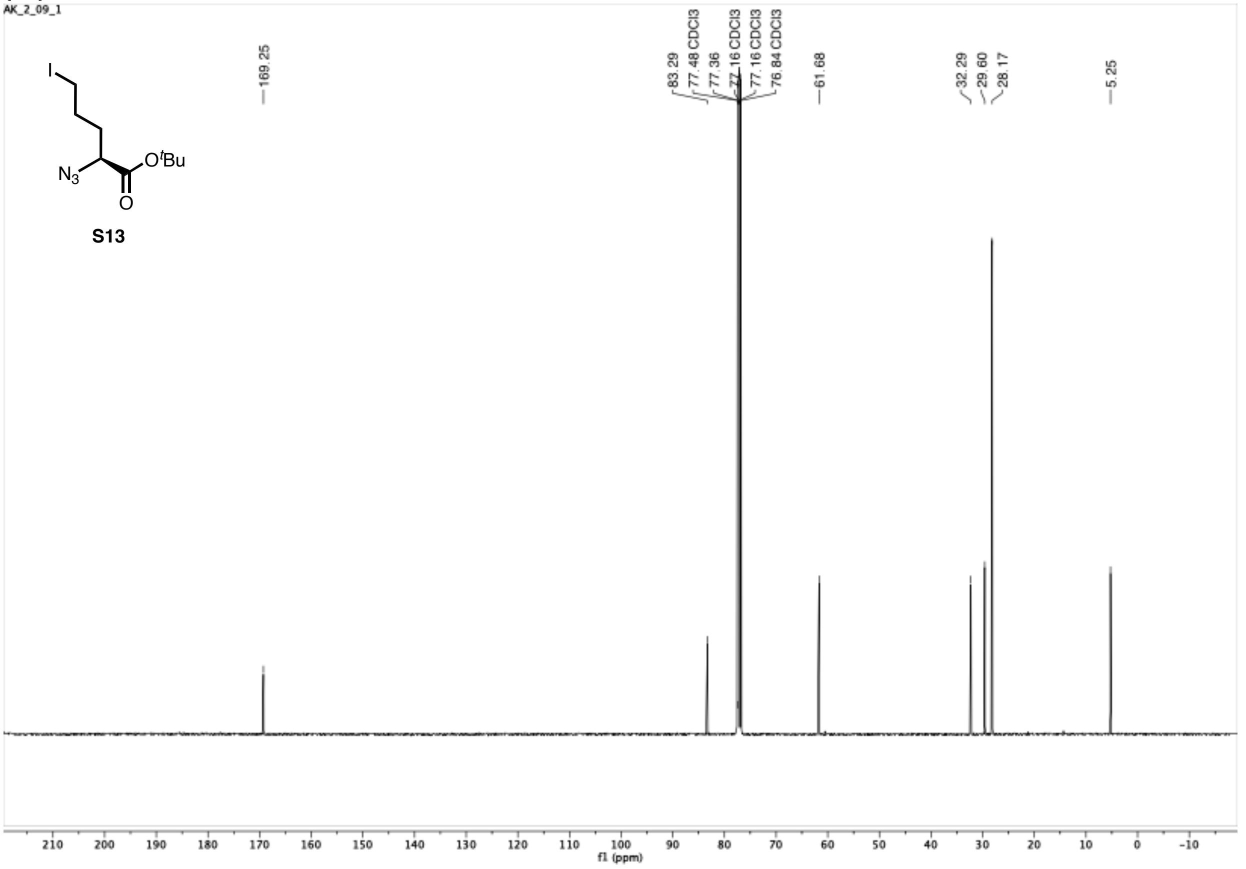

^{1}H-NMR of **S14**

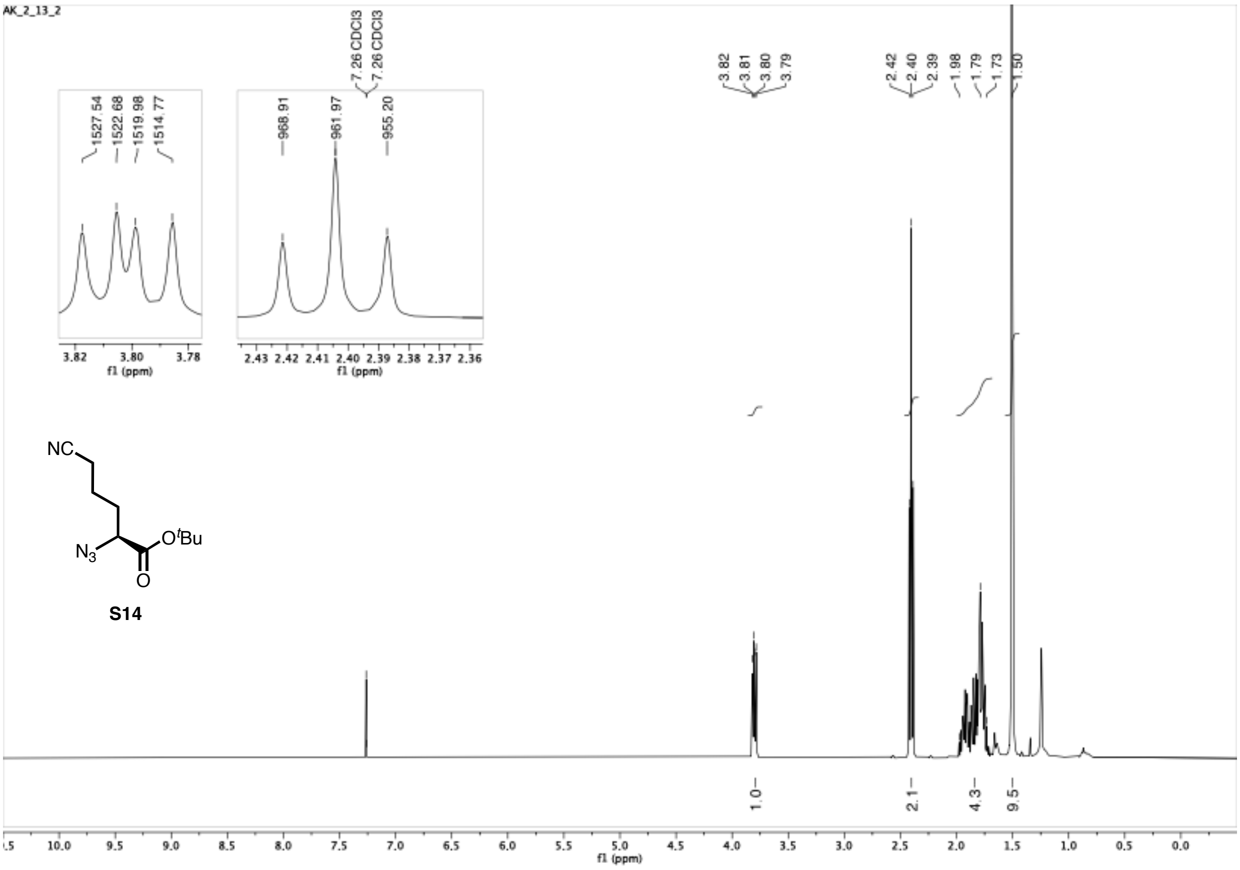

^{13}C{^{1}H}-NMR of **S14**

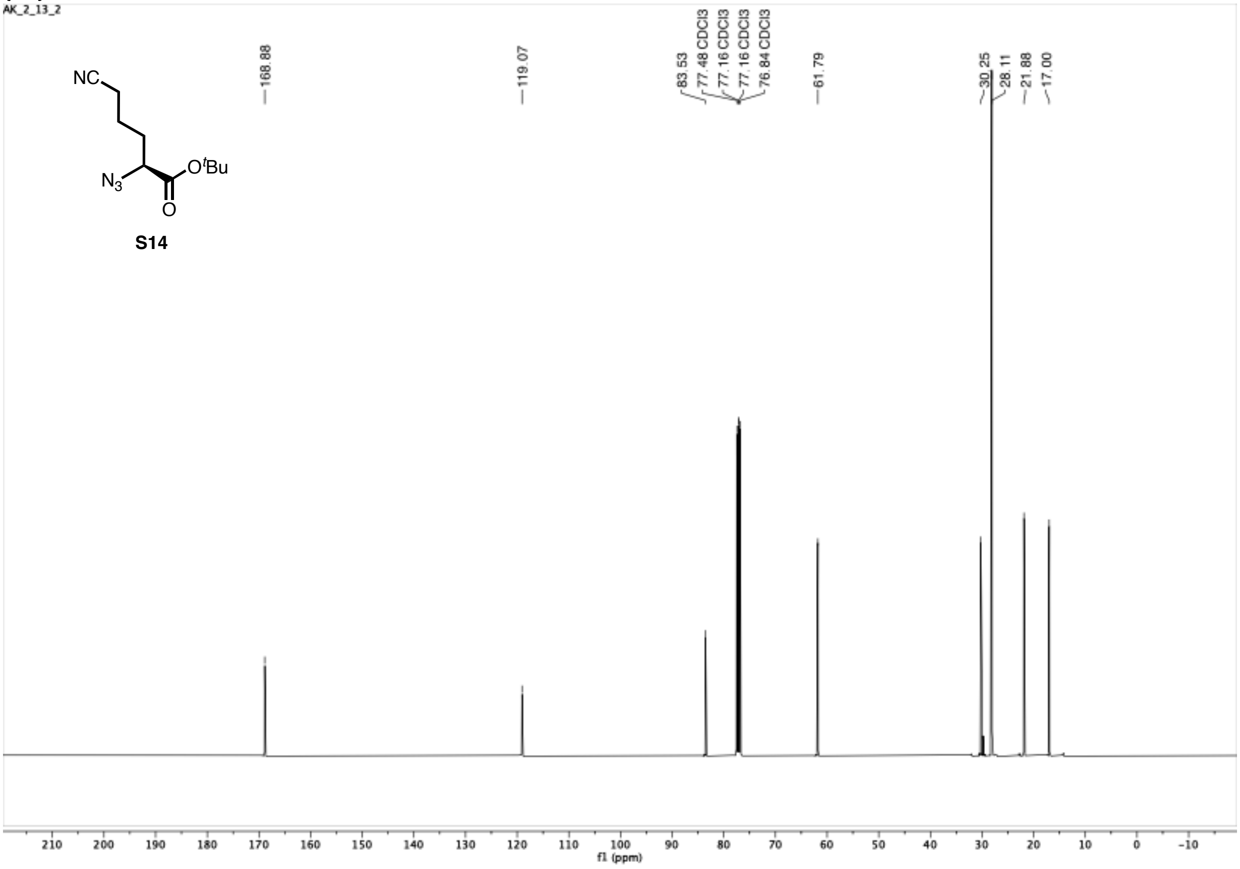

^{1}H-NMR of **S7**

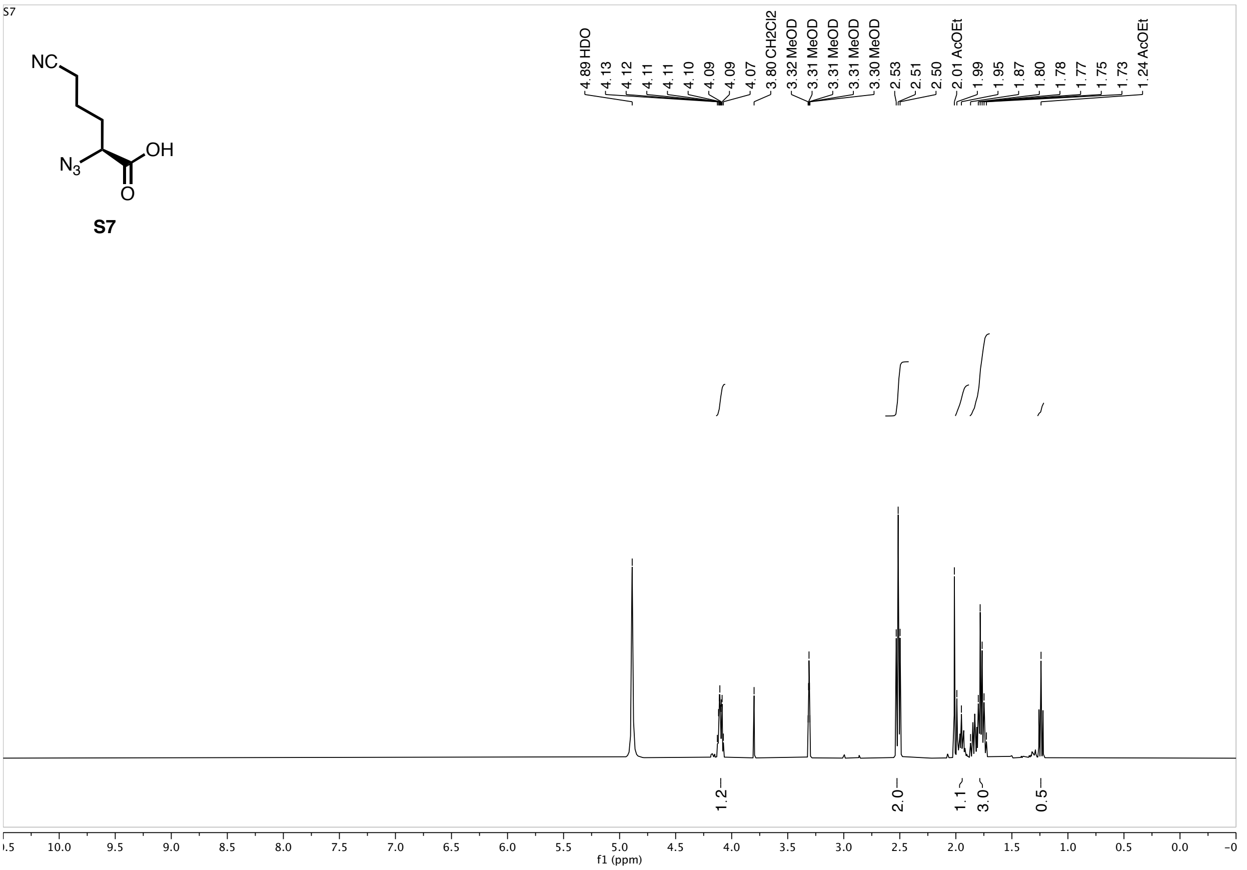
S7
NC
N3
OH
O
S7
4.89 HDO
4.13
4.12
4.11
4.11
4.10
4.09
4.09
4.07
3.80 CH2Cl2
3.32 MeOD
3.31 MeOD
3.31 MeOD
3.31 MeOD
3.30 MeOD
2.53
2.51
2.50
2.01 AcOEt
1.99
1.95
1.87
1.80
1.78
1.77
1.75
1.73
1.24 AcOEt
1.2
2.0
1.1
3.0
0.5
10.0 9.5 9.0 8.5 8.0 7.5 7.0 6.5 6.0 5.5 5.0 4.5 4.0 3.5 3.0 2.5 2.0 1.5 1.0 0.5 0.0
f1 (ppm)

^{1}H-NMR of **S16**

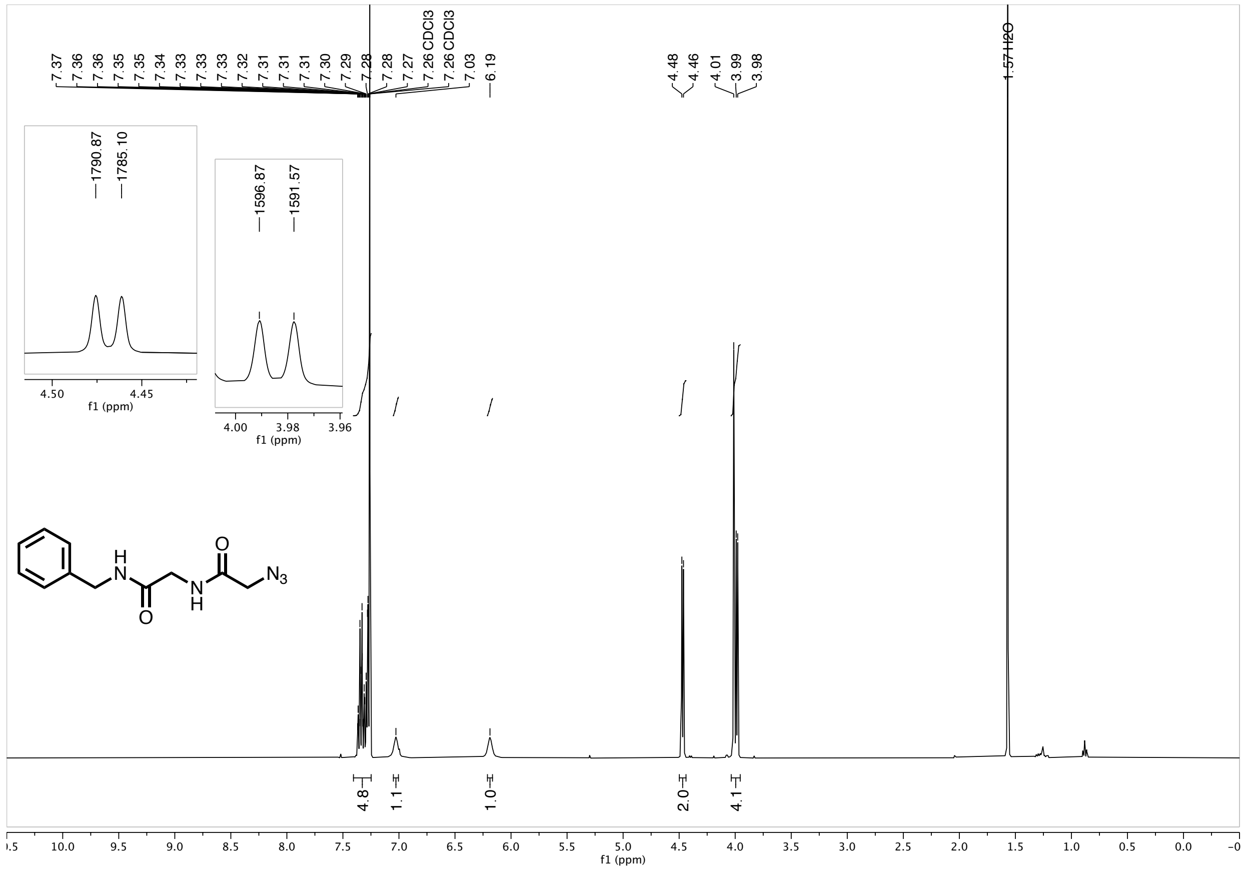

^{13}C{^{1}H}-NMR of **S16**

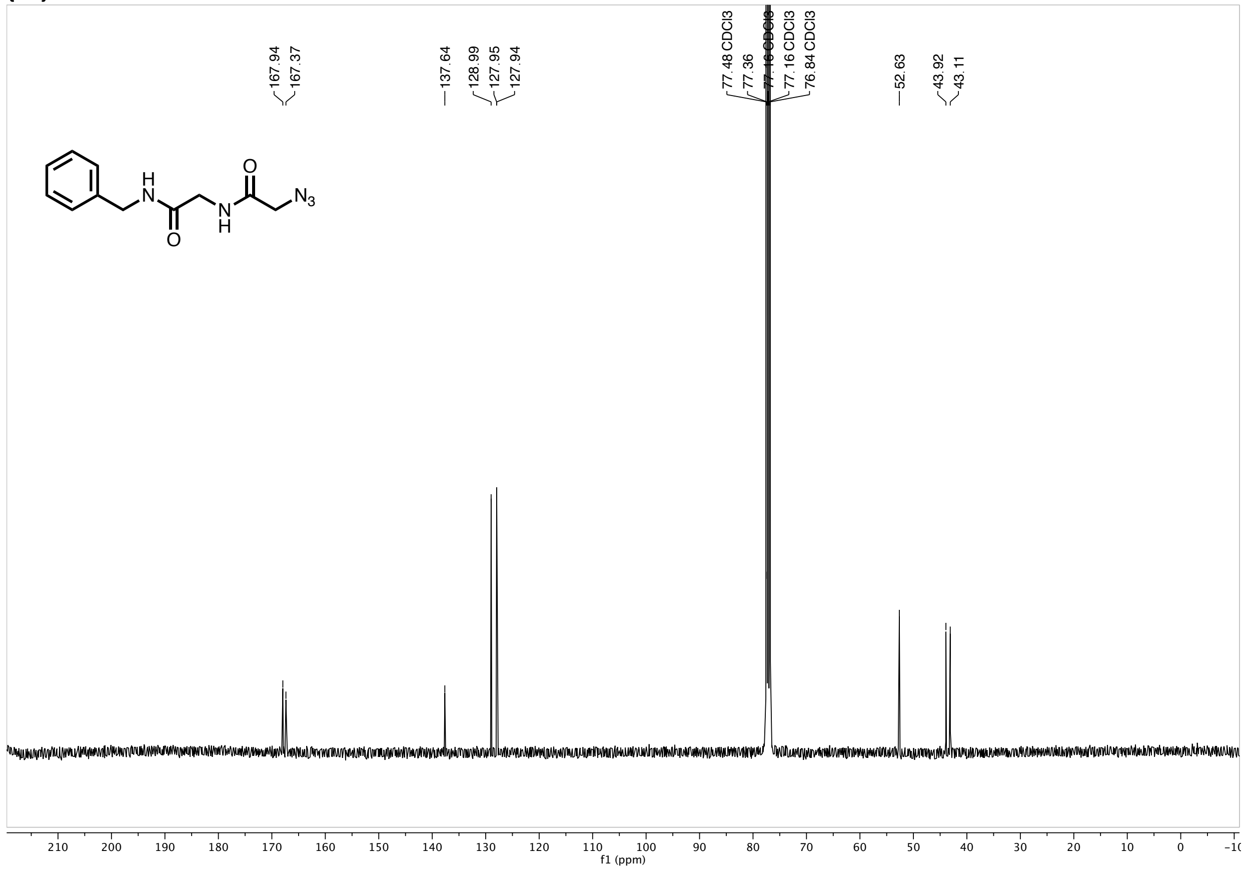

^{1}H-NMR of **S17**

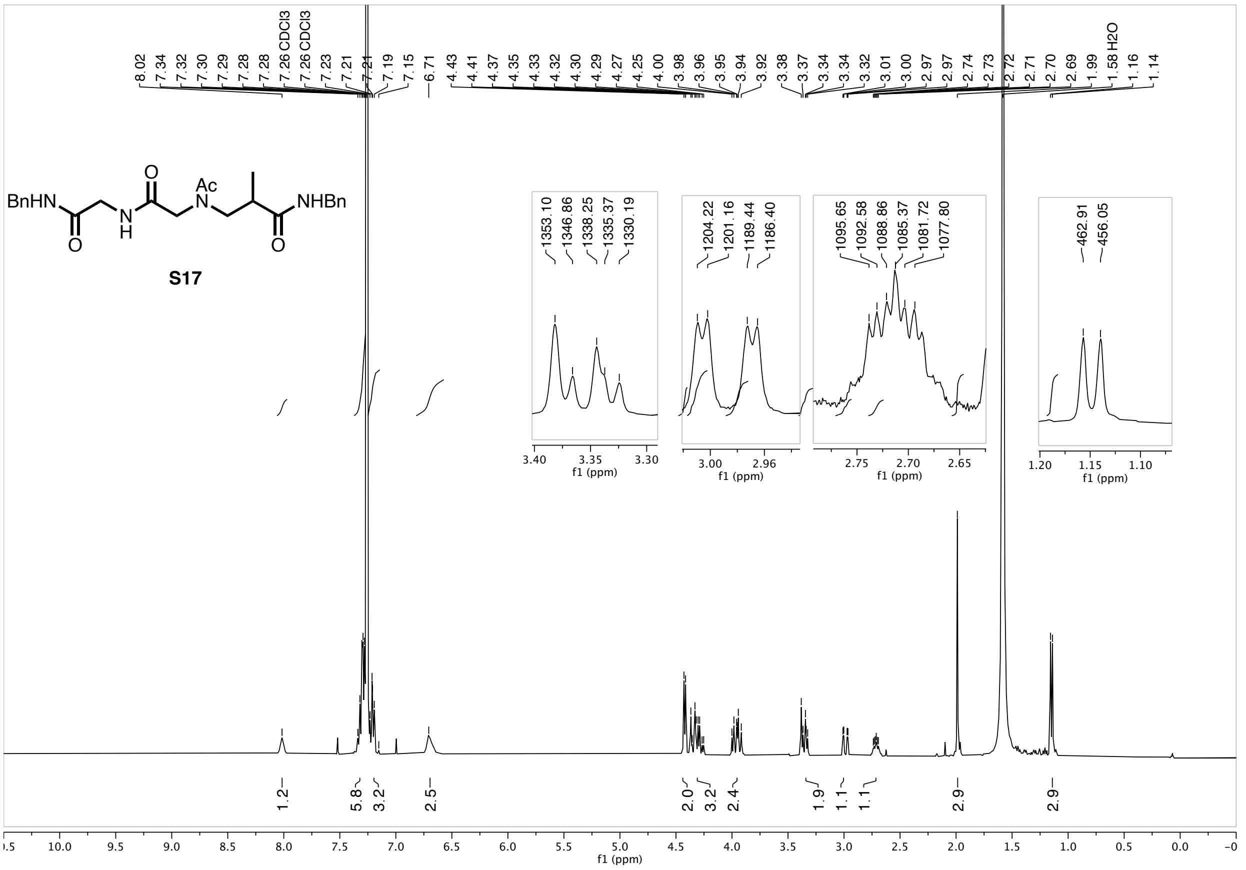

^{13}C{^{1}H}-NMR of **S17**

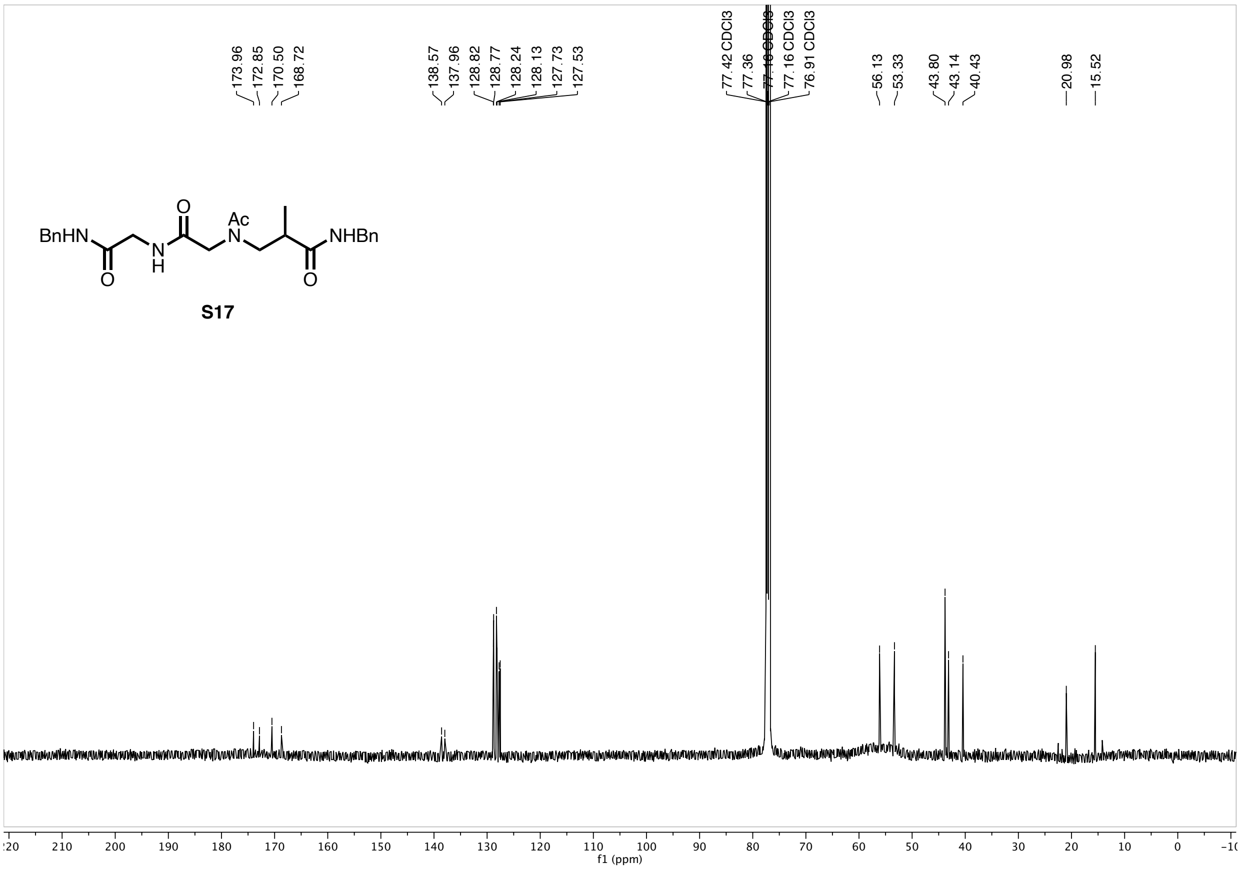

^{1}H-^{1}H COSY of **S17**

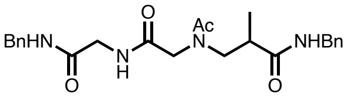

S17

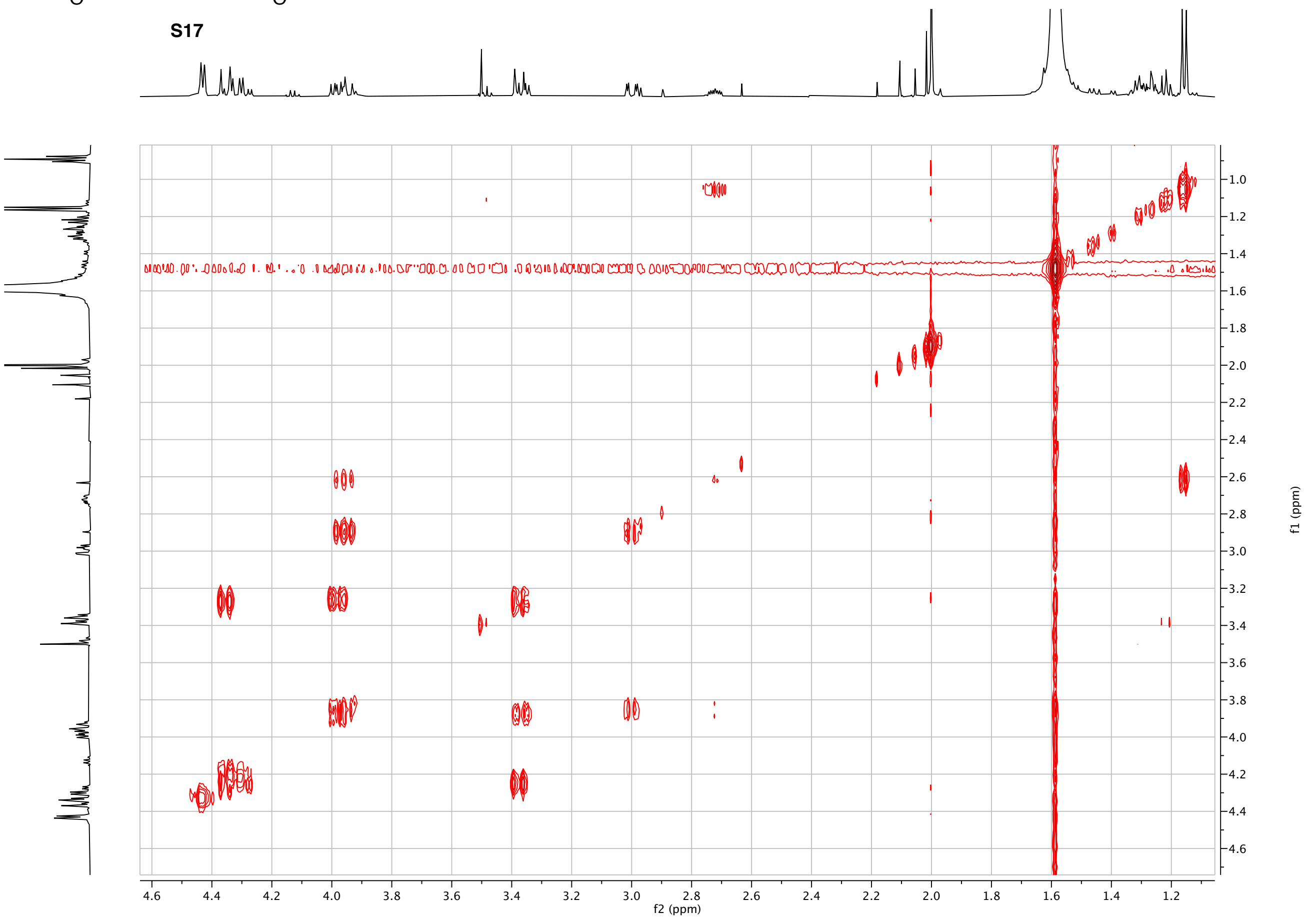

^{1}H-NMR of **3**

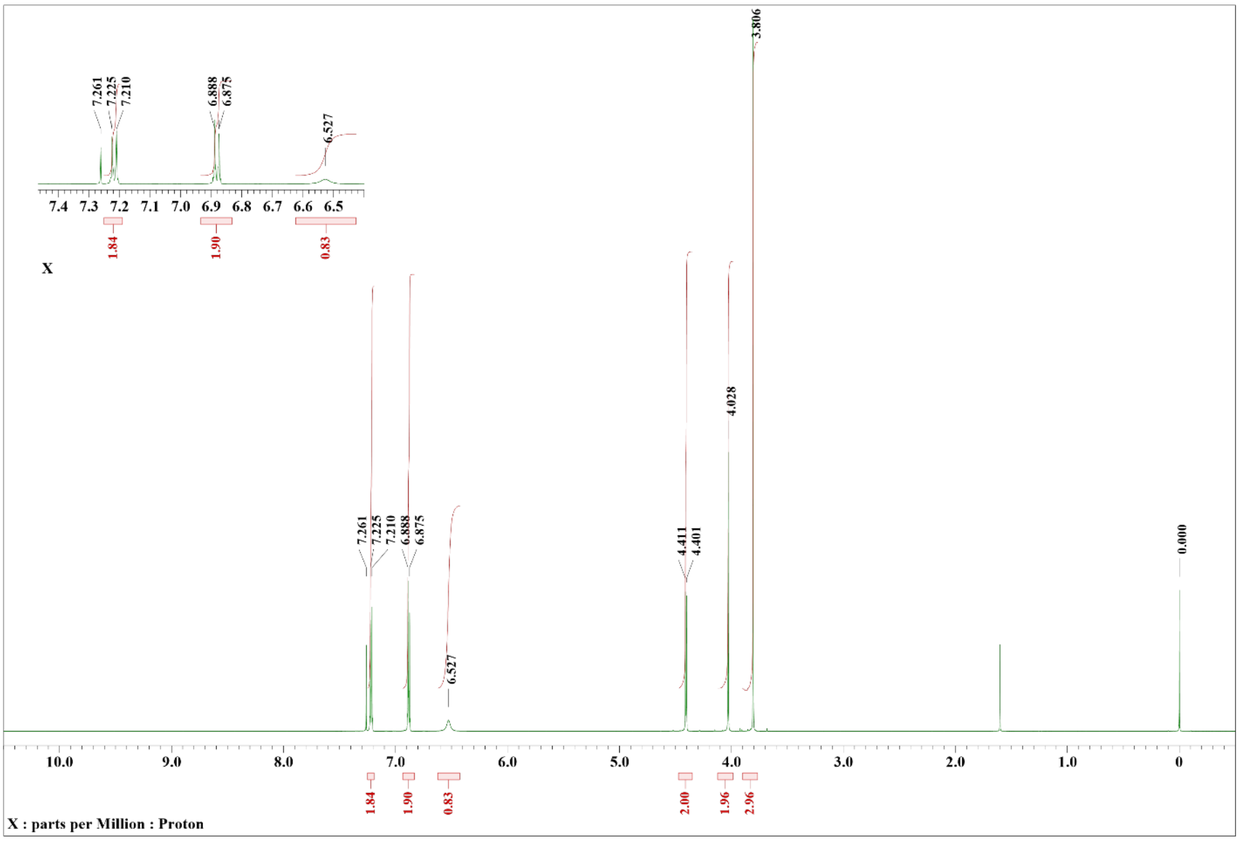

^{13}C{^{1}H}-NMR of **3**

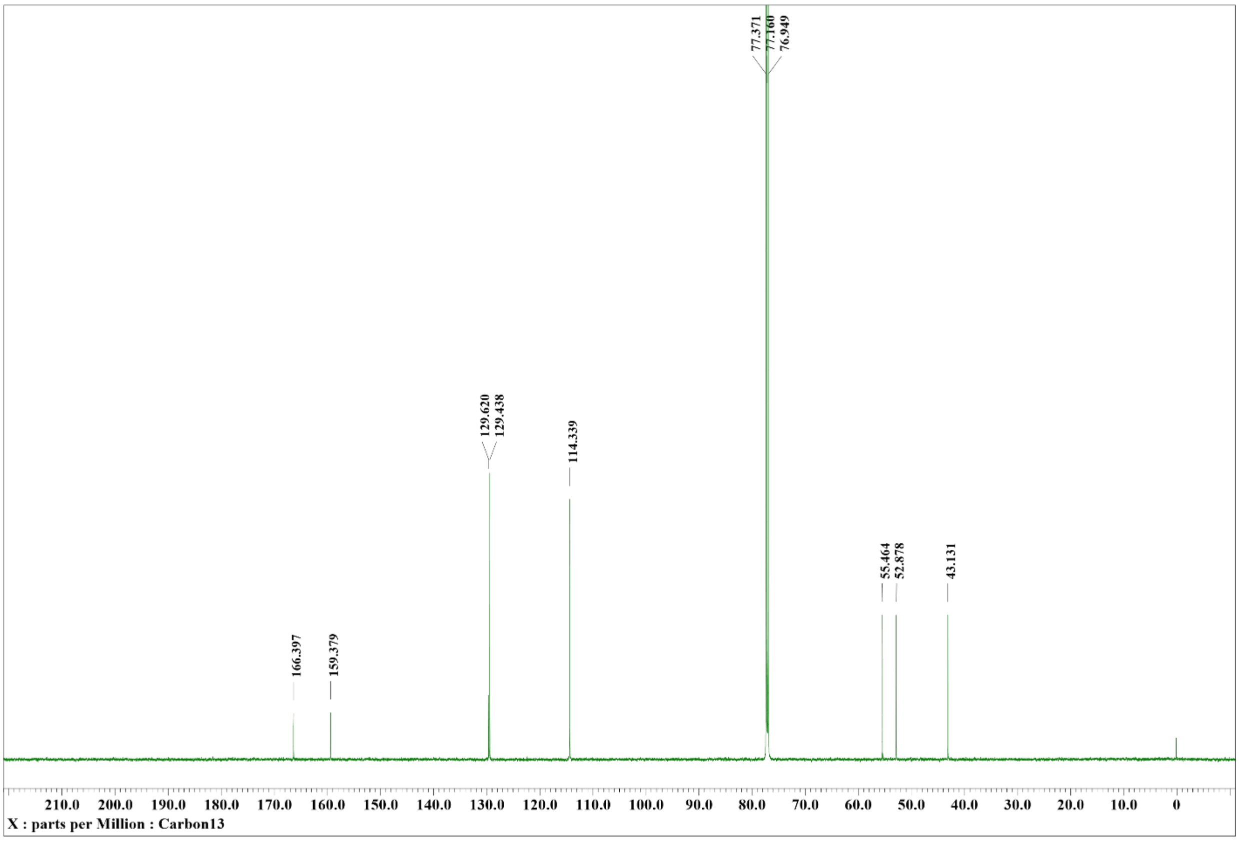

^{1}H-NMR of **8**

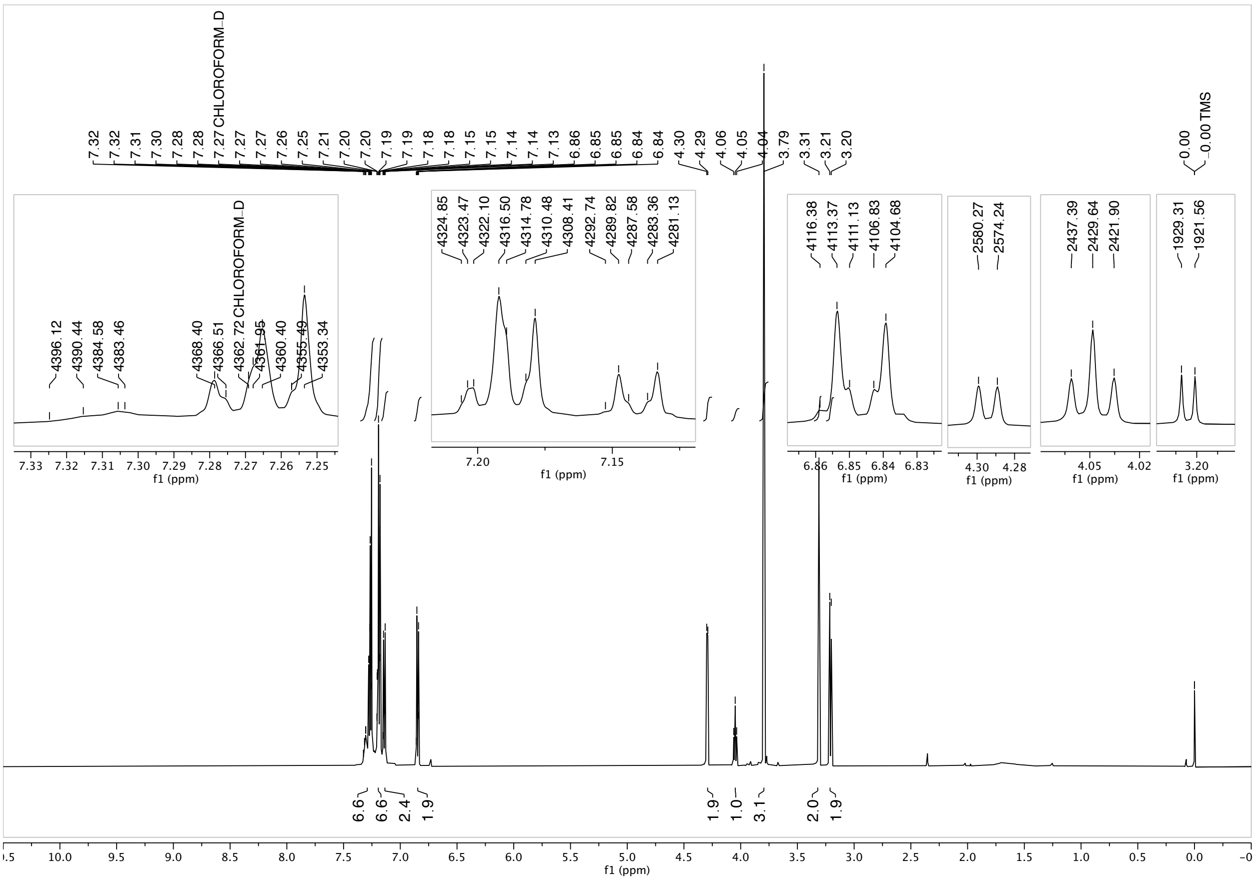

^{13}C{^{1}H}-NMR of **8**

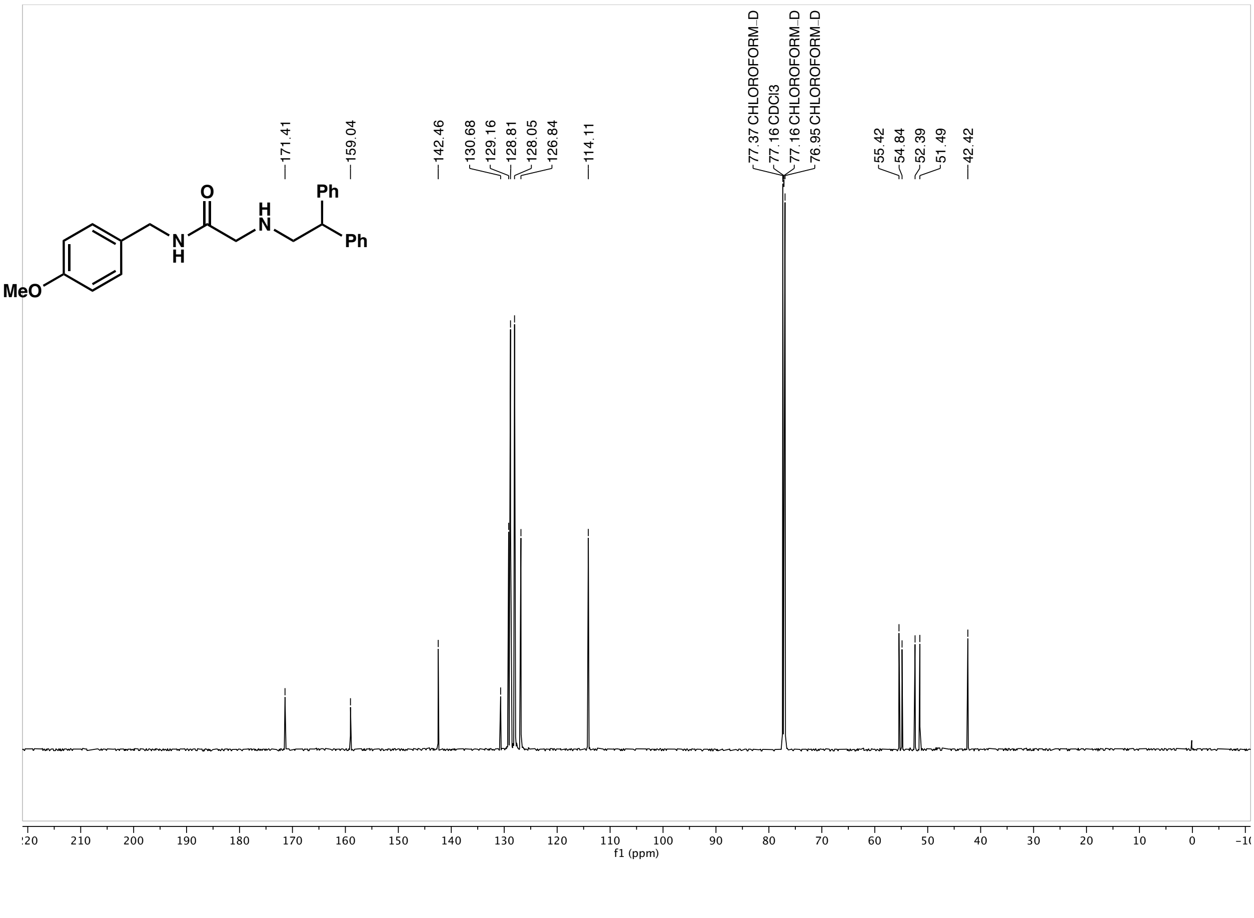

^{1}H-^{1}H COSY of **8**

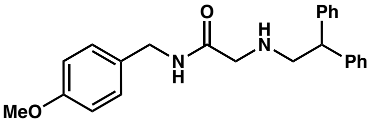

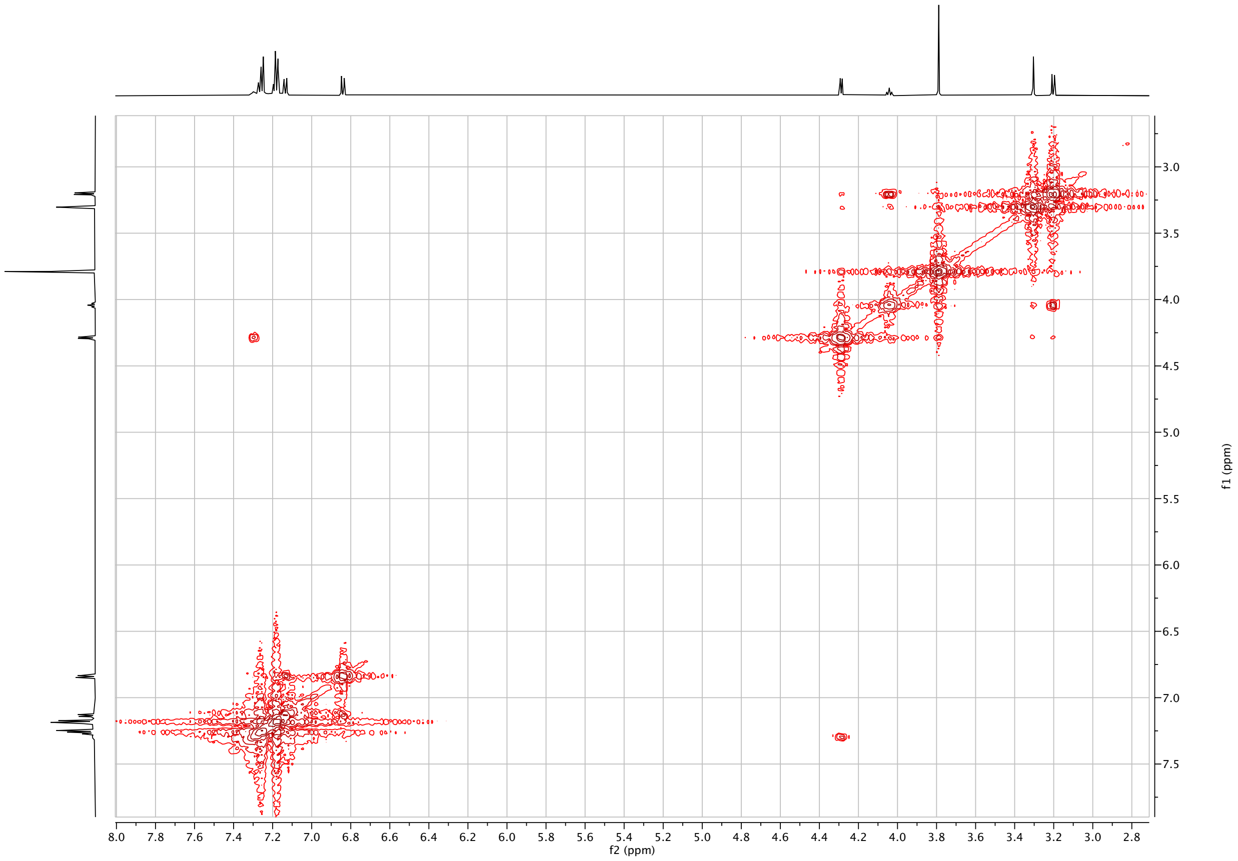

Supplement: SC-017-D5SC08732E-s003 [file SC-017-D5SC08732E-s003.pdf]
